# Development of a High-Throughput Resequencing Array for the Detection of Pathogenic Mutations in Osteogenesis Imperfecta

**DOI:** 10.1371/journal.pone.0119553

**Published:** 2015-03-05

**Authors:** Yao Wang, Yazhou Cui, Xiaoyan Zhou, Jinxiang Han

**Affiliations:** 1 Shandong Academy of Medical Sciences, Shandong Medical Biotechnological Center, Key Laboratory for Biotech Drugs of the Ministry of Health, Ji’nan, Shandong, China; 2 Shandong Institute of Endocrine and Metabolic Diseases, Shandong Academy of Medical Sciences, Ji’nan, Shandong, China; National Eye Institute, UNITED STATES

## Abstract

**Objective:**

Osteogenesis imperfecta (OI) is a rare inherited skeletal disease, characterized by bone fragility and low bone density. The mutations in this disorder have been widely reported to be on various exonal hotspots of the candidate genes, including *COL1A1*, *COL1A2*, *CRTAP*, *LEPRE1*, and *FKBP10*, thus creating a great demand for precise genetic tests. However, large genome sizes make the process daunting and the analyses, inefficient and expensive. Therefore, we aimed at developing a fast, accurate, efficient, and cheaper sequencing platform for OI diagnosis; and to this end, use of an advanced array-based technique was proposed.

**Method:**

A CustomSeq Affymetrix Resequencing Array was established for high-throughput sequencing of five genes simultaneously. Genomic DNA extraction from 13 OI patients and 85 normal controls and amplification using long-range PCR (LR-PCR) were followed by DNA fragmentation and chip hybridization, according to standard Affymetrix protocols. Hybridization signals were determined using GeneChip Sequence Analysis Software (GSEQ). To examine the feasibility, the outcome from new resequencing approach was validated by conventional capillary sequencing method.

**Result:**

Overall call rates using resequencing array was 96–98% and the agreement between microarray and capillary sequencing was 99.99%. 11 out of 13 OI patients with pathogenic mutations were successfully detected by the chip analysis without adjustment, and one mutation could also be identified using manual visual inspection.

**Conclusion:**

A high-throughput resequencing array was developed that detects the disease-associated mutations in OI, providing a potential tool to facilitate large-scale genetic screening for OI patients. Through this method, a novel mutation was also found.

## Introduction

Osteogenesis imperfecta (OI; MIM 166200, 166210, 259420, and 166220; also known as brittle bone disease) is a rare genetic skeletal disease, with a prevalence of 1/25,000–1/15,000. It is evidenced by the formation of collagen and mesenchymal dysgenesis[1,2]. OI is inherited mostly in a dominant pattern; however, a small number of families show autosomal recessive inheritance[3]. The OI patients exhibit various clinical features, including easy bone fracture, blue sclera, dentin hypoplasia, hearing loss, and scoliosis[4,5]. Genetic test has been the gold standard for diagnosing OI, thereby allowing an early intervention in preventing or delaying the development of the disease. Till date, more than 1000 different mutations have been identified in patients with OI, and these mutations are distributed in over ten different genes, such as *COL1A1*, *COL1A2*, *CRTAP*, *LEPRE1*, and *FKBP10*. Since the classical genetic analysis methods, like capillary sequencing, are time-consuming and expensive, it necessitates the development of an efficient customized sequencing platform in order to perform genetic sequence analysis more rapidly, and accurately, and simultaneously for multiple genes. The present study was undertaken to take advantage of the microarray resequencing technique that was recently applied in cancer and mitochondrial diseases[6–8], and utilize it for the investigation of genetic mutations in OI patients.

## Materials and Methods

### Samples

13 unrelated OI patients between two to six years of age, with typical clinical OI features, and 85 control subjects with no obvious bone disease were enrolled in the study, under the approval of Institute of Review Board (IRB) of Shandong Academy of Medical Sciences, China. A signed consent form was obtained from all the subjects and their families. In addations, the patients had no previous molecular analysis

### Resequencing Array design

**Fig 1:**
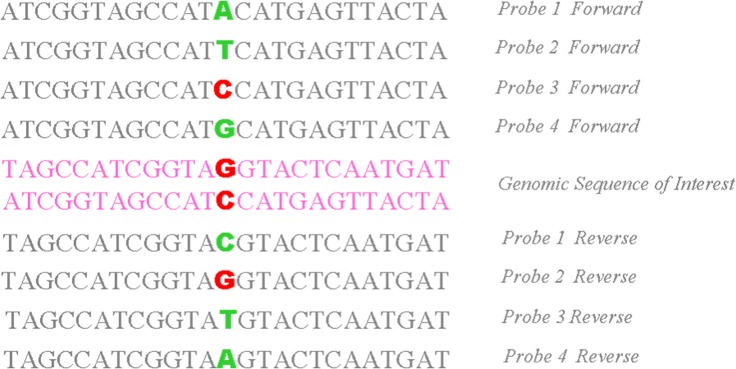
Principle of OI array analysis. Eight probes (four each for sense and antisense strands) associated with every queried site were used. Each probe consisted of a 25-base oligonucleotide, while the 13th base was different among the probes to cover all potential nucleotide mutations.

Most of the disease-causing mutations are located on the coding sequences of *COL1A1*, *COL1A2*, *CRTAP*, *LEPRE1*, and *FKBP10*[9]. Therefore, the sequencing of all the exons, 12 flanking base pairs of the splice junctions (potential splice sites), and 280–500 bases upstream from the first exon of these genes was emphasized in the present study. The microarray platform was adopted with GeneChip CustomSeq Custom Resequencing Array (OI array; Affymetrix, Santa Clara, CA, USA), tiled with 29,907 bases of target sequences[10]. In order to optimize the mutation detection efficiency, the OI array was divided into 2 sections: one group was primarily used for the detection of single nucleotide variations, while the other part was utilized for the detection of 173 previously reported insertions/deletions[11]. Both sense and antisense DNA strands were interrogated by sets of 25-mer probes containing a one-base difference at the 13^th^ position (A, C, G, and T) (Fig. 1).

### DNA extraction and PCR amplifications

Genomic DNA was extracted from 2 ml peripheral blood drawn from OI patients and control group using a Blood DNA Midi Kit (OMEGA, CA, USA). Due to the limited amount of isolated DNA, 22 LR-PCR primers targeting the candidate sequences were designed to amplify DNA for sufficient labeling and hybridization on OI array with an average amplification ranging from 5 kb to 6 kb. The PCR was performed using TaKaRa LA PCR kit, V2.1 (TaKaRa Mirus Bio, Madison, WI, USA), under the following cycling conditions: 95°C for 1 min, followed by 30 cycles of 95°C, 30 sec; 60°C, 30 sec; and 72°C, 30 sec; and final extension at 72°C for 10 min. A 7.5-kb Taq IQ-EX was amplified as a positive-control for each hybridization.

### Quantitation and pooling

Residual primers and nucleotides were purified from PCR amplifications using QIAquick PCR Purification Kit (Qiagen Inc., Valencia, CA, USA) and DNA concentration was measured using spectrophotometer (NanoDrop2000). Equimolar amounts of the 22 PCR amplified products were pooled into a tube as one sample to ensure equivalent hybridization; and the sample was further processed using the GeneChip Resequencing Assay Kit.

### Fragmentation, labelling, hybridization, washing, and staining

**Fig 2:**
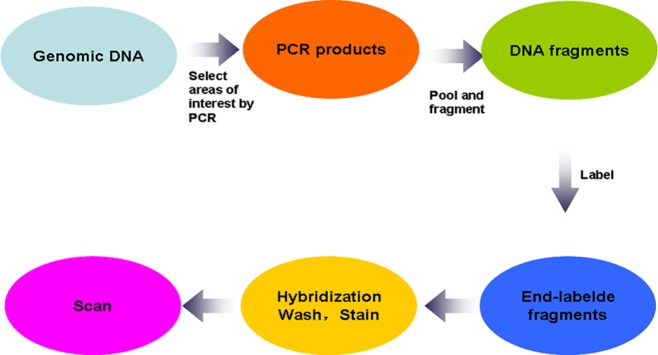
Procedures for OI microarray resequencing.

DNA fragmentation, labeling, hybridization, washing, and staining of the arrays were carried out according to the GeneChip CustomSeq Resequencing Array Protocol Version2.1 (http://www.affymetrix.com/support/downloads/manuals/customseq_prot ocol.pdf). In brief, approximately 20–200 bp sized fragments were generated from the pooled DNA using GeneChip Fragmentation Reaction Kit. The operation was followed by PCR at 37°C for 35 min, and 95°C for 15 min. Every reaction mixture contained an enzyme to fragment the amplicons. Subsequently, the fragmented DNA was labeled using Biotin and TDT enzyme at 37°C × 2 h, 95°C × 15 min by PCR. Prior to hybridization, the chip was incubated with 80 μl pre-hybridization buffer in a hybridization oven with 60 g/min for 15 min at 49°C. Then, the buffer was removed and 80 μl of hybridization solution (Tris, pH 7.8; Tween-20; acetylated BSA; herring sperm DNA; labeled oligonucleotide control reagent; and labeled fragments of amplicons) was added to the same chip. Following incubation with 60g/min for 16 hours at 49°C, the chip was washed with decreasing concentrations of SSPE and Tween-20, stained with anti-biotin antibody, and scanned with the Affymetrix GeneChip 3000 Scanner (Fig. 2).

### Data interpretation

**Fig 3:**
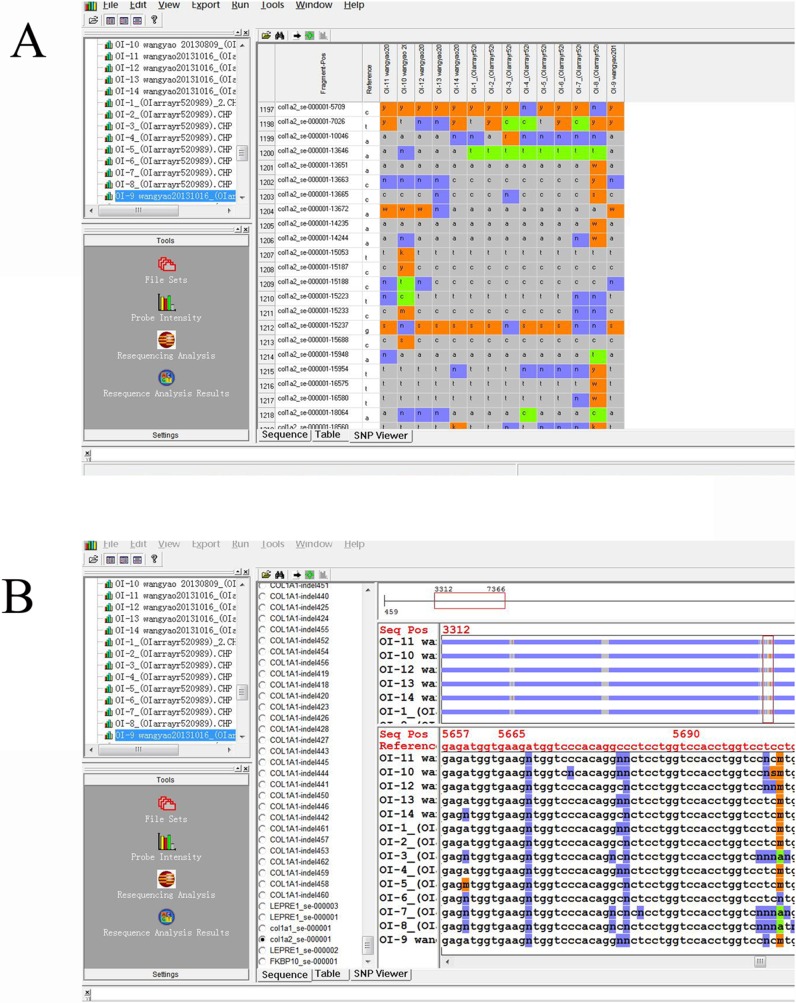
Variants identification, selection, filtering. (A)The name of the fragment, the position of the altered nucleotides and the reference nucleotides are signed on the left side. The altered nucleotides of the samples are itemized. The sign N (blue) corresponds to the intensity of the signal which did not allow for a specific base call. The signs y, r, k, etc. (orange) indicate a nucleotide change in the heterozygous state. Signs a, t, c or g (green) denote a nucleotide change to homozygous A, T, C or G. (B) Sequence output files of samples. Part of the sequence containing nucleotide alterations is shown. Reference sequence and positions of nucleotides are shown in red at the top. The signs are identical to those in image (A).

Analysis of the scanned data was performed using GeneChip Sequence Analysis Software (GSEQ) ver. 4.1 (Affymetrix, Santa Clara, CA, USA). The background noise was normalized by the signals of sterile water hybridized array[12]. The resequencing calling algorithm used was based on the adaptive background calling scheme (ABACUS) developed by Cutler *et al*[13]. This algorithm defined 11 genotypes: A, C, G, T, AC, AG, AT, CG, CT, GT, and “no call”. A minimum of 11 samples analyzed together on GSEQ obtains a robust base call, while more than 15 samples are considered to be an optimal sample size for analysis[14]. Probe intensities of A, C, G, and T of both strands were displayed in a tabular format along with the quality scores and in the form of an intensity plot. Variant calls and “no calls” were verified by examination of the probe intensities(Fig. 3).

## Result

### Evaluation of accuracy of OI Array

One of the unique characteristics of the OI array is that the targeted sequences are not always contiguous. This feature allowed us to select the candidate sequences or bases for further investigation. For the 29,907 bases tiled on the chip, the mutations and insertions/deletions that might alter protein structure/function were verified. To evaluate the efficiency of OI array for the identification of target genes, the nucleotide sequences for *COL1A1*, *COL1A2*, *CRTAP*, *LEPRE1*, and *FKBP10* were processed as reference readouts. According to hybridization intensities, GSEQ software could specifically define the target nucleotide sequence. For quality control (QC), the base-call rate and ref-call rate were evaluated. The base-call rate is defined as the fraction of individual bases that can be clearly detected and identified, while the ref-call rate is defined as the fraction between the called sequences and the reference sequences[15]. It has been found that higher rates of base-call and ref-call are positively correlated with the quality analysis of the assay. For the optimization of data analysis, parameters for GSEQ are shown in the following: AberrantSNR2 20; NoSignal 2; Model Type 0; QualityScore 4; SampleReliability 0.5; SeqProfile Threshold-0.175; Trace Threshold 1; WeakSignal 20. Our analysis of 98 test samples on OI chips showed a 97.01% (with a range of 96.10% to 98.20%) call rate and more than 99% ref-call rate. These results were consistent with that of a previous study, which reported >90% base-call rate and >99% ref-call rate[16]. For the 13 OI patients, the call rate ranged from 96.30% to 97.60% (Table 1), while the “no calls”, accounting for the remaining 3–4%, probably resulted from the compensation of background and signal intensity, as the signal intensity and the background are indistinguishable from the background value[17,18]. It was observed that most “no calls” were located at a region in the sequence with greater GC content. Similar results have also been reported by Booij *et al*[19].

**Table 1:** Quality control data of 5 OI-associated genes using chip analysis.

Patient	*COL1A 1*		*COL1A2*		*LEPRE1*		*FKBP10*		*CRTAP*		Average Callrate
	CallRate	RefCalls	CallRate	RefCalls	CallRate	RefCalls	CallRate	RefCalls	CallRate	RefCalls	
**1** ^**#**^	97.3%	99.9%	97.00%	100.00%	96.80%	99.80%	96.50%	99.80%	97.30%	100.00%	96.98%
**2** ^**#**^	97.00%	100.00%	97.10%	99.90%	96.70%	99.70%	96.60%	100.00%	97.30%	100.00%	96.82%
**3** ^**#**^	97.20%	100.00%	97.00%	99.90%	96.70%	99.70%	97.60%	99.70%	97.20%	100.00%	97.14%
**4** ^**#**^	97.30%	100.00%	97.10%	100.00%	96.70%	99.80%	96.60%	99.70%	97.30%	99.90%	97.00%
**5** ^**#**^	96.30%	100.00%	97.00%	100.00%	96.70%	99.80%	96.50%	99.80%	97.30%	99.90%	96.76%
**6** ^**#**^	96.30%	100.00%	97.30%	99.90%	97.10%	99.70%	97.60%	100.00%	97.10%	100.00%	97.08%
**7** ^**#**^	97.30%	100.00%	97.20%	99.90%	96.80%	99.80%	96.60%	99.70%	97.10%	99.90%	97.00%
**8** ^**#**^	97.00%	99.90%	96.40%	99.90%	96.60%	99.70%	96.90%	99.90%	97.80%	100.00%	96.94%
**9** ^**#**^	97.10%	100.00%	97.10%	99.90%	96.70%	99.80%	97.50%	100.00%	97.30%	100.00%	97.14%
**10** ^**#**^	97.30%	99.90%	97.10%	100.00%	97.10%	99.80%	97.40%	99.70%	97.20%	100.00%	97.22%
**11** ^**#**^	97.20%	100.00%	97.10%	100.00%	96.70%	99.70%	97.30%	99.80%	97.30%	99.90%	97.12%
**12** ^**#**^	97.30%	100.00%	97.20%	99.90%	96.90%	99.70%	97.60%	100.00%	97.30%	99.90%	97.26%
**13** ^**#**^	97.10%	99.90%	96.30%	99.90%	96.80%	99.80%	96.90%	100.00%	97.30%	99.90%	96.88%

To determine the sequencing accuracy detected by OI array, we compared the readouts from chip arrays to the results produced by standard capillary sequencing. Four to five randomly-chosen Exons were used as reference genes and most of the base calls identified by OI array were validated using capillary sequencing. The data showed an accuracy of more than 99.99% between the two methods, thereby suggesting a great coordination between array-based and capillary-mediated sequencing analyses (*P* < 0.05).

### Detection of disease-causing mutations using OI Array

**Fig 4:**
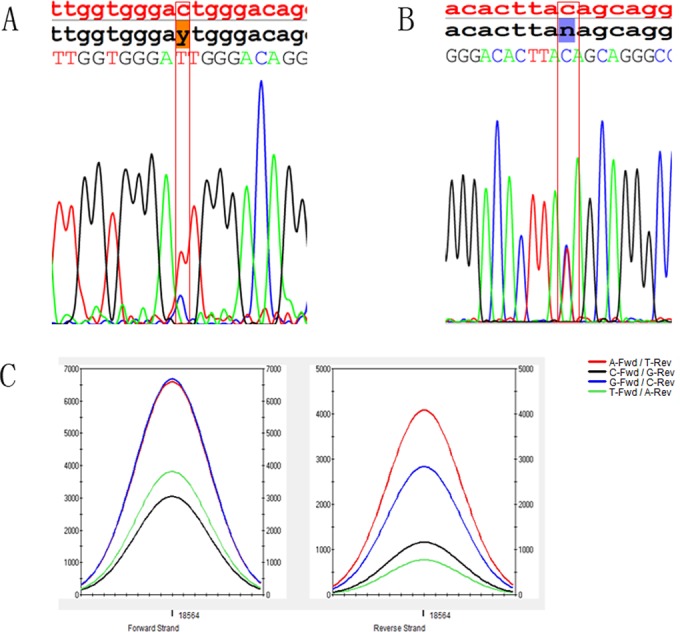
Comparison of nucleotide calls made by the resequencing chip and by standard capillary sequencing. The top panel depicts the reference nucleotide sequence in red, the chip readout in black, and nucleotides identified by capillary sequencing. (A) The c.104–1G>A heterozygous mutation identified by the chip (shaded in orange) was reproduced by capillary sequencing in patient 1^#^. (B and C) The intensity data for the “no call” base position can also be displayed as traces. The missense mutation G>A was successfully detected (C) and verified by direct sequencing (B) in patient 5^#^.

**Fig 5:**
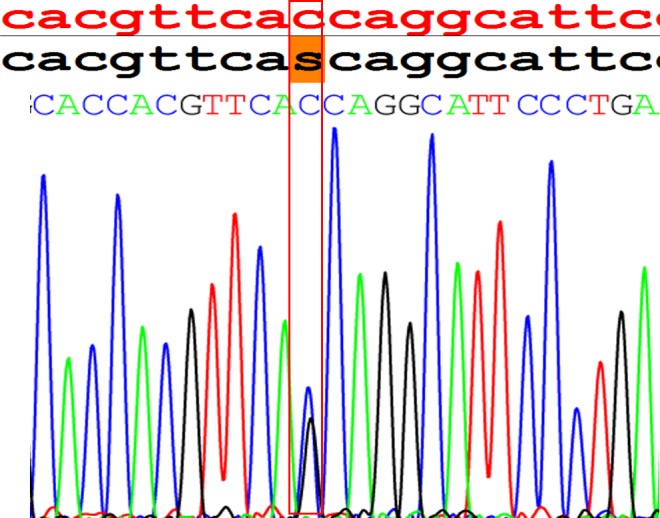
A mutation c.2191 G>C identified by OI array in patient 2^#^. This mutation was novel, and it led to p.Gly731Ala.

**Fig 6:**
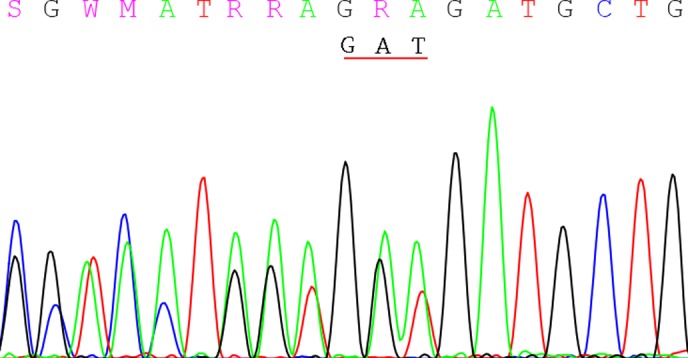
The insertion GAT verified by direct sequencing in patient 8^#^. It is a reverse sequencing, and the frame shifted to move right with the duplication GAT.

**Fig 7:**
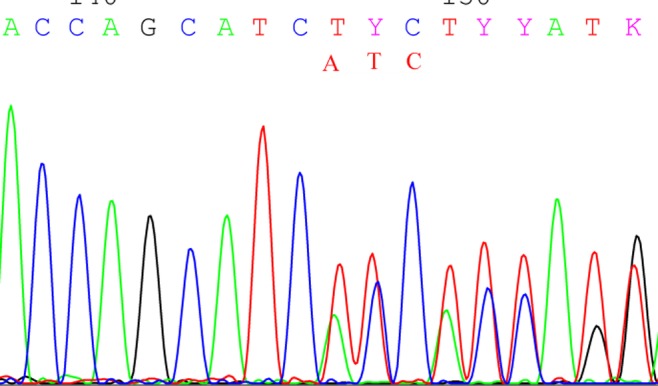
It is a reverse sequencing for the insertion GAT by direct sequencing.

In order to investigate the ability of the chip to potentially detect novel disease-causing mutations, we sequenced from the 13 OI patients using chip array. In this experiment, 11 pathogenic mutations were successfully detected in 11 patients. Interestingly, 6 out of the 11 mutations were located at *COL1A1*, including c.104–1 G>A (Fig. 4A), c.2191 G>C, c.2569 G>T, c.1155+1 G>A, c.967 G>A, and c.3433 G>A. The other 5 mutations were located at *COL1A2*, including c.1135 G>A, c.937–3 C>T, c.2332 G>A, c.1997 T>C, and c.4048 A>C. The results were further confirmed using standard capillary sequencing. Out of these pathogenic mutations, 10 have been reported earlier in Human Gene Mutation Database, while one mutation of patient 2^#^ (c.2191 G>C) was found to be novel (Fig. 5). Apart from the identified mutations, the position of one mutation showed “no call” in patient 5^#^, which was not identified automatically by OI array. However, conventional capillary sequencing analysis confirmed that this position was mutated at *COL1A1*—c.1155+1 G>A (Fig. 4B), and was also identified through manual analysis (Fig. 4C). In patient 8^#^, although no mutations were detected using chip analysis, a 3-bp insertion was identified by standard capillary sequencing (Figs. 6 and 7). In addition, the mutations in patient 1^#^, 5^#^, 6^#^, and 7^#^ were found in the introns, and were defined as splice site mutations (Table 2).

**Table 2:** Mutations identified in OI patients.

Sample	Gene	Exon/Intron	Mutation (cDNA)	Mutation (Protein)	Mutation Type	Zygosity of mutation
**1**	*COL1A1*	Intron 1	c.104–1 G>A	NA	Splicing	Heterozygous
**2**	*COL1A1*	Exon 32	c.2191 G>C*	p.Gly731Ala	Missense	Heterozygous
**3**	*COL1A1*	Exon 38	c.2569 G>T	p.Gly857Cys	Missense	Heterozygous
**4**	*COL1A2*	Exon 24	c.1135 G>A	p.Gly379Arg	Missense	Heterozygous
**5**	*COL1A1*	Intron 17	c.1155+1 G>A	NA	Splicing	Heterozygous
**6**	*COL1A1*	Intron 17	c.1155+1 G>A	NA	Splicing	Heterozygous
**7**	*COL1A2*	Intron 19	c.937–3 C>T	NA	Splicing	Heterozygous
**8**	*COL1A2*	Exon 23	c.1310_1312dupGAT	p.Asp437dup	Insertion	
**9**	*COL1A1*	Exon 15	c.967 G>A	p.Gly323Arg	Missense	Heterozygous
**10**	*COL1A2*	Exon 38	c.2332 G>A	p.Gly778Ser	Missense	Heterozygous
**11**	*COL1A2*	Exon 40	c.2467 G>A	p. Gly823Ser	Missense	Heterozygous
**12**	*COL1A1*	Exon 48	c.3433 G>T	p.Gly1145Cys	Missense	Heterozygous
**13**	*COL1A2*	Exon 52	c.4048 G>A	p.Gly1350Ser	Missense	Heterozygous

* new mutation found in the present study

Through OI array analysis, disease mutations were correctly detected in 11 out of 13 OI patients by the chip. After adjustment using conventional analysis, one “no call” result was compensated and the accuracy reached up to 100%, indicating that OI array could identify pathogenic point mutations with great accuracy. However, the ability of chip assay to identify mutations, specifically insertions, deletions, or duplications may be reduced; or in some cases, may be inhibited.

## Discussion

OI is a rare heritable bone disease. Genetic mutations that cause OI are often autosomal dominant. *COL1A1* or *COL1A2*, which encode type I collagen, are the most common mutational hotspots for OI. According to previous studies, 80–90% OI mutations were found to be located on these two genes[3,20–23]. Over the past years, most researchers have used the conventional sequencing method, such as capillary sequencing, to detect the mutations causing OI. Although this method proved accurate, its laborious and time-consuming features were the main shortcomings, which motivated the development of next generation sequencing technique and led to an improvement in the efficiency of diagnosis. In addition, a few potential mutations could not be detected due to the limited efficacy of conventional sequencing. The present study aimed at overcoming this issue by using array-based sequencing to identify mutations in 13 OI patients. The data revealed that the mutations present in *COL1A1* or *COL1A2* genes could be detected in all the 13 OI patients, whereas the mutations on other potential OI hotspots, viz. *CRATP*, *LEPRE1*, and *FKBP10* genes were not identified, as previously described.

There are several benefits associated with using OI array for genetic mutation screening, as compared to conventional capillary sequencing; and these include: (1) high-throughput technology: OI array has the potential to sequence up to 300 kb bases at the same time. In this study, we selected 29,906 bases, including all exons, 12 flanking base pairs of splicing junctions, and 280–500 bases upstream from the first exon for each gene; (2) highly effective: the analysis with GSEQ revealed a call rate of more than 96%, implying that the fraction of individual bases can be efficiently and specifically identified. Furthermore, OI array is capable of detecting a large number of bases with great accuracy, which has been confirmed by capillary sequencing (up to 99.9%); (3) high accuracy: the candidate point mutations could be correctly defined by the OI array. Our results showed that 12 of the 13 pathogenic positions were point mutations and one was insertion. Out of the 12 detected point mutations, 11 could be clearly verified automatically, while one mutation position, detected by manual analysis, showed “no call”, which was confirmed by conventional sequencing method. It has been seen that despite the potential problem of “no call”, the use of both sense and antisense sequencing probes alternatively overcomes the issues, while more than 90% of the “no calls” can be resolved by visual inspection of probe intensity[24]. In the other words, by taking advantage of automatic and manual analyses of OI array, all point mutations were successfully detected in all the 13 OI patients; (4) potential to define novel disease-causing mutations: OI array is able to discover rare variants potentially involved in disease susceptibility. Identification of OI associated genes becomes very difficult by using conventional sequencing approaches. Nevertheless, the resequencing of relevant genes is expected to be compatible with conventional sequencing, while allowing identification of rare mutations that contribute to disease development[25]. Interestingly, in this study, we found a novel mutation c.2191 G>C on Exon 32 in patient 2^#^ by using OI array; (5) Time-saving and cost-saving. In our study, because of the quantity of exons in five candidate genes, if we used capillary sequencing, it cost more than 2 months to sequence one sample from DNA extraction to results analysis. However, we spent only about 4 days to completing a sample by this technology. So OI array could save more time comparing with capillary sequencing. In addition, for five candidate genes, it could cost more than 7000 RMB to sequence one sample using capillary sequencing. It cost only 2000 RMB for one sample by OI array, which the cost reduced about three quarters comparing to capillary sequencing.

In previous studies, a similar approach would have probably not led to an accurate identification of insertions or deletions, unless they were specifically incorporated into the array design by the way of alternate probes[24,26–28]. However, in this study, a 3-bp insertion was detected successfully in patient 8^#^ by capillary sequencing, and not by OI array. This could be attributed to the presence of insertions or deletions that are inferred from disruptions in normal patterns of hybridization; and in case of OI array, the abnormal alleles may not have hybridized to the probes. This indicated that the ability to detect insertions/deletions may be a limitation of this new technology, as described previously.

In recent years, the GeneChip resequencing array technology was employed to explore the disease susceptible genes in cancer and mitochondrial diseases[6–8]. To our best knowledge, this is the first report that uses OI array for investigating the genes potentially relevant to OI in China. Although there are certain issues still to be addressed in the present study, the above-mentioned features of OI array will greatly facilitate the efficient identification of disease susceptible genes for the purpose of defining potential diagnostic markers for OI[16,29]. In the future, this method is likely to become an important clinical tool for the genotyping and molecular characterization of hereditary diseases.

## Supporting Information

S1 Datasets A list of Primers. 22 primer sequences are designed by us. Including all mutations we choose.
(XLS)

S2 Datasets OI-sequences. Including all the reference sequences of *COL1A1*, *COL1A2*, *CRTAP*, *LEPRE1* and *FKBP10*.
(ZIP)

S3 Datasets The probes of mutations. According to the selected mutations, we have made them into the probes to tiles on the chip.
(ZIP)
