# Correction: Development of a High-Throughput Resequencing Array for the Detection of Pathogenic Mutations in Osteogenesis Imperfecta

**DOI:** 10.1371/journal.pone.0127254

**Published:** 2015-05-01

**Authors:** Yao Wang, Yazhou Cui, Xiaoyan Zhou, Jinxiang Han

There are a number of errors in the published article.

Reference sequences for genes with identified variants were inadvertently omitted from the published pdf. The GenBank reference sequence records are:

*COL1A1* gene: NG_007400.1, *COL1A1* mRNA: NM_000088.3

*COL1A2* gene: NG_007405.1, *COL1A2* mRNA: NM_000089.3

The variant in patient 2, reported as c.2191G>C in Table 2 should be c.2192G>C, which yields the stated amino acid change p.Gly731Ala. Please see the correct Table 2 here.

**Table 2:** Mutations identified in OI patients.

Sample	Gene	Exon/Intron	Mutation (cDNA)	Mutation (Protein)	Mutation Type	Zygosity of mutation
**1**	*COL1A1*	Intron 1	c.104-1 G>A	NA	Splicing	Heterozygous
**2**	*COL1A1*	Exon 32	c.2192 G>C*	p.Gly731Ala	Missense	Heterozygous
**3**	*COL1A1*	Exon 38	c.2569 G>T	p.Gly857Cys	Missense	Heterozygous
**4**	*COL1A2*	Exon 24	c.1135 G>A	p.Gly379Arg	Missense	Heterozygous
**5**	*COL1A1*	Intron 17	c.1155+1 G>A	NA	Splicing	Heterozygous
**6**	*COL1A1*	Intron 17	c.1155+1 G>A	NA	Splicing	Heterozygous
**7**	*COL1A2*	Intron 19	c.937-3 C>T	NA	Splicing	Heterozygous
**8**	*COL1A2*	Exon 23	c.1310_1312dupGAT	p.Asp437dup	Insertion	
**9**	*COL1A1*	Exon 15	c.967 G>A	p.Gly323Arg	Missense	Heterozygous
**10**	*COL1A2*	Exon 38	c.2332 G>A	p.Gly778Ser	Missense	Heterozygous
**11**	*COL1A2*	Exon 40	c.2467 G>A	p. Gly823Ser	Missense	Heterozygous
**12**	*COL1A1*	Exon 48	c.3433 G>T	p.Gly1145Cys	Missense	Heterozygous
**13**	*COL1A2*	Exon 52	c.4048 G>A	p.Gly1350Ser	Missense	Heterozygous

* new mutation found in the present study

The variant in patient 4, c.1135G>A, p.Gly379Arg in *COL1A2*, reported to be in exon 24 actually lies in exon 21.

The variant in patient 7, c.937-3C>T in *COL1A2*, reported to be in intron 19 actually lies in intron 18.

Sources for the sequence files in S3 Supplementary data information can be found online in the Ensembl database: http://www.ensembl.org/index.html as well as the Osteogenesis Imperfecta Variant Database: https://oi.gene.le.ac.uk/home.php
