# Supplementary material for: Development of a High-Throughput Resequencing Array for the Detection of Pathogenic Mutations in Osteogenesis Imperfecta
Source: PLoS One. 2015 Mar 5;10(3):e0119553. doi: 10.1371/journal.pone.0119553 (PMC4350936; doi:10.1371/journal.pone.0119553)
Supplement: S2 Datasets — Including all the reference sequences of COL1A1, COL1A2, CRTAP, LEPRE1 and FKBP10. (ZIP) [file pone.0119553.s002.zip › OI-FKBP10_sequence.doc]

| 1 cccgagcctc tctccctggc caggccccag gtctcgcagc cagggatgga gatgggggga | | | | | | |  |
| --- | --- | --- | --- | --- | --- | --- | --- |
| 61 gggggaacct agagttcttt gtagtgcctc cctcagactc taacacactc agcctggccc | | | | | | |  |
| 121 cctcctccta ttgcaacccc ctcccccgct cctcccggcc aggccagctc agtcttccca | | | | | | |  |
| 181 gcccccattc cacgtggacc agccagggcg ggggtaggga aagaggacag gaagaggggg | | | | | | |  |
| 241 agccagttct gggaggcggg gggaaggagg ttggtggcga ctccctcgct cgccctcact | | | | | | |  |
| 301 gccggcggtc ccaactccag gcaccatgtt ccccgcgggc ccccccagcc acagcctcct | | | | | | |  |
| 361 ccggctcccc ctgctgcagt tgctgctact ggtggtgcag gccgtgggga gggggctggg | | | | | | |  |
| 421 ccgcgccagc ccggccgggg gccccctgga agatgtggtc atcgagaggt accacatccc | | | | | | |  |
| 481 cagggcctgt ccccgggaag tgcagatggg ggattttgtg cgctaccact acaacggcac | | | | | | |  |
| 541 ttttgaagat ggcaagaagt ttgattcaag gtaaccccgg ttgggcgccc ccggattcac | | | | | | |  |
| 601 cactccgtcc cctgaacccg gggtcctgtt tccttgttgc ctcttttaag ctctgcatcc | | | | | | |  |
| 661 caatactcta accctagaca gcacccctgg ggcctcccag acacccatct ccccaaagat | | | | | | |  |
| 721 accctcctgc ccccacttcc aaaccctcac ttctcaaagg atcccatttc tttctcacaa | | | | | | |  |
| 781 gctctccaca tcccggaaag gactcgataa tcaaggtgaa ctgccttgtg tagttaccag | | | | | | |  |
| 841 ggctgtatgt aatgacctga tggagagggg tgttctgatc tacagtgttg gggggaaggg | | | | | | |  |
| 901 ggtggtccct gctgcttaag aaccctggga tggtctatca gcctggaccc cgtacctttc | | | | | | |  |
| 961 cacccaccct gactcccctt ctggttccac tgttttcagt gcctttgtgg tcagagaaga | | | | | | |  |
| 1021 gggaatagaa cttttttttg gagggggtgg gagttttcag cggctggtgg gcagggaaaa | | | | | | |  |
| 1081 aggctggtgg gtattataag aagacagccg gctggacctg ggggtagcgc gtggctccct | | | | | | |  |
| 1141 gcggaggccc tgtctacgtg tcacacagtc ggacatgcca ccctgggctc cttggaggaa | | | | | | |  |
| 1201 tttcattcct ccctctctgg ttctcctgga accctcatct ctctccattt taggtacacc | | | | | | |  |
| 1261 aggccagagg cacaggccgc ccatttttgc cagccttggg gtttgtaagg tggcctcctg | | | | | | |  |
| 1321 gtctgaccca ggcctttggt ctgccccagc aagtccactt ttgttagcgg ggacagggca | | | | | | |  |
| 1381 gggctgtatg ccagcctcag gggtaaatct agggtcccag ttacacacag accccacagc | | | | | | |  |
| 1441 acactgaaag aagtcagtgt ttcttagacc ctcctctgaa acacacaact cagcccacag | | | | | | |  |
| 1501 gaaattagtg gccatgactt tagaggcagg catatattgg ggtaagccta caaactccaa | | | | | | |  |
| 1561 gagtcccaag accctggttc aaatccaggc tatgaccatg gacagagaat tttgtctctt | | | | | | |  |
| 1621 taggcatcac tttccttatc tgaaaagtgg agacaaaaac aggacctact tgctaaggct | | | | | | |  |
| 1681 gatgttagga ctccaaggac atggatgaat gaacgtaggc ttctcacagg gcctggcagt | | | | | | |  |
| 1741 cctccagcca gttcagcaca cattaacatt gaccctgagg aaaaccatat gacctgatgc | | | | | | |  |
| 1801 tgtggaactt gtagaaactt aatgcagagc ttaagccttc ataacaacac tgagaaggaa | | | | | | |  |
| 1861 acagtccaga cttaaaacct gtctgcttgg tgggcaaatt cagatatagg gtagtagcca | | | | | | |  |
| 1921 gaagtgcagc cctgagccct tgactgctag gagtgaggca ggggttggga tttaggggat | | | | | | |  |
| 1981 gtcatccctg atggggactc tgggagccca gctcatgagt gttgagcttg gaaaactttt | | | | | | |  |
| 2041 tgttttttag ctggaggctc actctgtcgc ccaggctgga gtgcagtggt gccatctcag | | | | | | |  |
| 2101 ctcactgcaa cctcctgggt tcaagcaatt ctcctgcttc agcctcccaa gtagctgtga | | | | | | |  |
| 2161 ttacaggcgc atgccaccac gcccagctaa tttttgtttg tttgtttgtt tttgtttttt | | | | | | |  |
| 2221 gagacacagt ctcacactgt tgcctgggct ggagtgcaat ggagcaatct caactcactg | | | | | | |  |
| 2281 caacctccgc ctctgcctcc caggttcacg tgattctcct gcctcagcct cccgagtagc | | | | | | |  |
| 2341 tgggattaca ggtgcacacc accacacccg gctaattttt ttgtattttt agtagagagg | | | | | | |  |
| 2401 ggtttcacta tgttggccag actggtcttg aaaaaggcgg gtgggtatta taagaagaca | | | | | | |  |
| 2461 gccagctgga cctgggggta gggcatggct ccctgcggag gccctgtctg tctacgtgtc | | | | | | |  |
| 2521 acacagtcag acatgccacc ccagcctcgt gatccgccca cctcggcctc ccaaagtgct | | | | | | |  |
| 2581 gggattacag gcgtaagcca cggcgcccag ccttaatttt tcatatttta gtagagatgg | | | | | | |  |
| 2641 cgtttcacca tgttgctcag gctggtcaca aacttctgag ctcaggcaat ccaccctcct | | | | | | |  |
| 2701 cagcctccca aagtgctaga attacaggcg tgagcccccg cgcccacaca acttagaaaa | | | | | | |  |
| 2761 cttttttttt ttttgagaca gagtcttgct ctgtccccca ggctggagtg cagtagcgcg | | | | | | |  |
| 2821 atctctgctc actgcaagct ccgcctcctg ggttcacacc attctcctgc ctcagcctcc | | | | | | |  |
| 2881 cgattagctg ggactacagg cgcctccaca attttttttg tatttttagt agagacgggg | | | | | | |  |
| 2941 tttcaccatg ttagccaggg tggtctccat ctcctgacct catgatctgc ccgcctcggc | | | | | | |  |
| 3001 ctcccaaagt gctgggatta caggcgtgag ccactgcgct cggccagaaa actttcttaa | | | | | | |  |
| 3061 cccatggccc cttcaattaa aaaaaaaaaa atctcccatc ctcactttac ttctgaaaac | | | | | | |  |
| 3121 ttaaaataaa ttaaaagtgt atataattgg ctgggcacag tggctcactc ctgtaatccc | | | | | | |  |
| 3181 agcactttgg gaggcccagg caggcagatc acttgaggcc aagagtgacc agcctgggca | | | | | | |  |
| 3241 acatggcgaa agccctctct actaaaaata caaaaattag ccagatatgg tggtgcatgc | | | | | | |  |
| 3301 ctgtgatccc agctgctact tcggaggctg aggcaggaga atcgcttaaa cccgggaggt | | | | | | |  |
| 3361 ggaggttgca gtgagctgat tgcaccactg cactccagcc tgggtgacag agccgagact | | | | | | |  |
| 3421 ccatctccaa aaatacatat gtataagtaa aaataaatac attaattaaa agtgtataac | | | | | | |  |
| 3481 taaaaacaaa caaaagccat ggcactttta gaataggctt tttttttttt tttttttttt | | | | | | |  |
| 3541 gagagggagt ctcactctgt cgccaggctg gagtccagtg gcacgatttc ggctcattgc | | | | | | |  |
| 3601 aacctccgcc tcccgggttc acgccattct cctgcctcag cctcctgagt aactgggact | | | | | | |  |
| 3661 acaggcgccc gccaccacac ctggctaatg ttttgtattt ttagtaaaga cggggtttca | | | | | | |  |
| 3721 ctgtgttagc caggattgta gaataggcct tttcaaaatg tgaaaccagc tcccacccca | | | | | | |  |
| 3781 cctctgctct cccgtctttt cttggagagc caatagtata atggcaaatt ttagaactca | | | | | | |  |
| 3841 gctgactaca tattagctgt gtgaccttgg gcaaggcact gagtctctca agtctccatt | | | | | | |  |
| 3901 gctctcatta agaaaaatga gaacaagaat gcccacctca caggggagac aaagaagata | | | | | | |  |
| 3961 cggaatgtaa aatgccaagc atttaatccc atatagtaat gctcctctgc ccctccgtgc | | | | | | |  |
| 4021 tgtacgtacc actccccact tgctgtcagc aaatagtagc agccatcagg cccgggcatc | | | | | | |  |
| 4081 ccacccaacc ccgtcccagg gcaatagcag cccttgtcct cctcagggac ttgcctttca | | | | | | |  |
| 4141 caacagtccg ttctcttaag gtgggcgtca cggccgacct aggaggggga cttgaaaagg | | | | | | |  |
| 4201 aggtgagaag tgtgtgtgcg tatccacacc tggctatagg gaggggggat tgtgtgcgtg | | | | | | |  |
| 4261 tgtgcaggca cacctgtgca tctgtgccac catgggcagt gtgtgtgtgt ctgaggtcac | | | | | | |  |
| 4321 tgtatcccat ctgtccctcc ccccccagct atgatcgcaa caccttggtg gccatcgtgg | | | | | | |  |
| 4381 tgggtgtggg gcgcctcatc actggcatgg accgaggcct catgggcatg tgtgtcaacg | | | | | | |  |
| 4441 agcggcgacg cctcattgtg cctccccacc tgggctatgg gagcatcggc ctgggtgaga | | | | | | |  |
| 4501 agggctgggg cacaggccgg gggtggagga gaccacgagg cagaatcagg gatcctgggg | | | | | | |  |
| 4561 tgagaaaact gaagtgcgga gatgaggagt gacttgccca atgtcacact gtgcacgcag | | | | | | |  |
| 4621 tatgaagagt ggcagctctg gctggggcag tagctgacat atatggtccc agagttttgg | | | | | | |  |
| 4681 gaggccgagg tgggaggatt gcttgaaccc aggagttcca gaccagccta ggcaacatag | | | | | | |  |
| 4741 tgagacctcg tctctacaaa aaataaaata aattatccag gtgtggtggt gtgtgcctgt | | | | | | |  |
| 4801 agtcccaact actcagaagg ctcaggtggg aggattgctt gaccccggga ggctgcagtg | | | | | | |  |
| 4861 agccatgatc atgccactga attccaccct gtgtgacaga gcaaaacccc tctttttttc | | | | | | |  |
| 4921 tttttctttt tttttttgag acagcatctc actctgttgc acaggctgga gtttagtggt | | | | | | |  |
| 4981 gcgatctcgg ctcactgcaa cctcagcctc ctgggctcaa gcagtcctcc ctctcagcct | | | | | | |  |
| 5041 cccaaatagc tgggactaca ggtgcacacc accacacgtg gctaattttt tgtattttta | | | | | | |  |
| 5101 gtagagatgg ggtttcgcca tgttgcgcag gctggtcttg aactcctggg ctcaagccat | | | | | | |  |
| 5161 ccacctgcct tggcctccca cagtgctggg ataacaggca tgagccactg tgtctggccc | | | | | | |  |
| 5221 cctatctctt aaaaaaaaaa aacaaaaata gtggcatctc tgtccctggt ttgggtctct | | | | | | |  |
| 5281 tggtgctggg atgagaggaa ggggaataac agggccctgg ctgggatcgt ggttggcaga | | | | | | |  |
| 5341 gcagaactct gagacctcca cgctgctgcg ctctcacagc ggggctcatt ccaccggatg | | | | | | |  |
| 5401 ccaccctcta cttcgatgtg gttctgctgg atgtgtggaa caaggaagac accgtgcagg | | | | | | |  |
| 5461 tgagcacatt gctgcgcccg ccccactgcc cccgcatggt ccaggacggc gactttgtcc | | | | | | |  |
| 5521 gctaccacta caatggcacc ctgctggacg gcacctcctt cgacaccagg tgaggggctg | | | | | | |  |
| 5581 gaggggagcc ctgaggcact ggggactgtg gcatggggag cgggaatccg gggcccagcc | | | | | | |  |
| 5641 tgcctctccc acctccacct cattttctgc agctacagta agggcggcac ttatgacacc | | | | | | |  |
| 5701 tacgtcggct ctggttggct gatcaagggc atggaccagg ggctgctggg catgtgtcct | | | | | | |  |
| 5761 ggagagagaa ggaagattat catccctcca ttcctggcct atggcgagaa aggctatggt | | | | | | |  |
| 5821 gagggtgggc aaggacacaa ggggaaattc cgcagaagag ggaaaaacca ggcctccaca | | | | | | |  |
| 5881 tacagttgct caggttgtat actgcacgag ggcatccaac caaggactca aggtgggatg | | | | | | |  |
| 5941 aaatctaccc ttggtgctac taagaagggg tgctttggcc gggcgtggtg gctcacgctt | | | | | | |  |
| 6001 gtaatcccag cactttggga agccaaggcg ggaggatcac gaggtcagga gatcgagacc | | | | | | |  |
| 6061 acggtgaaac cccgtctcta ctaaaaatac aaaaaaatta gccgggcgtg gtgggggcgc | | | | | | |  |
| 6121 ctgtagtccc agctactcgg agaggctgag gcaggaaaat gacgtgaacc cgggaggcgg | | | | | | |  |
| 6181 agcttgcagt gagccgagat cgcgccactg cactccagcc tgggtgacag agcgagactc | | | | | | |  |
| 6241 tgtctcaaaa aaaaaaaaaa aaaaagaagg ggtgctttat tctgattcac acgaaggctc | | | | | | |  |
| 6301 agtatgagcc ggtggtggcc ctggggaaaa cccagctcag gtcttactgg aggagcaaga | | | | | | |  |
| 6361 agcagggctg ctgatgggcg ggaaagggct ctggagagtg gggctagtgt cttgcatggt | | | | | | |  |
| 6421 gcccactggg ccttcctgag tcaagaagga gcctcggctt gctccccaat tttatggttc | | | | | | |  |
| 6481 aagccctatc ccttccccag ggacagtgat ccccccacag gcctcgctgg tctttcacgt | | | | | | |  |
| 6541 cctcctgatt gacgtgcaca acccgaagga cgctgtccag ctagagacgc tggagctccc | | | | | | |  |
| 6601 ccccggctgt gtccgcagag ccggggccgg ggacttcatg cgctaccact acaatggctc | | | | | | |  |
| 6661 cttgatggac ggcaccctct tcgattccag gtcaggaggg tcttgaggtg ggagggcggg | | | | | | |  |
| 6721 ggctgggtga aacgtggacg aagctggggg tcactctgag ctgcctggaa ggggagggcc | | | | | | |  |
| 6781 cctttgactc ccttcctggc cctcccgcct tgtattgcag ctactcccgc aaccacacct | | | | | | |  |
| 6841 acaataccta tatcgggcag ggttacatca tccccgggat ggaccagggg ctgcagggtg | | | | | | |  |
| 6901 cctgcatggg ggaacgccgg agaattacca tccccccgca cctcgcctat ggggagaatg | | | | | | |  |
| 6961 gaactggtag gggcgttccc cagccaccac ctcagctcct cctccgaact gcccattgtg | | | | | | |  |
| 7021 tctaggccac cccctcccac agtgggattc caggcaccgc tcggcccctc tcatcacaaa | | | | | | |  |
| 7081 aacatgcatg cagcttacat ctggtcaccc catctgattc ctgccacaca gactccatcg | | | | | | |  |
| 7141 gtttcctcca gggcagcgcc ccacttcgcc ccttccgcag tggagaaggg cagccaagtt | | | | | | |  |
| 7201 tggggaggga gggtggttat ggaaaaacag aaccagcata cccccaggac ccagctgtgc | | | | | | |  |
| 7261 tgggagcctc agtgtcctca cctgtcaagt gggcaagcca tgctgatccg cagggtaagt | | | | | | |  |
| 7321 tactgggagt ttgcaagacg gtgagtggaa aagggctttc ttactggaaa gctgccttcc | | | | | | |  |
| 7381 cctgccccct gccccagtga aagtttgtta gaattgactc tggacaaaca tcccgtccca | | | | | | |  |
| 7441 tcctcctttg gatcctcagg gtcgggaagg ggtatcaggt ctggggacct ccatggagag | | | | | | |  |
| 7501 acctcaagta gcctctccta gtgctctgag ctgaccacac tcccccattc tggcctcagg | | | | | | |  |
| 7561 agacaagatc cctggctctg ccgtgctaat cttcaacgtc catgtcattg acttccacaa | | | | | | |  |
| 7621 ccctgcggat gtggtggaaa tcaggacact gtcccggcca tctgagacct gcaatgagac | | | | | | |  |
| 7681 caccaagctt ggggactttg ttcgatacca ttacaactgt tctttgctgg acggcaccca | | | | | | |  |
| 7741 gctgttcacc tcgtgggtcc gggggggggc cgggactggg caggtgggtg ggcacaggca | | | | | | |  |
| 7801 tggggagtcc tcctcagtgc acccccgacg cctgctcctc cctcttggtc ctcgagcgcc | | | | | | |  |
| 7861 aggggagcat tcaacctctt gctgctttct gtaagtcccc atctcggagc atgtcgagga | | | | | | |  |
| 7921 gatgaaattc tctgcttcgc aggggaaggg aaggtgaagc caacagttgg gggagaactg | | | | | | |  |
| 7981 ctctttctat ttcacagagg ggaaactgac gcagggagcc atgagccctc gaggccacac | | | | | | |  |
| 8041 tttagggggc agaggcaaga tgagaaggga accccacgtc tgcacagctg ggcccctgca | | | | | | |  |
| 8101 ctctgctgcg tggcccaagt caccagtggg agtaactccg gaactgaggg cttgttctgg | | | | | | |  |
| 8161 gcccacctca gagggagagg ggtgtgcgct ggcaggggac agggtggctg ctgacctggg | | | | | | |  |
| 8221 catctgctct cccccaggca tgactacggg gccccccagg aggcgactct cggggccaac | | | | | | |  |
| 8281 aaggtgatcg aaggcctgga cacgggcctg cagggcatgt gtgtgggaga gaggcggcag | | | | | | |  |
| 8341 ctcatcgtgc ccccgcacct ggcccacggg gagagtggag gtgaggggct gagaccataa | | | | | | |  |
| 8401 tctttttttt tttttttttt tttttttttt tgagatggag tctgactctg tcacccaggc | | | | | | |  |
| 8461 tggagtgcag tggtgtgctc tcagctcact gcaacctcca cttcccaggt tcaagtgatt | | | | | | |  |
| 8521 ctcctgcctc agcctcccga gtagctggga ttacaggcac ctgccaccgt gcccagctaa | | | | | | |  |
| 8581 tttttgtatt tttagtagag acagggtttt gccatgttgg tcaggctggt cttgaactcc | | | | | | |  |
| 8641 tgacctcagg tgatccaccc gccttggcct cccaaagtgc tgggattata atcatgagcc | | | | | | |  |
| 8701 actgcgctcg gcccgagacc gtaatctgac tggcatctgt cccttttgct cctgcccact | | | | | | |  |
| 8761 gtgggtctga tgactggtgg gaggagtcag gaatgccttc aggatggctc cttaaacatc | | | | | | |  |
| 8821 ccatgcccca ctctccagcc cagccccagg aggggaaact ggcctgtggg ctgggaaaca | | | | | | |  |
| 8881 gtgaagccag gcccagaccc cggcctgact aggacccctc ccttctctcc tgccctccct | | | | | | |  |
| 8941 ccagcccggg gagtcccagg cagtgctgtg ctgctgtttg aggtggagct ggtgtcccgg | | | | | | |  |
| 9001 gaggatgggc tgcccacagg ctacctgttt gtgtggcaca aggaccctcc tgccaacctg | | | | | | |  |
| 9061 tttgaagaca tggacctcaa caaggatggc gaggtccctc cggaggaggt gggtgaaggt | | | | | | |  |
| 9121 tcagtcctaa tagccatgcc cacgcaatcc ccgcacccag gaagcatcga ggaagaagac | | | | | | |  |
| 9181 gtcccccgtc cggactgccc acccgccctg gtgctcctgc ctgcgctgag tcccacgcct | | | | | | |  |
| 9241 caggctcctt gtccctgctt tttcctgggc acacatgcag gctgttccct acctgagacc | | | | | | |  |
| 9301 agtcacagac tatccccatg ccacgcctcc accccagccc ccaccaggac cccagcacca | | | | | | |  |
| 9361 gtgcctttcc cagcccttcc tgagttacag ggtgcggggg agcctgggaa aaaagaagaa | | | | | | |  |
| 9421 aaagaaagca ctcactggcc tccacccggg gcccctgccc ctcccaaggc catgaccctc | | | | | | |  |
| 9481 actgcccgct cccccggctc tcccctgccc cagttctcca ccttcatcaa ggctcaagtg | | | | | | |  |
| 9541 agtgagggca aaggacgcct catgcctggg caggaccctg agaaaaccat aggagacatg | | | | | | |  |
| 9601 ttccagaacc aggaccgcaa ccaggacggc aagatcacag tcgacgagct caagctgaag | | | | | | |  |
| 9661 tcagatgagg acgaggagcg ggtccacgag gagctctgag gggcagggag cctggccagg | | | | | | |  |
| 9721 cctgagacac agaggcccac tgcgaggggg acagtggcgg tgggactgac ctgctgacag | | | | | | |  |
| 9781 tcaccctccc tctgctggga tgaggtccag gagccaacta aaacaatggc agaggagaca | | | | | | |  |
| 9841 tctctggtgt tcccaccacc ctagatgaaa atccacagca cagacctcta ccgtgtttct | | | | | | |  |
| 9901 cttccatccc taaaccactt ccttaaaatg tttggatttg caaagccaat ttggggcctg | | | | | | |  |
| 9961 tggagcctgg ggttggatag ggccatggct ggtcccccac catacctccc ctccacatca | | | | | | |  |
| 10021 ctgacacagc tgagcttgtt atccatctcc ccaaactttc tctttctttg tacttcttgt | | | | | | |  |
| 10081 catccccact cccagcccct tttcctctat gtgacagctc cctaggaccc ctctgccttc | | | | | | |  |
| 10141 ctccccaatc ctgactggct cctagggaag gggaaggctc ctggagggca gccctacctc | | | | | | |  |
| 10201 tcccatgccc tttgccctcc tccctcgcct ccagtggagg ctgagctgac cctgggctgc | | | | | | |  |
| 10261 tggaggccag actgggctgt agttagcttt tcatccctaa agaaggctcc tttccctaag | | | | | | |  |
| 10321 gaaccataga agagaggaag aaaacaaagg gcatgtgtga gggaagctgc ttgggtgggt | | | | | | |  |
| 10381 gttagggcta tgaaatcttg gatttggggc tgaggggtgg gagggagggc agagctctgc | | | | | | |  |
| 10441 acactcaaag gctaaactgg tgtcagtcct tttttccttt gttccaaata aaagattaaa | | | | | | |  |
| 10501 ccaatggc |  |  |  |  |  |  | |
