# Supplementary material for: Development of a High-Throughput Resequencing Array for the Detection of Pathogenic Mutations in Osteogenesis Imperfecta
Source: PLoS One. 2015 Mar 5;10(3):e0119553. doi: 10.1371/journal.pone.0119553 (PMC4350936; doi:10.1371/journal.pone.0119553)
Supplement: S2 Datasets — Including all the reference sequences of COL1A1, COL1A2, CRTAP, LEPRE1 and FKBP10. (ZIP) [file pone.0119553.s002.zip › OI-LEPRE1_sequence.doc]

| 1 gacagaatgg gcaaatttcc attgtctgaa caaaccaagt aaaaaaaacc atgaatcatt | |
| --- | --- |
| 61 tattctttgg ttgtctacac agacacttaa gtactgtatc gctgtcatgc agcggcctgt | |
| 121 ggaggcccct gggggtggct gggcctgtgt cctgagccct cagccagatc cagggggtgc | |
| 181 ggtgtctggt catgtccact ccaagagcag tagcaccatg tagaaggctg tgagcagggt | |
| 241 cccctcggct gagtggcaga tgtaggctca ctgctctgca gccccgaggg gctggccagc | |
| 301 tcagagtgca gaagagttcc tctccatggg tctagtcacc catccgtctg acctggacgc | |
| 361 tgtcatagct catccttggg cttcgattca ctgcctgaga gagactcttg tgcaggttcg | |
| 421 gggggaccct gctgggcatc caggggctgc tcctgggaga ggtccatctc ttctgggctg | |
| 481 aagagcatct tcaccaggtc atctgcctgc accctgtcct gcaaggacaa acccctgccc | |
| 541 attcagagag gcctccgctc cagacactcg ctatctctca gctgctactt cccaggggac | |
| 601 aaggaaggtt ccttaatggc cccaggctca tctccttccc agcaggtcct actctctgga | |
| 661 aaaggacagc tgaagtgggg cccaaggagg gaaccaagaa ctcagcatcg ctccctgtct | |
| 721 tgacctcttt ccccacctgg atcccgttca cagcaggccc agggaggaca aggcaatgtg | |
| 781 accataatga cagggcgttg gagctggtgt cccaagtgct cctttcgtgt ctcagccact | |
| 841 ggcaatggga tttggggaag agaaaggcaa accaagggcg ctctgggaac gggtcaccac | |
| 901 agcaccagct gctctcaccc gctcgagctg ctctcacccg ctcgctgtgt cgagggtcca | |
| 961 gggtgaacca cagggcgatg gcacagcgct gccccctggt gacagccttc actccatgtg | |
| 1021 ggttttcagt gcctgaagag aatcccacgg ctcttccaca ctgaggctgc acctctgcct | |
| 1081 aagggggaca gaaagggagg ggggttgcac aggacaaaga ctgtaatcca ctctcagaag | |
| 1141 agacatgggc tcattagggc tcacgaccga gggcagctct tcagtatggc ttccctaaga | |
| 1201 ccagaactaa agagaaaggg tgactggtta gaggagtgat ggacacgtct caaagcccct | |
| 1261 ctggatgggg gaagggtacc gcccactggg aagcccagtt ggttattttt gtccactgca | |
| 1321 ctttgggaac atcagctgta gaaaacagac tttttatctc ccagggacaa ggacaaggga | |
| 1381 gcactcaccg tcacggtctt ggcatccagt tcagtgaaat aaaagtttcc gccatcgaag | |
| 1441 tccccattta ggtaaaggat ggcgctggga aaggcagaga catctcatca tggcccttcc | |
| 1501 ctgcctccct ttcccctgag ccttcacctc caggtgctct caaccagcag caggagggtg | |
| 1561 gctttgtgtg ggagggaaac ctttaagaac ctatactgat gccgtacaaa ctccaccttt | |
| 1621 ccactctcca ccctcgcctt agagtcccct gtgcccactc acactaccgt tcttgtcaca | |
| 1681 ccacgatatg attccctctt tgcctatctt cttatccagc gggtttgggg ataaaggatt | |
| 1741 ggactctggg aacagctagc cccaaccagt gtgtgtgtgc tagggtgggg tagtagagtg | |
| 1801 cagggcactg tggggcctct gaggagggca cagacctcct caccctgcac ctgccccgca | |
| 1861 ctcacctgta gtcgcggaag gtgtaggctg ggggctcttt gacacacacg agggtctcgg | |
| 1921 cattcaggat gcagttgtcc acgtggactg gatgactatc atccttcctc tctgcctgga | |
| 1981 cctctggggc caatgtcaca catgttagca agggagcacc tcgggagacg gcatagctct | |
| 2041 cttcttggca ggggaggtgc ttcccaaaat gtgtttgagt ctacggaatg cccaatgaac | |
| 2101 taggggggtg tctttggcta agccacaagc ctaggggtca ggacaagtct gcaggcttcc | |
| 2161 taggacagtt cttggcagaa gtgagcaggg tgtggcaagc aggaatgaag ggctccaacc | |
| 2221 aagggcagat cctgggcaga atggcacaac cctggtttgt agctggactt tacaaaatac | |
| 2281 aaaggcccag ggaagtgaga atgagggatc aggaggtggg acggacgcag ggcagagtgg | |
| 2341 gtcagtcctc catcaggact gcaggagatg gggctgagtg gcaggaagtg gggcagagaa | |
| 2401 gagctggcta gcaaagggaa agcatgaact tcagtggggc tctggggtgg ggtgggtgga | |
| 2461 gaaaccatgc agggagaagg tcacgaaagg gaacagaccg ggctcactgt caaatgccag | |
| 2521 gcagaccacg tctttcccca acgcaaacct ttggcatgct ctatgtaggc accgtcacgt | |
| 2581 cgtggtgggt gcctccttct ccattcccac ttgtccagcc agtcaccagg tcctgtccat | |
| 2641 ccacctcctg caccttcccg gggcctcact tctccctcct cccactgcca cttcctgagt | |
| 2701 ttagggcttt gcccttgtcc acagaggtca ttgctgacca gcttcccttt ttggtttgaa | |
| 2761 atctctccac acacacagca gcaaaatgaa tcctttaaaa gtcaaggcct caccacgtcc | |
| 2821 ttcactgctt gatgaggcct ccctggcctc ctggcacctg tgcttgtgct ctcccaccgg | |
| 2881 gggcttgtga cacacccagt tgtgtgtctg actggtccct ttgggtgtgc ccagattgtg | |
| 2941 acagaattcc aaacattctg aggtagagga aagccctctg atctgtgcca cgtaccaatg | |
| 3001 gccatcactg ccaggggcct gctggaggct ccagtatgac agagccggct ctgggccaca | |
| 3061 taggatcctt gcagcactag ctgaacctct ccccttcttg gacatgcaaa agggaatgtg | |
| 3121 ggtgggcgag ggaatgagga ataacctcca ggccactctc acttgtaaat cacgaagctc | |
| 3181 tctcacaaat ttggtattta acaattagta aaagagaatc tgccacatag ctatggttga | |
| 3241 gctaaaggat aaaaattccc ttgaatgtag ctctccagga tttggcacct ttcttagttc | |
| 3301 ccctgactac caggctgcac aatgcctcca ctcctgtgca aacaccatcc caccacccgg | |
| 3361 agtactccgt aatcgtattc atcctgcaaa actgccattg cttcctctgg gaagccttct | |
| 3421 ttttgttgtt gttgttgttg ttattatttt tgcttttaac aagcccttct gttgagggct | |
| 3481 gtctttcaac agatggcagc gagggagctg ctcagctatg tatgaaaccc taacccggag | |
| 3541 gcaggtcctc tatgaatggt ttagcaccag cttccccatt aaagtgcgtt gtgtgacaag | |
| 3601 tgacgggtgg ccccctttct ggctgcaccc catttccctc tgggaagcct tcttgatccc | |
| 3661 caggtaggtt gactataact tcctccatgc tactgtggca tgcatcacac tgggatgcca | |
| 3721 cccatgtatt tacattggtt ccccaactga agaaaaggga ccgcatcctg ttctctctgt | |
| 3781 gtcttcagca tccagcacag agctgaggta cagcatgcgg gggcgggtgc tgcaggggta | |
| 3841 atgaggccca ccttcgatgg cagtgcggca caccagatga gagtaggaaa agtagagggg | |
| 3901 cgtatccagg cggaagtagg actccatgat gcgccgcacc ttctccgtca cgttgtagta | |
| 3961 caggtgggca ctctgcagag gaactttgcc ttcttgcccc agctgccaag gagacagatg | |
| 4021 gtcagctgcc ctcaacgact gatatggttt ggctctgtgt ccctacccaa atcttatctt | |
| 4081 gtagttccca taattcccat gtgttgtgga agggacctgg tgggagatga ctgaataatg | |
| 4141 ggggtggatc tttcccgtgc tgttctcata atactgaata agtctcacga gatctggtgg | |
| 4201 ctttaaaaaa atgggagttt gcgtccggga ggtgaggggc gcctctgccc ggccgcccct | |
| 4261 actgggaagt gaggagcccc tcagcccggc cagccacccc gtccgggagg gaggccgggg | |
| 4321 ggggtggtcg gccagccgcc ccgtccggga gggaggtggg ggggtcagcc ccccgcccgg | |
| 4381 ccggccgccc tgtccgggag gtgaggggcg cctctgcccg gccgccccta ctgggaagtg | |
| 4441 aggacccctc tgcccggcca gccgccccgt ccgggaggga ggtggggggg tcagccccct | |
| 4501 gcccggccag ccgccctatc caggaggtga ggggcgcctc tgcccggctg cccctactgg | |
| 4561 gaagtgagga gcccctctgc ctggccagcc gccccgtccg ggagggtggt gggggggtca | |
| 4621 gccccccgcc cggccagccg ccccatccgg gaggtgaggg gcgcttctgc ccggccgccc | |
| 4681 ctactgggaa gtgaggagcc cctctgccca gccacgaccc cgtctgggag gtgtgcccag | |
| 4741 cggctcattg gggatgggcc atgatgacga tggcggtttt gtggaataga aaggcgggaa | |
| 4801 gggtggggaa aaaattgaga aatcggatgg ttgcggggtc tgtgtggata gaagtagaca | |
| 4861 tgggagactt ttcattttgt tctgtactaa gaaaaattct tctgccttgg gatcctgttg | |
| 4921 atctgtgacc ttatccccaa ccctgtgctc tctgaaacat gtgctgtgtc cactcagggt | |
| 4981 taaatggatt aagggcggtg caagatgtgc tttgttaaac agatgcttga aggcagcatg | |
| 5041 ctcgttaaga gtcatcacca ctccctaatc ttaagtaccc agggacacaa acactgcgga | |
| 5101 aggccgcagg gtcctctgcc taggaaaacc agagaccttt gttcacttgt ttatctgctg | |
| 5161 accttccctc cactattgtc ctgtgaccct gccaaatccc cctctgcgag aaacacccaa | |
| 5221 gaatgatcaa taaaaaaaaa ataaataaat aaataaataa ataaataaaa taataaaaaa | |
| 5281 taaaaaataa aaaaatggga gtttgcctgc agaagttctc tctttgcctg ctgccatcca | |
| 5341 tgtaagatgt gacttgctcc tctttgcctt ccaccatgat tgtgaggctt ccccagccac | |
| 5401 atggaactgt aagtccaagt aaacctcttt cttttgtaaa ttgcccagtc tcgggtacgt | |
| 5461 ctttatcagc agtgtgaaaa cggactaaca caacgcccct ctttaggggc cagagacaat | |
| 5521 gagtaccagg cccatcccac gtccccacac ccccactgaa gctgcagcct ctgctgccca | |
| 5581 cgcccaatac ccctactata ttcctcttcc tcctgtcctt cttatctctc tttatgagca | |
| 5641 cactgtgcat cctgcacaga agcacagccc tctagaatca gcctttctaa agaaagcatt | |
| 5701 ctcatagctt tggcgaacag tcttatcctg ctcactttgt tttgattaca ctcggtctaa | |
| 5761 caggattcaa aagtgaacct cacgcaacat aactcatcct cgcttcctgg caacaagaat | |
| 5821 gttggcaggt ggaccctctt cacctttcct gccaccagcc ccaactcttc ctcaaagcct | |
| 5881 gagcttcccc cttccccacc cctttctcca cactctagaa tgcccagtgt tgatcttacc | |
| 5941 ttgagggctt tgaagacagt gacaccatag aacttttcat tgggagtatg tggggaggtc | |
| 6001 tgaccccggt agccatctcc tgaggttgct gccacctaca aggcccaaaa cacaaggtga | |
| 6061 tgttagccag ggcagctgag ggcttgagaa gaggtggtca actgtggcca ggaagaggaa | |
| 6121 ggcgaaggct accacccaag gggcacttgg tggggggatg ctggaccctt gggggaaggt | |
| 6181 gcagtcactg ggcttccctt acattggtca gtctctgcag ctcctgacac tcgtggtcag | |
| 6241 agattacgcc gtccatcacc acccgctggg aaccattcag gagtttggag ttcatggtga | |
| 6301 gactgatgcc ttcatacagc agggggccac ctgcaaagca atgacaaaac tctagctaaa | |
| 6361 tctagcagca gctagaccaa agactctcca ttggatattg cctcctcgtc acaaatagaa | |
| 6421 attccagctg tttccttttc accagcaaaa tctcccgcta ggtcattttc aaaggtagaa | |
| 6481 tagtccagag gacagaacat agagctgaaa gatgagattc ccagtcccag ctccatgacc | |
| 6541 aaatagctgc tggcttttgg gccatgacac actcttgagg aaacatcaga aacacaatcc | |
| 6601 taaaacatga gctcgggaac gaggaagaac tgactttgac tcctagatcc tccattgatt | |
| 6661 agctgtgtga ccttggcaag ttacttaacc tccctgaggt taattttaat taattaaaat | |
| 6721 gttctcatct gtcaaacagc atctacctta tatgtttgat gccagtataa tgttaagaac | |
| 6781 aaggaaggca agttaaaagc ttagtacaat atttggcata tggtaagtcc tcaaaagttt | |
| 6841 ggctaccatg gtatcatcat cattattcat ttaaaataag gatcttgcaa tctgcatgac | |
| 6901 tcacctctca gttataagaa tcaagagaaa taacaaagtg aaagtggtct gaaaacaaag | |
| 6961 gaatgctgga caaattcttt tcatacgcta atcttgttta attaaccact gttaatttaa | |
| 7021 gcaactatag ggcaaaggtc aacatttctc tcttacagtt ctcatggtgt ttctctgtgc | |
| 7081 tagggacata ctaatactag agactcagtt caactccact tgataactga ggcctcagtg | |
| 7141 tgtaccagga actgtgccag atagggtgag agatgaatcc tagaagacag tacctgttct | |
| 7201 ctaacaatta tgcaaataac tagcataaga cagaccataa atgccctaag aagttcaaaa | |
| 7261 gaaaggaggg gactccaatc atctgggagg ttaagaaagg ctttacggca gggcacagtg | |
| 7321 gcttatgcct ataatctcag cactttggga ggccagggcg ggtggatcac aaggtcagga | |
| 7381 gtttgagacc agcctggcca acatggtgaa accctatctc tactaaaaat acaaaaaaat | |
| 7441 tagccaggca tggtgccaca tgcctgtaat cccagctact tgggaggctg aggcaggaga | |
| 7501 attccttgat gatcccggga tgtggaggtt gcagtgagcc aagattgcac cactgcactc | |
| 7561 cagcctgggc aacagagcaa gactccatct ccaaaaaaaa aaaaaagaaa ggctttgcag | |
| 7621 ggtatatctc agatgccctc atgggcaggg atggtgggaa gcgtactagg agagccaggc | |
| 7681 ataaacacag actggtgggc agtgggcggg ctcacggaac aaagtgcagt cctgtgtgaa | |
| 7741 gagacggtgt aaacaggaca agagtagaac attcggttaa ggggcaggca cggaggatgg | |
| 7801 gtcagaggcc agtgtctgga agactggcct agctaccttg tgtgggcagg ggcaaggaca | |
| 7861 ggccagacgg ggaaagggtg ggtgcagata ggaggtgcca gggcctgaag gaagcctgag | |
| 7921 gcaatgaaaa acggaaaagg ctggggagag agctgagaag cactgcagag cagagccaca | |
| 7981 atcagtaggc cttcccagca gcttgcagaa aacgggcaca ggaggaagag gagccaaagg | |
| 8041 aggcctgaag gcgttgagtc tcagtaaagg gacgaatggt gaaaaccggg agaaaacagt | |
| 8101 ttgggagtgg ggagacagga tgagttaata cctatggaag tggacaacac tagtcactca | |
| 8161 tgtttctgtg acacagaagt ctcatttctt tctttttttt taaagatgag gtcttgctat | |
| 8221 attgcccagg ctggattcaa actcctgggc tcaagcgatc ctcctgcctc agcctgccaa | |
| 8281 gtagctggga ttacgggtgt gcaccactac atcctggagt ctgatttctg agccaaaggc | |
| 8341 tgattctcag tgctctttcc agctgaaagt cccgctggtg agttaggaag gatgtccact | |
| 8401 gaacttgcac cctaagggtg gctggctcat ggtgagctct gcaatgctgg ggtggagcgc | |
| 8461 tgcctggcaa atgtggggtg acctgcctgg ctccctgaca acagccagac atgcccctat | |
| 8521 ttctgtcctc tcaccttccc gggtcagtct gctcacatcc agtgactcct tggtcttctc | |
| 8581 ttccacaagg gtctcgattt ccttcataag gttcccaatc tcctgggaga tgcgtacggc | |
| 8641 tgtttcccgt tctgacctat gagcacagcc gctctgagga ctgcattcca gggccagccc | |
| 8701 tgggctccac cccgacttcc caaactggac ctgcccaccc cttgccagga gttcacatct | |
| 8761 gcctgggctc aggaagcctc aagtgcttca tcagactgat ccaaccatgc agtttcttca | |
| 8821 aggtcctcac ttctgtttct cttgcaatct cttgggaatc acttcttctg gagtccatga | |
| 8881 atcctaagta ggtcaggaag aaatgaaaaa ggtaagatga tctacttaat cttccaggtg | |
| 8941 tctcaatgca taaaataagg cccacaggag taatccagct tatcaccctc ttcctttcag | |
| 9001 gagacaaaat gggaggtttc cacatctaag tcccttctag ctgcccagtg ttcccctttc | |
| 9061 atctgtctct catccctgcc ttgcacagat cccaggctag gctcagcctc cagcaagttt | |
| 9121 tctctcagaa tcgcacagtg ttcccctcct ccccattcat ctctcctgct cactctttcc | |
| 9181 ccgctccctt ccggctcctg taccctagct caccggatcc acaaagggaa ttccaaaaac | |
| 9241 atcataagcg aagaaaagca gttctttttc cagtaggctt cgctgtcggt actccttggc | |
| 9301 actctgaggg taaaaaagca aacaaatccc ccagaactca gccctgtctt ttaacctctg | |
| 9361 ggagttcccc tccctggcac cccacactga tgctcccatg tgacctgagg cctcctctcc | |
| 9421 ctaccaatgc tctctgtctg ttccccagtt tgagacaaaa gtctaatttc tgtgtcctct | |
| 9481 gcctggcaca agcaacagta cctctgaggt actggaataa ccaggctttc cttaagggaa | |
| 9541 aaagccctgc tcctccccca tcccaacccc cattagtctc taggacagat aatctgatga | |
| 9601 ttctcaaaag tggaaatatt cttcaaagaa ggagaattcc ttcttgactt ctacaggagt | |
| 9661 ccacaaagca ggaaaaggaa tcaggcatta ggcaactgac atgttattca tttgatcttt | |
| 9721 tatagccaca tctgaggttg atactagtat gctcattcta cagaggaagc atctgaggca | |
| 9781 cagaggagat gtaccactgt ctaaggtgac actacaggac atgggagagc agcgacctac | |
| 9841 agtcaaaccc aggtcttctg ttatcttttt cttttttttc atagcagaat gtgcatttgg | |
| 9901 acagataaag ccgggtcttt acagtcctaa agcccatgtg ctttctgctc tgccacctgc | |
| 9961 ctcctctcag accctaacca gaccacaccc ctccctgagg ataagcagct aaaggggagg | |
| 10021 gcctggcact ctgggcttcc cacatggact cccaggcaag gcttacccag ggcagggaaa | |
| 10081 ctgagtactt tccggggtca catctttccc taggtgccct gctaatctcc aagcagcagg | |
| 10141 aggaggaagg aagagaaaaa ggagacatca gttgaaataa aacatgaggc agttcaagga | |
| 10201 ggaattaaag gagtcaatga atcctatgtc atctactata tttacctaag aggaagagag | |
| 10261 acgttttctc ttcaggagtt tacattactg gcttctgtca ccaaatgcta tgttatctca | |
| 10321 tttaaccctc tccaatgacc ctataaggct gctatgaagc aaaccccact tccatcttta | |
| 10381 ttcattcgat gctgatttac tgagtgtcag ccatgtgtct ggagctgtgc ctgcactggg | |
| 10441 tccaagcagg gaacagattc cggtcaccac tgactggcca caagccactg cacaagttac | |
| 10501 ttcatctctc agatgctaac agtcctcatc cctaaagtgg gaagaagagt gccaaccttg | |
| 10561 cagggtggct gtgatgatta aatgagatga tggatacagc ccaccccgca cagagcttcg | |
| 10621 cctggacagc ggctcaacaa atgttagttg ccttctttcc aaaaggggac ttctagttaa | |
| 10681 aaatgctaga ttggccacgt gggtttattt cccctcactc ccctcagact ccccttaatg | |
| 10741 gccataagga aggcagggaa aaggtattaa ccaaacaaga caaaagagac cggaagtgcc | |
| 10801 atcttaaagc cagatggtca ccatctagtg gctaaagagg acaactacaa tgacaaccca | |
| 10861 cacaggaggg gcagcaagag gctgtttcta ctggaggctc tcagactcca gagctcggaa | |
| 10921 gaagagcctg ggtgtctcct ttcagcgcag cagtctttgt gttatacaga tatttctcct | |
| 10981 ggttttggag agagacaggg aaaatgctga aagatgggag agaacaaaca atggcaggaa | |
| 11041 gaagaagaga tgggaaagaa aatggagaca gaaaacaggt gagcagaaaa aaaacaacca | |
| 11101 gtattaagtg aaaaatgata gctcttggcc aaataatatc gagtgtccaa gttaggggtg | |
| 11161 ccaagagggg aaagaatatg ctcaccgcgc agttctgcgg gactcagaga atagacttct | |
| 11221 gggcccagat aacgctaatt gaggctcctg tgtactcccc acactccctc cctgtgctcc | |
| 11281 cttctcctct gaataacaag ccgagtgact gaactcacat ctggccccaa cactgacatc | |
| 11341 tggcccctgc cctgcacgca cagcgcctac tcccctctgc taccccactc ttccgccctc | |
| 11401 aggtctgagt tcagctggca gctgtcataa cagaaggaag tctctcacct cacgggggcc | |
| 11461 gatggatctg gtgtgttctt ctccaagcat agctgcataa taggccaaat tttggttcat | |
| 11521 cacctcgtca ttggggaaga agagaagata ggtcttggca cattcaacag cctgtgtata | |
| 11581 attcccaact gcaaagtgga aagaagaatg gctgagagga aagagtcaaa aggaaaggat | |
| 11641 aaaatcctag caccagggac cacttagtgt tcagtctcca caaagcttga ggcaatggca | |
| 11701 gcatgacaag ctggtgcctg ctacttaaca tgatggcctg atttcagggg caggagttaa | |
| 11761 gtaatacaca tagaatgtgt gattaatttt aaggtgaggt ggtcaatgtc ctcagggctg | |
| 11821 cctaacaatg aaacagaaaa gaatcagaat tctctgcatg cccacatctt ttaagattca | |
| 11881 caaataattc caatactcct taatattttg cctcaataca ctcaatcatt agatcaattt | |
| 11941 ccctgtcttc ctaccttctt ttttctatag attttcagaa gacaaggaag attacaatag | |
| 12001 cagattgtct ctttggcaaa atagttcttg ttgatatttg ttagtcatct caactcaact | |
| 12061 gtaaatattg tacactcatc aatatgtttg taatgcttag cacacagtag ggactctgca | |
| 12121 aagagttggt tttctgggta ttaattggca gccccacaaa agcatcagtg ctataccagg | |
| 12181 gagagggcta gcaaatgtcc tctgagctag tttaaactgt ggcataccct tattttgcta | |
| 12241 ttaactttaa ttcaaaagca aaaatagatt acaagttata gaagggacaa gagatgaggt | |
| 12301 cctaagtctc cctcccccag ctttttgggt tgctggtatt ggcttcctag gccagttgtt | |
| 12361 tataacaaac tacccaggga tcacttggca tggtgcctcc catagctact gaaataagcc | |
| 12421 aaacaccttg aggaagtaag tggctgtaat ccctgcccac cttgccttta gcttcttagt | |
| 12481 tagccagcag agaaagagga aggaaagtag gccttactgt tatagtaggc aaactgcaga | |
| 12541 taattataat gcgatgggag gaagtcttca aagggcttct ctcgacttgg gtgggaagca | |
| 12601 agctccgtga cacagttctg cttacagttg aggacctgga tgtaatgatc tgaaaggaat | |
| 12661 caaacagaag gaataactat gaggtcactc tttagaactc aaattctggt taagatgaac | |
| 12721 aaattcaaat ggaagcaatt tttcatcagc ttctcagatt taataaattg aggaatgagg | |
| 12781 gtgaagagtc cccatttata ttatcagatg gtgaccttag agtcccagga ggcctggctg | |
| 12841 aaggtctggc tctgaactgc acgcacctgt gatggcctgg aagaggtcag cgttgtactc | |
| 12901 aaggtagttg tagccatcgt agtcataggg cccttcgcag agggcacggc actcctcata | |
| 12961 ggccacaaag tattcttgca gcgccgcctc taggtggggc acagcttcct gtggctgttc | |
| 13021 ctctgagtag agtcgcactc ccagtcgaaa ttcttgctac tgggaagaag gagcactcaa | |
| 13081 atgcaggcca cagaggagga accctctctc cttgccctca gccaccttca caggggtcct | |
| 13141 aatttcctgt tcctcaggtt atggatggaa aggggtgacc tgggtaccac tataaaatga | |
| 13201 aggccttttg taaatggtct ttacaaaata tttagctctg gaaatttaaa aacatttagc | |
| 13261 tctttttttc tttttttgag acagagtctc actctgttgc ccaggctgga ctgcagtggc | |
| 13321 acaatctcag ctcactgcaa cctccacctc ctgggttcaa gtgattctct tgcctcagcc | |
| 13381 tccctagtag ctgggattac attgtgccca gctaattttt gtattttagt aaagacgggg | |
| 13441 tttcaccatg ttggccaggc tggtctccaa ctcctaacct caagcaatcc tcccaactcg | |
| 13501 gcctcctaaa gtgctgggat tacaggtgtg agccactgtg cctggccaaa aaacatttaa | |
| 13561 ctcttaatct agatgaaaga tatctaggag ttcattatga ttttcttgta cttttcttcc | |
| 13621 ccccccatta gtgacagatt cttgcacttt tttttttttt tttttttgag tcggagtttc | |
| 13681 actcttgttg cccaggctag agtgcaatag tgcgatcttg gctcactgca acatccgcct | |
| 13741 ccagggttca agcaattctc ctgcctcagc ctccagagta gctgggatta caggtatgcg | |
| 13801 ccaccacgcc tggctaattt tgtgttttta gtagagatgg ggttttgtca tgttggtctg | |
| 13861 gctggtcttg aactgctgac ctcaggtgat ctgcccgcct cggcctcccg aagtgctggg | |
| 13921 attacaggca tgagccaccg tgcctggcct aggttcttgc acttttttgt agggctaaca | |
| 13981 gttttcaaat aaacacaact tactttttgt tttgttttgt tttgagacag agtttttttt | |
| 14041 tgagacagac agcccaggct ggagggcagt ggcatgatct cagctcactg taacctccac | |
| 14101 ctcctgggtt caagtgattc tagcgcctca gcctcctgag aagctgggat tacagacata | |
| 14161 caccaccaca cctggttaat ttttgtattt ttagtagaga caggatttca ccatgttggc | |
| 14221 caggctggtc ttgaacttct ggcctcaagt gatccactca cctcggcctc ccaaaattct | |
| 14281 gggattacag gcatgagcca ccacgcccgg ccaacacaac ttactttttt ttgtgtgtgt | |
| 14341 gatggagtct cgctctgtca cccaggctgg agtgcagtgg cacaatctcg gctcactgca | |
| 14401 acctccgcct cctgggttca agcgattctt ctgcctcagg ctcctgagta gctgggaata | |
| 14461 caggcacaca ccaccacgct cagctaattt ttgtattttt agtagagaca gggattcacc | |
| 14521 atgttaggca ggacggtctc aatctcctga ccttgtgatc cgcccgcctt ggccttccaa | |
| 14581 agtgctggga tgacaggtgt gacccactgc acctggccac aacttacttt taaaagtcat | |
| 14641 tctttttttt tttttttttt tgactgttgc tctgtcaccc aggctggagt gcagtggcac | |
| 14701 aatcttggct cactgcaacc tccaccttcc aggttcaagc tattctcctg cctccatgtc | |
| 14761 gagtagctgg gattacgggt gcatgccacc atgcctggct aatttttgta tttttagtag | |
| 14821 agatggggtt tcaccatatt ggccaggctg gtctcaaact cctgacctta agtgatccac | |
| 14881 ccacctcggc ctcccaaagt gctgggatta caggtgttag ccactgcgcc tggcctataa | |
| 14941 gtcattctta tattgtttct aacattccta ttttataatt aaaatgtaag gaaccataaa | |
| 15001 ctctctataa aatgctatat catcacagac agtccctcct ttggaacttt tcctgctaaa | |
| 15061 caaatagaat gagaacattg aaggatactg taacatttat tgtagtgaaa atcaaaaaca | |
| 15121 tacatgacca aaaaaatgtg actagtgaat taatatagtc tctgcataag atggactatc | |
| 15181 atgcagccat aaacaattat gttttcaaat aaaactgaag acatgagaaa agactcatga | |
| 15241 taataatgtt agttaaaaaa tgatgatggg gctggccatg gtggctcaca cctgcaatcc | |
| 15301 cagcactttg ggagcccaag gcagtaggat cgcttgaggc caggagtttg aaatcagcct | |
| 15361 gggcaacaca gtgagacccc gtctctacaa aaaataaaaa caatagctgg gtatggtggt | |
| 15421 gcatgcctct agtcccagct atttgtgggg ctgaggcggg aggatcactt gagcccagga | |
| 15481 ggtcaaggct gcagtgagct atgatcacac cattgcactg cagcctggac aacaaagtga | |
| 15541 gaccctgcct caaaaaaaat aaaaaagatg ataaaaaatg catacatgat ataacaccaa | |
| 15601 attagatgaa aaaattatat acggaagact taaaaaatag ggcaaaatat aaactggttt | |
| 15661 tcttaaggtg gcggtattac aaatgattac tttttatttt atacttacct acatttctac | |
| 15721 aatgttatac aatgaacatt cattacttct gtaatgtaat tagaaaacaa ccttagtaat | |
| 15781 gtcatttaaa aaaaaacaaa agaatgcatg cctgtagtcc caactactca ggaggccgag | |
| 15841 actagagggt ctcttgagtc cgggaatttg aggttacagt gagctatgat caagccactg | |
| 15901 cactccagcc tgggtgacag agcaagactc tgtctctaaa ataaatttaa aaagaaagaa | |
| 15961 agaaagaagg ggataaagtt tttttcacca tatggggttg agtctcaaga tccttgaagt | |
| 16021 cggcctcctt cactccagac atggtttggt aatagtctag gttctgctgc atttccatgt | |
| 16081 gctcaggatt gcccacgaag aaggtgtgtg ctgcagcaac agctttctcc aacttgttga | |
| 16141 tctaagaatg aagcatgaga aggtggataa tgctcacatc caatctctga acgtttgtgt | |
| 16201 ccacatgagc agtccacata aacaggaaat ccctgcctcc ctgagccctc cactcagcgt | |
| 16261 gcccagcccc tgccagcccc aggaagcaca cctattgcag gaactaccca cgacagatta | |
| 16321 ggagcatagt gccctgggca gatttgtagc tctttcccaa gtcagattta ggggttcccc | |
| 16381 agattacagg aagtagcaat gttccatttt aaaacaaagg tcatattgta tggggagtgc | |
| 16441 tgtgaaagac tgactgccca cataatatgt tttcatgctg ggtgcggtgg ctcaggtctg | |
| 16501 taatcccagc atttttagag gccgaggcag gtggatcact tgagctcagg agttcaagac | |
| 16561 cagtctgggc aacatggtga aaccctgtct ttacgaaaaa tacaaaaatt acatggtact | |
| 16621 atatgcctgt agtcccagct actctgcagg ctgaggtggg aggacgcttg ggcctgggaa | |
| 16681 gcagaggttg cagtgagcca agatggtgcc acagcactcc agcttgggtg acacagcagg | |
| 16741 accctgactc aaaaaaaaaa aaatacatat atatatttca tgaatcaaat gtaatgcctg | |
| 16801 agctgaagaa attctgtggc ttcaaggatt taggggtaga gaaaaaagag cagctaacag | |
| 16861 actatgccta agaattttcc tagaaaaaaa tttttttttg cataaaataa aatgaagaat | |
| 16921 ttttgagaca tttagggaaa aggtggatgg ctaaaaattc taaggtcctt tactaagtcc | |
| 16981 agacatagta ttttctgact caaagctcag ggctcatgag gttcaaacaa atagctctaa | |
| 17041 gaaatctgag ggggtggtcg ggggcggtag ctcatgcctg taatcccagc actttgggag | |
| 17101 gccgaagggg gcagatctct tgaggtcagg agtttgagac cagcctggcc aacatggtga | |
| 17161 aaccccgtct ctactaaaaa tacaaaaatt agctgggcgt tgtggcgcac acccgtaatc | |
| 17221 ccagcttctt gggaggctga agcaggagaa tcgcttgaac ccgggaggcg gaggttacag | |
| 17281 cgagccgaga tcacagcacc atactccagc ctgggtgaca gagcgagact cttgtctcaa | |
| 17341 aaaaaaaaaa aaaaaaaaaa aaaaaagaaa tccaaggggg gagaaatagg ctacagacca | |
| 17401 caatgcccac agcaatggac tccagcatgt caaagtaaca caggtttcat atatagaatc | |
| 17461 caaccaaata aatcactgat gatctagtaa ttaactatgt ggacactggt atcaaagaga | |
| 17521 ccagccgtgg ccaggcacgg gggttcacgc ctataatccc agcactttgg gaggctgagg | |
| 17581 cgggcggatc atgaggtcag gagtttgaga ccagcggatc atgaggtcag gagtttgaga | |
| 17641 ccaacaatag ctgggcgtgg tggcatgtgc ctgtaatccc agctactcag gaggctgagg | |
| 17701 caggagaatc gcttgaaccc aggaggcgga ggttgcagtg agccgagatc gcaccattgc | |
| 17761 actctagctt gggcaacaag agcgaaactc cgtctcaaaa gaaaagaggg ctgggtgcgg | |
| 17821 tggctcacgc ctgtaatccc gcactttggg aggccgaggc aggtggatca caaggtcagg | |
| 17881 agatccagac catcctggcc aatatggaga aaccccatct ctactaaaaa tacaaaaatt | |
| 17941 agccggatgt gatggcacgt gcctgtaatc ccagctactc gggaggctga ggtaggggag | |
| 18001 tcacttgaac cagaaaacca gaagtcagag gttgcagtaa gccaaggtcg ccactgcact | |
| 18061 ccagcctggc aacagcgaga ctccatctca aaaaaaaaaa aaaaaaaaaa gaaagaaaga | |
| 18121 aaagagacag gccagcatca catctgggct taatgactca ctgactggct gggcagtaga | |
| 18181 caagatactt aaagtttact ttctaaactc agtttcccta atctatcatc tggagatagc | |
| 18241 attctctagc tatctcctag cattgttgag agttcaacga gataatatgc agcctgtaat | |
| 18301 acatggtgct gggcacagat taagtgctca ataaatttta ggtggtatga ttaatatgct | |
| 18361 aagaaaactt attttcttat cattggtact agtaaaggca cactaaagga gtcagccctg | |
| 18421 gttaggtgac ccaaggtaaa taactcccca tctctgtacc tcagtttctt taactgtaaa | |
| 18481 attagaaatt ttttcaggtc tgtccctttc atggaattac tctagctctt gtgtctttca | |
| 18541 gcgctgctcc tgcctttctc tgttctccct catgtcctca ctcctctctc cacccccagc | |
| 18601 cttctttact ctctaagcac tgaggtactt aacctcctct cttgtggaca aatgtctgcc | |
| 18661 tcctaatcaa gtgccaccac tttctccttc ttccaaggct cattcaccat ggcttccaga | |
| 18721 ataagcgttt taaaacccaa atctgaggat ggctccctgc agtccgcaga attctcttcc | |
| 18781 aaatctggcc tcactgactt ttctagccgc ctctcctact actccccaca cacatctcga | |
| 18841 cattgcaatc acaagagttc attttttttc ctgaatacat catgctttgg ttctactttt | |
| 18901 ctctctatct ggaggagcct tctttcctct ccccctggca atacttaggg caggcttcaa | |
| 18961 tattacctct tctttgaagt ctaatttctc tgagataaag ctagagtctc cctcctttgt | |
| 19021 gctcccgcag ccctaagtat atgcccttgc agtagccctt actacagtaa ctcttaacag | |
| 19081 caaagcaaga caaagcaaag acgttagtcg agcatttaat tacttgtaca agaactggaa | |
| 19141 tgcagtaggt atgcaataca tgtttgtcaa atagttttag aaagtgctat gatgaagcga | |
| 19201 cataaccaaa gacataacgc aattattttg atttcaagtc cagcgctctt ccgagtaact | |
| 19261 aggaaaaatg aggggaactg tgtaggcaga atgctgtgga ggggtggaaa tctgaggtcc | |
| 19321 ccaagagtct ctaagtgtct ctttagatcg aatcgaaggt cttttaaaca acagatatac | |
| 19381 cagatataat gcacaactct ttctcccccc acccccaata cagtgggact aataacagca | |
| 19441 tctacctgac aagatattat gaagattagg gcaaatgcgt taataaatgc aaactgctta | |
| 19501 aaatagtgcc tggaagtatg tgctggctat catcatcatc atcatcgtca ccatcaccac | |
| 19561 caccaaacaa gcctggaacg ctccaagccc aagtgtacac ttggacacat ggcactctgg | |
| 19621 cattccgaaa aatactcatt ttctttgtaa acctggcaga ctagccagag ggtccccccc | |
| 19681 ccgcccccac acacacacag aaagggccag gcagcccctg ctggcgacgt ggcaacctca | |
| 19741 catcctgaaa gagagctgct gaatcaaaca gcaggaaaag caccactgga aaaccaaccg | |
| 19801 gaagggagat attgtagacc gctactagct tctcactcgg acacttacaa tgacatcaga | |
| 19861 atgggagacc caaactgccg ctttaattcc ttccaggaga tttcccaggg aacaaaatgg | |
| 19921 ccatgagttt tgcttggcct gtgccctaac cccaggctgg acctttgggg ttagcatctg | |
| 19981 cccatgcgaa ctgggcgagg aaggtctcct gccagggagc agctctcccc ggctccgagg | |
| 20041 agagctcccc agccaggagg ccactttccc ctccctgcgg acacttcccc gatcccccaa | |
| 20101 gctcctcagg tcccctgcaa cactcctctc cccagaagga ccccggccgc ctggttccag | |
| 20161 gcaggtctgc accttgaagt aggcgacctg caggtagttg taggggctcc gcttgcggaa | |
| 20221 ctccagctcc atctcttcgc tgagcgagtg ggcggccggc ggcccgaggc agcggcgcag | |
| 20281 gcaggcagcg cgacgcagaa ggcccccgaa gaagctcagg tcgcgcaggg cggcggcgcc | |
| 20341 cgaggcctgg gccgggctgg gggaccagtc ggggtccagc tcccacggga agtcggcggc | |
| 20401 acactgggtg cggcagcgca ggcgaagggc gcggagggct gcccgggagc gcagcgcccg | |
| 20461 ttccatgctc aggaccaccc cgggccagtc cccgcgcgcg taggctgcgg tcccctcggc | |
| 20521 gaagagcaga tcaggcgtca ccatgcccca tcctgcctcg gactcgacct cggcttggga | |
| 20581 ggcagcggcc acgacagcca gcagtgtggt cagcagcttc aacgcgcgta ccgccatcgc | |
| 20641 tccctcagac ctaacggaac cgccagccac ccgccaccaa ggccggagtc ctacccccgg | |
| 20701 cgaaggcccg cccccgggcg aagccccacc ttccaagccg ggcggcgcat |  |
