# Supplementary material for: Development of a High-Throughput Resequencing Array for the Detection of Pathogenic Mutations in Osteogenesis Imperfecta
Source: PLoS One. 2015 Mar 5;10(3):e0119553. doi: 10.1371/journal.pone.0119553 (PMC4350936; doi:10.1371/journal.pone.0119553)
Supplement: S2 Datasets — Including all the reference sequences of COL1A1, COL1A2, CRTAP, LEPRE1 and FKBP10. (ZIP) [file pone.0119553.s002.zip › OI-col1a1_sequence.doc]

| 1 ttatgtttgg gtcatttcca catgctttat tccagcaatc aaaataatta aaaacatctc | | | | |  |
| --- | --- | --- | --- | --- | --- |
| 61 aaattattat acacatacaa aataggtaca gagtcttttg cttcctccca cccctagggg | | | | |  |
| 121 gaaaaactgc tttgtgcttt gggaagttgt ctctgaaacc cggggacaga ggacgcagga | | | | |  |
| 181 cagactagga gggagccggg aggatgggct gcagctgtgg aggagggttt cagaggagag | | | | |  |
| 241 aggtcggaga gcagaggcct gagaagccag aggcaggtgg agagagggtg gaaagtgagc | | | | |  |
| 301 agcgggctgg gctggagccg cacacgctct cctcccatgt taaatagcac ctttagaaaa | | | | |  |
| 361 attcacaagt ccccatccac aaaaaaaaaa aaaaaaaaag aaaaatatca aggaataaaa | | | | |  |
| 421 atagactttg aacaaaaagg aacatttgct ggcctggggg ggcatctcaa tttctatagc | | | | |  |
| 481 accagtgatt ccctccccac cccacccatc acatagatgt agcacctttg gtataaaatg | | | | |  |
| 541 gggagccgct tccaccctgc ccccatcccc gcccccaggc agttgccccg gtgacacatc | | | | |  |
| 601 aagacaagaa cgaggtagtc tttcagcaac acagttacac aaggaacaga acagtctctc | | | | |  |
| 661 ccgcccagcc ctgcggcaca agggattgac acgcgttccc caaatccgat gtttctgctt | | | | |  |
| 721 tgtcgtggcc cttcctgact ctcctccgaa cccagtgagg ggctggtggc tcccccggca | | | | |  |
| 781 tgaccccctc aaaaacgaag gggagatgtt gcaagagcca tgggagcgcc agatggcaag | | | | |  |
| 841 gcttctttgg cagtctgaga accccaggtc ccccagggcc tgggggtgct gggcgggcag | | | | |  |
| 901 gagcgggctg agggtggggg ccacttgggt gtttgagcat tgcctttgat tgctgggcag | | | | |  |
| 961 acaatacatt gtttcctgtg tcttctgggg agacagattt gggaaggagt ggaggggagg | | | | |  |
| 1021 ccccaagggg ggtgtggaga aaggagcaga aagggcagca ttggggtttc ataagcccaa | | | | |  |
| 1081 cgggcagaaa gggacttacc cccgcatggg tcttcaagca agtggaccaa gcttcctttt | | | | |  |
| 1141 ttaaaaagtt atttatttat tctttttttt tttttttttt tggtaaggtt gaatgcactt | | | | |  |
| 1201 ttggtttttg gtcatgttcg gttggtcaaa gataaaaact aagtttgaga gatgaatgca | | | | |  |
| 1261 aaggaaaaaa atattttcca aagtccatgt gaaattgtct cccatttttt ggcttttgag | | | | |  |
| 1321 ggggttcagt ttgggttgct tgtctgtttc cgggttgggg ggaaagttgg ttgggtggga | | | | |  |
| 1381 gggagccagg ttgggatgga gggagtttac aggaagcaga cagggccaac gtcgaagccg | | | | |  |
| 1441 aattcctggt ctggggcacc aacgtccaag ggggccacat cgatgatggg caggcgggag | | | | |  |
| 1501 gtcttggtgg ttttgtattc aatcactgtc ttgccccagg ctccggtgtg actctggggt | | | | |  |
| 1561 ggggcggaga caacgggagg gggcattacc acgtgggagt gatggagaga gggcactatg | | | | |  |
| 1621 gcctggccaa aaagcccaag gccggagagg tggggccctg cctggggatt ctgggcactc | | | | |  |
| 1681 accgtgcagc catcgacagt gacgctgtag gtgaagcggc tgttgccctc ggcgcggatc | | | | |  |
| 1741 tcgatctcgt tggagccctg gaggagcagg gccttcttga ggttgccagt ctgctggtcc | | | | |  |
| 1801 atgtaggcca cgctgttctt gcagtggtag gtgatgttct gggaggcctc ggtggacatc | | | | |  |
| 1861 aggcgcagga aggtcagctg gatggccaca tcggcagggt cggagccctg gccgccatac | | | | |  |
| 1921 tcgaactgca ggggagggga gagagggaag agtgagccgc tatgcgggaa cctctagtcc | | | | |  |
| 1981 tgcctggcct ccctgtccag ggtcctcaga gagctgccca atgcaccgtt atatcgagag | | | | |  |
| 2041 gaggcaccac ctgcccatcg gcagcctgtg tctgaaccac tatcagggac ctgagacccc | | | | |  |
| 2101 tgcctcccag cccagctctg tccatcaccc ttagcagaga cctactccag gacttcatgt | | | | |  |
| 2161 cccttctgag cactgggcta gcccatctcc taacactggc tctgaggtcc agctcacgca | | | | |  |
| 2221 cctggaatcc atcggtcatg ctctcgccga accagacatg cctcttgtcc ttggggttct | | | | |  |
| 2281 tgctgatgta ccagttcttc tgggccacac tgggctgagt ggggtacacg caggtctcac | | | | |  |
| 2341 cagtctccat gttgcagaag actttgatgg catccaggtt gcagccttgg ttggggtcaa | | | | |  |
| 2401 tccagtactc tcctgtggta gggcagggca agatggagtc agggaaaggg agcagccagc | | | | |  |
| 2461 accatatggt aggggcacat atgggcatgg ggaccctggc atggcaggag taggagggag | | | | |  |
| 2521 ggagaggcta gggcaggccc tcaccactct tccagtcaga gtggcacatc ttgaggtcac | | | | |  |
| 2581 ggcaggtgcg ggcggggttc ttgcggctgc cctctgggct ccggatgttc tcgatctgct | | | | |  |
| 2641 ggctcaggct cttgagggtg gtgtccacct cgaggtcacg gtcacgaacc acattggcat | | | | |  |
| 2701 catcagcccg gtagtagcgg ccaccatcgt gagccttctc ttgaggtggc tggggcagga | | | | |  |
| 2761 agctgaagtc gaaaccagcg ctgggaggac cagggggacc aggaggtcca ggagggccgg | | | | |  |
| 2821 ggggaccctg cacagagagg gaagagagtg gggattaccg gcatccaagt gctttggggg | | | | |  |
| 2881 ctggagggcc atgagcagag gggatgaggg gctacataca acaggaccag catcaccagt | | | | |  |
| 2941 gcgaccgcga ggaccagggg gcccaatggg gccagggaga ccgttgagtc catctttgcc | | | | |  |
| 3001 aggagcacca gcagagccag ggggaccctg gagtggggga aatggtttga gaaaggctgc | | | | |  |
| 3061 cagaagcccg aacaacccca gctctggagg agaggcccac caccctccct gctggctctg | | | | |  |
| 3121 gccccacggc tcatagagcc agcctcagcc tctttccata ggacatgcca tagctcctcc | | | | |  |
| 3181 cagccgctga gccaggggag gacggattgg ggagcagaga ggccaaagct agatcagatt | | | | |  |
| 3241 gtttgttcag gattcttcct ctctttgccc actccctgtc cctgaaccct tctccagaga | | | | |  |
| 3301 ggcaaagggt gcctgggtcc ctggcaaggg tccccgaggt gagcctgggc ttggggctca | | | | |  |
| 3361 ggaagaggag agagaaggca tgacttactc ggggaccagc aggaccagag gctccagagg | | | | |  |
| 3421 gaccttgttc accaggagag ccctgaagga cagataaaaa aggcagttca ggcccagtga | | | | |  |
| 3481 gcgtcaaatg tagcctgagg gcctggctgg agagacaagg gcagtgtggg caacccatgc | | | | |  |
| 3541 cccttcatta ttctggaaat ctagcccgag ggtgtccata ggcagagacc tctcccataa | | | | |  |
| 3601 tccctctctg tgtacccctc accctctcag aagctacttg gacatgccca caaatcttca | | | | |  |
| 3661 gaccccagtc cccactaggg aggggaaaga atgactatcc agagggggaa actgaggcga | | | | |  |
| 3721 agctccccct cctatcccac agcacagcat ggggactggg gaggggctga gcatacttac | | | | |  |
| 3781 aggagggcca gggggaccct ggaggccaga gaagccacgg tgacccttta tgcctctgtc | | | | |  |
| 3841 gccctgttcg cctgtctcac ccttgtcacc acgggggcct tggggtccct agaagagaga | | | | |  |
| 3901 aagggacaaa ctgtcaggcg gaagttccat tggcatcgag tggggcactg tctgcatctg | | | | |  |
| 3961 tagagttcta aaggcatggg ggacacagca gggtacttac ggcggggcca cgggcgccaa | | | | |  |
| 4021 cagggccgac aggaccggtg ggaccagcag gaccctgggg agagcaagga aagcatgagc | | | | |  |
| 4081 tcttggccag ggaaggctga ggctggggct gcaggatgag gcctcccctc tgctggatct | | | | |  |
| 4141 ctctctcctc agctgcttcc cactgtggcc atctctccca actcccaggg aaacctcccc | | | | |  |
| 4201 actgcaatct tcacgggagc tggggccaac tcatgggaga ggcggtcctg tctgggagag | | | | |  |
| 4261 gggacttggg gctgagcttt aactcagttt tttggattaa ggccctgaca tcttgcagga | | | | |  |
| 4321 tctcctttcc tccccctgcc tgggtgaagt ccgacaccca tccccaggcc tctaaggagg | | | | |  |
| 4381 cctgaagagt ccctggcctg accaggtaca gggaactgga gcccagctac ttacagtctc | | | | |  |
| 4441 accacgatca ccactcttgc cagcagggcc aacggggcca ggggcaccag gagcaccagg | | | | |  |
| 4501 agcaccaggg ggtccagcgg ggccggtctc accacggtca ccctggcggg gagagcaggg | | | | |  |
| 4561 gaatatgggt cagccccggg tgaagggcca ggatggggca gggaagcagc agacaaggct | | | | |  |
| 4621 gtggtcatgg agtgttgcca tcttaccttg gcgccaggag aaccgtctcg tccaggggaa | | | | |  |
| 4681 ccttcggcac caggagcccc ctgcagagag agagagagag aagtgagagt cagccgggga | | | | |  |
| 4741 agagggctta ggcaaggcca caatggccat gctgagggta ctggcatggg ggctggggac | | | | |  |
| 4801 tgctcacctc acgtccagat tcaccagggg gtccagccaa tccagggggg cccatgggac | | | | |  |
| 4861 cagggggacc acgttcacca cttgctccag agggaccttg tttgccaggt tcaccctaag | | | | |  |
| 4921 ggagaagaaa gagtcaggcc agagataggg tctgggagga cccttgagtc cgctggagtc | | | | |  |
| 4981 atctctacca aatctgttct ccttggctcc gccccactcc aagtcctgtg atggtttttc | | | | |  |
| 5041 tcagggcccc ccaaggtgag gggggcactt acagaggggc caggaagacc agggaagcct | | | | |  |
| 5101 ctctctcctc tctgaccagg caggccgacc acaccacgct gtccagcaat accttgaggc | | | | |  |
| 5161 ccgggagtac caggagcacc ctttgggagg caaacagggg tgaggtgcca gagagcagca | | | | |  |
| 5221 cagggacccc tccccagctc tgcacacctc cggagctgca gagatctgag ctggcactta | | | | |  |
| 5281 cagcaggacc atcagcacca ggggatcctt tctcgccagc agggccaggg ggaccagggg | | | | |  |
| 5341 gaccaacttc accaggacgt ccagcagggc cagtctcacc acggggacct ttgccgcctt | | | | |  |
| 5401 ctttgccagc aggaccagga gggccagggg gtccagcatt tccctggatg aggataggag | | | | |  |
| 5461 gggctgtcag actccagggg gctctggtgc atcattgggt cctcagtcag ccccaccatc | | | | |  |
| 5521 cttctggcag cccccaccca gcacccccaa cctagagcag tggactctgc tgcagagact | | | | |  |
| 5581 tacagagggg ccaggaggac cgactcggcc agcagcacca gggaaaccag tagcaccctg | | | | |  |
| 5641 gaaggagaga acataggaac agtcagaggg agaacagcca actcatccga cccagctgcc | | | | |  |
| 5701 ctcacctgcc accgctgcct ggggagaggg gagaggctca acagagaggc gggtgatact | | | | |  |
| 5761 cacaggggga ccagcgctgc cgcgagcacc tttggctcca ggagcaccaa cattaccctg | | | | |  |
| 5821 taggagagca cagaggcatc aagcctggac ccgtcctggg tcccagccca ccagcctcgt | | | | |  |
| 5881 gggcacagag ggccaagcca ctcacaatgg ggccaggggg tccagcgggt ccggcagggc | | | | |  |
| 5941 cagggggacc agcatcgcct ttagcaccag catcaccagg ttcgccttta gcaccaggtt | | | | |  |
| 6001 ggccgtcagc accctggggg aggaagcagg gcggtgaatg gagggaagga ggcaggagtt | | | | |  |
| 6061 tccactacct gggggaggag cagtaatgga ggcaggaaga tgcttgggtg ggaaacaatc | | | | |  |
| 6121 ccgtctccac ccttctcccc tgaggatggc tgacgccttt gtcctcattc cgtccctcga | | | | |  |
| 6181 ggtcccaggt cccagtcggt gatgaaaaat gatgggggtc ttggtactca caggggggcc | | | | |  |
| 6241 agcaaagcca gcagggccgg ggggaccagg ctcaccacgg tctccctaga agaaaaggag | | | | |  |
| 6301 tcagattgga gagatgcgct gacaggaggg aaggcgggga tgcggtgagg ggagaggcaa | | | | |  |
| 6361 ggggtgaagg cacagacagg gccaggggtg ctgtgtgaag ggagggaagg gccaagtatg | | | | |  |
| 6421 gggtcttaac aggtcttctg tacttacggg ggcaccacga gctccagtgg gaccagcagg | | | | |  |
| 6481 gccgctggga ccactttcac cctgagagca agggacaaga ggctcagggt cagggcctcc | | | | |  |
| 6541 cctgaatact cctagtagat gaccccagga gagcctcccc tccttctggt ccctccaggt | | | | |  |
| 6601 tcccaggttg acagctcagt ttggcaggac ctgctctccc aacgcaaccc cacggaaccg | | | | |  |
| 6661 tgcccaggcc tgctgaggag gctatgtgtt agggcagaag gtggggaggc ggccacctca | | | | |  |
| 6721 ccttgtcacc aggggcacca gcagggccag gaggaccaat ggggccagtc agaccacgga | | | | |  |
| 6781 cgccatcttt gccaggagag ccatcagcac ctttgggacc agcatcaccc taaagacatg | | | | |  |
| 6841 gataagcttg agatttccag tgtggggcaa ggaggtgacc tatagtgttc tgcttgtgtc | | | | |  |
| 6901 tgggtttcct gagaggccct ctgcagctct gccctgcctc cacctgggcc aaggactcaa | | | | |  |
| 6961 agattctttc aggaaaaagg gtgcctgcct ggagactcag gtggaactga ggttaagagg | | | | |  |
| 7021 cccctgactt gtgtagcggg atgaataagg gagcctccac caaggagatg cccccacctc | | | | |  |
| 7081 tcatcaccat ggtaacccat ggggatagat gggcctggac cacagggaga ggattcatca | | | | |  |
| 7141 gcaaaaaggc cagaggggaa aggggaagaa gggaggatta gcgagaagag ggacagatcc | | | | |  |
| 7201 cagagagaag gagagatgct gagagattca aagcaggcag agatgggagc catgtagggc | | | | |  |
| 7261 tcaggggagg gggaaggttg aacttactct gtcaccctta ggccctggaa gaccagctgc | | | | |  |
| 7321 accacgttca ccaggcattc cctgaaggcc aggggcgccc tggctaccgg gagctccagg | | | | |  |
| 7381 ggcaccagca tcaccctatg tgacaaccaa gaagactgga gtgaggcctg gggccccaag | | | | |  |
| 7441 agtgggtcac agccctaagc cccgcttggt atctcccagg cttgtttcca aggccaacga | | | | |  |
| 7501 caagccttga gaaaagatga aaagactcac agcccagact ccaggctgtg tgtgggcccc | | | | |  |
| 7561 tgccctggtc ttttcccccc accccacacc ctatctccat ggctttggtc atggcccgcc | | | | |  |
| 7621 atcccctgct gcaggagggg tgagacctgg tccctgggcc acttgccaga gccccttcca | | | | |  |
| 7681 cgctgccctc accttagcac catcgttgcc gggagcaccg ttggcccctc ggggaccagc | | | | |  |
| 7741 aggaccaggg ggaccttgca caccacgctc gccagggaaa cctctctcgc cctagaaggg | | | | |  |
| 7801 aaggacaggg catgtgaagg ctgctctgga gatagggcca agtacgacgc accttgacgg | | | | |  |
| 7861 atgcagcgag agaggcctac ttactcttgc tccagagggg ccaggggcgc caaggtctcc | | | | |  |
| 7921 aggaacaccc tgagggggag ggagagagga acagacagtg agcaaaaccc acctggggcc | | | | |  |
| 7981 actctgctgg aaagcacggt ccttcctcct ggggtctggc cgtgattaga gaggaacccc | | | | |  |
| 8041 ttctcagcac tgaattgaga ttatcccaaa cagcccctct cttcctccta gggatgtgtc | | | | |  |
| 8101 aaaggcttcc cccctcccca aactatggac caagatttat caatagaagg gttgagggaa | | | | |  |
| 8161 agtcacagca tattccagca gctcttaaaa gaagtcaccc agactagcaa tcatgcagcc | | | | |  |
| 8221 cccacttccc tctgtgctgc tcccttcatg ggaggaagtt ctttccggcg tctaacctca | | | | |  |
| 8281 atccctctag ttgatggctg tctgattagc taggagcggg ggcctgtccc tctggattcc | | | | |  |
| 8341 ctgcatctcc cacccctctg gccggctgct ccctcttacc tgttcaccag gtttgcctgc | | | | |  |
| 8401 ttcacctgga ggaccagcag gaccagggag accctgtagg tgggaaatgg gggaagaagg | | | | |  |
| 8461 gagggaaggt ttagaatctg gaagagacca aagaggtgtt tcctacccct acctcccagc | | | | |  |
| 8521 atcctgacag ccatgaggcc tcacctggaa tccgggggag ccagcagggc cttgttcacc | | | | |  |
| 8581 tctctcgcca gcgggaccct gcacagagag aacactacag tcacggggag gccgaggaga | | | | |  |
| 8641 cgaggggctg agggtgtctc cccttttctg ctccccagat ctccccatca gggacactca | | | | |  |
| 8701 cagcagggcc agggggtccc tgagctccag cctctccatc tttgccagca ggaccctgca | | | | |  |
| 8761 gggagagagc aaaggggaac tcagggttag gaggccccga gcagctgagg accgtggcct | | | | |  |
| 8821 ctagcacccc tcctgcaggg aggagaaagt gccggggcag caatgggaag gaggtaggga | | | | |  |
| 8881 tggaaaggag atacttacga cagcgccagg gggtccggga acacctcgct ctccagcctt | | | | |  |
| 8941 gccgggctct ccctgtggag aaagggagtt agggttgagg gggctgaagt gagaagccag | | | | |  |
| 9001 ggcctcctgg ggcctctcat ttactctgag tgggactttt taagggactt tcttttcaaa | | | | |  |
| 9061 agactgtttg gcccccgggg aatgcccctg caggtgcttc ttgctgggcc ctttctgagc | | | | |  |
| 9121 ccactcctcc caagatgccc ttccaggctc ctctgccttt tccctgttag atgcccgctg | | | | |  |
| 9181 tggcctgcac tgcaaagggc tttcatcttc tctgctatct ccttttggac ctcaaagaat | | | | |  |
| 9241 ccaccaagac tgggggtgcc tatcatatca aaggcctgtc ctatccccac gtgtcggggt | | | | |  |
| 9301 ggagggccac caacaacact tatttccagg acagagcagt ggtcatggag cctggagaag | | | | |  |
| 9361 tgtttcattt tttctttctt tctttcttct tttttttttt ttttgagatg gaatctcgca | | | | |  |
| 9421 ctgttgccca ggctggagtg caatggcgtg atctcagctc actgcaacct ccacctcccg | | | | |  |
| 9481 ggttcatgca attctcctgc ctcagcctcc tgactagctg gggttacagg tgcacaccac | | | | |  |
| 9541 cacacccagc taatcttttg tatttttagt agagacgtgg tttcactatg ttggcccgac | | | | |  |
| 9601 tggtctcaaa ctcctgacct catggtctgc ccgccttggc ctcccaaagt gctgggatta | | | | |  |
| 9661 caggcatgag ccaccgtgcc ccgccgagaa gtctttcatt ttacagatag ggaggctgag | | | | |  |
| 9721 gtccagaaag tgagagtgag tggccacacg gcaggtcagc ggcacagctg agcccagagc | | | | |  |
| 9781 ccaggactcc ttcaagtctc aggtgtgttt gtccctggct cttcatggat cctcacttaa | | | | |  |
| 9841 tactcacagc agcaccttta ggtccaggga atcccatcac accagcctga ccacgggcac | | | | |  |
| 9901 caggtgggcc tgggggtccg gggcgaccat cttgaccggc gggaccctaa ggatgggagg | | | | |  |
| 9961 cacgaaagca gcagtgagga cagcagggag gcagacagga caatggcagg gggttcaggg | | | | |  |
| 10021 ggagtgatac ttacaggggg gccagttttg ccatcaggac cagggctgcc agggcttcca | | | | |  |
| 10081 gtcagaccct agggaggcag agaggtatga gtgggacttg gggagaagca tgatggaggt | | | | |  |
| 10141 gggggaggac tccagagggc agacccttgg gcctgatcca gaacgcctca tcccagaccc | | | | |  |
| 10201 tacacgggat ggtcagggcc tggccaagcc aggctgaaag cctggggcct caccttggca | | | | |  |
| 10261 ccaggcagac cagcttcacc gggacgacca gcttcaccag gagatccttt ggggccagca | | | | |  |
| 10321 gggccaggag aaccacgttc accagcggga ccctggttgg gggaagtcac aggaacagtt | | | | |  |
| 10381 agggtctcaa gtttgtggct ctttgccacg ggccaaaaga ggaagaagat gcccagggag | | | | |  |
| 10441 cggcagggtc agccccccgg ccgcaaggag aggttacctt gggaccagca acaccatctg | | | | |  |
| 10501 cgccagggaa accacggcta ccaggtccac cctgcaggag gagaggaggc cagtgaactc | | | | |  |
| 10561 cgcgacacac aggcaccagc caggcaatga gggtggagcg ggagggggcg ggcagggaca | | | | |  |
| 10621 cttacacgct cgccaggggg tccgggcagg ccagtgggtc cgggttcacc tcgagctcct | | | | |  |
| 10681 cgctttcctt cctctccagc agggccaggg ggtccttgaa caccaacagg gccctggaga | | | | |  |
| 10741 gggccgagag gaggaggcgg cctgtggtga ggggccatcc tgtgccagcc tcagagccgg | | | | |  |
| 10801 ctgaggctgg gcctccagtg tcaggggttc ctgggggtgt ggcagggact cccccagaag | | | | |  |
| 10861 actaggggct cctcttcctt tctggatttc cctcaggggg ctcctagggc agggtgggct | | | | |  |
| 10921 gggctgcaag aaggatggcg gggagactta caggctctcc cttagcacca gtgtctcctt | | | | |  |
| 10981 tgctgccagg agcaccaggt tcaccctgca aggggggaga agaggatgag ctgagagtcg | | | | |  |
| 11041 ggggcgctca gttggcctgc gtcttcctgc tccccagatg agagccgcac tggagccagt | | | | |  |
| 11101 gcatggggtg ggcagaaggg agagtttggt actcacgctg ttacccttgg gaccaggagg | | | | |  |
| 11161 gccgccgggg ccctggggtc cagaggggcc tcgggcacca gggaagccag gagcaccagc | | | | |  |
| 11221 aataccagga gcaccctgtg ggaggcagac agccagggcg tgagcctagg agcagaggga | | | | |  |
| 11281 aaggggcagg caggctgcag gcggcaggag tgggactgaa gcctggcagg atacttacat | | | | |  |
| 11341 tggcaccttt agcaccaggc tgtccatcag caccagggtt tccctgtggc acagagaaag | | | | |  |
| 11401 gagtgtcagc aacaggcaag gactctgagg ttagaaagtg gcaaagggga cactgagtcg | | | | |  |
| 11461 gggacactta cagcagggcc agcagcacca gcagggccag gggggccagg ctcaccacgc | | | | |  |
| 11521 acaccctggg gaccttcaga gcctcggggc ccttggggac cagcttcacc ctgaatcaga | | | | |  |
| 11581 agaaaggaca tatcagaagc caccctggga aacccaacct tgcctctcgg gatggcagga | | | | |  |
| 11641 ggaaggggag acagggacag gagagcaatg atcaggactc taagatcaga gacggagaac | | | | |  |
| 11701 ccaggacaaa ggtgttagtg aacgggctgc tctcttgcca ctctgggtcc ctttggtttg | | | | |  |
| 11761 gggaacaggg agacatgaac cccttggccc atgagggtca tgcttagagg agagtggggg | | | | |  |
| 11821 gtctcacctt agcaccaaca gcaccaggga agccaggagg accagcgggg ccggtgggac | | | | |  |
| 11881 cctgtgaatg aaatggagat gtcagcgaga aggaagagat ggcagctgca agtcacaccc | | | | |  |
| 11941 tgggacagag gaagtcctgg ggttcagacc aacataacct gctcccattg tcagccccaa | | | | |  |
| 12001 gagcagatac tgagacccct ccccactccc aggccctgag gcctacaggc cacactcaca | | | | |  |
| 12061 gggggcccgg cagcaccagt agcaccatca tttccacgag caccctgcag gagagagggg | | | | |  |
| 12121 aagccccgtt aagtccactg agcactggcc agtccctaga gttcctgggg agccccttcc | | | | |  |
| 12181 agagctcagg gatcccccaa ggggccagga gtacttacag cagggccagg ggctccaggg | | | | |  |
| 12241 cgacctctct caccaggcag gccacggggg ccctgacaac caaaccaaga gaagtcagat | | | | |  |
| 12301 gagatgggag acagccttgt tcccccaggc ctccatcttg cccctgcctc ctgccccatc | | | | |  |
| 12361 cctgccctct ggaactgggc acactcacca tctgaccagg agctccattt tcaccagggc | | | | |  |
| 12421 tgccaggctc accctgtaga tcagagaata atgagtgaga aattcattca tggtgggact | | | | |  |
| 12481 ctggggatgt ggaggaccat gatgttcaga cagcctctta ccttaggacc agcaggacca | | | | |  |
| 12541 gcatctccct tggcaccatc caaaccactg aaaccctaaa gcaggaaaga ggtagaaggt | | | | |  |
| 12601 aagaacctgt ggagggggtg gaacagcctt gacatccacc tagatctgag aaagagcctt | | | | |  |
| 12661 gggaggtcat caccgccatc cctttgtttc tgagctagac taaaccccaa ctggctctag | | | | |  |
| 12721 atctcaccca atcgtttaat cgggactgct ttcccatgga gggaggtgct tttggatgtc | | | | |  |
| 12781 cactctctgt cccttgggac ttctgtagct gccacccacc atgatgcaac tcagtatctt | | | | |  |
| 12841 ttggggaaga ggttgggact ggatgggggt atgctaggga cttggggagc ttaaatgact | | | | |  |
| 12901 caaaggtgac tcactctgtg tcccttcatt ccagggaggc cagctgttcc gggcaatcct | | | | |  |
| 12961 cgagcaccct ggagagagat gaagaagaca aggaagggcc attagaacac atcactgtgg | | | | |  |
| 13021 acccagctcc taaatgaagc ccaagtgcag tgaagcccag gttcagccac agccccctgc | | | | |  |
| 13081 tcacctgagg cccaggaggc ccacgctcac caggacgacc aggttttcca gcttccccct | | | | |  |
| 13141 gagagggaga gaaaagacca tcatgcctct gcctccccac caccgcctag gggctggaaa | | | | |  |
| 13201 agtggagaag gtctcagtct tctttggatt gttgcaggtc aggtttgggg gcccagaatc | | | | |  |
| 13261 ttatctttga atctagagct cagtttaaat ctacagtgaa accttcgttt ggtgaaatct | | | | |  |
| 13321 gagtgcctct aaaggaattt ctgtctactt ctgtcaccag cattttaatt tcaccatctg | | | | |  |
| 13381 cgcaccaatt tccccaaagg gatggggttg gggattgctc taaggtggct ggggtgagga | | | | |  |
| 13441 gaacagtcac tttgttgggg acatggaatc atctcactaa gcgaaaagat cattgttcca | | | | |  |
| 13501 gggcagtccg tgcatcagag aggacttgcc ctccttcctc tgagtatcgt tcccaaatgt | | | | |  |
| 13561 ggtggagtgg aaattgcagg acccaaccca tggaggccat ggggtcagat ggtatcttct | | | | |  |
| 13621 tgctggggat acttacatca tctccattct ttccaggggg acctggggga cctcggggac | | | | |  |
| 13681 ccatgggacc ctagaaaaga tagaagaggt ggttagaata tggataagaa aaaaagaagg | | | | |  |
| 13741 ggaaggctgg gattgaaggg aagaggtaag gaagacccca ggcctgggag ttcttctata | | | | |  |
| 13801 ggagagtctg tgtgtttgta gaaggagtat gaatctgtat agagagtgct tactgaagct | | | | |  |
| 13861 ccaggctcgc caggctcacc agggggacct tggaagcctt ggggaccctt gagaagaagg | | | | |  |
| 13921 aaaaagatgg gttagaagac aagtccctgt caaccttctc caatcttacc aagagatctc | | | | |  |
| 13981 tgagcatctc tcctgccctc atcccagtct tccctccaaa agaccaaagc ccaaggaggc | | | | |  |
| 14041 atatgaagac gtcctggata ctcacaggtg caccaggggg gccagggaga ccacgaggac | | | | |  |
| 14101 cagagggacc ctatagaggg agaagaaagg ggggtcatgg tgatccctct gtaggaaagc | | | | |  |
| 14161 atgtggatgt agtcattcat gcctgttggg accacccagt cgtcctgcat cctttctgga | | | | |  |
| 14221 ctctgaggtc ccaaaggtga tctggacctc ccaagctgtc tataccagcc gcctatgccc | | | | |  |
| 14281 acctcctctg gatacccatt ctctcttctg tcatccatgc tccccctgct ggctcaccat | | | | |  |
| 14341 ggggccaggc acggaaattc ctccggttga tttctcatca tagccataag acagctgggg | | | | |  |
| 14401 agcaaagttc tagaacaaac aagagaagtc agagtgagga cagtgaattg aaaggcagaa | | | | |  |
| 14461 gacggcactg aggatggaag aaactgacat catgcacttc ctggtgtgat gtgctgagaa | | | | |  |
| 14521 gaataacatt gcttttaaga cattcctgca aagatgcatc cctgcatcta atctaatgag | | | | |  |
| 14581 acaatcccaa attgagggac agtctgcaaa acaactggac tcatcaaaac tcccagtgcc | | | | |  |
| 14641 actaaagaca aagaaagtct gaggaacgtt ctacatttgt ggaaactaga gacatgatga | | | | |  |
| 14701 ttaaacgcaa tacataatcc tggactggat cccagattgg ggtggtgggg ggtggaatct | | | | |  |
| 14761 ataaaggaca ttatcgggac atcggtgaac tttgaatacg gactgcacat tagagagtga | | | | |  |
| 14821 atttaatcat tgtgtaggtc agttaagaga atgctcttgt ttttaggaaa aacacaccta | | | | |  |
| 14881 agtatttagg cgcaaaagag cctgatgtta gcaacttgct caaacagttc agaaaaaaaa | | | | |  |
| 14941 agatacatat acataaacag agactaataa agcacatgtc acaaactgtg aagggtatgt | | | | |  |
| 15001 gagagtttcc tgtaggattc ttgcaacttt tctgtaagtt tgaaattatt tcaaagtata | | | | |  |
| 15061 aaattatgtc atctgccaaa aaacaaaaac aaaacagggg agagtggaca cacaaggcct | | | | |  |
| 15121 ctccacttac tcctccgagg ccagggggtc cgggaggtcc ggggggtccg gggggtccgg | | | | |  |
| 15181 gaagtccagg ctgtccaggg atgccatctc ggccaggggg gcctgcgggt ccctgcaggg | | | | |  |
| 15241 ggagagggcg gggccggggt gagcgtgggg ccgagagcca tgcccacctg cagcccccca | | | | |  |
| 15301 cagcccagag tgcaacgctt acccttgggc ctcgggggcc agtgtctccc ttgggtccct | | | | |  |
| 15361 gtgggattgg gggagaagaa acaagaggcc aggttagaga agggaggact gtgaggagtc | | | | |  |
| 15421 acgggccgcg caggggcaaa attcgagggc aggagattac ctcgacgccg gtggtttctt | | | | |  |
| 15481 ggtcggtggg tgactctagg ggacgaagag acgcgcgtta gagccaaggt ttgctaatgc | | | | |  |
| 15541 tgctcccgtc ggcagaggcc tccagcacgg agggccagcg agcgccgacc acccccagcc | | | | |  |
| 15601 ccagccccag gccccaggcc ccaggcccca ggccccaggc cccgagcgca gccgcacctg | | | | |  |
| 15661 agccgtcggg gcagacggga cagcactcgc cctcggggac ttcggcgccg gggcagttct | | | | |  |
| 15721 tggtctcgtc acagatcacg tcatcgcaca acaccttgcc gttgtcgcag acgcagatcc | | | | |  |
| 15781 ggcagggctc gggtttccac acgtctcggt catggtacct gaggccgttc tgtacgcagg | | | | |  |
| 15841 tgattggtgg gactgggaca ggcggaagag gggcgttgtc agtagtgact gcaaccccca | | | | |  |
| 15901 gcttaaccac ctttcccccg ggtctaagag gcacacttgg atttttcact tcccgcccct | | | | |  |
| 15961 ccccccgttc ttctttcctc aaatccttaa aagctcgcct gctcctcatc agcgcgggtg | | | | |  |
| 16021 acagcgccga ggcgcctggc cagggaggct gtaactcttt ccagttctca ggaatttaaa | | | | |  |
| 16081 caaagcttta gtccgcggtg gcttccaact ccaacctcag cccattggcg ctgaagccaa | | | | |  |
| 16141 gtgaaataaa aaccacaggg tgagaggggg agggcggggg gagaatagga ggggccccat | | | | |  |
| 16201 cgggagggca ggctcgtggg gagtggcttg cgtggtagag acaggaggag ggcgagggag | | | | |  |
| 16261 gagagaaggg aggtccagcc ctcatcccgc ccccattccc tgggcaggtg gggtggcggg | | | | |  |
| 16321 ggcggggcta ggagaatggg agcggctgat tggaagggag gcccccccaa gacttgaaat | | | | |  |
| 16381 gtacctcaaa attaggagac accctaacct ccagcaccta gacatcttaa aagcccgctc | | | | |  |
| 16441 tcggggccat ccccagtccc ccaaagccag ccaatgcctc caggtcactt ttagccaaag | | | | |  |
| 16501 aaccaaaaag tccgcaaata gtccagaagt tagctaacca ctcccaccgc gctagcggct | | | | |  |
| 16561 ccccagacgg ccactaggtg tcgggcaacg ctaagcaatg gggccggggc ggggccgacc | | | | |  |
| 16621 tcaccggccc gactacctct ttaagaggct tgggctgccc catccctggc tttactttcc | | | | |  |
| 16681 agatctagag gtgggggtcc gcacccagca ataacccggg tcttcccgct ccggctcctg | | | | |  |
| 16741 ccccagtaag cgttggaccg ggagacgcag tgctcagcat cggtcagcag ggggcgcaag | | | | |  |
| 16801 gaccccgccc cgccgagtcc gcgccaaagt ttctcatcct ccacccgccc acgctccgca | | | | |  |
| 16861 ccccctccgc ggctgcccag cacccccacg gccccagcaa cactctagcc cgcagagagg | | | | |  |
| 16921 gcaatctttc cttatgaatc atcccacggc cttcctgatt gcttctacat cttgggcttc | | | | |  |
| 16981 aaatgccccg ctcggcgccc ccatcccatc ttcgacgtgc tttcctcact ctgcacccag | | | | |  |
| 17041 atccccactt ccccggttgc gcgctcggga ttctctcttg caccaactca cgccgcgctc | | | | |  |
| 17101 caccctcacc tcccgctccc ggggcgcgtt caaccccaac ctcgccccag ggtgagctcc | | | | |  |
| 17161 ctcctgtctc agggcgccga ggaagagccc tcatcatctc ccttccattc ccgagtctcc | | | | |  |
| 17221 ggatcatcca cgtctcgttt taagccgcgg cccccgggac caagcgcaag gcgcgatata | | | | |  |
| 17281 gagtatcctt gcactcccaa aagtttggga cttactgtct tcgtcttggc cctcgacttg | | | | |  |
| 17341 gccttcctct tggccgtgcg tcaggagggc ggtggccgct aagaggagca ggagccggag | | | | |  |
| 17401 gtccacaaag ctgaacatgt ctagacccta gacatgtaga ctctttgtgg ctggggaggg | | | | |  |
| 17461 ggttagcgtc cgctcatgcg tggcctcaca ctccgcgtgc ctcctgctcc gaccccgagg | | | | |  |
| 17521 agaaactccc gtctgctccg acga |  |  |  |  | |
