# Supplementary material for: Development of a High-Throughput Resequencing Array for the Detection of Pathogenic Mutations in Osteogenesis Imperfecta
Source: PLoS One. 2015 Mar 5;10(3):e0119553. doi: 10.1371/journal.pone.0119553 (PMC4350936; doi:10.1371/journal.pone.0119553)
Supplement: S2 Datasets — Including all the reference sequences of COL1A1, COL1A2, CRTAP, LEPRE1 and FKBP10. (ZIP) [file pone.0119553.s002.zip › OI-col1a2_sequence.doc]

| 1 gtgtcccata gtgtttccaa acttggaaag ggcgggggag ggcgggagga tgcggagggc | | | | | | |  |
| --- | --- | --- | --- | --- | --- | --- | --- |
| 61 ggaggtatgc agacaacgag tcagagtttc cccttgaaag cctcaaaagt gtccacgtcc | | | | | | |  |
| 121 tcaaaaagaa tggaaccaat ttaagaagcc agccccgtgg ccacgtccct tcccccattc | | | | | | |  |
| 181 gctccctcct ctgcgccccc gcaggctcct cccagctgtg gctgcccggg cccccagccc | | | | | | |  |
| 241 cagccctccc attggtggag gcccttttgg aggcacccta gggccaggga aacttttgcc | | | | | | |  |
| 301 gtataaatag ggcagatccg ggctttatta ttttagcacc acggcagcag gaggtttcgg | | | | | | |  |
| 361 ctaagttgga ggtactggcc acgactgcat gcccgcgccc gccaggtgat acctccgccg | | | | | | |  |
| 421 gtgacccagg ggctctgcga cacaaggagt ctgcatgtct aagtgctaga catgctcagc | | | | | | |  |
| 481 tttgtggata cgcggacttt gttgctgctt gcagtaacct tatgcctagc aacatgccaa | | | | | | |  |
| 541 tgtaagtgcc ttcagcttgt ttgggggaga ctgggtagag aggttagatg ggagggcacc | | | | | | |  |
| 601 ctgccctgaa aaggaaaacc tgtaacctga attccaggta cacttggagg gcagactctc | | | | | | |  |
| 661 aggcatgtgg gaaaacgccg gaattgataa gaaacatgga aattacttta aaaaatgaaa | | | | | | |  |
| 721 acataaaagc cttgccaaaa gttagggaac ttttcctcta agttcagagt gagacagtta | | | | | | |  |
| 781 actcggtctg gctcctcagc ttagtaaccc ccaaagggag cggaaggtct ttttccctaa | | | | | | |  |
| 841 ggatgagata ttaacgacca atgtggtgga ggaagtcaag ggcctgcacc ccacaggccc | | | | | | |  |
| 901 cataaccgca ctgatgtcca ccttgtaaaa cttgaggcct gcgttagaaa gcccttcaac | | | | | | |  |
| 961 tgagtaatgt aaaactcacc tcctaagagc ttttatcttc tgggcattgt aaggcttgtc | | | | | | |  |
| 1021 cggaggagga ggatgacgat gctgatatga tgatggttat aaggcgccct ctggaggaag | | | | | | |  |
| 1081 gaaaatgaaa gtacagggga cagggcctta agcagatgga atcccaatta aagcttctac | | | | | | |  |
| 1141 ggatttatac agattaatga tcagcatttc tggttggagc ctttcccagt ggctagtcag | | | | | | |  |
| 1201 tgaaccctgg aaagaagaat ggatgctact tggagtgggt acattctgaa aagtaatata | | | | | | |  |
| 1261 agtgtctcaa ttcactttct agtcatggaa atggtaacat tttttaactc aaatctgctc | | | | | | |  |
| 1321 taaattttgt ttgagcctga gaattacccc tttgacatgt tcccagtgat aagcaaacat | | | | | | |  |
| 1381 tatgaacgca gcaagttgag aaatatcaac attgagatga gactcaagag accggggttt | | | | | | |  |
| 1441 ttcccatgag tctgacacca atttgctgcg tgactttggg caagtcaaac ggccttttct | | | | | | |  |
| 1501 aaaatgtgag acagagatta aagggacccc aaggccactt tccagctcta ggttccatgg | | | | | | |  |
| 1561 ccagactttc atgtcaacag agaatgaaga agatcagtcc gttttcatct tgaaaatggc | | | | | | |  |
| 1621 tgccaaagtg ctagacaaag atattgacta gatgggggat ggtattgtct gaccacaccc | | | | | | |  |
| 1681 agtactccaa aaagttgttc cacccacaca gcacggtgtc taccactgca taatttctaa | | | | | | |  |
| 1741 tgcatttgtg tgcttgtgtg tgtgtgtgtg tgtgtgtgtg tgtctgtgtg tctgtgtgtc | | | | | | |  |
| 1801 tcttccccct tcattcactt ttagtataca tactgtggat actaaggagt aattgcagtg | | | | | | |  |
| 1861 aacaaattca cattaccgag ttcatatttt taatgagatc ttgagagtgg gaggaaagag | | | | | | |  |
| 1921 tcggctccta gagaataaaa tgaaggcaga cttagggaaa tttgaaggta caaaggcaac | | | | | | |  |
| 1981 ttaccttctg atcaacagcc aaccacagtc tggaataaat gttatcaaac acacattctt | | | | | | |  |
| 2041 caaaatggtc catgtctgag taattaaaag gcaaatttcc aaaatcataa ggacttccgt | | | | | | |  |
| 2101 taatcaagtc aggcataatt attcttccta ctgatggaca caatgaagta aacatatcat | | | | | | |  |
| 2161 tcttgtaatt taacagtaat tctcgtaaat tgcccttaaa tgtcagtgct ggatgtggtc | | | | | | |  |
| 2221 caccctccta aattgtgact gttgcaacag atgttctcac ttcaaataac gcacttcttg | | | | | | |  |
| 2281 gccacctaat taaagcaatt tttggggtga ttcatcctac tgcaagcttg gccacacttg | | | | | | |  |
| 2341 tatcctgtat taacctataa tttttgtacc gtaggagaag aattcactct ttaaggactt | | | | | | |  |
| 2401 ataacaatta tggcaaaagg ggggatagta cttttgttta ttttttctat tatttttcaa | | | | | | |  |
| 2461 gatctttaat ccggtttttc catttataca aaactctttc tccgagacaa aaatgataca | | | | | | |  |
| 2521 tattggtaaa atgatcttac ctaatttaag tgaactaatt taaagcaaaa ttcagatgtc | | | | | | |  |
| 2581 tgaattaatc cattttcata gttaataatg tgcaaattag accttttgaa aaaggatatt | | | | | | |  |
| 2641 aagaatggta caaactcaat gaagtactag gtaacttcaa tgttttataa aaaagtaagt | | | | | | |  |
| 2701 cagcttcaat gtttcataaa aaacaaattc aatatagaat tttaaggtaa catactttcc | | | | | | |  |
| 2761 taaattttac cttttttcga tatttaggta ttaaaaatga tcaaaatcat aaattatttc | | | | | | |  |
| 2821 ctcatcaatc tactagtctt acattcagcg attcatctgt gcactttacc agcttaattg | | | | | | |  |
| 2881 ctaagcattc aaaatatcct tcagacacat taatatttca caacagttat aaaatagtaa | | | | | | |  |
| 2941 ataattaata atttaattca aaatacattt acatattaat attgcaaaca aatcaccctg | | | | | | |  |
| 3001 ctgatccctg ccatactttt gacctgcata atttctaggt cattaaaata ttcttaaaaa | | | | | | |  |
| 3061 aatataattg gtccttaatt aggtaattca attctataaa cttgtttctc tatttgttaa | | | | | | |  |
| 3121 ttattgctat tgatccatga agtgatacta ataattgttt cctacttttt cttttttttt | | | | | | |  |
| 3181 tctacagctt tacaagaggt gagtaaaact ttttttagaa tttttaaaaa tactttgatt | | | | | | |  |
| 3241 cccttggcta cagtgatgtc ttctcttgga agggaagaag ttacattaat attgaccatc | | | | | | |  |
| 3301 ctagattccc aagaaaattg tgaagaatta tgatagtcaa aacctttctg gctgccttag | | | | | | |  |
| 3361 aaagtaccca cccaattttc caaaataggc ggggctactg aataagacta ggtttataaa | | | | | | |  |
| 3421 atattcataa gaaatataga gtaaataatc caatagaagt ttgagtttta ggatcagctt | | | | | | |  |
| 3481 ctatgaagca gaagatttca ctgagctaga gaatcttttc actcctttgg aattatttgc | | | | | | |  |
| 3541 aaaagcactt attgttaaca cattcttagc tcatgagttg aatttgaggc ataagtacag | | | | | | |  |
| 3601 gtacgtattg ctatgtattt tttgttctgt aggtacatat ttttatttga catgttggta | | | | | | |  |
| 3661 aaattttaaa ttgtagtttg aaatattaaa ctgagataat agtaaatgca taatgtaatg | | | | | | |  |
| 3721 aattgtgaag gtatatttgt atactacacc aaaatggaag ctgtttttaa atatatatat | | | | | | |  |
| 3781 acaattttct tcataataat ctttgattta ttcttttcta ggaaactgta agaaaggtaa | | | | | | |  |
| 3841 gagtacacta cttctccata aatatctaaa attatcaggg ataacataat ttaactaaat | | | | | | |  |
| 3901 ttatagtaga ctatagaagg aaaatacttt atcaaaattt tgttcatatg aatatacatt | | | | | | |  |
| 3961 agctaaagca taaaataaag tagctttgat gtttaagata acaaagttta attatcttct | | | | | | |  |
| 4021 ggaatcatct gtaattacat ttatgtgata caaactggtg atttacatac aaaaggaaaa | | | | | | |  |
| 4081 aaaaagactt gtttttattc tggagatgga aggcatatta tgttaattat agggagtaaa | | | | | | |  |
| 4141 aaaagtttat tttaaagggc tttgactata taaatgtgct gttaaaaatg taacaaaatg | | | | | | |  |
| 4201 atcatttaat ctacagttat catcttattc aaaatgctat gcatagtatt gacctaatag | | | | | | |  |
| 4261 ctgaagacta tagcagcttc caatcctcca gctgaaaaaa aattacgtat aattacaatt | | | | | | |  |
| 4321 aaaatatata cttttatcta ttgcattgtg tcaatttttt atatgctatc taataacatt | | | | | | |  |
| 4381 gtagttacat cagtcttacc aactaattat tatcaagaat gatttgtttg ttcactggaa | | | | | | |  |
| 4441 attacttctt aggcatttat tattgtcctg tttgtatctt tcctgtaggg cccagccgga | | | | | | |  |
| 4501 gatagaggac cacgtggaga aagggtgtgt aatttttgaa ctataaaggg cttcgtcccg | | | | | | |  |
| 4561 tatttgaata actatatgtt agaaactaca ggaactggca atttataaga atattatgta | | | | | | |  |
| 4621 tccagataat tgtacacccc tttaaacagg taatgcactg cagaagaagc gaatgagcat | | | | | | |  |
| 4681 tattatatat gatcaatatt tgttttaggt caaaattacc gttaaaaaag aaaaactgtt | | | | | | |  |
| 4741 acagtcatat tctttgcatg gtctactttc tttatttgta attgacccat ccaacacatg | | | | | | |  |
| 4801 cataatggaa atatatctac ctaccaccac agtcctcttt ttaacacatt tcatttgctt | | | | | | |  |
| 4861 ttgaactaag atcccttagg tagcttggaa ataatagtga attagtagtc agtaacatgt | | | | | | |  |
| 4921 ttctctgctc aaattcatgc atgtacaagt caggcttaca ttttatttgt ggcattctta | | | | | | |  |
| 4981 aatctccctg ctatgcttat ttgacattta taactatgtg gttttgcatt gtataacact | | | | | | |  |
| 5041 tttgccaata tatgaatacc tatatcttat atctattagg aagaggagac ttacatgtat | | | | | | |  |
| 5101 ttcactcaat ttattagaaa tagaattaaa tcagttaatt attttaacaa tacaagtagt | | | | | | |  |
| 5161 taatgatagt aaatctgcag gattttctct cctatgataa agtgacctta ttaactgtca | | | | | | |  |
| 5221 catcagttaa ttcattcaca tgtaacatac caaaacaatt gaatcagttt gtcacagtca | | | | | | |  |
| 5281 gagatcggca ataaaaatac gatgtaagtc cttgtgcact gttaaacata tgaagcacgt | | | | | | |  |
| 5341 ggaaccatac attttggcta taatttttat atttgaatac tggagcttca gtatgaatta | | | | | | |  |
| 5401 atattcaatg gccgagatag ttctttagga aaactaccct gtgatatctt aagagttatt | | | | | | |  |
| 5461 aaccctcttt ctaaaataga ctcataagtg aatttcaatc aatgacaaat atagtatatt | | | | | | |  |
| 5521 aaatttccac cctacttgca catagaaagg tctgaacaac tgatcttacc acatataatt | | | | | | |  |
| 5581 cttaggtttc tacagggcct gtctaacctg accttactca ctttttacat aacagggtcc | | | | | | |  |
| 5641 accaggcccc ccaggcagag atggtgaaga tggtcccaca ggccctcctg gtccacctgg | | | | | | |  |
| 5701 tcctcctggc ccccctggtc tcggtggggt aaggtgtctt acgtattgct aacttttagc | | | | | | |  |
| 5761 taacttcagt tgaaagaagg tttattgtgg aatttatttt tagcagttaa gggataattc | | | | | | |  |
| 5821 ttccatttga aaattagtat attttatttc atttatttgg ttttttcact caagattctg | | | | | | |  |
| 5881 ctttacccat tctctttgtg agcccttgtc aattacagga ctggtctttg tgtgcactga | | | | | | |  |
| 5941 agttagctgt ggccatcatt accattattt aatttggaga tttaatatct tttattagta | | | | | | |  |
| 6001 aggcacaaat aagaggtgtt gcattattaa ggattttgat tagattgaac tgtgtaagtg | | | | | | |  |
| 6061 aaatccctga tcttaagcaa ttttacaaac atcctacgct ttttattctc cttgacttga | | | | | | |  |
| 6121 agtctgctga accaacattc aaagcggttt taggtttaat ttgcttgaaa ctaatttgag | | | | | | |  |
| 6181 aaaagtacat ttcccttttt cattaatatc ttcttttagc ttcatgtctt taacaatggt | | | | | | |  |
| 6241 atgagtgcca aatgacctca ctgcaggaag gaaaacatat ttgcttaatt ggttagcact | | | | | | |  |
| 6301 atgaatcaga agcctgattc taatacccaa ctgtatgtca gtaaaataag acctttctcc | | | | | | |  |
| 6361 cccaaagatc ttattatgat tgcttatcta tgaattgcat taaaaagcag cttctttaat | | | | | | |  |
| 6421 agagctacca ctataagaga gatctttaac agtaaagtta ttactgtgaa ctagttttta | | | | | | |  |
| 6481 gaagttttat cttccaaggg gtattttaat ttaattttcc tctaaacttg aaaactcttt | | | | | | |  |
| 6541 atgcccttcc tgaaactcca gcaagaaaaa gatctcttag tcattttgtg tagctccggt | | | | | | |  |
| 6601 ggggaagggc aacaggtgaa aatgtgaaga tgtcctcttg agctctgtct aatttgtcag | | | | | | |  |
| 6661 gagcccttag taacattaaa agtttagaaa gcttcccttc ctcagagtag aggtaaaagg | | | | | | |  |
| 6721 tgggagtgga gacaccgagt taaggcagag gaagggctca aaaagtaaag tagggaagtt | | | | | | |  |
| 6781 ctccatttca aagaggtgtc ggccaagttt ttgacgtaca gctctcataa ctttttagga | | | | | | |  |
| 6841 atttagttca atatagaatt ttaaactaat aattatatca aaaacattgc cctcttttaa | | | | | | |  |
| 6901 ataacaacag aaaaatattt acaagtagaa tgagaaaatg aactacatga ctagtaacta | | | | | | |  |
| 6961 aaaatatttt atatatatat ataatttttt ttttttactt ctctagaact ttgctgctca | | | | | | |  |
| 7021 gtatgatgga aaaggagttg gacttggccc tggaccaatg gtatgcttat ctgtttatct | | | | | | |  |
| 7081 tagccaaaaa aattgctaaa taaatcattc attttatgtc acattttacc acgccattta | | | | | | |  |
| 7141 tttagctacc taagttaaca ctcaatactt agattatata aaaacaactc tttttgtttt | | | | | | |  |
| 7201 caaatttatg aaaacataag ttaaggagtt cacttttctt tacaaaagaa agattaattg | | | | | | |  |
| 7261 atcttttatg attatatgat ctttttgatt atatgatcct cattaagata gatcatatac | | | | | | |  |
| 7321 ttatgtccaa gaaataatct ttggacatag taaccataac ttgggcaaat caatttaatt | | | | | | |  |
| 7381 taaaacagta atcactctga ttaatttttt aatattcttt aacattgctt agaattttaa | | | | | | |  |
| 7441 gcaacactta gaggcataga actatttatt aagttctctg aacttgttgg aaaggatcaa | | | | | | |  |
| 7501 caagttctat ctagtccagc taactcattt taaaatggga gagtttaagc ccttttctca | | | | | | |  |
| 7561 aagtcatcca ggtaactaat gacataacta gaactagatg ccaggcaaga tgtctaatat | | | | | | |  |
| 7621 ttgcttacat catggtttat gtacctagtc cttgaataaa ccactcattt agtcaacaga | | | | | | |  |
| 7681 tattaatcag atgccttcaa tgggccctaa actgtattag gaactgggga aattacaagg | | | | | | |  |
| 7741 aatatgacag attctgatcc ttcctcaagg agttaacaat ataggaaatg tttctttttc | | | | | | |  |
| 7801 tgaattttga ccaaaaaaat ccttttttag tctattgatt gtaaatctat atagaagaga | | | | | | |  |
| 7861 gtatgagtaa aaatctagca tttatgtcac tcagtacaaa tattcagcac cataccctat | | | | | | |  |
| 7921 cagtggagca ccgcttagaa acattcccta tatgatgatg atgatgatga tgactattaa | | | | | | |  |
| 7981 caaatgaagc ttctaacaag cattagagag aagtttgaag ggaaaaatgc taatacgagc | | | | | | |  |
| 8041 atgcaaaatg tatactagca tatatgaaat agaggggaaa actgccagaa gtcaaagtgt | | | | | | |  |
| 8101 taggttgatt aagcactaca gaatttaatg tatacacaca cacgcaattt agtgatttta | | | | | | |  |
| 8161 attaattgtt tcaaaacaaa ggtatttatc tgcccaaagt caacaaggtc tttaaaatgt | | | | | | |  |
| 8221 aaattttacc tgggcagtgc acttagtgct ctatcttcaa aagaagatgt tctgctggag | | | | | | |  |
| 8281 ctaatggccc acagtaagct aatatactct aagggtgaga taatattttc tgtaaattaa | | | | | | |  |
| 8341 aactcccact tgagaaataa tgtaccttta attgacgact tctaattccc taattttttc | | | | | | |  |
| 8401 tggtagttta aaatgttcat atctgaaatg aaaaagtaga gtgtttcttt tggctttgtt | | | | | | |  |
| 8461 tatattggat ttttgaaatt agctgtttca gctaatgctg gacattagtc agttttaaag | | | | | | |  |
| 8521 cagtacctac atctcaagaa gaagcaaggg ggcggaaagt aaagagctac taaatgtcat | | | | | | |  |
| 8581 ttttaaaaag cccactaagc tgggaaaatt aatatggatt tcagataccc ctgttttcgg | | | | | | |  |
| 8641 aacatctgtc ttggcataaa gcagagtatt ttactttgaa atatcagtga aatataattt | | | | | | |  |
| 8701 aagcttgcac atccacacac atgcacagac atatgtaatc aacagatatc tgtttcacaa | | | | | | |  |
| 8761 atagggaaga taggcagcaa taaagtatta aaataatttc catgttggaa aatcaataac | | | | | | |  |
| 8821 tataaaaccc cacagggttc ttctctgaat taatgagtaa tcacagcctc catgaaatac | | | | | | |  |
| 8881 actacatttt atgtaaatga aattgttgca aatacatgaa aaaataaata taattagaaa | | | | | | |  |
| 8941 ttcatgatgt caaagaaaat tattttttaa tgtatgccta aaaagctatt gtgatggaaa | | | | | | |  |
| 9001 agtgacagtt tcttttaatg tcagagcaat ttctaaaacc aaatgaataa ttcttataat | | | | | | |  |
| 9061 taaaatgaca tacattttag ataaaatcca tgttatttca ctctaggcat taatacagta | | | | | | |  |
| 9121 aggtaggttt gactgcagag tccccacagc tgatgtcatg aacaaattac ttgagactgg | | | | | | |  |
| 9181 tacatgaaat attttcagca ttatgaggaa cagaccctac ggatgagctt acacaggcac | | | | | | |  |
| 9241 tgattactgc aaagaggagt caagaaagtg tatttagctt acaaactatt aacagcctgt | | | | | | |  |
| 9301 tttaccctac ttttgtgcta tggaaacaac aaaggggaaa acaatcttcc atcatttggg | | | | | | |  |
| 9361 ccatattttc aacaataata tcatataata gactcttcca gaaggctgtt tcaataatgt | | | | | | |  |
| 9421 tttatttttc cttcacccct cattacatcc acttttgttt gacattttca tcagtcacca | | | | | | |  |
| 9481 ataaccctta gaggagcgat aaggttataa caaacttctc tctaatcatt aagaaggact | | | | | | |  |
| 9541 tttgattctt ttcaatttat gtcctttgtg gcaataaaaa taccaatttc ttagctaaat | | | | | | |  |
| 9601 atgacatagg aagatgacat atgatcaaag atatccaaat ggacatgctt catctgctgt | | | | | | |  |
| 9661 atagaagaca attgtatatt ctgcacttct gcaaagactg attcacttca ttgcatcaga | | | | | | |  |
| 9721 acaatctcaa tatgcccaat tgtgcacaac tttaaggaac ctatctgccc cgtctaattc | | | | | | |  |
| 9781 tcattgattt ctgttgataa ggattgggag aaaaggaaaa gcaaagggag agaactagtg | | | | | | |  |
| 9841 caggaagttt gagtccttaa attcttcctt gggaggaata aaaactatgg aatcaaacca | | | | | | |  |
| 9901 caacaatggc actgctaagt tggtcatatc tgaccccagc caacaccatg acaacttatc | | | | | | |  |
| 9961 agtgctaact gttgatatat ctgctttctt tacagggctt aatgggacct agaggcccac | | | | | | |  |
| 10021 ctggtgcagc tggagcccca gtaagtactg aaagcttgta atgcctctta tgtaaaaaga | | | | | | |  |
| 10081 cagagaatta agaaataaag gcttggagta tgacattctt tttttctttt agggccctca | | | | | | |  |
| 10141 aggtttccaa ggacctgctg gtgagcctgg tgaacctggt caaactgtga gtacattttt | | | | | | |  |
| 10201 ccacctttgt gataagtttt tttccaggaa gtttatgaat ataaccttag tgaaatgatg | | | | | | |  |
| 10261 ggtctcccat tttcttaggg tcctgcaggt gctcgtggtc cagctggccc tcctggcaag | | | | | | |  |
| 10321 gctggtgaag atgtaagtat ttactcttaa gcactttcaa aatgctattt aaatactctt | | | | | | |  |
| 10381 gcctcaacaa gattttctag attcaaatta agtattctgc caaaagctga atatgcctga | | | | | | |  |
| 10441 cagaactctt aatgtatggg aaatattatt ttaatgaaat attaactaac ctacttgtat | | | | | | |  |
| 10501 taagggaaag attaaatata tatctggatc catattttta tgtgataact ttctcccctt | | | | | | |  |
| 10561 ttgtaaaaac caagattccc ccattttgtc tgatagttta ccaagaagaa gttgactcta | | | | | | |  |
| 10621 caatgttttc atgtttaggg tcaccctgga aaacccggac gacctggtga gagaggagtt | | | | | | |  |
| 10681 gttggaccac aggtgagact ttttacattg gtagatagca caaacatcat aggcctataa | | | | | | |  |
| 10741 gatagttgct aaaactagca tcaatctaaa tgacaacata gatgtcaccc aaactcataa | | | | | | |  |
| 10801 catgaatcga aggcatctaa taaagaaaaa agcctagtta aaaaaaaatg catatacatt | | | | | | |  |
| 10861 ttattcatgc aaataatgga atataaatga cagcaagcat accataagca actaaattgt | | | | | | |  |
| 10921 gttttctaca aataccgtat tattagttac tcacattaga gcaagttaat ttgtcactct | | | | | | |  |
| 10981 gtgcttagag gtatactaga ctttggttca aagcttgaac tttgatgaga ataaatactt | | | | | | |  |
| 11041 tggagggaag aagtcactgt ctttttattt atggtaaaac attattcacc atcttctgta | | | | | | |  |
| 11101 tttctttcta agggtgctcg tggtttccct ggaactcctg gacttcctgg cttcaaaggc | | | | | | |  |
| 11161 attagggtga gcacattctt tactcagaag agagaaaatg cctattaatt tttggaaaaa | | | | | | |  |
| 11221 actcaagtat gtttaaaatc ttgggtgaca tatactcact ttcaaatccc tggagtttgc | | | | | | |  |
| 11281 caaagggaag aaagagttaa agagtcagat ttcttgaaag taaagtgggg tgcaattttt | | | | | | |  |
| 11341 tcagtctgtt catagctacc aaaaaacagg ctcactacag agaaaattat atagaacatg | | | | | | |  |
| 11401 tattacttat tgagtattta caaccgtctg aaaatcataa aattattaag gatggaaaag | | | | | | |  |
| 11461 atgtgagaga acacctagtc ctccatcctt ctctctcaat ggcaagaaag ttaagtgacc | | | | | | |  |
| 11521 tatctagggc aatagactga gtttgctggg acctggaaca ctggacttct ttctactgca | | | | | | |  |
| 11581 gcagacaaga cttacccaag agagattaat ggcaaagata tacaatacaa tttttatttg | | | | | | |  |
| 11641 accaaacact atcatggaac agcattttat aataaggctt tcctttcagg gacacaatgg | | | | | | |  |
| 11701 tctggatgga ttgaagggac agcccggtgc tcctggtgtg aaggtaaata ttaaattaga | | | | | | |  |
| 11761 agcactgttt ttaagcactt gattgaaatt ccccatgacc tccaaaaaag tatattatac | | | | | | |  |
| 11821 tgaagactac ccatattaca aaaagtattt ttattttttt tctttcctgt acttcaaatc | | | | | | |  |
| 11881 cctcaaggat ggggactatg agagtctgtg aaaaaaggtc aattattaat atttattaaa | | | | | | |  |
| 11941 attcaatatc tattaaacaa ttgagataaa aataatatta atagtttctt gttccatttc | | | | | | |  |
| 12001 ctttcctccc tctataattc cagtgtatct ctgcagccaa aataaaagta aataaacata | | | | | | |  |
| 12061 taatcagaga ttacgacact ctgtattatt ttaaactgta aattctcctt tgccacacac | | | | | | |  |
| 12121 taattagata ggtacattca tgtcgctata cacttttcaa cctctttcct gtgatttatc | | | | | | |  |
| 12181 tgtgcacact caaaaaaatt ttaattaggt aattaaagtc tcagaagtgt gttatctctt | | | | | | |  |
| 12241 ggctaggctc ttctctgaca gcgttttcaa ctataaaatg ttctctttcc tattaaggag | | | | | | |  |
| 12301 ataatgtgat attaaagtga ataccaacgt aattacaaat taatgagtaa cgaatactag | | | | | | |  |
| 12361 cgggaccaga aatgaacatg aatatggaga atctattcta actttccagc tgccacacaa | | | | | | |  |
| 12421 atggataagg tcaaactcat tctcccaaga gcccgatata acagctcaga ctactaatca | | | | | | |  |
| 12481 ctgtatcata aaatgttaga gctgcaagga gctttggaga ccccctcatt ttgcagaggt | | | | | | |  |
| 12541 gggaaactga ggcttcgtga gagcaattga cttgcccaaa gtcacacatc tagaggttag | | | | | | |  |
| 12601 aaagtcatag gctagaaatg atcccccctt gccacttcaa tgcttattcc caaagaatag | | | | | | |  |
| 12661 acttcacata gaatcctgga aattaagggt ccttatgagg tctcttaaac catatttccc | | | | | | |  |
| 12721 ctatatctaa atcagattat ctttaaaaaa agttctttta catgtgccat agtattaaat | | | | | | |  |
| 12781 cccactacta ctactactac taccctggtt tttactcagg ataagaatat agattggaaa | | | | | | |  |
| 12841 taaatatgat ggctctaaaa aataccatga agcttcaatt tttcatgcac attttatgaa | | | | | | |  |
| 12901 agtgataaca ctgagtgttc aaaataactt taaaaaggat aaatatggtt acattgaaag | | | | | | |  |
| 12961 caaatttatc ctttgccatc tctttttatg atattgtttc tagtatataa ttgatatcct | | | | | | |  |
| 13021 gaatctaagg gagaaattgg ggaggaggta cactcaaata accacatctc cttagaacct | | | | | | |  |
| 13081 ggatatgtgg tactatctga ataaaaactc atgttagcac attttaaaat ctgtgtgtct | | | | | | |  |
| 13141 ggcataattg aaaaacaatc tatatgtgta agaaatatta tgaagtatat gaatggttca | | | | | | |  |
| 13201 aagtaaaaaa aatagagtaa aattgcacta tcaggaaaaa taattgttat atttaatgaa | | | | | | |  |
| 13261 caaaaactca atccttctcc atgtagggtg aacctggtgc ccctggtgaa aatggaactc | | | | | | |  |
| 13321 caggtcaaac agtaagtatt gactacttca ttgtaaattt aaatgtgtac actctttatg | | | | | | |  |
| 13381 agatggaact tctttaatgt ttttgctaat cactgtatcc ttcagcattg tattctttga | | | | | | |  |
| 13441 tgtttttcta atagccttct gatacttaat tgaaatccac tactgtttag ttggaattag | | | | | | |  |
| 13501 aaggcaactt atttattttt agtgtattct tgtacaggtt ggaaactgaa caaagcaaat | | | | | | |  |
| 13561 gatgcctgtg acttttttta aattagcatt ctggatttta ttgaaaatat ttctgcttct | | | | | | |  |
| 13621 agggagcccg tgggcttcct ggtgagagag gacgtgttgg tgcccctggc ccagctgtaa | | | | | | |  |
| 13681 gtgcttccat ttttgttcag tttcatcctt ttaaaaaatc ttctaatggc tgtcatttaa | | | | | | |  |
| 13741 gtttccacct gatcttccct ttattttctt cttagggtgc ccgtggcagt gatggaagtg | | | | | | |  |
| 13801 tgggtcccgt gggtcctgct gtaagttttg acactgggga gtttgaaagg agttgagaat | | | | | | |  |
| 13861 gtggggtggg tgctgtcttc ttcattaatc tcttacgaaa tagcatcatt tcagacactt | | | | | | |  |
| 13921 taccaaatgt tctgtgaggt cttttgaagg ctccatttat aagtagtgta agccatttat | | | | | | |  |
| 13981 aagtacctga acttttgatt gatgtataaa gcaaaatatc cccaccctgg ataccatgaa | | | | | | |  |
| 14041 tgtcttgcct ttgatgagat cctaacgaca acagactggt tgtcagtttt tttctttact | | | | | | |  |
| 14101 aatataaaca gtgtcatgcc actgtaagca acttcaatct tctgccattg ttattgtttt | | | | | | |  |
| 14161 cttaatttac ttggaggaaa tttcttacca ccttctgctt tgatttcagg gtcccattgg | | | | | | |  |
| 14221 gtctgctggc cctccaggct tcccaggtgc ccctggcccc aaggtaaaaa cactggtgac | | | | | | |  |
| 14281 cattgtcact actttgataa actttttact gtgatgtgaa agattggaac tgtgtttgca | | | | | | |  |
| 14341 gataaagaga taattacgaa acagttacct taattattcc ttcccttcaa aatggacata | | | | | | |  |
| 14401 gaatgaccag ttttctcact ctacatttga aatagatcat ttctctgcac tgtgcactgt | | | | | | |  |
| 14461 gcccatcgat atagatgaca acatggaaat tgtctctagg actagttagt taggactgac | | | | | | |  |
| 14521 tgagaaccag agtcaaccac agagagacag aaggagaggg aaggtagtaa cagtagccaa | | | | | | |  |
| 14581 gatggcagaa tcaagcaagg aaaataggaa accaaactca aatcttgtaa taaaacggat | | | | | | |  |
| 14641 aagaaaaata attgcaattt tgaagtttta tgaagacatt tcataaaact tggcatctta | | | | | | |  |
| 14701 aaaacagata tgctgtttca ttatttgctg gttaattcct tggtttaatt tcctctttta | | | | | | |  |
| 14761 gggtgaaatt ggagctgttg gtaacgctgg tcctgctggt cccgccggtc cccgtggtga | | | | | | |  |
| 14821 agtgggtctt ccaggcctct ccggccccgt tggacctcct gtaagtagcc actgtcttta | | | | | | |  |
| 14881 aactttattg agtaaaagaa aacaaaggtg gagtatgggg aagaagaaga atgaagatgg | | | | | | |  |
| 14941 ggtcaaagaa gaaccgaaat attccaatta actgatatcc ttctcctttc cttttcctca | | | | | | |  |
| 15001 tagggtaatc ctggagcaaa cggccttact ggtgccaagg gtgctgctgt gagtatacct | | | | | | |  |
| 15061 gcgtagctaa aatgtgctgc tatgatttta aaggcattta atgtgtgctg cctctacagc | | | | | | |  |
| 15121 ccatcacctc cctaatggac cacactgcat tttccttcac agggccttcc cggcgttgct | | | | | | |  |
| 15181 ggggctcccg gcctccctgg accccgcggt attcctggcc ctgttggtgc tgccggtgct | | | | | | |  |
| 15241 actggtgcca gaggacttgt tgtaagtggt catgactgtg gttctcatca tcctgaaata | | | | | | |  |
| 15301 ccacctctgc catcatttca tcactatcta gacttccact tgtagtttta ttattcctat | | | | | | |  |
| 15361 ttttctcttc cttagcattt ttagtttata tttcttatat atatatgtac actcccgtct | | | | | | |  |
| 15421 gctatatgca cacagacatg cccttcctgt tatcttaaat cattacctca aggtaaatga | | | | | | |  |
| 15481 ggcaaagttc tacaatatca gttttgtccc ttcgaccaat aataccattc ccctgtactc | | | | | | |  |
| 15541 aatttaaata tgaacagggt acatttccta gagaacttga gcttctcttt accttgaccc | | | | | | |  |
| 15601 acaaatattc taagagattt gtctgcaaga gagtttcaac aaatgtttgt cctttgacca | | | | | | |  |
| 15661 ctgttctgta ttgaacccta gggtgagcct ggtccagctg gctccaaagg agagagcggt | | | | | | |  |
| 15721 aacaagggtg agcccgtaag tagctctatc atcacacttt tataaagtta attgtttttc | | | | | | |  |
| 15781 tcattccagt ttctccagct ggacatagta ttaaaattat ttttttactc cctcttcttt | | | | | | |  |
| 15841 tgttcttttc attaaacagg gctctgctgg gccccaaggt cctcctggtc ccagtggtga | | | | | | |  |
| 15901 agaaggaaag agaggcccta atggggaagc tggatctgcc ggccctccag gacctcctgg | | | | | | |  |
| 15961 gctgagagta ggtttcaaat gctcccaaca ccctaacaca ccagaggcag attatgatac | | | | | | |  |
| 16021 cccttcactg ggaattggtc aaaattactg actgtgtttt cttaggcaaa aaaagcatct | | | | | | |  |
| 16081 gctttccatc tgccttatta aatcagtgac tctcaattta atatgttata aaattggcct | | | | | | |  |
| 16141 ggaaacaatg ttgacctact tttgcaggat gctcatctat gaattcctct aggggttggg | | | | | | |  |
| 16201 tgaagtgttt tggcttggtt tgtgtctgta tctcccctgt aaggagatca tgctattttt | | | | | | |  |
| 16261 tacaaactct accttatcaa agccaagaga tttctttaat tctctctatt tcatgtactt | | | | | | |  |
| 16321 tcttgcaggg tagtcctggt tctcgtggtc ttcctggagc tgatggcaga gctggcgtca | | | | | | |  |
| 16381 tggtaagctg tctatcactt acttcctaga aaggggcttg ctgcttctgg tggtgggtgt | | | | | | |  |
| 16441 gtcattagct ttagcatcct cctcctctat ctgttttttt tttttttttg aatagggccc | | | | | | |  |
| 16501 tcctggtagt cgtggtgcaa gtggccctgc tggagtccga ggacctaatg gagatgctgg | | | | | | |  |
| 16561 tcgccctggg gagcctggtc tcatgggacc cagagtaagt ttcaaactga ttctgagcaa | | | | | | |  |
| 16621 atcacacctg gcattacttc cttctttaaa gggttggtta atattgaaga taacaataaa | | | | | | |  |
| 16681 aacatcaaaa gtaaatttgt tagtagtctt gctgacagtt gcatttttga ctttatcaaa | | | | | | |  |
| 16741 gctcagtaga tattttcatg catttaatta gttcataaat tttctattta ttacttgata | | | | | | |  |
| 16801 caatggctat gaggtttttg gaagaataga tctattttaa tatatccaaa ttagattggt | | | | | | |  |
| 16861 cctcctatca gcatgaatct tttatcttaa tttgtgagtt ttatataagg tgttcatgaa | | | | | | |  |
| 16921 atatattagg actatacatt tttggtttat tagattcata agtgaagtct ttttcctagc | | | | | | |  |
| 16981 aatcacaaag tgctgtaatg tattcagcat cacactagct atggagaaat aacctctagg | | | | | | |  |
| 17041 tccatagaca cactaatcca tagcaataga gtaatttttt tgcctccatt gcctcttatg | | | | | | |  |
| 17101 ggtgaatatc aactgtaatt gtaccataaa caagtaatag ggacaccaaa tatagcaata | | | | | | |  |
| 17161 agaaatccac tttggaaatt gtttactaaa agtattagtt tttctattat gaggtaaata | | | | | | |  |
| 17221 acgtgataca ttttgcccat atacatgttg cttaacagtt tcttgagata tctataaaag | | | | | | |  |
| 17281 gatgagttgc actaaatttc aataaaagga aagccacaaa aaaatagaag aaaaatttca | | | | | | |  |
| 17341 gaactctttt cacacttccc agctagtggc taatattcct aatgatttac cctaggcaac | | | | | | |  |
| 17401 aaacaaaaag tcgggggaaa aggtgccttt gttagacttc agttaatcta aggcttgagt | | | | | | |  |
| 17461 atgtaagtta aagtgccaat ataaaaacat cctcatttat tttatagggt cttcctggtt | | | | | | |  |
| 17521 cccctggaaa tatcggcccc gctggaaaag aaggtcctgt cgtaagtatt gctcattttc | | | | | | |  |
| 17581 cattatattt tcaaggacac ttattgcacc cttatcaagt ctattttgtg gcttatttat | | | | | | |  |
| 17641 acatgaacac attgaaaata aatatcagac acatacatca tctgggaatg cagagtaata | | | | | | |  |
| 17701 gattgtaatt atggagtcca aatgaataca ggactgaaag cagagcaggg gagagaaaaa | | | | | | |  |
| 17761 catggcaggg aaaattgaag caggtgacaa ggggatgcaa gagaagggaa tgagggaaat | | | | | | |  |
| 17821 tgcatacata cgagattgaa ttggctatgt gtgtactgac atcctagtta gaaaaggaaa | | | | | | |  |
| 17881 atggattcat aatttattaa cgctttatac aagaagctct atgcattcag aaaactattc | | | | | | |  |
| 17941 tgtttcatcc gtggcagcat cataagcttg aggttgtgag aatatgttga cactgagtaa | | | | | | |  |
| 18001 acttgaaata actctgcttt cagggcctcc ctggcatcga cggcaggcct ggcccaattg | | | | | | |  |
| 18061 gcccagctgg agcaagagga gagcctggca acattggatt ccctggaccc aaaggcccca | | | | | | |  |
| 18121 ctgtaagaat caccacaact ttcttaccct cagcactttc tgtagccaaa ttttaccaaa | | | | | | |  |
| 18181 ctctagtatt tatctcctgc gaatcagtcc agtctcaggg agtttccttt caacacagga | | | | | | |  |
| 18241 aaactgcagg ccacttatca cattaaaagt ttacctctag tgtatcctta tatccctgct | | | | | | |  |
| 18301 aaaaatccat ctcctgagcc ccatgcttcc acagacacag ggacatctta ctgtacatgg | | | | | | |  |
| 18361 agctgcatgg tgatggatca tccttagata acagaaacca cagactaggg atctcaaaag | | | | | | |  |
| 18421 aacacaaaaa caagcaggat tcaacattgc aaaatcaccg tggttaattt gacattaaat | | | | | | |  |
| 18481 gtgcaaagct gttctttgtt ttgtttttca tttttactct agggtgatcc tggcaaaaac | | | | | | |  |
| 18541 ggtgataaag gtcatgctgg tcttgctggt gctcgggtag gtgctaactt gtgtacagat | | | | | | |  |
| 18601 ctattcacat agcattcatc taagaaccac actttttttt ttacaccatc tgatatcatt | | | | | | |  |
| 18661 ttgtcacttt cttttcaaga tggcatcccc aggggtcctt ttactatcat aaaatgcctt | | | | | | |  |
| 18721 tttaaaaacc aaacttataa aacagtgagc aaaaacaaat cagaatatac attaggtcaa | | | | | | |  |
| 18781 aaatacagaa gcacttggct tttattttat tcattttgta attaaaaggg tatgaatatg | | | | | | |  |
| 18841 tagtagcatt ctctggcctt tataaattgc cttgtgtcgc atacttcgct tgagtcatat | | | | | | |  |
| 18901 caaaagttag taggcaaacc cataaatata tatacctact atgtacccac acaaattaaa | | | | | | |  |
| 18961 aatttaaaaa gttagtaggc agtatttggg ctttcgtggg aacccacaat gagtttaatt | | | | | | |  |
| 19021 catgctaaaa tgacaaactt gttttaagga agtaatacct gaggctttga gacatcttaa | | | | | | |  |
| 19081 actacctggc ttgcagctaa ccatcagcct ttctgttaaa tatttttagg gtgctccagg | | | | | | |  |
| 19141 tcctgatgga aacaatggtg ctcagggacc tcctggacca caggtgagta tttctcccac | | | | | | |  |
| 19201 tcttgtgctc ttctgcacta gaatgtatat agtcctcaaa ctggccatct ccattttcag | | | | | | |  |
| 19261 tccaaaagtt atacagctag acaacagtgg tgacatacgt tgctatttat gctctctttc | | | | | | |  |
| 19321 ctgtcacttt cagggtgttc aaggtggaaa aggtgaacag ggtccccctg gtcctccagg | | | | | | |  |
| 19381 cttccaggta agtcaactca aacatataca atactgcctt tggtcagcct attgagctgt | | | | | | |  |
| 19441 aaatcaccat accgtacctc tcttctccac cacaataaac atgatttcag gactgaagca | | | | | | |  |
| 19501 aagaaaggtg catttttttc aaacaaactt ttgtgtaatg cttaataaca tacaatcgtg | | | | | | |  |
| 19561 ctcatgttga tatttggtag ccaccacccc caaactcaat tattagcaaa tctcctgaac | | | | | | |  |
| 19621 gtagccatgg gattgagatt tgtatttctt ttcattttta gggtctgcct ggcccctcag | | | | | | |  |
| 19681 gtcccgctgg tgaagttggc aaaccaggag aaagggtgag taaaacaagt aatagtaagt | | | | | | |  |
| 19741 agtaactact aaacttgaga atttccccct gtttaatacc ccactgctat gcaattataa | | | | | | |  |
| 19801 tatgtaaaag aaaatttcgt atttcatatg ttaatgatag tgttttacat actttggtgc | | | | | | |  |
| 19861 tgatggagag aatgagccaa attactttag ttctgattac tttgttttac agatttaatg | | | | | | |  |
| 19921 aaacatcacc ttatgaaagt aaaatctatc aatgaatatt ttatttaata gccttacttt | | | | | | |  |
| 19981 ttgtattgtt cttgataacg ttaagataca aattatttcc ttccccatag tgaaaaagta | | | | | | |  |
| 20041 aatgcacaat tttcaatcaa actagatccc gaaaaattcc ttttgtgttt ttcttggcat | | | | | | |  |
| 20101 tcagacatga cactactata cacaatcagg gcatgagttc tgagtcattt tctctctaat | | | | | | |  |
| 20161 tgtgatgaat gtgcccctat ttagttacat tctgtggcct ggtctccttt gtcaacagta | | | | | | |  |
| 20221 ggacatatta aggagacagc tggtcagtaa taaaagagat acacttgggt atacaattaa | | | | | | |  |
| 20281 ctaggcaatg tacagaatat gataatttct cttaagaaga cgatctgtta tacagctaaa | | | | | | |  |
| 20341 aataggcaat gtctaatatt cattattatt tatcttatta ttgaaggaaa tagtctgtca | | | | | | |  |
| 20401 ctttttaaaa agcaataaac taaataatgg gaaacaaatt ttttgatacc aagttctggg | | | | | | |  |
| 20461 atggatacat ttttgtcacc aataaaattc tctctttctg tatctttcca tactaaaagt | | | | | | |  |
| 20521 tgttcttatt agcctgtgta cttatgcact catgtagata ctgccaggtt tatttcactc | | | | | | |  |
| 20581 tttccaaatt tttcaaatat tttaatcata agtgaattta gagatcacac acagatttca | | | | | | |  |
| 20641 tgctttattc tcatgttttg tctagggtct ccatggtgag tttggtctcc ctggtcctgc | | | | | | |  |
| 20701 tggtccaaga gtaagtgtta cttcattaac tttcataaac tctggcaatg tgtttttaaa | | | | | | |  |
| 20761 agtagtagtg ctttctcctt aaagccactg atgaccctgc aacaagtctc tgatgctctt | | | | | | |  |
| 20821 ctatagtcaa atgtaatctg tagaaagcat tagatttcta agttgatagt agaatttttt | | | | | | |  |
| 20881 gtttcattgc tcatatttcc tattcaataa ttacatagtt ataagaaaca cataaatcaa | | | | | | |  |
| 20941 tatatattat agtcagtgat ttatatagac aactatgcta catttgtgac agtggctcaa | | | | | | |  |
| 21001 cttgagctaa ggaaaaataa tatgttacta agatataaag ttaattttgg ccatgtgtgt | | | | | | |  |
| 21061 tgatgttcaa agcctaaagc cgaacttatg agtagtcata taaagaaaaa aaaacttagt | | | | | | |  |
| 21121 ttctctatgg gtattagcat cactgaaatg attaatttgc cctgaaagta ttccatcatg | | | | | | |  |
| 21181 tcactagtta acacatatgt aggaagctca aagaaccaac ttataacaag gtcctttgaa | | | | | | |  |
| 21241 acagttacaa cgtggaccta tgtgataaat attttgggct atagaatgct atgctctagt | | | | | | |  |
| 21301 gatatttaga tgtaaattgg gagatattta gatagacaga catatatata tatacacaaa | | | | | | |  |
| 21361 tacatatatc aaatatacat atgaatattg taactgttat atcattatta cacagggtta | | | | | | |  |
| 21421 taaaaagggg gcatagatag gagaatatct aatgttatct acacctgcca ttgctattca | | | | | | |  |
| 21481 actaaaatga cacaatcttt tctttgaata ctacacataa ctggcatctg tttttaccta | | | | | | |  |
| 21541 tggatttatc acagaaaaga ctcctcaaag aaggggaatg aattgcaaaa attgaatatt | | | | | | |  |
| 21601 atactctaga agcaacgaat tctggagtcg tagtcatgga acattagagc taagtgacac | | | | | | |  |
| 21661 tttagagaat atctcatcaa tctcttcatt ttactagtgg agagattagg aacaaaagaa | | | | | | |  |
| 21721 attttaattt gctaataaat gcaaaccagg gctcggaagc tacacaaatg taaactctca | | | | | | |  |
| 21781 tatgtaaaac agtatcactg aaagtgatga atggtgcaac acttcttcta atcacttttt | | | | | | |  |
| 21841 tcagggggaa cgcggtcccc caggtgagag tggtgctgcc ggtcctactg gtcctattgg | | | | | | |  |
| 21901 aagccgaggt ccttctggac ccccagggcc tgatggaaac aaggtaaaat cttatgtttt | | | | | | |  |
| 21961 ctatattgct ggtttggccc agtctgcctg gaataagtag accctttaca atagaaagat | | | | | | |  |
| 22021 aattgttttt cagattttta tttatttcca gttctgtgat gacttccctc tcagtaaaca | | | | | | |  |
| 22081 gcaatccgat tccagtggac ctgaattatt ctaaacaaac aaacaatagc aacaaactgt | | | | | | |  |
| 22141 gggggaaaat tcagagttcc caaaacataa atgaattagt atgggttgtc actcttttct | | | | | | |  |
| 22201 cctcacgctg tttatgcttt tgttttaatc tagaaacatt gtattcattg gacattattt | | | | | | |  |
| 22261 tcagagaaaa taacttttta tcttaacatc tcatcccata gagtaaaatt tcaacaagta | | | | | | |  |
| 22321 gtctgacttt taataataag agtttatgat gatgaaaatt cattgggcaa tacattcacc | | | | | | |  |
| 22381 cccaaaaatt tgtctggaaa cttgtgttcc aaaatagaat ctgtggttta gattttaaaa | | | | | | |  |
| 22441 tagatttaat atacattcct gaaaaagaga ttaccttaac cacaataaaa agaagacaac | | | | | | |  |
| 22501 acatattatt ttcattctta actctagggg aaaaaatgta aacattagtt gcaaaaagct | | | | | | |  |
| 22561 attttagtgt atggaaggat gttcttggga aaaaaataaa aacataaaag ggagagagga | | | | | | |  |
| 22621 aataaagaaa ccacggtttt gtgaggtagt actttcaaag ggatctatgt atctcagaag | | | | | | |  |
| 22681 ctagtcaaca ggttttaagt atgtggaatt gtagggtttt atataaaaat ggaagataca | | | | | | |  |
| 22741 gtctctactc ttaaggagat taaaacacaa acatctcctc aattgacaag gtctctttcc | | | | | | |  |
| 22801 atgctttcta tctgggctaa gagacttatc cttgaaaaat gtttgtgggt aaacattttt | | | | | | |  |
| 22861 tactctctgc ttcccattgt cctatcctct tctccatgcc tgccatcctt aagaggactg | | | | | | |  |
| 22921 aagcaggtta tagaggaatc gcagctgtgc actcccacta ccctcatctc ttcagtcacc | | | | | | |  |
| 22981 atgtcattaa cagcatctct ctctgctata ttctccctcc tttcaatagc ccagccttct | | | | | | |  |
| 23041 ttgtgtttca aagcaggcaa gaagcctgtc tagctagctg tttaaattgg aattcttcta | | | | | | |  |
| 23101 gagtttgatt cttcattttc ttctttctcc actaaaattg atttcacatg tgtttgactc | | | | | | |  |
| 23161 aagggtgaac ctggtgtggt tggtgctgtg ggcactgctg gtccatctgg tcctagtgga | | | | | | |  |
| 23221 ctcccaggag agaggggtgc tgctggcata cctggaggca agggagaaaa ggtacgtgtt | | | | | | |  |
| 23281 gacccctatt acatattgtt gatgaactct agtaaagaag gctgcacaag gatgcccaag | | | | | | |  |
| 23341 ttttcacaat tcttggcagg tggtctggta gcattttcat atctatctat atacatttcc | | | | | | |  |
| 23401 ctctgccacc tagcacctac acatttctaa actcactaat ctggcaaaat tccttgctac | | | | | | |  |
| 23461 catggaattt cacacaaaca gatggtgttg agtaatacat gaggctcatt ttaatgccac | | | | | | |  |
| 23521 taacaataat gcctcatcct gtcctaatta atgggaagaa gctacattga acagctgtca | | | | | | |  |
| 23581 accatgctgc tgcattagtt atgccgtaag agtgatcagg cgctgcagcc cattgtgatg | | | | | | |  |
| 23641 ttgccttaca attctgtcca catgaatctg taccttgctt gattatgctt caggagagtg | | | | | | |  |
| 23701 tacggaaatt agaaaagatt gtttaacaat aatctggaaa tggccttgaa ttattttttc | | | | | | |  |
| 23761 ctcattattt ttctcgatta acattctaca gaatggtaag gaatcgagac attgctaaaa | | | | | | |  |
| 23821 atcttaaatg actgaaggta tcatagcatc ttctgtaaaa aagaaaaaaa cttcatatta | | | | | | |  |
| 23881 atttcgattc aaaattttgg tcagaaaaca aaaagttgct cttgctttat actttcaggg | | | | | | |  |
| 23941 tgaacctggt ctcagaggtg aaattggtaa ccctggcaga gatggtgctc gtgtgagtag | | | | | | |  |
| 24001 aattttgttt gtatgtttct tcgtacttgg attttttttt tatgtttgaa ttgagaagtt | | | | | | |  |
| 24061 ttccaaatta gaactacaca cactttattt atcaagttta ataaaataat attcctttct | | | | | | |  |
| 24121 ctcctgggtc tatgactaat aatatcattt tacatgttcc aaaggaaaaa ttaaatggga | | | | | | |  |
| 24181 tttaacctct ttgaaaataa tatcctgaat tttctaactt ccctagtgtc aatgatacca | | | | | | |  |
| 24241 actacaaact atagaccaaa agctttaggt ttaatagaat attaaatgat tgcttcaagt | | | | | | |  |
| 24301 gataacagag attaaaataa ataaataaat aagtctccta tgctttagga agccgggacc | | | | | | |  |
| 24361 tctaacaaga ttctatagtt attcaaacct actccctaga aatttatcac ccaaagagca | | | | | | |  |
| 24421 gccccaaaga ttagctgtta atgccatgaa gatgccaaag ataatcccat gacagtctaa | | | | | | |  |
| 24481 ttaccttatc tcgtatgtca gcctcatggg tcttctaggc cacagtcggc ctggattcct | | | | | | |  |
| 24541 ttattcacct ctccttcaga gctgaaaact gactgtagca catctgtaat agtctttctt | | | | | | |  |
| 24601 ttgaatcacg tagttctaac agtttcaaac aaggctactc atttgctgct ctccagggaa | | | | | | |  |
| 24661 ttttacaata gcggaaagtt cagatctccc aaatttctga cctgctattg acttacacat | | | | | | |  |
| 24721 ttccataacc tttattactg gagtaccctc cttctgagag tggcttctaa tagtcttgtt | | | | | | |  |
| 24781 aattagaacc aaaatacatc agaggccttc tagatatcca accagagtgc agtgaaagtg | | | | | | |  |
| 24841 ttcagtcact gtataagcac agaaaaaaag aatgacaagg ttcacttttg atgatacggg | | | | | | |  |
| 24901 gtgttattaa taagacatgt ttcctttttg gtactagggt gctcctggtg ctgtaggtgc | | | | | | |  |
| 24961 ccctggtcct gctggagcca caggtgaccg ggtaagcatg cattttcact aagccaacag | | | | | | |  |
| 25021 caatatctaa aatttcccgc cttccctagt cccaaagagc cccagcaatt catttttatg | | | | | | |  |
| 25081 gcttggtata aagcctactt atttaaaaac ctagctattg tgatagagca gcaggaaaca | | | | | | |  |
| 25141 aatgctgtgt gtttaaaatt acttttccct tcctatagat ttgccagcta tctgatctat | | | | | | |  |
| 25201 actctaatcc ctagcatttg ttttaaagtc tctccatgtt gcgcattaac aatatcctaa | | | | | | |  |
| 25261 tgcactgagg cttctcaaag ccttcaatta ttaccaaaaa atcaataaaa tacatagtgt | | | | | | |  |
| 25321 gcccatttca cattgaactc tccacttaaa atagatctta tttattgtat tgcaaagatt | | | | | | |  |
| 25381 gccacaaata gatcagcccc gtgtccatct aaaaattaaa atgtcctcct cctggtattg | | | | | | |  |
| 25441 taggcactga tttatagtgt tttctcaagt gtataaccca tactacttaa cccccaaaat | | | | | | |  |
| 25501 gaatatagca ttaagtaaaa atccacttca ttttactctg tgagatgtgc gtcagttatc | | | | | | |  |
| 25561 tcttccaagg caactacaga ctctgtctgt ccaccactgt tctctctccc tcccagttct | | | | | | |  |
| 25621 ttgagcatct atgtcaggca cattaacaga ttcatctttg gtcccattat agggcgaagc | | | | | | |  |
| 25681 tggggctgct ggtcctgctg gtcctgctgg tcctcgggga agccctgtaa gtaagaacct | | | | | | |  |
| 25741 gggtcatttt gtatactcac acctcacaat gtttagacat tgatgaacct aggattgata | | | | | | |  |
| 25801 acacattttt aaatcccttc tcccacctag ggtgaacgtg gtgaggtcgg tcctgctggc | | | | | | |  |
| 25861 cccaatggat ttgctggtcc tgctgtgagt atcacataat gaagattaat ctgaaaacat | | | | | | |  |
| 25921 cctaagttgg ggagtagagt gggtcggaat accagagctg taactgttta tttccaacag | | | | | | |  |
| 25981 ggtgctgctg gtcaacctgg tgctaaagga gaaagaggag ccaaagggcc taagggtgaa | | | | | | |  |
| 26041 aacggtgttg ttggtcccac aggccccgtt ggagctgctg gcccagctgt aagttgaatt | | | | | | |  |
| 26101 cactggtggt ccacacagca gctacccatt agatcttcca attaaatata tatccgtcaa | | | | | | |  |
| 26161 gtgcctgcta tgcaacaggg aatataccag atagaagatg gaaaataacg gaaggattaa | | | | | | |  |
| 26221 catttgcaca ctgctttaca aagtataaaa gtttcatgaa tattgtttta ttttaattct | | | | | | |  |
| 26281 ctgataacct cataagggtg gtaatattga agaacattct gacacagata gtcatttttt | | | | | | |  |
| 26341 atttctatat tttcttctaa gagatgcggg aatgatccac ttgaagaaaa gagtagcatt | | | | | | |  |
| 26401 tacaagggtt tgtttgtgat ttgactccat ctttttgttt gcatttaggg tccaaatggt | | | | | | |  |
| 26461 ccccccggtc ctgctggaag tcgtggtgat ggaggccccc ctgtgagtat ttacaatgga | | | | | | |  |
| 26521 ctctcgccgc ttttcttttt tcagaatcta ttaaggacac ttgaaagttt tgaaattttt | | | | | | |  |
| 26581 ggtaaatttg gactaccatg aggaaacttt tgagattcaa gttcattcta ttcagagcaa | | | | | | |  |
| 26641 ttccgatatt gatgttaact tgaactcagc tggaactcag tgtatgttgc tatcagctca | | | | | | |  |
| 26701 cttgaggtaa taaccaaggt gggccctagg cagtttaatt gtaaagtcgg aaaaaatatt | | | | | | |  |
| 26761 ccttttggcg tttattaata tgccccttct tctgcctgac catgtccttc tcctttgcag | | | | | | |  |
| 26821 gcaatgctat cacaacaatt ctctagagac ccagagctcc ccaaaaatga actttactga | | | | | | |  |
| 26881 cttcttctct cactggacag tgctgaatta tctaggtcat ttgttattct tttgtccatg | | | | | | |  |
| 26941 aacaccatta cctattaagt gtccatttcc ttaccactca gccaggtggt aaagatagtt | | | | | | |  |
| 27001 attaatgtat acacattaat gtgtaataat gacatagtgt cttatcttca tacctttaca | | | | | | |  |
| 27061 accataagat aatatgtcag catttcagaa aggaccatcc aaaccttaac gcaaaatatg | | | | | | |  |
| 27121 ggcattgcaa ctggtaatat gctggtaagg aagatgtgtg gagaaggagg gccttcaggg | | | | | | |  |
| 27181 tcctggctaa ataatgccct atatgaagct gcctacctcc tactccttgg tctattcctg | | | | | | |  |
| 27241 gtcacatgta ctgattttcc aaaacaaaga aattcccatc ttacccaaat tcttggagtt | | | | | | |  |
| 27301 gatgttgact gtggaattct aatgtgcttg gctcttaggg tatgactggt ttccctggtg | | | | | | |  |
| 27361 ctgctggacg gactggtccc ccaggaccct ctgtaagtaa atcactgtaa acgtgtcttc | | | | | | |  |
| 27421 atttactcta gccaaaaggc ctggcttctg ataggaaact ggtaagaaac tcttcatgaa | | | | | | |  |
| 27481 aacacatcac taatattcgc tattactctc ctggtctgaa gtcagctttt ctgaaccatt | | | | | | |  |
| 27541 aaggtatttc atcacaagtt atattttata atatcagttt aagaggcttt tattcatgtg | | | | | | |  |
| 27601 aacaccagtc ccctttcagg ggcatggtct ttttgaaaaa aaaaaaacaa aaaaacgaac | | | | | | |  |
| 27661 agttttagcc acatatcaga tatttctata tctaattatc ctttatggct aacattctgc | | | | | | |  |
| 27721 ctccattgtt aaggtataat tgttcctgaa tttaaaggtg gtttggcctc taatttaatt | | | | | | |  |
| 27781 ctgattcaga ctctcctgtc aggactcaag aaaatttaat taattaccaa ggattaagtc | | | | | | |  |
| 27841 ttctggttaa ggtttctggg aaaaaaaaat agcaaagatg ttgatttctt ggaatccttt | | | | | | |  |
| 27901 tacaggttca taacagaaaa atcttcattc cctgtaggca tttaattaaa cctagttgag | | | | | | |  |
| 27961 aagtgtgtgg gattcctcaa ttatgaacaa aacacgtata ttggctttct ttaaaaaaaa | | | | | | |  |
| 28021 aaaaaaagaa gaaaaaagaa aaggcaaagt ccttcgaaac tcagagtccc attcatttat | | | | | | |  |
| 28081 cattaactcc tatcattcta catagttctg attccaatat gccagggtac cagtggcatg | | | | | | |  |
| 28141 acattgtttt tcctcataga aatttgccat agtctctcct ccattatttg gttggttaca | | | | | | |  |
| 28201 gcctcataaa ggaagacagg agttgcttct ttctgcaaga aagaaggtta aaaactataa | | | | | | |  |
| 28261 atatttcccc caaatggcca gggtattatt ttattgcatc acattgtttg catcttaaga | | | | | | |  |
| 28321 tctagaatct ttgctgctct cttccaggcc cttggtgatt aacagaaagg aaatgacctt | | | | | | |  |
| 28381 gtacatttgc tcatagggta tttctggccc tcctggtccc cctggtcctg ctgggaaaga | | | | | | |  |
| 28441 agggcttcgt ggtcctcgtg gtgaccaagg tccagttggc cgaactggag aagtaggtgc | | | | | | |  |
| 28501 agttggtccc cctggcttcg ctggtgagaa gggtccctct ggagaggctg gtactgctgt | | | | | | |  |
| 28561 aagtgatttc caactcctct ttcttaatac cttatgctga attaaaataa agcccctaca | | | | | | |  |
| 28621 caaatcttca agtggcatct atttgttgat gagtattgca ggctctcaag tagagctcag | | | | | | |  |
| 28681 ttgagccagg aaatctgtcc agcacacact gaggggctgt ggcttccaag atgctcagaa | | | | | | |  |
| 28741 agcacaaaat cgggaagaca ataaatgagg gaactcagtt ttatcacaaa gcccttaaag | | | | | | |  |
| 28801 ctattgaagg caccttactg gtaccaggat tagaacagag tcccattgct gtggccatcc | | | | | | |  |
| 28861 tactacatat taatcaatct cagtaggcta ccaatttctt aaacatacac tgtccagtat | | | | | | |  |
| 28921 tagtgctact ctgaaaggtg cccctattag accttagagg ccaaagtcaa aatttgcttc | | | | | | |  |
| 28981 cttttgatat actgatttta ctgatttcct ttttgttttt gttttttgtt tttgtttttg | | | | | | |  |
| 29041 tttttttgtt ttgggatgga gttcccctct gttgcccagg ctggagtgca gtggcacgat | | | | | | |  |
| 29101 ctcagctcac tgcgacttcc gcctccccag tttaagtaat tatcttgtct cagcctcccg | | | | | | |  |
| 29161 agtagctggg actacaggca cacaccatca tgcctagcta atttttgtat ttttagtaaa | | | | | | |  |
| 29221 gacggggttt caccatattg gtcaggctgg tctcgatctc ctgacctcag gtgatccacc | | | | | | |  |
| 29281 caccttgacc tcctaaagtg ctgggattac acatgtgagc caccccaccc agcctgattt | | | | | | |  |
| 29341 cctttccttt gtgtatatac ccagcagtgt gattgctgga tcttatggta gttctatttt | | | | | | |  |
| 29401 tagttttttg aggaacccct cctactatgt acaactatta tgtatccata acaatttaaa | | | | | | |  |
| 29461 tttttttatt tgtttccctg cctagaggct ataaaaactc tatttcacca ccccaagtgt | | | | | | |  |
| 29521 ctttataaat ctcaaccaca tatttttaaa tgttgtgcca ttggtctcaa ggatgaatca | | | | | | |  |
| 29581 gatacaaaag tattcatgcc aagatgtaaa ctcaccgtca tcactagaga aaagatatcc | | | | | | |  |
| 29641 aaggatatgt cctagtaata ggaggtcatt agcctttttc taagctgaag acagtttatt | | | | | | |  |
| 29701 ctcacaatct tcaagccaac ctgtgttatc acctagggtc ttacccataa tactcagtat | | | | | | |  |
| 29761 tttttctcta tttagggacc tcctggcact ccaggtcctc agggtcttct tggtgctcct | | | | | | |  |
| 29821 ggtattctgg gtctccctgg ctcgagaggt gaacgtggtc taccaggtgt tgctggtgct | | | | | | |  |
| 29881 gtggtgagtg cttgacagta ttctgactcc attaacataa gaaaagattt taaaagctgc | | | | | | |  |
| 29941 cacttcaaat gtgacagatt gatcactgaa taacttcact taagattttt attcatggca | | | | | | |  |
| 30001 ttttctttat acagatcaca tgtcacttat ctaagaagct ttaataccac cttacttaga | | | | | | |  |
| 30061 cacacactat aaagacacag cttaaaatta tgcagaatga tttttgttct ttccacgcac | | | | | | |  |
| 30121 ttaggaatga tacaatctct aattgcgttt actcctctgc aatatgaaat gctggcatca | | | | | | |  |
| 30181 tttatcctgt aggaagaatg aaactctgga agttctgaat cgttccatta aaccttatac | | | | | | |  |
| 30241 gtgacaacaa ctagaaacca tctcatctcc ataaaatata tcaacttttt gaattcattt | | | | | | |  |
| 30301 catttgggat actgaatgac acgaggctca ctttttacag agcaacatcc cgtgattaca | | | | | | |  |
| 30361 taaagctggc catctacatg tggagaagga gggcagagat gatactaatg atacttctta | | | | | | |  |
| 30421 ccattgtgtg acccattcac attcaattta ccttcttcct tcaaactaga atctcctgag | | | | | | |  |
| 30481 tagggttgtt ttggagggga aggttagcat tccatcgaat aaggggaatg tcattttatc | | | | | | |  |
| 30541 ttctctgcct gtttagggtg aacctggtcc tcttggcatt gccggccctc ctggggcccg | | | | | | |  |
| 30601 tggtcctcct ggtgctgtgg gtagtcctgg agtcaacggt gctcctggtg aagctggtcg | | | | | | |  |
| 30661 tgatgtgagt ccaacacttg gtttgtaaaa taaaactgag caggatttca ttgtgtgaaa | | | | | | |  |
| 30721 ctttatgtcc tgagctgagg ttctcttctt ccaaatttct gaacaagatg gtcagcttct | | | | | | |  |
| 30781 ccatagtatc tacacctagt actgaaaata tgaaaattgc ttaggccaaa gaatgggctt | | | | | | |  |
| 30841 ttcaatagca cactgcaaaa ctgggcccaa gtatttaaaa catctctaaa aaatatctag | | | | | | |  |
| 30901 gttggcaggt ttttatccct agttttaaca gtctgaaaga gggttcgtta ctgagcactg | | | | | | |  |
| 30961 gaagtgatga agacagagta gctacaacat aggggctggt aggcagcaga gcctcaccaa | | | | | | |  |
| 31021 cagccttaat ttgtgtggtg tcttcacagg gcaaccctgg gaacgatggt cccccaggtc | | | | | | |  |
| 31081 gcgatggtca acccggacac aaggtcagta cacttttcat ctttctctaa ttcaaaagtg | | | | | | |  |
| 31141 attaaaatgc aacccagatt gatgctaagc ttcattttgc ctttggtagg gagagcgcgg | | | | | | |  |
| 31201 ttaccctggc aatattggtc ccgttggtgc tgcaggtgca cctggtcctc atggccccgt | | | | | | |  |
| 31261 gggtcctgct ggcaaacatg gaaaccgtgg tgaaactgta agtttgtgaa taccagtccc | | | | | | |  |
| 31321 tcagtgcagc attctcgtgg gcttcacttc tgacttcccc acacttgggg atggtggagg | | | | | | |  |
| 31381 agtggggagg ggtatcttgg gcctagctaa gttgtgtttt tctttttcat ttcacagggt | | | | | | |  |
| 31441 ccttctggtc ctgttggtcc tgctggtgct gttggcccaa gaggtcctag tgtatgtaca | | | | | | |  |
| 31501 tgctgaagat ttctttgcaa cactaacatt tagagagaat cagtccaaaa catctgttaa | | | | | | |  |
| 31561 gaaaataaac aatatatcag ctagacttaa tattttttaa aaatttcagt ccatgctgag | | | | | | |  |
| 31621 aattgataca aataacttga gctattttaa atctcttatg cttgtttgta ttaaacaatt | | | | | | |  |
| 31681 acaaggatct agccacttta cagatagcac aactaaagca gattaccagc agaggtgaga | | | | | | |  |
| 31741 gcctagctaa accattacat gtcctgagtt acctttgtaa acgaattaag cagtatttgt | | | | | | |  |
| 31801 ggtgaagtga gtgccatttt tttaaaacgg taagtcttat ccatccttct gtttctttat | | | | | | |  |
| 31861 agggcccaca aggcattcgt ggcgataagg gagagcccgg tgaaaagggg cccagaggtc | | | | | | |  |
| 31921 ttcctggctt aaagggacac aatggattgc aaggtctgcc tggtatcgct gtaagtaaac | | | | | | |  |
| 31981 tgtagccatc tcgcacataa actgatcctg aaggccttca gctcagaagg attttcatat | | | | | | |  |
| 32041 tttcactgct attgttccag tatagcctat ataatatcca tttcccattc tctggctaac | | | | | | |  |
| 32101 tccatctcac tcttggaggt aatgctattt cataccaaca tgaaaggtga ggattaaggg | | | | | | |  |
| 32161 agatagaaat agacatacaa taaaatctcc tggtaacaat gtccttcaac cccacttaaa | | | | | | |  |
| 32221 ataaacataa ttagaggaat gactaatatt gcactgctga aataggttgt gaaaaaaaaa | | | | | | |  |
| 32281 ttgaatataa tagacataat gggagaaaag agccccactt tacattttca attttctcaa | | | | | | |  |
| 32341 tccggagtcc atttaactaa agtttcccat tcaatttgga aaaaaaaaaa aaatatgtct | | | | | | |  |
| 32401 cttgacatgt gctctgaaag tgtgattttc ctcttctgtc tttaaagggt caccatggtg | | | | | | |  |
| 32461 atcaaggtgc tcctggctcc gtgggtcctg ctggtcctag ggtaggtgga ctcaagagaa | | | | | | |  |
| 32521 gacagttcat ctctgaaata gaggctaaag cgagcagtga gccccaggct gctgctccct | | | | | | |  |
| 32581 tggtgggatt caccagctca catgtacctg gtgtctgtct tccttagggc cctgctggtc | | | | | | |  |
| 32641 cttctggccc tgctggaaaa gatggtcgca ctggacatcc tggtacagtt ggacctgctg | | | | | | |  |
| 32701 gcattcgagg ccctcagggt caccaaggcc ctgctgtaag tatgatttgg ggaaataata | | | | | | |  |
| 32761 aagaagatca cggacctaag gaatgttttc ttcagactaa accaagacaa ctttgacaac | | | | | | |  |
| 32821 ccattaaagt tagccccatt tcaatatatc ctctaaaata tctggaaatt gtctatatgc | | | | | | |  |
| 32881 aatgggcttg ttaagtccat ccctgcaagt gtgcctgggg gctcgttatt tatttatgtg | | | | | | |  |
| 32941 aacttgatta ttttttactg atgagaacat gcttccgtgt gaagctcaac tgaaaatctg | | | | | | |  |
| 33001 ctgccatgga tgtctctcac tgtaacaaaa tataaagcct ctcctatctc actttcacct | | | | | | |  |
| 33061 ttgcagggcc cccctggtcc ccctggccct cctggacctc caggtgtaag cggtggtggt | | | | | | |  |
| 33121 tatgactttg gttacgatgg agacttctac agggctgacc agcctcgctc agcaccttct | | | | | | |  |
| 33181 ctcagaccca aggactatga agttgatgct actctgaagt ctctcaacaa ccagattgag | | | | | | |  |
| 33241 acccttctta ctcctgaagg ctctagaaag aacccagctc gcacatgccg tgacttgaga | | | | | | |  |
| 33301 ctcagccacc cagagtggag cagtggtagg tcaagatgtc cagaccagac tgacccttct | | | | | | |  |
| 33361 cacaagttga gcttttcaaa attagtttcc attgacattt agagtgaaaa tgcatttggg | | | | | | |  |
| 33421 taaagattac attatgtgaa atcacaccca attaatggag cgtcatcttc tcccaaccag | | | | | | |  |
| 33481 cacccaacct catttccctt aaaatgtatt tttgcacttt tcatagtaat aagtaccctg | | | | | | |  |
| 33541 atttgatttt tcatggagga ggggagggaa ggaactgtct aatcttaaaa atagccaccc | | | | | | |  |
| 33601 tcttcctctt aaatatgggg tagacaatca aaaatgttac ttatgagagt cagtatcttt | | | | | | |  |
| 33661 cattagttat tattagaatc tgtgttctgc tcaatgagaa gtttcatgat ctgaatgtta | | | | | | |  |
| 33721 ttttcttaaa aggttactac tggattgacc ctaaccaagg atgcactatg gatgctatca | | | | | | |  |
| 33781 aagtatactg tgatttctct actggcgaaa cctgtatccg ggcccaacct gaaaacatcc | | | | | | |  |
| 33841 cagccaagaa ctggtatagg agctccaagg acaagaaaca cgtctggcta ggagaaacta | | | | | | |  |
| 33901 tcaatgctgg cagccaggtg aggaatccca caaacacctc tccttctgct aaataatatt | | | | | | |  |
| 33961 ttggtaggac tgtttgttaa ttatctgcat tttaatctct gacaaaaatg ggcttattaa | | | | | | |  |
| 34021 aaaaagacct gttcctttcc tgggttccaa ttttgtccta aattgcacat tagaagatgg | | | | | | |  |
| 34081 attgattgga cacatccatg taattcaaag ttattattca aatttgactt aattggtaat | | | | | | |  |
| 34141 cattgaaaaa actgactaat gtcatttagt gtgaaggagc actggccagc tatatgccac | | | | | | |  |
| 34201 actcatacat atgcattttc agaatgtgag cagcttttct gaatttttaa tcaaaccttt | | | | | | |  |
| 34261 tcaccaactt tactgaatgc ctactggaat tccataaatt acaaaatgac agaaaaagaa | | | | | | |  |
| 34321 aaatgtcaga atttctacct cctcattctc ttattctaaa gaagaagcat atgcaaaaag | | | | | | |  |
| 34381 gattaattga aacagataac ttttttagat gaccttgcct cagtctagta ggtcttatgt | | | | | | |  |
| 34441 tcatctaggt aactgatact tcaaagacaa gtgaattaag ttttctttaa aagtaccctt | | | | | | |  |
| 34501 ttcctaagct tggatctgag tctactcttc ctgagatctt tttttttctt tttttttttt | | | | | | |  |
| 34561 ttcatgtttg actcttagta tctgagtcct tctccactta actggaattt catcctattt | | | | | | |  |
| 34621 tctgtagttt gaatataatg tagaaggagt gacttccaag gaaatggcta cccaacttgc | | | | | | |  |
| 34681 cttcatgcgc ctgctggcca actatgcctc tcagaacatc acctaccact gcaagaacag | | | | | | |  |
| 34741 cattgcatac atggatgagg agactggcaa cctgaaaaag gctgtcattc tacagggctc | | | | | | |  |
| 34801 taatgatgtt gaacttgttg ctgagggcaa cagcaggttc acttacactg ttcttgtaga | | | | | | |  |
| 34861 tggctgctct gtaagtaata gtgaaatatg ggaatagctt tgggaagtgg gatggagggg | | | | | | |  |
| 34921 gttctaactt agactgcccc caaggggggg tctaaagggg ggttaaaaga acagaagaat | | | | | | |  |
| 34981 gagagaacta acttatttca taagtaaatt cagtttttgt atgtatttta tatttattta | | | | | | |  |
| 35041 tttatacgta ttaatttcgt acttaaattc agatgataaa ttcagagtat tcttatcaga | | | | | | |  |
| 35101 tagtgccttc tgaaatgctg aaatgtatac tatgtccatg cattgttttt tctttagcat | | | | | | |  |
| 35161 gttttttaaa tggtaatgtg tgcccagaac ttaaaatttc ttgagacttc agtggcctaa | | | | | | |  |
| 35221 actataattt atagttatgt gtattttatt ttacttatta gtatggccta catttaactt | | | | | | |  |
| 35281 ttaatgcttt ttctacaata tgctataaat ataagaaaaa ttaaaattca ctaacagcaa | | | | | | |  |
| 35341 gactacatac ccacccaggt cccgctccca aagacacaca tagagggaca tacacacaac | | | | | | |  |
| 35401 aatcctaaaa atgactttgt agagataggt cacttggaat gtgtgttgaa atgttgttgg | | | | | | |  |
| 35461 tttttttggt ttgtttgttt gtttgttttt tgttagactg atagggagcc cctcccacta | | | | | | |  |
| 35521 aagacaccct tgatactgtt atttcaagga tgaacttatt tatctgggac agacatcttc | | | | | | |  |
| 35581 agaatgacac atgccaaaca gtggttctta ttaaatcaaa ggttcagata ttatcagatt | | | | | | |  |
| 35641 cagaaatagt gatgctttgt gtatctattt tcttctcttt aaacagaaaa agacaaatga | | | | | | |  |
| 35701 atggggaaag acaatcattg aatacaaaac aaataagcca tcacgcctgc ccttccttga | | | | | | |  |
| 35761 tattgcacct ttggacatcg gtggtgctga ccaggaattc tttgtggaca ttggcccagt | | | | | | |  |
| 35821 ctgtttcaaa taaatgaact caatctaaat taaaaaagaa agaaatttga aaaaactttc | | | | | | |  |
| 35881 tctttgccat ttcttcttct tcttttttaa ctgaaagctg aatccttcca tttcttctgc | | | | | | |  |
| 35941 acatctactt gcttaaattg tgggcaaaag agaaaaagaa ggattgatca gagcattgtg | | | | | | |  |
| 36001 caatacagtt tcattaactc cttcccccgc tcccccaaaa atttgaattt ttttttcaac | | | | | | |  |
| 36061 actcttacac ctgttatgga aaatgtcaac ctttgtaaga aaaccaaaat aaaaattgaa | | | | | | |  |
| 36121 aaataaaaac cataaacatt tgcaccactt gtggcttttg aatatcttcc acagagggaa | | | | | | |  |
| 36181 gtttaaaacc caaacttcca aaggtttaaa ctacctcaaa acactttccc atgagtgtga | | | | | | |  |
| 36241 tccacattgt taggtgctga cctagacaga gatgaactga ggtccttgtt ttgttttgtt | | | | | | |  |
| 36301 cataatacaa aggtgctaat taatagtatt tcagatactt gaagaatgtt gatggtgcta | | | | | | |  |
| 36361 gaagaatttg agaagaaata ctcctgtatt gagttgtatc gtgtggtgta ttttttaaaa | | | | | | |  |
| 36421 aatttgattt agcattcata ttttccatct tattcccaat taaaagtatg cagattattt | | | | | | |  |
| 36481 gcccaaatct tcttcagatt cagcatttgt tctttgccag tctcattttc atcttcttcc | | | | | | |  |
| 36541 atggttccac agaagctttg tttcttgggc aagcagaaaa attaaattgt acctattttg | | | | | | |  |
| 36601 tatatgtgag atgtttaaat aaattgtgaa aaaaatgaaa taaagcatgt ttggttttcc | | | | | | |  |
| 36661 aaaagaacat at |  |  |  |  |  |  | |
