# Supplementary material for: Development of a High-Throughput Resequencing Array for the Detection of Pathogenic Mutations in Osteogenesis Imperfecta
Source: PLoS One. 2015 Mar 5;10(3):e0119553. doi: 10.1371/journal.pone.0119553 (PMC4350936; doi:10.1371/journal.pone.0119553)
Supplement: S2 Datasets — Including all the reference sequences of COL1A1, COL1A2, CRTAP, LEPRE1 and FKBP10. (ZIP) [file pone.0119553.s002.zip › OI-CRTAP_sequence.doc]

| 1 aggctggcgt ccccgccccg aaagcactgg gcccgccgcg tcgcaccgtc ctctttcctt | | | |  |
| --- | --- | --- | --- | --- |
| 61 tccttctccc tccccttttc ccttccttcg tcccttcctt ccttcctttc gccgggcgcg | | | |  |
| 121 atggagccgg ggcgccgggg ggccgcggcg ctgctagcgc tgctgtgcgt ggcctgcgcg | | | |  |
| 181 ctgcgcgccg ggcgcgccca atacgaacgc tacagcttcc gcagcttccc acgggacgag | | | |  |
| 241 ctgatgccgc tcgagtcggc ctaccggcac gcgctggaca agtacagcgg cgagcactgg | | | |  |
| 301 gccgagagcg tgggctacct ggagatcagc ctgcggctgc accgcttgct gcgcgacagc | | | |  |
| 361 gaggccttct gccaccgcaa ctgcagcgcc gcgccgcagc ccgagcccgc cgccggcctc | | | |  |
| 421 gccagctatc ccgagctgcg cctcttcggg ggcctgctgc gccgcgcgca ctgcctcaag | | | |  |
| 481 cgctgcaagc agggcctgcc agccttccgc cagtcccagc ccagccgcga ggtgctggcg | | | |  |
| 541 gacttccagc gccgcgagcc ctacaagttc ctgcagttcg cttacttcaa ggcaagtccg | | | |  |
| 601 cctcgccccg tcccaggccc cggccccgcc cctgacccag cctccaggcc ctagccccgc | | | |  |
| 661 ccctgcgccc ggggccgcgt tgcccctcca gttctagacc tggctccctc ccatccagcc | | | |  |
| 721 ctgccaaagg tctcctccaa gtcctgtctc cttcttcatc tcaactagag atgaaaatgc | | | |  |
| 781 ctttcttgaa gggtaactct aaggtagaga atacgtacac agaacgcagt acagcgccag | | | |  |
| 841 gcacccaaca aagtgcctga taaatgttct ctgttatttg agggcagacg cctcttttct | | | |  |
| 901 gtatatacca ttaaagtcgc cttttttctc aagtgagcgg tgacttgcga taataaagca | | | |  |
| 961 gaagacctga ggagccgaca agtacctttt atttttattt tttgttgaga cagggtctca | | | |  |
| 1021 cagtcgccca ggctggagtg cagaggcgct atcatggctc actgcagcct tgacctcctc | | | |  |
| 1081 aggctcaggt gatcctccac ctcagcctcc tgagtagctg ggattatagg cgcgcgccac | | | |  |
| 1141 cacacctagc taattttttg gtattttctg tagagacaga gtttcgccac gttgcccagg | | | |  |
| 1201 ctgatctcga actcccggac tcaagtgatc ctcccacctc gcctcccaga gttctgggat | | | |  |
| 1261 tacagtcgtg agccattgca cccagctgac tagttccttt taaagaggat gaaattgagg | | | |  |
| 1321 ccagggaagg gcagggactc cttgcaagga cacatccctg cttaaagggg aactcgtgca | | | |  |
| 1381 ccgtgagtca tcaggattgg ccctctccgc tgtttctctc tttcatctag aatttaacac | | | |  |
| 1441 agttcactgc ctccttttaa tggctgctta agttctctct gtgattgctg gctaaaaata | | | |  |
| 1501 gccacaaatt gcagttctct atcctatggg ttctcactcc gaggacccac aagttttaag | | | |  |
| 1561 aatggttaga ttccctgtca ctagataaag aggctctagc ttggtgggtc ctcacagggt | | | |  |
| 1621 ttggctaaaa gagccctctc aaagcccagt ttctactttc tctgagaaag attttaaaag | | | |  |
| 1681 gaatatattg tgttttttaa aattactaca aaaatagatt catggcatat cctagactct | | | |  |
| 1741 tgaggtagaa ggtttttgtt tttaaacttt gttttcagtt tctggactgc aagatctgtt | | | |  |
| 1801 ttttcagatt tccaccgaga cagagccacg tgtatattgt atttatatga gcaataaaat | | | |  |
| 1861 aacacaaaac cccaaagagg ggaaaagctg ggttacttct atacttctat gcttagttac | | | |  |
| 1921 agttttattt aaagtatcag ttttgtgaat gtttgttctg gcaattaaaa aaaaacagtt | | | |  |
| 1981 ttaaaacaga agcagggaat gagattaata aaattccaga atgttgaaaa tgagggctgg | | | |  |
| 2041 ttcatacaaa gcatcatttt ttaagctaaa aatgacttat ctaattcttt ttttgtatag | | | |  |
| 2101 cttttggcaa atatgttctg aaccaaattt aggtcacata atcaagagga aatcatttta | | | |  |
| 2161 tttttaaacc tagagaatac tagaacctct gatttcttta atatacagat gaatttgaac | | | |  |
| 2221 aagtgttaca gggaagaggg gaagaacatt ttactcaatg tgaccaaaag gcatgggttt | | | |  |
| 2281 tctctttcat catggtggcc ccaaaaggtt aggatggtca tagcttctct tcaaaaccct | | | |  |
| 2341 ttacaggtct tgtaacaatc acttagttga aagtcagtaa aatactatct atctatctgt | | | |  |
| 2401 ctatctatct gttttttttg agacacagtt tcactttgtc acccagactg gaatgcagtg | | | |  |
| 2461 gcacgatctc agctcgctgc aacctctgcc tccagggtgc aagcaattct tgtatctcag | | | |  |
| 2521 cctcctgagt agctgggatt acgggcatgt gccaccacac ccagctaatt tttgtatttt | | | |  |
| 2581 tagtagagat ggggtttcac catgttggcc aggctggtct cgaacttcta acctcaagtg | | | |  |
| 2641 atccacctac cttggcctcc caaaatgctg agattacagt ctgggatttg aaccaccatg | | | |  |
| 2701 cccggccacc atcttaacat tttgagatct catatagggc aggaaaaagc taagaatttt | | | |  |
| 2761 attaataaac gtgaatgtta cttaattgtt tcaatggaag atataactga aaaagtaatt | | | |  |
| 2821 tttggtggag atattgtatg gtgtattttt tctcaatgat gtttatggct tggcatgatg | | | |  |
| 2881 gctcatgcct ataatcccag cactttggga tgccaaggtg ggaggatctc ttgagtccaa | | | |  |
| 2941 gagttcaaga ccagcctggg caatataatg agatctcatc tgtacaaaaa cattaaaaaa | | | |  |
| 3001 ttagccgggt gtggtggcgt gcctttctag tttcagctac tcaggaggct gaggctggag | | | |  |
| 3061 gatcacttga gcccagaaag tcaaggctgc agtgagctgt gatcttacca ctgcactcca | | | |  |
| 3121 gcctgggtga cagaacaaga ctcttgtgtc aaaaaaaagt gatgtttacg tgtttaaact | | | |  |
| 3181 ctgtttatgg atagagagga aaagatgggt ggcaggcaca tacattctac ctggcacctg | | | |  |
| 3241 aaggctggag gtcgatatga aggtagatag tgcgctgtgg caggacatgg ataaatgtta | | | |  |
| 3301 ctctgctgtt ctgagttgca gttctctgtt tcagtgatta aggagaaatg cctcaattga | | | |  |
| 3361 tcactggaat agcaatggtg ggttccgact gccagaggga ggcgaggagg tttggatgtg | | | |  |
| 3421 tgctcttgta gaaaagggct gtgctactgg tgtgcagcct caggtgcatc ataggtttct | | | |  |
| 3481 cgtcaccagc ctactctagg tccatgtcat tagctttctt tgggttctct ccacaattac | | | |  |
| 3541 catcttccac gaaaaactca tttttctcat aggccaagca aaaagtaggc cggagcactt | | | |  |
| 3601 catcaaatcc ttaatgactg ttgagctcta acctggacag agaacactga ctcacaggca | | | |  |
| 3661 ctgttaaccc caggccttgc ctggctgtgg cctctaactc cccggaacca cagggcctgc | | | |  |
| 3721 ctctgtgtca tggctgattt agggtggctc atgctggtgg ctgtatctca ggaacccaag | | | |  |
| 3781 gcaaggtggt tccctccatc cctaccacct tctccaaaat ctgcctgggg tctgtcagag | | | |  |
| 3841 gtttggtagc caagatgtgt ctccatgttc cagctgcaga gcctgagttg aagtaaataa | | | |  |
| 3901 aatcactctt ttctcttcag gaagagattc cgttttgtaa aacatcctaa gttagaagcc | | | |  |
| 3961 ttaaaaatcc tactctgtgc ccctctttct attcctttct ttctttcttt ctttcttttt | | | |  |
| 4021 tgagacggaa tctcactgtg tcacccagac tggagtgcag tggtgcgatc ctggctcact | | | |  |
| 4081 gcaacctccg ccccccgggt tcaagtgatt ctcctgcctc agcctcccga gtagctggga | | | |  |
| 4141 ttacaagcac ctgccactgt gcctggctaa tttttgtctt tttagtagag atggggtttc | | | |  |
| 4201 atcatcttgg ccaggctgat cttggactcc tgacctcgtg atccacccac ctcgacctcc | | | |  |
| 4261 taaagtgctg ggattacagg catgagccac tgctcccggc ctctgtgccc ctctttctaa | | | |  |
| 4321 gtgctgttct gagaattagt gactctaatt tcactggcaa atgctttaaa aggcagcatt | | | |  |
| 4381 cataccctct cccttccttc cagctgaaga ctagcctttt tctggccagg agtgtgaagc | | | |  |
| 4441 tttctgtagg gctgttttaa aggcagccct gccacaagcc actaattggc attcccaaaa | | | |  |
| 4501 catcagcatt gacatctggg agtctgccct catcaccacc caccacttct ctgatgtggc | | | |  |
| 4561 cactgccatt gtctcaaaat gacttggttc caattctggg gcaattcggt accgatatat | | | |  |
| 4621 gccatcctat gactcttaga agtctagggt ataccaaata ttcctttatt ttctctgtct | | | |  |
| 4681 aaaccgaatt ccctaattcc cgccttggag cgtccctctg gattggctga gtctctgtgc | | | |  |
| 4741 acatgcacag gtgcctgccc ttctccctac taggttgtgc caagagtcca tcagcatggg | | | |  |
| 4801 ggccctctgg ttggtggtca tgagtcctca cagggaggcc tctgcatcct tatttgcctg | | | |  |
| 4861 ccctcctgag gtcctgccat aacatacctg ttcttcctga ggacttctgc cgtcttgaac | | | |  |
| 4921 actgccgata agtgccagcc ctgaagctcc ggggcccgca gccctgctgc tctcacagat | | | |  |
| 4981 cccaagggaa acttttttcc tagcctggga gattcccttt ctccctcttc ttctcccagg | | | |  |
| 5041 ccagtcagct tcttcctgca gctcctctcc aatttctttc tcccctcaaa cctgtcaggc | | | |  |
| 5101 tcttttgtga gtgcaggctt ttttcaagtg aataatttgc tggttctgac cagatatcta | | | |  |
| 5161 cctcccctga gacagtgaaa ctcagaatca ttggcccctt caatggagag aagacagagg | | | |  |
| 5221 ggaaaagaga ggaaaatgtg aattaatttt attatatttt atggtcataa tgtcattatg | | | |  |
| 5281 ttcaaatcac agatacttat tgtgtcctgc cttgtcctga ggactgtgtg aggcaccagg | | | |  |
| 5341 aatccagata atgaatgaca cacagctggg tcccccgagg atcatagtct gatgggggag | | | |  |
| 5401 atagacgtac cactgagatt gtagggtggc gtaaaatgcg ggaagacaga acccagaact | | | |  |
| 5461 agcccaacag agaggctctt aattgaggcc actaggagac agggagaact taacagaata | | | |  |
| 5521 tatggtccat gcaggtgggg attttttttt cctgttttgc ttattgttat agacccacaa | | | |  |
| 5581 cctagaaatg gtgctcttgt aatatgacgt ttattgctgc atgaatggat gaataagtga | | | |  |
| 5641 ggaggtggtg gagtcattga gaatgttagc agaaggtctg tgggggattt gggaaaacaa | | | |  |
| 5701 tatatttgtc aaggtgccag tggaaaagat atggtgcagt catattgggt aattggagga | | | |  |
| 5761 aagtttaaga aggtggttat ttacaaaggt gtgtgcagtg catggggaat ccacaagaga | | | |  |
| 5821 tagtgttctg ccacaggggt gatagcagtg ggatatgacc aaccctaggc caaaaggggc | | | |  |
| 5881 aggggagaga gcagtgattg aaaccgggaa ggagggagac tctatggaga gggtcacctt | | | |  |
| 5941 gagaagaact gtgatcttca attaagggac atagcagccc aagaggactg tgcagggagg | | | |  |
| 6001 gaaccaggaa atgaaataac atgaccttac tctcttccct tcctcagtca tctactgggg | | | |  |
| 6061 ctcctcatgg gtgaacctct ttggaagcca gagagcgagg gagtctgttg ttatagtcca | | | |  |
| 6121 cacaggccag cctcctgggg catgtagcca gtgcagagta gattgggaaa ggcagaccga | | | |  |
| 6181 ggccatccag acagtcgctt tcaggaaatc agcacttgtc tcacagtgcc tggacttggt | | | |  |
| 6241 cttgaggggc cctggaagtc atggaacctt tagctggaaa agttatagca acacaaagtt | | | |  |
| 6301 tctgaaggac taagcagata ttcttgtttt acaaaaatta ccacttagca tccatggagt | | | |  |
| 6361 agcttttatg ttctttgtcc ttttaggcaa ataatctccc caaagccatc gccgctgctc | | | |  |
| 6421 acacctttct actgaagcat cctgatgacg aaatgatgaa gaggaacatg gcatattata | | | |  |
| 6481 agagcctgcc tggtgccgag gactacatta aagacctgga aaccaagtca tatgaagtat | | | |  |
| 6541 gtttggattt ttatgtggca gttagtggca acctggactg ttaaagaaat gtctctccat | | | |  |
| 6601 aagcagctgc tgatagctaa ggacctctag tgatttttgg tgacatttgc tgaatattat | | | |  |
| 6661 gacaatagaa atgattgact tttagcacaa agtgatccat ttaataatga tattttgtat | | | |  |
| 6721 gccgtgatga ttttaaaagc catgttccct ttaaatgcta cattttagaa atatggtcat | | | |  |
| 6781 ttaattattt agttttaaga gggtagatta aagacatatc ttggaaatca tctaaaatga | | | |  |
| 6841 ccaggaacac aagaaataga aacataaatc tccagttttg atgaaatgag aaggcatata | | | |  |
| 6901 tttttataca cacttgcaca caaacgcacc ctaaatatat taaggtagaa aatgaagacg | | | |  |
| 6961 agttaggaag gctgatgtga cagagtagag gacacttaaa tctgcgcatc tacgaagaag | | | |  |
| 7021 agttttgatg agaattagtc gtaagaacaa caataagaaa gaacgatctc gcccttttga | | | |  |
| 7081 gcactcacta ttgtcaaggt cttctggaac tcaggcactg ggggcctggg tttcaagcaa | | | |  |
| 7141 gggcagggca ttgttgaaaa tgggaatttg gctgggcaca gtggctcatg cctgtaatcc | | | |  |
| 7201 cagcactttg ggaggccgag gcggatggat cacctgaggt caggagtttg agaccagcct | | | |  |
| 7261 ggccaatatg gtgaaacccc gtctctacta aaaatacaaa aattagctgg gcgtggtgac | | | |  |
| 7321 tcagcatgtc tgtaaaccca gctactcgag aggctgaggc aggagaatca cttcatcccg | | | |  |
| 7381 ggagacagag gttgcaatga gctgagattg cgccactgca ctccagcctg ggcgacagag | | | |  |
| 7441 cgagactctg tctcaaaaaa aatgaaagaa aatggggata tgtggaaaat atccactaga | | | |  |
| 7501 agttatagtt cccaaggcct gaccacaggg ttttctgtag agtgatcaga tctcaggctc | | | |  |
| 7561 actttttgac tgctagggaa aatgtatgtg tggaagtctc tttttggggg tatctgtcag | | | |  |
| 7621 ttttccaggg aagacttcac cagccttctg cagggctggt ggggtgcgca tactcctctg | | | |  |
| 7681 ctggtgtctg agaaccagtg tggggagagg gacagggtca cccaggtgag taggaatact | | | |  |
| 7741 tcacctgctt cctgatttta gcctcatgcc tccacttggc ctctgctacg tctggtccct | | | |  |
| 7801 gactcctcag cctttatggg taaagtctct agaataaact gccactctct ggtgaagagt | | | |  |
| 7861 ggctttggga gatatgatct cttgggtggg tgggtaggtg ctgggagcgg acccaggacc | | | |  |
| 7921 ccaacagcct gttcaaactt gcagccctgc ttctagctcc atgccttacc ccagcctccc | | | |  |
| 7981 gtggtccctc atgcctgcgc atgccaatgg gacatatagc ttgcttcccc tctggcccct | | | |  |
| 8041 cctttgtgac tatttcactt cctgcctttc caaaagttgt tgaaatctca tctgctgctt | | | |  |
| 8101 gctcccttcc cattctccca taaaagcaat ttatgccttt catagtcttc actgttgttt | | | |  |
| 8161 tcatagggtt tggggaacga agccgagtgc gtgtggtcag tgccctgggc ttatctggaa | | | |  |
| 8221 ttcagctagt cctgttggcc ctcactggag cccttttggg gtggctgtgc tgccctcctg | | | |  |
| 8281 tgtgatcagg ggcctcctct ttctgcttgg tccttcttgc tgctgctgct gccccgctgc | | | |  |
| 8341 ttctgcagaa ccagctgtct ggagttggct acttattgac ccattcttca ggaaaggctc | | | |  |
| 8401 cttcagttct cagcctgtct tatttccagg gcatgtggcc tgcccaggag gtttaagaaa | | | |  |
| 8461 atccaccctc ggccaggcgc ggtcctcatg cctgtaatcc cagcactttg ggaggccaag | | | |  |
| 8521 gtgggtggat cacgaggtca ggagttcaag accagctggg gcaagatggt gaaaccctgt | | | |  |
| 8581 ctctactaaa aatacaaaaa aattagccag gcgtggtggc aggcacctgt aatcctagct | | | |  |
| 8641 actcgggaga ctgaggcaga gagaattgcc tgaacccagg agttggaggt tgcagtgagc | | | |  |
| 8701 tgagattgca ccactgcatt cgagcctggg caacagagca agactctgtc tcaaaaaaaa | | | |  |
| 8761 aaaaaaaaaa aaaaagaaga agaaaatcca ctgtctttac ctgaggcctt aagcttggtg | | | |  |
| 8821 aaaattctag gtctaagggg tgccctcaga taccaccatt ccagagcagc tgagaactcc | | | |  |
| 8881 aaatggaggg tcccatgctt attcagggcc tgtcctggcc tctgcaagtt agcaggtttt | | | |  |
| 8941 gtgtgtatcc aggatctcta cttatttctt ggagaccaag ctacttcctt ggtcctagtt | | | |  |
| 9001 tatttctctc tctctttttt ttttgagatg aagtctcgcc ctgttgccca ggctgcagtg | | | |  |
| 9061 caacggcacg atcttggctt actgcaacct ccacctcctg ggttcaaatg attctcctgc | | | |  |
| 9121 ctcagcctcc tgttatttct cttctaatcg agtctaatca acaattagca gaaagtcctt | | | |  |
| 9181 gaagtttcca ctatctggca tgtggttgcc cttcctagta actagtgctg atgcatggtg | | | |  |
| 9241 gcacttctgt ttaactgcat tgtcggagga agtggggtga cagacacctg ctgatatggt | | | |  |
| 9301 ttggatctgt gtctccacct aaatctcatg tccagttgta atccccaggg ttggaggtgg | | | |  |
| 9361 ggcctggtgg gaggtgattg gatcatggga gtggatcctt tattaatggt ttagcaccat | | | |  |
| 9421 cctctctgtg ccattctcat gatagtgagt gagttatcat gagacctagt tgtttaaaag | | | |  |
| 9481 tgtgtaacac ctccccgctc tcttccttct tctctggcca tgtgaagtgc cggctccctg | | | |  |
| 9541 tttgccttct gccgtgattg taagtttcct gaggcctccc cagaagcaga agctgctatg | | | |  |
| 9601 cttcctgtat agcctgcaga accatgaacc aattaaacct cttttattta taaattaccc | | | |  |
| 9661 agtctcggtt tttttttttt tttttttttt ttgacagagt ctcgctctgt cacccaggct | | | |  |
| 9721 ggagtacagt ggcacaatct cagcttactg ccacctccgc ctcctaggtt caagtgattc | | | |  |
| 9781 ttgtgcctca gcctcgtgag tagctgggac tacaagcatg tgactacacc tggtaatttc | | | |  |
| 9841 tatattttta gtacagatgg ggtttcccta tattggccat gctagaggta tttctttata | | | |  |
| 9901 gcaatgtgag aacagactaa tatacctgct ctcaaactta catttttctt ttcgttccat | | | |  |
| 9961 tttaaaaacc agttttattg aggtactatt tatatacaat atatttatca attttaagtg | | | |  |
| 10021 tataatgcag taagttttga caaatacatc cagtcctgta actgccatca tagttacgac | | | |  |
| 10081 atagaatatt ttcatcaccc ctgaacaatt tcctcttgtc cttttgtagt caagtttttg | | | |  |
| 10141 ctgtaacccc tgacttctgg cagccactga tctgatctct gtcatagttt tcccattttc | | | |  |
| 10201 cttaattcag attattatca agggcctgct actttttgaa ttgaactaaa taagaaatgt | | | |  |
| 10261 acccatcata agcaaagtag aaaaatctag atataaagta actcactgat tgacttcttc | | | |  |
| 10321 aagggccagt gtggcttctt cagagctaat gagagctagc ttggtggtgg tcttggttcc | | | |  |
| 10381 ctttgagatt tcatgactgt ctcccttcac gcccaagagc gagcttcact ggcttctcca | | | |  |
| 10441 tgcctttcag agcctgttca tccgagcagt gcgggcatac aacggtgaga actggagaac | | | |  |
| 10501 atccatcaca gacatggagc tggcccttcc cgacttcttc aaagcctttt acgagtgtct | | | |  |
| 10561 cgcagcctgc gagggttcca gggagatcaa ggacttcaag gatttctacc tttccatagc | | | |  |
| 10621 aggttggtgg taggtcaata ggctcactac tcccatggaa tagtcttcct tagatgtctg | | | |  |
| 10681 gtttctgcct gcatgcctgc tagatgcctg gagcagcctc tgaggaattt ttgagcatac | | | |  |
| 10741 ttattaacgg gtaactgagg gtagtcacca cctgcataga taaacctttg acttggcagg | | | |  |
| 10801 ggcttctagt gaaatcactg agagaaggtt caagcacagg cctgccaagg tagaattctg | | | |  |
| 10861 tgtctgccct acttgcttac agatttccta cagtcatttt gaaaaataaa aatgggccca | | | |  |
| 10921 ggagcacctc agtgactcac attactgaga taactttgat gatggcaatt ttccaacaca | | | |  |
| 10981 tataaaggta gaatgaacct ttaagtggcc atcactaagg tagctttttg aaattttctt | | | |  |
| 11041 ttctgattat aaaacaatgt gtgctcatta taggaagttt ggaaaacaca gaaaggaaaa | | | |  |
| 11101 atgaagaaag gaagaaaatg taaaactcta cacataggta gcccagattt ctctatagtg | | | |  |
| 11161 cctagacatt tcccaagtgc tttactgggc agcatcactc cagaaactgg gaaatggcta | | | |  |
| 11221 agggaagctc tttcccaaag tggtttgaga acactgatcc tagaactgtg ccttcctgaa | | | |  |
| 11281 agcactagat aagtaaaatc tggactgtac cttggataaa ggtatcatgt ctatgtactt | | | |  |
| 11341 gtaaataagt ctgggaatgg gctgagcact gactgcatct cccacttgaa gaatcattgt | | | |  |
| 11401 gcactttaat ggttaaaggc ccttagaaat cctgcagtag agaagctctt ttaaagttat | | | |  |
| 11461 tgaatccagc gttttcggtt ttgttttttt ttttcagtca ctttttattc attaatctct | | | |  |
| 11521 ttctacaaag taggtttttt tttttttttt ttttttttgc cttaagaaaa cttcttgtta | | | |  |
| 11581 tggaaaattt caaacataca caaaaaacag agagaagagt agataaacca ccatgtaccc | | | |  |
| 11641 atgacccaac ttcacaattg tcaactcatg gctacttgag cttcctctaa ttcccaccta | | | |  |
| 11701 cttttcctct catgttattt tgaagcggat tccaaatgcc atattatttc atttgtaata | | | |  |
| 11761 tttcagtata tacctttaga aggtaaggac tctttgtttt ttaaacataa caccaaacta | | | |  |
| 11821 ttacccctgt aagaatttta acaatcattc tttgatctca tcattaaata cctaaacagt | | | |  |
| 11881 gtcccaatca tttcagaaat gtcataaata catgtgtgtg cctttaattt accatttgtt | | | |  |
| 11941 tgaatcagga tccaaataag gtccatccat tgcaatgggt tatctcttga aaatttttat | | | |  |
| 12001 tgtggtaaaa tatacgttaa tatgaacttt attattttag ccattgttca atgtacactc | | | |  |
| 12061 cagtggcatt gactacattc acatgattgt gcaaccatca gcaccatcca tctctagaac | | | |  |
| 12121 tttttatgtt tccccaaatt gaaactctgt atgcgataac tccccatatc cccttccccc | | | |  |
| 12181 cagccactgg caacaacctt tctactttct gtctttgtga atttgactac tctagataca | | | |  |
| 12241 tcacgtaagt ggagtcatac agtatttgcc tttttgtgac tgactcattt cacttggcat | | | |  |
| 12301 aatgtcctcg tttcatccat attgtggcaa gttggttatc ttttatggct cgtttaatcc | | | |  |
| 12361 ataggttctt ccgccatatc ttttcccctt tgctattgaa gaatctgcat tttttgtccc | | | |  |
| 12421 gtagagattc ccatggtctg ggttttgctg actgtatccc catgttcctc tgtcttctgt | | | |  |
| 12481 atttcttgta aatcggtaat tggatgtaga ggcttgaaca ggctcggttt gattttgttt | | | |  |
| 12541 tagggtttta aattttggtg ggagtcaact gcttcatagt tgatgctgag ttctttcatc | | | |  |
| 12601 aggaggcaca taatgtctgt ggtttctctc catctatctt tgatacagct aagatatata | | | |  |
| 12661 tgtattgtaa atcaaagagt atctgagaca agtctcaatc aatttagaca gtttattttg | | | |  |
| 12721 ccaagttaag gatgtacttg tgacacagac tcaggaggtc ctaacaacat atgcccaagg | | | |  |
| 12781 tactcagggc acagtttggt tttatacatt ttagggagac atgagacatc aatcaatata | | | |  |
| 12841 tgtaagatgt acattgcttc agtccagaaa ggcaggacaa cccaaagtgg gagggggttt | | | |  |
| 12901 ccaggtcata ggtaggtgag agacaaatgg ttgcattctt ttgagtttct gattagcctt | | | |  |
| 12961 tcactgaata tgcaatttgc aggaatagtc acttatgctt tagtctggct tagtgaaaca | | | |  |
| 13021 acagagcaaa ggaaacaacc atatatacat ttgttccatg tgagcagagg gatgactgag | | | |  |
| 13081 ttctgcctgt cctttgtcca caaaaacttc cttgtgggca aattatgaga gagatttgtg | | | |  |
| 13141 gctttttttt ttttttttta aatttttgta gctatcttat ttaggaatag gatgggaaac | | | |  |
| 13201 acatttgtcc tacatggttc ccagcttgac ttttcccttt ggcttagtga ttttgaggtc | | | |  |
| 13261 ctgagattta ttttcctctc acagtataga tatatagata tttagagttt ctttagtttt | | | |  |
| 13321 tatttttaaa taatttcaaa cttgtagaga agtcggaaga atcatacaaa gaactctcat | | | |  |
| 13381 gtacacctta cctagattca ccaatttgta acattgattg tgccacattt actttattgc | | | |  |
| 13441 tctctccata tgtgctgtgg gtataattat tattgttatt attttattta tttatttatt | | | |  |
| 13501 tatttttgac atggagtttc cctcttgttg cccaggctgg agtgcaacgg cgcgatctcg | | | |  |
| 13561 gctcactgca acctctgccc cttaggttca ggcaatcctc ctgcctcagc ctcccgagta | | | |  |
| 13621 gctgggatta caggcatgca ccaacacgtc tggctaattt ttatattttt agtagagacg | | | |  |
| 13681 gggtttcacc acgttggcca ggctggtctc gaactcctga cctcaggtga tccacccacc | | | |  |
| 13741 tcggcctccc aaagtgctag gagtacaggc gtgagccacc gcacccggcc tattatattt | | | |  |
| 13801 tatttttgag actgagtctc actctgtcgc ccaggctgga gtgcagtggt gcgatcttgg | | | |  |
| 13861 ctcactgcaa cctctgtctg ccgggttgaa gcaattctcc tgcctcagcc tcctgagtag | | | |  |
| 13921 ctgggactac tggcaggcgc caccacacct ggctaatgtt ttgtattttt agtagagaca | | | |  |
| 13981 gcgttttgct gtgttggcca ggctggtttc aaactcctga ccccaagtga tccgcccgcc | | | |  |
| 14041 ttgccctccc aaagtgctgg gattataggc ttgagccacc gcacccagct ctgtataatt | | | |  |
| 14101 atttttattt cagaactatt tggaagtatg ttgcacatgc attattactc tttaccccat | | | |  |
| 14161 tatacttcag tttatttcct aagaacaata atattatcta aatatccata gtaattaaat | | | |  |
| 14221 tcaggaaact taatatacta gatacaatgc ttgtattcaa tctgtagttc atattctctt | | | |  |
| 14281 tttgttaatt gtcacaataa tgttctttct agcacctttt tttttcctgg tacaggttcc | | | |  |
| 14341 agttcagaat catgcttcct atttatttgt catcttcctt tagaattctt taatctggaa | | | |  |
| 14401 caatcctttg gccttctttg tctttttatg ccaatgatat tttttaagaa tataggctaa | | | |  |
| 14461 ttactttgtc aacattcctc aatttgggtt tctattatgt gtcttcacag ttacattcag | | | |  |
| 14521 gttgtgtatt tttggctgga ccgctatata aatgtcctcc tcaggatatc acgtcaagat | | | |  |
| 14581 atgtatgatg tttgtttgca cccttattgt tgatgttaat tttttgttgt tggtggtagt | | | |  |
| 14641 aagtatgtaa ggcttttttg ttggtgatgg tagtaagtat ggaggcgtgt tttcagagtc | | | |  |
| 14701 aaccaaagtg gaaataatag ttcaaagagt aaatgtttct gtaaattaat tttagtgttt | | | |  |
| 14761 taaggtatca ttttgtactt cctaccagca atgtttgaaa gtgcctgttc ctccacagtc | | | |  |
| 14821 ttatgaaatg tgtgcaaccc ttctggagtc ttttgcttct agctgcataa agggagaaaa | | | |  |
| 14881 gggagggagc caaggatcat gtgggagatt ttagggatta ggcctggaag tagcattcct | | | |  |
| 14941 taattgttac tctcaacatg tcattggcca cgactagtca tatggcccca acctagctgc | | | |  |
| 15001 atgggaattg tagtctttga ctatatttag gttcacctta ctctctttca aaggctgtgt | | | |  |
| 15061 agattctgtt atttttatat actgtaattt attaatcctc atcctattga ttggcattta | | | |  |
| 15121 ggtacttaca agtttgtttg tttgttgtta taaacagtgt ttatgtatgc ctgtgtctaa | | | |  |
| 15181 agaaaatttc ctagaagtag acagctgggt tggaaaatat gtgtatttta acctttgata | | | |  |
| 15241 aattttgcca aattgttctc caaaaagttt gttcctgttt tgcttccatc aacagaatat | | | |  |
| 15301 gagagtgtaa gtttcactgt tctcttctta atactacagt atgaatttgt aaaacttttg | | | |  |
| 15361 caatttggta gtttaaatta ttttgggggt aattgtattt cctaattaca aaagaggtgt | | | |  |
| 15421 agtgtctctc aatgtttttt agacatttta ataatttcta ttaatatcat ttggccattt | | | |  |
| 15481 atatagtgga tgttcatctt tttcatattg atttgaaaca actttgtata ttaaagattt | | | |  |
| 15541 tcagctcctt gtaggtgttg aaaatatttt tctttttctt tttttttttt tttttttgga | | | |  |
| 15601 gacggagtct tgctctgttg cccaggctgg agtgcagtgg cgcgatctcg gctcactgca | | | |  |
| 15661 agctccacct tccgggttca cgccattctt ctgcctcagc ctcctgagta gctgggacca | | | |  |
| 15721 caggcgccca ccacgacgcc cagctaattt tttgtatttt tagtgagaca gggtttcact | | | |  |
| 15781 gtgttaacca ggatggtctc gatcctcctg accttgtgat tcgcccgcct cagcctcccg | | | |  |
| 15841 aagtgctggg attacaggcg tgagccaccg cgcccggcct gtaaatattt ttcttttaac | | | |  |
| 15901 tttttcattt gggcaggagg cagtagcata ttaagaaagt aatggatcca ctgctctgaa | | | |  |
| 15961 aatttatatg ttttgtttca gatcattatg tagaagttct ggaatgcaaa atacagtgtg | | | |  |
| 16021 aagagaacct caccccagtt ataggaggct atccggttga gaaatttgtg gctaccatgt | | | |  |
| 16081 atcattactt gcagtttgcc tattataagt gtaagtaatc ttctgtgaca tcttgggtga | | | |  |
| 16141 ggcacttagt ccttctaaga ctgtaaaata gaggtaataa ctcttagcta cggttgttga | | | |  |
| 16201 gagttcatca aggtccactg acaaccctgg tgctatgctt tgcacaaaaa ggcagcatgg | | | |  |
| 16261 cttagtgatt cagggcagag cctgtaacca cagtgcctgg gttcgatttc agccttatca | | | |  |
| 16321 cttactagct tctctgtgct ttagtttcct cattgtgaat agagattgta gtactaacct | | | |  |
| 16381 cataggattg tcctgaggag taaatgagtt aatagatgtg aaatgtctgg ggtattccca | | | |  |
| 16441 cactggcaca tagcaaactg tgtgtttgtt tttgttatca ttatttccct tcctccttat | | | |  |
| 16501 cactagcttt acttttcaga ctgttagagt ttattttctt ttcttttttc tttctttctt | | | |  |
| 16561 ttcttttctt tttttttttt tttttttttt tttgagacag agtcttgctc tgttgcccag | | | |  |
| 16621 gctggagtgc agtggtgtga tcttgactta ctgcaacctc cgcctcccag gttcaagtga | | | |  |
| 16681 ttccccttcc tcagcctcct gagtagctgg gattacaggt gtgtgccacc acgcctagct | | | |  |
| 16741 aattttttgt aattttagta gatatgtggt ttcactatct tggccaggct agtcttgaac | | | |  |
| 16801 tcctgacctc aagtgatcca cccacttcag cctcccaaag tgctgggatt acaggtgtga | | | |  |
| 16861 gccactgcgc ccgtcctgtt agtttcattc tagttggtcc tcccttgatc tgaaatggga | | | |  |
| 16921 gtttctcaat cagaacgttc tagtggacaa tgggaagcaa acgtgggaga gaaggaagtt | | | |  |
| 16981 cagggttaga cttcagagcc tgcgtgtttc ggcttttcct cctctccctg tacctgactt | | | |  |
| 17041 tgccaagtgc cacttgttca agcagtgtgt cccttggcag ttcagttcca agcacagaag | | | |  |
| 17101 aattattagg gaaagtggca gtaggacttc ctctgcccta atgaaatgcc agggtctagc | | | |  |
| 17161 ggtggcaggt ttctggctag aatagatccc ctgggtcgcc ccttcacact caggagtggc | | | |  |
| 17221 tggtctcagg gtgccttggg aagagtgaga gaggtgatct tggctttagc cctcctttcc | | | |  |
| 17281 cactgtggta attgtgtttt ttttttgcgg gggggggttc ctctttatct cacctcccat | | | |  |
| 17341 cctcttcaga acacgtaagc cttgcaccct gaaggtcaga aaaagaaaaa gaatatgtat | | | |  |
| 17401 tatataaata taaaaagcca taaggtcttc tagctcactg agcatggctt ggttctaaat | | | |  |
| 17461 gtataaagct agaacactag gaatttagaa gtgttctgga tgcatgcggg ctctggcagc | | | |  |
| 17521 tccctgggcc tctgaaatcc tcacgtgccc tgaaaatcgc cctgcatatc agaaactggc | | | |  |
| 17581 aaagacttat caacactggt ggactgaggc ctctagctat gcttgctgct tcccaggaaa | | | |  |
| 17641 tctgctttcg gggatcaaca gtatgaatga agtgtacttc ctgaaatgag cttcctgaaa | | | |  |
| 17701 atctgccccg ttttgaattt tttctagacc acctaccatg ctacattgct tgagggcagg | | | |  |
| 17761 tggtattatt tatatttgaa gagtaaatgt ccctaagagc tggctttaaa gtagtcatta | | | |  |
| 17821 caggaaacag ccactgggtg gcacaattta ttcataccca agctttggct tctccggcca | | | |  |
| 17881 accacagctt tagcaaaggt actgttgtgt aaattagagt ggaatgtgcc agaatacata | | | |  |
| 17941 ccaggggctt gtgtctcatc acctcttcct catcctgcac tcccaagaca aatcccaagc | | | |  |
| 18001 cctgcaaaat ccctatgctg aaggtactgt cacaggtggg caacaccaag cagagaggct | | | |  |
| 18061 ggaggtggga gttccaacct caggaggtgg caaaactagg cgactttcca aatcccctcc | | | |  |
| 18121 aacccagagg ctccaccatc ctttgggttt tccaggtctg gtttagctgt aggggagcta | | | |  |
| 18181 acctagatct ggagactgtg gtcccatcca gatgggatcc agacactcta ggacccagat | | | |  |
| 18241 gagacacctg tttgctgagc acatcctccc tatctccctc agccccagga caatccagct | | | |  |
| 18301 ttttccagct gcttccaggg acctgagacc agctcaggtc accagagctg gaagtggaac | | | |  |
| 18361 ataggcattt ctccataggc atgtgcctaa ctagaactaa gtttcaagcc tggcctccag | | | |  |
| 18421 gctgccgtgg agaccccatc tgtggcctgc ccacccaggg ctgccagtcg gccccaggct | | | |  |
| 18481 ctctgaagaa ggactgtgga gaaggggctt gttcatatgg cctttttgtt tagaagcaga | | | |  |
| 18541 gaaattatag ggtgtggttt gctgatttct tcatttgtct tttcttccca accctagtga | | | |  |
| 18601 acgacctgaa gaatgcagcc ccctgtgcag tcagctatct gctctttgat cagaatgaca | | | |  |
| 18661 aggtcatgca gcagaacctg gtgtattacc agtaccacag ggacacttgg ggcctctcgg | | | |  |
| 18721 atgagcactt ccagcccaga cctgtaagtc tggtgcacca caccttccct tttctcttca | | | |  |
| 18781 ttcttgcctt ctggagacta cctcgtgcct taagagaaag ggttattttg gaaataaagg | | | |  |
| 18841 ggctgggcgc ggtgactcat gcctgtaatc ccagcacttt gggaggccga ggcgggcgga | | | |  |
| 18901 tcatgatgtc aggagttcga gaccagcctg gccaacacgg tgaaacccca tctctactaa | | | |  |
| 18961 acatacaaaa attagccagg cgtggtggca cgtggctgta gtcccagcta cttgggaggc | | | |  |
| 19021 tgaggcagaa gaatcgcttg aacccagggg cagaggttgc tgtgagccga gattgcgcca | | | |  |
| 19081 aaaaaaagga aatcaaggaa aagtcatttt tttctgtagt tttgtgcatg tgtgtgtttt | | | |  |
| 19141 aacattttac tgcttcattc tgatttctga atctgtaaaa actctacaaa tttcaaacat | | | |  |
| 19201 tacagaaata ttttactttg aaaacaaaat tctctcataa tcctacacct tctttctctc | | | |  |
| 19261 tcggaaaaca aaattctctc gtaatcctac accttctctc tctctttttt tttttttttg | | | |  |
| 19321 agacagagtc ttgctctgtc acccaggcta gagtgcagtg gcgtgatctt ggctcactgc | | | |  |
| 19381 atcctctgcc tcctgggttc aagcgattct cttgccttag cctcccgagt agctgggatt | | | |  |
| 19441 acaggcgtgc accaacatat cacactaatt tttgtagttt tagtagagat ggggtttcgc | | | |  |
| 19501 tgggctagtc tcgaactcct ggcttcaggt gatctgccca cctcagcctt tcaaagtgct | | | |  |
| 19561 gggatttaca ggcatgagcc actgcgccca gcctgtgcca tctctttatc tcctgcttta | | | |  |
| 19621 aaggtatata tttactcata tatatagtaa atatttgtgt atttttataa aaacaaggtc | | | |  |
| 19681 atatttacat agttttacaa ctttttgcaa ctttgtcttt ctgacaattt gtcgtgaata | | | |  |
| 19741 tttctccatg tcacatccat gtatcatctt aattattatc ctttaacagc tccctggtag | | | |  |
| 19801 tctcttttgc ggatggacca tcatttaatt cccttgatag gcaaactcac cacttagcat | | | |  |
| 19861 gtgttgtcag tgaaccccat tcattatata ttactttcaa aagatttttt ctaattgatc | | | |  |
| 19921 tttgctcttg tattttcatt gggatttcta tatgccatct tgattttcat aagtttgtct | | | |  |
| 19981 tccactgagg gctggccact cttttttaaa aattgagata taatttatat aggatagaat | | | |  |
| 20041 tcaccttttt aaagtgtaca attcagtggt tgttagtata ttcagagttg tacagccatt | | | |  |
| 20101 accactatct aattttaaac attttcatca ccttagaaaa aaccccatat cctgttcctc | | | |  |
| 20161 cctcctccca gttctaagat gaaaaggttg gtcctataaa ctgttctctg ttgtgtctga | | | |  |
| 20221 acaggaagca gttcagttct ttaatgtgac cacactccag aaggagctgt atgactttgc | | | |  |
| 20281 taaggaaaat ataatggatg atgatgaggt aagttttcat gcttagcaca tgtctggtgg | | | |  |
| 20341 ctacgagaaa atattactca catctttgct agaatcactg gaaggctgag tcctccctga | | | |  |
| 20401 agtaagaaac agaggggacc ttgagaaaga cagttcctgt gggtgtttca gtgacagtca | | | |  |
| 20461 gaatgctggc atgggaaaga gcggctttct aggaaaccta ggcaggagtg ccacatttcc | | | |  |
| 20521 tgatagcgaa acgggtgtgg ggccacactc caggaattca cagtggtcat tcttcatctt | | | |  |
| 20581 ttcactttat ataaaaacag tgaattttta attgctcagc actctgaaga gacatggata | | | |  |
| 20641 ctcacacccc aatttttctg ctagactatt gctattatta ttttttctaa attgcatctt | | | |  |
| 20701 tactgtaagc attttgtcat tctctagaat tgcttgaaat tcctgattcc accttactca | | | |  |
| 20761 gctgacccct tttctgaccc tgactcaact ggttatggag tggtcatttt gtgatgagtc | | | |  |
| 20821 tggtcacaga aaagccatca ttagcggtag tatttagagt atttgtttgg aacaaactgg | | | |  |
| 20881 aaaatgctta ctgagaaagc tggggacttg tcagctattg gagctcgaag agagctgtga | | | |  |
| 20941 gcttgctctg gtctgatcct tcgctttata gatgaggagc atggggccag caagtttcct | | | |  |
| 21001 gagcctccag gtctgctctt cctatacccg ttttaggtga agtacctctg aataagacat | | | |  |
| 21061 cttcgtggaa tttgttttct gtctataaat atgtatggga tgaactcatc actggcatta | | | |  |
| 21121 aacaactgag tctcagttct ttatctataa aatgggaata ataatactgc cctcccaggg | | | |  |
| 21181 ttgtgagaat aaaatactgt gttgcatgtg gaaatgcttt ctaaactgga aagtgatgca | | | |  |
| 21241 atgcctatgt cttggtttgg gtatccttga agcagagcct gagacaggat cccaagatgc | | | |  |
| 21301 cacaggggcc attcccccat tccctaaagg aaggaacacc agccagatac caacaagatt | | | |  |
| 21361 ggccactgtg gtttacaaag tatagcagag gctgggtgta gtggctcaca cctgtgatct | | | |  |
| 21421 cagcatttgg gaggccaaag caggtgattg cttgagacca ggagttcaag accagcctgg | | | |  |
| 21481 gcaacatggt aaaacctcgt ctctacaaaa aatacaaaaa aattagctgg gtatggtgga | | | |  |
| 21541 gtgcacccgt agtcctagct acttggaagg ctgaaggagt ctggggagtt tgaggctgat | | | |  |
| 21601 attgcgccat tgcactccag actcggcaat tggagtaaga ccctgcctca aaaaaaaaaa | | | |  |
| 21661 aaaggaaagt atagaaaaat atatttagaa aaggtaagag aagttgagtt cccagttatc | | | |  |
| 21721 tagaggcaag ctaatatgga tattttggta tatatctttc cattttttta ttattaaaga | | | |  |
| 21781 aaaaatttta tagttttaaa tttgtttttc tacatattta ttataaactc agctataatt | | | |  |
| 21841 atgtctcatg actttttcac tttatattat tatgtgagca gtttctcttg ttatattttt | | | |  |
| 21901 ctaaaacagc tttatttaga tattattcac aaaaaatgaa attctccttt ttaaagtata | | | |  |
| 21961 caattcagtt gtacttgtta tatccacaaa attttgcatc catcactgtt atttaacttt | | | |  |
| 22021 agaacatttt tattgccccc aaaagaaact ccatagctaa gtcacttccc atttctcctc | | | |  |
| 22081 tgggccaaca ttcccactcc tgcctccacc tccagcccct ggaagactct aatctacttt | | | |  |
| 22141 ctgtttctgt ggatttgcct gttgtggaca tttcatataa atggaaatat agggtatgtg | | | |  |
| 22201 gtcttttgtg actggcttct ttcactgagc atatgttttc aaagttcagc catgttgaac | | | |  |
| 22261 atgttgcctg taaaatttcc tctgtactac caagtaatat tcttcttttg gatgtaccgc | | | |  |
| 22321 attctgttta tccattcttc agttgatgga tgtttgggtt ggttccactt tttggctatt | | | |  |
| 22381 ttgaataatg atgctatgac catttgtgta caaatttttg tttgaacata agctttccag | | | |  |
| 22441 ttttttatac ctaggagtat atacataggt attatgttag gccatttttg catcactgta | | | |  |
| 22501 aagaaatacc tggctgggtg cagtggttca tgcctgtaat cccagcactt tgggaggcca | | | |  |
| 22561 aggcaggagg attgcttgag cccaagacta gcctgagtaa catagtgaga ctctgtctct | | | |  |
| 22621 acagaaaatt taaaagttag ctgggcatgg tggcacagga agtgtggcaa tggcatctgc | | | |  |
| 22681 ttggcttctg gtgaggtctc aggaagctta tggtcatggc aaagggggag ctggcgtatg | | | |  |
| 22741 tcacatggca agagcatgcc acactctttt aaacaactag atcttgtgtg aactcagagt | | | |  |
| 22801 gagaactcac ttattatcat gaggacagca ccaagacatt tttgaggcat ccgcccacct | | | |  |
| 22861 gacccaaata ccttccacca ggccccactt ccaacaccgg agattacatt tcaacatgag | | | |  |
| 22921 atttggaggg gacatatatc caaaccatat caggtacata cctaggaatg gattgctggg | | | |  |
| 22981 ccatatggta atggtttaac ctgttgagaa actgccagac tgtttttcaa agtggctgca | | | |  |
| 23041 ccatttacat tcccatcagc agtgtatgag agttccaact ttgcaatatc ttcaacaaaa | | | |  |
| 23101 cttaatattg tctttttttt ttaatagcca tgctagtgga tgcaagtagt atttcattgt | | | |  |
| 23161 ggttctgact tccactttct gttgtttttc ttttcttttt gagacagagt tttgctcttg | | | |  |
| 23221 ttgcccaggc tggagtgcaa tggtgcgatc tcggctcact gcaacctccg cctcccaggt | | | |  |
| 23281 tcaagtgatt ccctgcctca gccatcctag tagctgggat tacaggcgcg caccaccaca | | | |  |
| 23341 cccagctaat tttttgtatt gttagttgag atggagtttc actatgttgg ccaggctggt | | | |  |
| 23401 ctcaaactcc tgacctcagg cgatccaccc gcctcagcct cccaaaatgc tgggattaca | | | |  |
| 23461 ggcgtgagct actgcaccca gcttgacttg caccttctta gtgactatgt tgagcatccc | | | |  |
| 23521 ttcatgattg gccatttgta tatctttgaa gaaatgtcta ttcaaatctt ttgtccactt | | | |  |
| 23581 taaaattgtt acttatcttt ttattattgt gttgtagaag ttctttagat attctagtta | | | |  |
| 23641 taagttcctt atcagatata tgaattgtag atattttctc acaatttgtg ggttgtcttt | | | |  |
| 23701 tcagttgcta aatggtgtct tgaagcacaa gttttcaaat ttgatgaaat tcaatttatc | | | |  |
| 23761 tgttctttct tttgttgctt ttgtgttgtg tgtcatatct aagaaaccat tgcctaatcc | | | |  |
| 23821 aaggtcacaa agatttactc ctgattttta ctcctacgat ttttgtaatt ttaactcctg | | | |  |
| 23881 catttaaatt tacaacccat ttgagttaat tttatgtgtg atattaggaa gaggtctagt | | | |  |
| 23941 ttcattctct tgcatgtgaa tagccagttg ttacagcact atttgttgaa aagacttttt | | | |  |
| 24001 caccattgaa ttgtcttggt accctttgaa agaaatcagt tgaccataaa tgtgagagtt | | | |  |
| 24061 aattttccag actctcagtc tataccactc atcttgattt ctatccttaa agttagtact | | | |  |
| 24121 acactgtctt cattaatata gctttttttt tttaaattac gtggactttc aaccaagatt | | | |  |
| 24181 agattactgt agctttatac tatattttga aatttgaatt gtgagtcctc caacgttgtt | | | |  |
| 24241 cttcttttcc aagatgattt tggctatttt gggtcctgta catttccata tgaattatag | | | |  |
| 24301 gtttagcttt tcagtttttg caaaaggcag ctgggatttt tttataggaa ttccactgaa | | | |  |
| 24361 cacataaatc tctttgggga gtattggctt cttaatattg tcttctaatc catgaacatg | | | |  |
| 24421 agaagtcttt ccacttattt ggatttttta aaaatttatt tcacagtgtt ttcttatttt | | | |  |
| 24481 attattccta aacattctat tcttttttaa aaaattaaaa taaattcttt ccaaccccta | | | |  |
| 24541 gtattcaagc attttattct ttttgatgct attataaatg aaattgtttt cttattttca | | | |  |
| 24601 cttttgggca tattctaaaa gaatagaaat acaatagatt ttgtgtattg attctgcaaa | | | |  |
| 24661 cttgctgaac ttatttatta gtttgtatgt gtatgtgtat tatttttctt gcgtaattgt | | | |  |
| 24721 tctagttaaa acttctagta ctatgttgaa tagaagcaga gagagtgggc ctggcacagg | | | |  |
| 24781 ggctcattcc tctaatccca gcactttggg aggctgaggc aggaggatca cttgaaccca | | | |  |
| 24841 ggagtttgag accagcctgg gcaacatagc aagaccccat ctctacaaaa aataaaaaaa | | | |  |
| 24901 ttaggctggg tgtggtggct cacacctgta atcccagcac tttgggaggc tgaggtgggc | | | |  |
| 24961 agatcacctg aggtcgggag tttgagacca gcctggccaa tcggtcgaaa tcttgcttta | | | |  |
| 25021 ctaaaaatac aaaaaaatta gctgggcgtg gtggcacatg cctgtaatcc cagctactca | | | |  |
| 25081 ggaggctgag gcaagagaat cgcttgtacc caggaggtgg aggttgcagt gagccgagat | | | |  |
| 25141 cacaccactg tactgtagcc tgggtggcac agtgagacac tgtctaaaaa aaaaaattag | | | |  |
| 25201 ctaagtatga tggtgcacat ctgtaatcct agctactcag gaggctaaag tgggaggatt | | | |  |
| 25261 gctggagctc aggagtggga gggtacagtg aaccataagc atgccactgc acactaggct | | | |  |
| 25321 aggtgacaga gcaagaccct gtctctctgg aaaaaaaaag aagtggagag agtgaacatc | | | |  |
| 25381 cttgtcttgt tcccagtctt aaaaggaaaa agcatacagt cgtctttcag cattaaatgt | | | |  |
| 25441 gaaagtacgt tattagctgg gggttttttg taggtgccct ttattaggtt gaggaagttc | | | |  |
| 25501 tatttttaat tcgttgaatt tttttttttt ttttttgaga cggagtttca ctcttgttgc | | | |  |
| 25561 ctaggctgga gtgcagtggt gtgatctctg ctcactgcaa cctccgcctc ccaggttcaa | | | |  |
| 25621 gtgattctcc tgcctcgccc tcccaagtag ctggaattac aggcatgtgc caccacaccc | | | |  |
| 25681 gggtaatttt tctgtattta gtagagacgg gggtttcacc atgttggcca gtctggtctc | | | |  |
| 25741 gaactcctga cctcaggtga tcaacttgcc ttggcctccc aaagtgctgg gattacaggc | | | |  |
| 25801 gtgagccacc gcacccggcc tgaatgtttt tatcatgaat ttgtgttgaa ttttgtcaaa | | | |  |
| 25861 tgctttttct gcatctattg agatggtcac atgatttttg tttcccttgt taatattcat | | | |  |
| 25921 tataaaaatg acttttttgt gactaatatt acatgatata gataatttta agaaatatag | | | |  |
| 25981 cctttttgta ggcatttagt ttgcttccaa ctttttgctt ttacaaatag catagcaggg | | | |  |
| 26041 actaaattgt ttacttcttc tctaatcatt tccttagaaa aaattcccag tggtgaaatt | | | |  |
| 26101 cctggcctaa ggctgtgaac attttcaaga cttctgatgt acaagaacaa catatataga | | | |  |
| 26161 aagcaaatca tagatactag aacacgaaga gatttttcac atgattcagt aaagctctct | | | |  |
| 26221 catgtcacag ataaggaaac aaactcagaa aggcagagtg gcttgctcat agtcacacag | | | |  |
| 26281 agggttagta atcatctaat gaacacgcca agcttattgc aggctttaaa attcctagga | | | |  |
| 26341 ttcttggatt tggcactttc aagataaaag gactttgttg aaggttctgg agtagtttgc | | | |  |
| 26401 ttattaattg aggctttaga attgtggaag aactaatcca tctctaaaga atgaaccatt | | | |  |
| 26461 ttcttactat ataccaaatt atggcaagta atactttgaa atgaaaatat aaccaaaatc | | | |  |
| 26521 atcaaattct tagttagtcc ttccattttc caaaggacat aatactcctt tgaaacaaag | | | |  |
| 26581 catcagtaca ctttagctgc tgatatgtgt ggcattcttc cttattatct ttctggtgtt | | | |  |
| 26641 gaaatgtgta taagcactcc tgaaagttta cgcactggtt cagaattttg cagaggttta | | | |  |
| 26701 ttacagtttc agaaactttt taaaacaggc tgaaacagat gcttcaggtt tcaagttctg | | | |  |
| 26761 atggttgttt gcttcaggga agtccaaaag aaatagaata taatttgttg gaaggatgta | | | |  |
| 26821 ttgagggagt gctctcaggt aggaacggaa tgagggtcag gggaaggagc tcaacaaata | | | |  |
| 26881 cgtggtctca gctggagtca agcttcgcct gctcccatgg gggactctgg agcatgaatt | | | |  |
| 26941 gcaccacaga gttgttcttg ccttttgtag tcccccttat cagtctgtca ttcgttcttc | | | |  |
| 27001 gttgccagga agggctggtg tgtaacctcc ctgtcaagac tgctgttttt ttttgttttt | | | |  |
| 27061 gtttttgttt ttggccaaag gcaattgccc agagaagggg cagctgctag ccattagcaa | | | |  |
| 27121 gcagccctca caggagttgg ggatgagtgc actgggaagg agatctgagt gaggccccaa | | | |  |
| 27181 cagtattcac tacaacagcc atgcagggga tgcatttctt tcgtctgtgc tgcctcgggc | | | |  |
| 27241 acttattact agtgagctaa aagcaaacaa taccatcatc ccgcaagtca caacagacac | | | |  |
| 27301 acagcaggat atagtaactc cctaagcaga agtcatagct atacacataa tccctctact | | | |  |
| 27361 atgctgtgtt ttccttttgg ggtctagaaa gcaactgaaa aacaattttt ccaagctgta | | | |  |
| 27421 gtgtctacag tttgagtttc taatctcagg catgacatag tagaagtatt tgatgataat | | | |  |
| 27481 actaagaccc cccatttgtg tctcatgtac ttgataggtt ttccagttat tcccaaatgc | | | |  |
| 27541 tgcaagattt ttataaattt ctctgtgtta aaacatcaaa aggagccaac aggctcttgg | | | |  |
| 27601 cctgggatgg tgagctcagt gaaggaggtc gagttttagc agggttggtg gggccagtct | | | |  |
| 27661 gtgttgactc ttatggaagg ggcctcagcc ttggtcattc actcattctc tgcagaaaag | | | |  |
| 27721 agaatgggtg tcatcagctg gtggtccagg agggttatag aagagaagtc aatgtggaga | | | |  |
| 27781 aagaaattgg gaagggtgct gggctgcttc aagttgaagt ataaggctga ttgcagaagg | | | |  |
| 27841 agggagcagg tgagctcctg gtaccgacac tgggcaagtg gatcccacta tccctgtgtg | | | |  |
| 27901 tctctcctgt aagaggagtg attgcttcag tcacaccagc agtatgtatg caagcactca | | | |  |
| 27961 atcattcatc cttcaacaca tgtattgagc accttctgtg tgctggactc tggggaagaa | | | |  |
| 28021 aaatcaaccc agtcccctgg tccttgagaa gtttgggttc tggtggtcaa ggcagaaagg | | | |  |
| 28081 gaacagatag aaaatgttcc tgtgggagac ctgcacacat gagaggggag gaaggggagt | | | |  |
| 28141 aggcagagat gcacacagga gggccactta gtagagctgt tcacaggctg ggctgtccgc | | | |  |
| 28201 ctcacacagc atgttggcgg atctcgcaga gcacttgata ccaagttcaa atggagtgga | | | |  |
| 28261 agccgaggtc ccctccacag ggagaaatca ggtcctctgg gccccagacc agcagaaaaa | | | |  |
| 28321 acctgtttca gccaaagcag acagtggtga tggcctctcg ggatactgtt tctgctcatc | | | |  |
| 28381 agtactttta aaagtccaat agcccattta ataaagttgt cctgttgtct cttacaggga | | | |  |
| 28441 gaagttgtgg aatatgtgga tgacctcttg gaactggagg agaccagcta gcccacagca | | | |  |
| 28501 accaaagaga cttcctcttg gcgttcagga aacacagatt ctttgtcctt ttcccaacag | | | |  |
| 28561 cccaggctgt tgatacctca gagccttctc tttactctcc aaagtgaaag ggaagccccc | | | |  |
| 28621 gtctctctaa ctgcatgtca tcaggggtga gcctgccttt cctatcttca cacctgccac | | | |  |
| 28681 ctcatgttca cacctatctt tctcaccttt tttttgagat ggagtctcgc tctcttgccc | | | |  |
| 28741 aggctggagt gcaatggcac gttctcagct cactgcaacc tccgcctctt gggttcaagc | | | |  |
| 28801 aattctgctg catcagcctc ccgagtacct gggattacag gcatgtgcca ccacgcccgg | | | |  |
| 28861 ctaattttgt atttttagta gagacggggt tttgccatgt tggccaggct ggtctcgaac | | | |  |
| 28921 tcttgacttc agatgatcca tctgccttgg cctcccacag tgctgggatt acaggcgtga | | | |  |
| 28981 gccaccatgc ccggcctctt tctcaccttt acacctgtct tcttatcctc acatctgttt | | | |  |
| 29041 tcacaccttc atccctgtct tcctcatgtt cacacttgtc ttccccatgt tcatagctgc | | | |  |
| 29101 ctttcttacc attttggttt gaagggcagt cttctctggc ttgttttttt gtttttccca | | | |  |
| 29161 gaaaatcagt attatttttt aaataagaaa aacattccta gaagatgata attgtgaaaa | | | |  |
| 29221 cctcctttgg cttatttgct tttccagatt ttagtctcct ttctccccat ccgggaaaga | | | |  |
| 29281 tggtggaaga cataggctaa atttctccag cctcacaatg gtcttcactt ggtctgactt | | | |  |
| 29341 gtaccaattc tagcacccac tgaaaaacaa gttgagtaga gagtgtagag tgcagaaatg | | | |  |
| 29401 tggcttttgc cccactttgc atctccaaaa ttacaacggt tggccgatcc catttgagga | | | |  |
| 29461 caatgcttag ttataagtct ccgagttgga aaaggaagaa agccagagct gtctagtttc | | | |  |
| 29521 attcattctt tcagtaaata tttattgagt acctactgtg tgctaggcat tgacctggga | | | |  |
| 29581 actagagata cttcacagaa taacagggaa agttccctgt gctcatggag cttacattct | | | |  |
| 29641 acagggagaa agagatagcc aatacatagg aataaatata tacaaggtat catgtagtga | | | |  |
| 29701 taattgctgt ggagaaaaat aaagcagggg agggagtaag aaatcctgga gatgaggctg | | | |  |
| 29761 cagttttaaa tggggcctca ctgggaatgt gacgttgagc agagacgtta gggaagtgga | | | |  |
| 29821 tcctggacaa ggcattccag gcagaggaac aggatgtgca ctgccccaaa gtgagaactt | | | |  |
| 29881 gctctacgtg gtcaggaaag agcagggaga ccaagcagag tcgtgggcag gggtagaatg | | | |  |
| 29941 gaaggagagg cggctgggga ggacaggtgg tggagggcct tggcttctgc taagtgagat | | | |  |
| 30001 gggaaccact ggagggtttg aacagaggag tgccttgatt gatttatatt ttgcaagggt | | | |  |
| 30061 cattctagct gccatattgt gaaaaacttt agtggacaag ggcagaagga agagggaaga | | | |  |
| 30121 cctgttagga agctactgca aggttccagg cttgggcctg ggccacagca acagcagtgg | | | |  |
| 30181 tcaaatatct agatttattt tgaaaagagc caataggatt tgctgagagt ttgaatgtgg | | | |  |
| 30241 agtgtaagag aaggaagagt taatgatgac attaaggttt ttggcctgaa tagcaggaaa | | | |  |
| 30301 gatggagtta ccagttactg aaatagggaa ggatgggctg ggtaagtatg gaatttggtg | | | |  |
| 30361 caaagcaggc tgtctgtggt tggaatggga ggttctggct gcaaatcaaa gtggagagtt | | | |  |
| 30421 ctctcaggtc aggtctgcag cagagctcga gacagggatc tgaatgcact tggtttattg | | | |  |
| 30481 ttgggggtgc tctcagaagg aacctgtgaa agcctttatc agtcatttat tggctgtgag | | | |  |
| 30541 aagttctctg ggagtgtggg tacatttgaa ggcaagtgac ttcagttgag ggcaagtctc | | | |  |
| 30601 tggaaaagag gctgtaggca tctggcagct accatgcatg gtagtgtgtt gggggtgggg | | | |  |
| 30661 gtcctgggca ctggctgtgt gaagggatct ggcagggcac cacagcgccc cctactgaac | | | |  |
| 30721 catcagcatg tcagtggcat ttaaagccat gcagctggag gggccactga gattgtctct | | | |  |
| 30781 gagtattact gagaagcaac agaaaagagc catggatgga gcccttgggc tctctgggaa | | | |  |
| 30841 atgggaaatc agccaaagga ctgagaagga gttaccttaa ggtcagagaa aaccaagaga | | | |  |
| 30901 gtgtggtgtt ctggaagctg agctttcttt attcaacctc attcccttct ccaaataagc | | | |  |
| 30961 cacttgtgta gttgggcccc tccagggttg aaggcaagag gagaaaggca cagcgtttgg | | | |  |
| 31021 gaaacaagac ttttcctgca atagcctggg aaggaataaa aggatagagt gtttgggttt | | | |  |
| 31081 ttgtgtaatg gtggttaatt ggggtggaac actcacacgt tgtgcttttt ctgggcttcc | | | |  |
| 31141 cttatccccc agaacactct accaacctcg gggaactcgg gcacatcctt ctgtttctcc | | | |  |
| 31201 ttcagctcta tcctgctttc ctcatccctt ctgacaccac gtcctcactc acctgcacaa | | | |  |
| 31261 gaatccctgc atcaggttct cctttgaggg tacccaccca ggacagtccc ctaccacttc | | | |  |
| 31321 tgtcttgggc tgaagttgcc cacgtccaca aaatctgtac tcccagcggg ggtgtttggc | | | |  |
| 31381 ccgaggagtc agtgttatta ctggtggatg caccgtgtcc acagcagccc ccaatcccag | | | |  |
| 31441 cgatgcgtca gatcttacgt ggcttcctgc tgggggagat ggccttcacc cacgggatgc | | | |  |
| 31501 cgggttctcc tttctttcct caccccaacc tttactccac cagagaaact tccttttgaa | | | |  |
| 31561 ctcagtgggg aagagggtga tgagacagga ctagaaagta gtgggggacc cagcgagtgg | | | |  |
| 31621 acgccctgct ccgggattcc tgagtctgta aatagtgtgc ccagcagctg tgaactcccc | | | |  |
| 31681 ttatagcctc aggctgcagt gtccttccca gctgtgtgag aaaatgaaag ccgacgtcca | | | |  |
| 31741 cagggaccca ggcagggttg ggtgttgtga ctcactccac ctctgtgccc tgcagaggta | | | |  |
| 31801 ctgttgggtc cttgtcttgt gagcctgggg tgagctctct gtacatgttg ttgttccacg | | | |  |
| 31861 tatgggttga cttggcatgc tggggggtcc tcgttcactc tctgaagttg gcctcctttc | | | |  |
| 31921 actggggatt gaaaagcacc tccaccccta ccctagtgat gtcccctgag gacccgggtg | | | |  |
| 31981 atagtacagt caatattgtc agtactttgc tttgattgaa ggctgtagag ctgagttacc | | | |  |
| 32041 aaaatttcta tttcaaagga aaccaaacct taaaaaaaaa aaacaaaaac tgggctgggt | | | |  |
| 32101 cttccaaacc taccatgaaa ccctggtgtg caggctgcac tcaatgacct caacccaaca | | | |  |
| 32161 cctccctgag tgtgcttctt ggaagagcct agaagattcc tggatggaga ccccattggt | | | |  |
| 32221 tcagcctcaa gtctggcccg tcttcgaaaa aacaaacaca tttgtaagct ttgtgggagc | | | |  |
| 32281 ttccaggcct gctctaagat gccttgcttg tcctttgacc catcagcatg gagctcagtg | | | |  |
| 32341 gttgctgttt ggttctgcag gctggtgggg aggccgccca tcgtggtggg gcatctgtcc | | | |  |
| 32401 agccccattg ccactcaggg catccaaaca ggaggcaccc gctgggaagg gtctaaagat | | | |  |
| 32461 actccttgtg gccactgcta ctgttcacac ttgacttgtg gagaagcgaa gggctgaggg | | | |  |
| 32521 gaggtttgtg tacacccatg tatttaaaag tgactgactg actgaaatga gcacataccg | | | |  |
| 32581 acatatgcaa catactaata ccttcctgat tttcgagact ttctaattac tacaactaac | | | |  |
| 32641 ctgttgtgct cacctctgga attcagaaag agagccactg cgagcactga ccacaagggc | | | |  |
| 32701 tgccttagga aggaaatgtg tgctttcagg agttcttatt aggggaattt taagagcaca | | | |  |
| 32761 aaaattttat tgaacccacc ttagtgacaa acagaaaatg atttgttaga ttgtcagttg | | | |  |
| 32821 gaaggggttt attatgactg ctggtgaaat aaacagtgac cagtttctgc caacatttat | | | |  |
| 32881 ggaaataacg tttctaggtt ttaaatgtga gccgtaaact gaagctactt cagttaaaaa | | | |  |
| 32941 aaaaaaaatc aatacttaaa ctgtagggaa aaggtggatt ggtgccagag aaaacattat | | | |  |
| 33001 ttaatagtgt cagaacatgg aactgcaaca cagtttgtag caaggcactg agaaaaaccc | | | |  |
| 33061 acaactaatc cttcatcgca gctgtctgaa ctcttgatca tctgccatcc cccaaacagt | | | |  |
| 33121 gacttctttt tttctggtga ctccaggcct gaatgaccta gtgtggagag tttaaactat | | | |  |
| 33181 gtacaaggga ggaaagaaaa aaaggaaagg aaccttaagt aagcctcagc caaaggtttt | | | |  |
| 33241 atgcattgga cttcctgtgc ctgcccccag gggcaagact gatccccatg cctgtgccca | | | |  |
| 33301 tgacatctcc ctgaaagagg acaccatgac agcccggctt tgccttgact gacccactgc | | | |  |
| 33361 taccccagaa ataagaatca agagcagcta ttgttatcct tagagtgttt tccgtctagg | | | |  |
| 33421 gccggctcgt gaacagccac atatccttgc acctgacact gtccccacac aaatagctgg | | | |  |
| 33481 ctttcgttgc ttgttgaatg aatgagtgag ttggctctat atccccttgg agctggccgg | | | |  |
| 33541 taagatatta gtgcctcatt ttacaagaag agaataggaa ggaattaagc aatttggcca | | | |  |
| 33601 acagatacaa gatagattcc agagttttat ctcccacttt agggtggcag ccagtaggcc | | | |  |
| 33661 aaactccaaa gaccgttgct gatgtctttt tctgcctccc ctctttgggt tagtgtggta | | | |  |
| 33721 tgtacaagct cactcttgtt gaaaattaga aaatagttga aaacaaaagg tttttgtttt | | | |  |
| 33781 tctttttaaa tcacattaaa tgttttacat tgctta |  |  |  | |
