# Supplementary material for: Development of a High-Throughput Resequencing Array for the Detection of Pathogenic Mutations in Osteogenesis Imperfecta
Source: PLoS One. 2015 Mar 5;10(3):e0119553. doi: 10.1371/journal.pone.0119553 (PMC4350936; doi:10.1371/journal.pone.0119553)
Supplement: S3 Datasets — According to the selected mutations, we have made them into the probes to tiles on the chip. (ZIP) [file pone.0119553.s003.zip › COL1A1.doc]

窗体顶端

| Mutation type | Total number of mutations |
| --- | --- |
| Missense/nonsense | 315 |
| Splicing | 102 |
| Regulatory | 4 |
| Small deletions | 94 |
| Small insertions | 24 |
| Small indels | 6 |
| Gross deletions | 12 |
| Gross insertions/duplications | 2 |
| Complex rearrangements | 2 |
| Repeat variations | 0 |
| TOTAL | 561 |

**Missense/nonsense**

窗体底端

[NM_000088.3](http://www.ncbi.nlm.nih.gov/entrez/viewer.fcgi?db=nucleotide&id=NM_000088.3): c.64G>C; [NP_000079.2](http://www.ncbi.nlm.nih.gov/entrez/viewer.fcgi?db=nucleotide&id=NP_000079.2): p.G22R

CTCTTAGCGGCCACCGCCCTCCTGACGCAC(G/C)GCCAAGAGGAAGGCCAAGTCGAGGGCCAAG

[NM_000088.3](http://www.ncbi.nlm.nih.gov/entrez/viewer.fcgi?db=nucleotide&id=NM_000088.3): c.77G>A; [NP_000079.2](http://www.ncbi.nlm.nih.gov/entrez/viewer.fcgi?db=nucleotide&id=NP_000079.2): p.G26D

CCGCCCTCCTGACGCACGGCCAAGAGGAAG(G/A)CCAAGTCGAGGGCCAAGACGAAGACAgtaa

[NM_000088.3](http://www.ncbi.nlm.nih.gov/entrez/viewer.fcgi?db=nucleotide&id=NM_000088.3): c.182G>T; [NP_000079.2](http://www.ncbi.nlm.nih.gov/entrez/viewer.fcgi?db=nucleotide&id=NP_000079.2): p.C61F

ACGTGTGGAAACCCGAGCCCTGCCGGATCT(G/T)CGTCTGCGACAACGGCAAGGTGTTGTGCGA

[NM_000088.3](http://www.ncbi.nlm.nih.gov/entrez/viewer.fcgi?db=nucleotide&id=NM_000088.3): c.436C>A; [NP_000079.2](http://www.ncbi.nlm.nih.gov/entrez/viewer.fcgi?db=nucleotide&id=NP_000079.2): p.P146T

GGACAGCCTGGACTTCCCGGACCCCCCGGA(C/A)CCCCCGGACCTCCCGGACCCCCTGGCCTCG

[NM_000088.3](http://www.ncbi.nlm.nih.gov/entrez/viewer.fcgi?db=nucleotide&id=NM_000088.3): c.572G>C; [NP_000079.2](http://www.ncbi.nlm.nih.gov/entrez/viewer.fcgi?db=nucleotide&id=NP_000079.2): p.G191A

agGGTCCCTCTGGTCCTCGTGGTCTCCCTG(G/C)CCCCCCTGGTGCACCTgtgagtatccagga

[NM_000088.3](http://www.ncbi.nlm.nih.gov/entrez/viewer.fcgi?db=nucleotide&id=NM_000088.3): c.572G>A; [NP_000079.2](http://www.ncbi.nlm.nih.gov/entrez/viewer.fcgi?db=nucleotide&id=NP_000079.2): p.G191D

agGGTCCCTCTGGTCCTCGTGGTCTCCCTG(G/A)CCCCCCTGGTGCACCTgtgagtatccagga

[NM_000088.3](http://www.ncbi.nlm.nih.gov/entrez/viewer.fcgi?db=nucleotide&id=NM_000088.3): c.580G>C; [NP_000079.2](http://www.ncbi.nlm.nih.gov/entrez/viewer.fcgi?db=nucleotide&id=NP_000079.2): p.G194R

TCTGGTCCTCGTGGTCTCCCTGGCCCCCCT(G/C)GTGCACCTgtgagtatccaggacgtcttca

[NM_000088.3](http://www.ncbi.nlm.nih.gov/entrez/viewer.fcgi?db=nucleotide&id=NM_000088.3): c.589G>C; [NP_000079.2](http://www.ncbi.nlm.nih.gov/entrez/viewer.fcgi?db=nucleotide&id=NP_000079.2): p.G197R

ttctaacccatctttttccttcttctcaag(G/C)GTCCCCAAGGCTTCCAAGGTCCCCCTGGTG

[NM_000088.3](http://www.ncbi.nlm.nih.gov/entrez/viewer.fcgi?db=nucleotide&id=NM_000088.3): c.590G>A; [NP_000079.2](http://www.ncbi.nlm.nih.gov/entrez/viewer.fcgi?db=nucleotide&id=NP_000079.2): p.G197D

tctaacccatctttttccttcttctcaagG(G/A)TCCCCAAGGCTTCCAAGGTCCCCCTGGTGA

[NM_000088.3](http://www.ncbi.nlm.nih.gov/entrez/viewer.fcgi?db=nucleotide&id=NM_000088.3): c.589G>T; [NP_000079.2](http://www.ncbi.nlm.nih.gov/entrez/viewer.fcgi?db=nucleotide&id=NP_000079.2): p.G197C

ttctaacccatctttttccttcttctcaag(G/T)GTCCCCAAGGCTTCCAAGGTCCCCCTGGTG

[NM_000088.3](http://www.ncbi.nlm.nih.gov/entrez/viewer.fcgi?db=nucleotide&id=NM_000088.3): c.589G>A; [NP_000079.2](http://www.ncbi.nlm.nih.gov/entrez/viewer.fcgi?db=nucleotide&id=NP_000079.2): p.G197S

ttctaacccatctttttccttcttctcaag(G/A)GTCCCCAAGGCTTCCAAGGTCCCCCTGGTG

[NM_000088.3](http://www.ncbi.nlm.nih.gov/entrez/viewer.fcgi?db=nucleotide&id=NM_000088.3): c.599G>T; [NP_000079.2](http://www.ncbi.nlm.nih.gov/entrez/viewer.fcgi?db=nucleotide&id=NP_000079.2): p.G200V

tctttttccttcttctcaagGGTCCCCAAG(G/T)CTTCCAAGGTCCCCCTGGTGAGCCTGGCGA

[NM_000088.3](http://www.ncbi.nlm.nih.gov/entrez/viewer.fcgi?db=nucleotide&id=NM_000088.3): c.607G>C; [NP_000079.2](http://www.ncbi.nlm.nih.gov/entrez/viewer.fcgi?db=nucleotide&id=NP_000079.2): p.G203R

cttcttctcaagGGTCCCCAAGGCTTCCAA(G/C)GTCCCCCTGGTGAGCCTGGCGAGCCTGGAG

[NM_000088.3](http://www.ncbi.nlm.nih.gov/entrez/viewer.fcgi?db=nucleotide&id=NM_000088.3): c.607G>A; [NP_000079.2](http://www.ncbi.nlm.nih.gov/entrez/viewer.fcgi?db=nucleotide&id=NP_000079.2): p.G203S

cttcttctcaagGGTCCCCAAGGCTTCCAA(G/A)GTCCCCCTGGTGAGCCTGGCGAGCCTGGAG

[NM_000088.3](http://www.ncbi.nlm.nih.gov/entrez/viewer.fcgi?db=nucleotide&id=NM_000088.3): c.608G>T; [NP_000079.2](http://www.ncbi.nlm.nih.gov/entrez/viewer.fcgi?db=nucleotide&id=NP_000079.2): p.G203V

ttcttctcaagGGTCCCCAAGGCTTCCAAG(G/T)TCCCCCTGGTGAGCCTGGCGAGCCTGGAGC

[NM_000088.3](http://www.ncbi.nlm.nih.gov/entrez/viewer.fcgi?db=nucleotide&id=NM_000088.3): c.613C>G; [NP_000079.2](http://www.ncbi.nlm.nih.gov/entrez/viewer.fcgi?db=nucleotide&id=NP_000079.2): p.P205A

ctcaagGGTCCCCAAGGCTTCCAAGGTCCC(C/G)CTGGTGAGCCTGGCGAGCCTGGAGCTTCAg

[NM_000088.3](http://www.ncbi.nlm.nih.gov/entrez/viewer.fcgi?db=nucleotide&id=NM_000088.3): c.634G>A; [NP_000079.2](http://www.ncbi.nlm.nih.gov/entrez/viewer.fcgi?db=nucleotide&id=NP_000079.2): p.G212R

CAAGGTCCCCCTGGTGAGCCTGGCGAGCCT(G/A)GAGCTTCAgtaagcactctctatacagatt

[NM_000088.3](http://www.ncbi.nlm.nih.gov/entrez/viewer.fcgi?db=nucleotide&id=NM_000088.3): c.635G>A; [NP_000079.2](http://www.ncbi.nlm.nih.gov/entrez/viewer.fcgi?db=nucleotide&id=NP_000079.2): p.G212E

AAGGTCCCCCTGGTGAGCCTGGCGAGCCTG(G/A)AGCTTCAgtaagcactctctatacagattc

[NM_000088.3](http://www.ncbi.nlm.nih.gov/entrez/viewer.fcgi?db=nucleotide&id=NM_000088.3): c.658C>T; [NP_000079.2](http://www.ncbi.nlm.nih.gov/entrez/viewer.fcgi?db=nucleotide&id=NP_000079.2): p.R220X

ttctatcttttctagGGTCCCATGGGTCCC(C/T)GAGGTCCCCCAGGTCCCCCTGGAAAGAATG

[NM_000088.3](http://www.ncbi.nlm.nih.gov/entrez/viewer.fcgi?db=nucleotide&id=NM_000088.3): c.661G>T; [NP_000079.2](http://www.ncbi.nlm.nih.gov/entrez/viewer.fcgi?db=nucleotide&id=NP_000079.2): p.G221C

tatcttttctagGGTCCCATGGGTCCCCGA(G/T)GTCCCCCAGGTCCCCCTGGAAAGAATGGAG

[NM_000088.3](http://www.ncbi.nlm.nih.gov/entrez/viewer.fcgi?db=nucleotide&id=NM_000088.3): c.670G>T; [NP_000079.2](http://www.ncbi.nlm.nih.gov/entrez/viewer.fcgi?db=nucleotide&id=NP_000079.2): p.G224C

tagGGTCCCATGGGTCCCCGAGGTCCCCCA(G/T)GTCCCCCTGGAAAGAATGGAGATGATgtaa

[NM_000088.3](http://www.ncbi.nlm.nih.gov/entrez/viewer.fcgi?db=nucleotide&id=NM_000088.3): c.670G>A; [NP_000079.2](http://www.ncbi.nlm.nih.gov/entrez/viewer.fcgi?db=nucleotide&id=NP_000079.2): p.G224S

tagGGTCCCATGGGTCCCCGAGGTCCCCCA(G/A)GTCCCCCTGGAAAGAATGGAGATGATgtaa

[NM_000088.3](http://www.ncbi.nlm.nih.gov/entrez/viewer.fcgi?db=nucleotide&id=NM_000088.3): c.716G>A; [NP_000079.2](http://www.ncbi.nlm.nih.gov/entrez/viewer.fcgi?db=nucleotide&id=NP_000079.2): p.G239D

ctccctctcagGGGGAAGCTGGAAAACCTG(G/A)TCGTCCTGGTGAGCGTGGGCCTCCTGGGCC

[NM_000088.3](http://www.ncbi.nlm.nih.gov/entrez/viewer.fcgi?db=nucleotide&id=NM_000088.3): c.725G>A; [NP_000079.2](http://www.ncbi.nlm.nih.gov/entrez/viewer.fcgi?db=nucleotide&id=NP_000079.2): p.G242D

agGGGGAAGCTGGAAAACCTGGTCGTCCTG(G/A)TGAGCGTGGGCCTCCTGGGCCTCAGgtgag

[NM_000088.3](http://www.ncbi.nlm.nih.gov/entrez/viewer.fcgi?db=nucleotide&id=NM_000088.3): c.742G>A; [NP_000079.2](http://www.ncbi.nlm.nih.gov/entrez/viewer.fcgi?db=nucleotide&id=NP_000079.2): p.G248R

CCTGGTCGTCCTGGTGAGCGTGGGCCTCCT(G/A)GGCCTCAGgtgagcagggggctgtggctga

[NM_000088.3](http://www.ncbi.nlm.nih.gov/entrez/viewer.fcgi?db=nucleotide&id=NM_000088.3): c.751G>T; [NP_000079.2](http://www.ncbi.nlm.nih.gov/entrez/viewer.fcgi?db=nucleotide&id=NP_000079.2): p.G251C

cccttccttgtcttcttcatctctctccag(G/T)GTGCTCGAGGATTGCCCGGAACAGCTGGCC

[NM_000088.3](http://www.ncbi.nlm.nih.gov/entrez/viewer.fcgi?db=nucleotide&id=NM_000088.3): c.757C>T; [NP_000079.2](http://www.ncbi.nlm.nih.gov/entrez/viewer.fcgi?db=nucleotide&id=NP_000079.2): p.R253X

cttgtcttcttcatctctctccagGGTGCT(C/T)GAGGATTGCCCGGAACAGCTGGCCTCCCTG

[NM_000088.3](http://www.ncbi.nlm.nih.gov/entrez/viewer.fcgi?db=nucleotide&id=NM_000088.3): c.760G>A; [NP_000079.2](http://www.ncbi.nlm.nih.gov/entrez/viewer.fcgi?db=nucleotide&id=NP_000079.2): p.G254R

gtcttcttcatctctctccagGGTGCTCGA(G/A)GATTGCCCGGAACAGCTGGCCTCCCTGGAA

[NM_000088.3](http://www.ncbi.nlm.nih.gov/entrez/viewer.fcgi?db=nucleotide&id=NM_000088.3): c.761G>A; [NP_000079.2](http://www.ncbi.nlm.nih.gov/entrez/viewer.fcgi?db=nucleotide&id=NP_000079.2): p.G254E

tcttcttcatctctctccagGGTGCTCGAG(G/A)ATTGCCCGGAACAGCTGGCCTCCCTGGAAT

[NM_000088.3](http://www.ncbi.nlm.nih.gov/entrez/viewer.fcgi?db=nucleotide&id=NM_000088.3): c.769G>A; [NP_000079.2](http://www.ncbi.nlm.nih.gov/entrez/viewer.fcgi?db=nucleotide&id=NP_000079.2): p.G257R

atctctctccagGGTGCTCGAGGATTGCCC(G/A)GAACAGCTGGCCTCCCTGGAATGAAGGGAC

[NM_000088.3](http://www.ncbi.nlm.nih.gov/entrez/viewer.fcgi?db=nucleotide&id=NM_000088.3): c.787G>A; [NP_000079.2](http://www.ncbi.nlm.nih.gov/entrez/viewer.fcgi?db=nucleotide&id=NP_000079.2): p.G263R

CGAGGATTGCCCGGAACAGCTGGCCTCCCT(G/A)GAATGAAGGGACACAGAgtgagtcaccttt

[NM_000088.3](http://www.ncbi.nlm.nih.gov/entrez/viewer.fcgi?db=nucleotide&id=NM_000088.3): c.788G>T; [NP_000079.2](http://www.ncbi.nlm.nih.gov/entrez/viewer.fcgi?db=nucleotide&id=NP_000079.2): p.G263V

GAGGATTGCCCGGAACAGCTGGCCTCCCTG(G/T)AATGAAGGGACACAGAgtgagtcacctttg

[NM_000088.3](http://www.ncbi.nlm.nih.gov/entrez/viewer.fcgi?db=nucleotide&id=NM_000088.3): c.797G>A; [NP_000079.2](http://www.ncbi.nlm.nih.gov/entrez/viewer.fcgi?db=nucleotide&id=NP_000079.2): p.G266E

CCGGAACAGCTGGCCTCCCTGGAATGAAGG(G/A)ACACAGAgtgagtcacctttgagtcattta

[NM_000088.3](http://www.ncbi.nlm.nih.gov/entrez/viewer.fcgi?db=nucleotide&id=NM_000088.3): c.805G>A; [NP_000079.2](http://www.ncbi.nlm.nih.gov/entrez/viewer.fcgi?db=nucleotide&id=NP_000079.2): p.G269S

ttcttaccttctacctctttcctgctttag(G/A)GTTTCAGTGGTTTGGATGGTGCCAAGGGAG

[NM_000088.3](http://www.ncbi.nlm.nih.gov/entrez/viewer.fcgi?db=nucleotide&id=NM_000088.3): c.806G>T; [NP_000079.2](http://www.ncbi.nlm.nih.gov/entrez/viewer.fcgi?db=nucleotide&id=NP_000079.2): p.G269V

tcttaccttctacctctttcctgctttagG(G/T)TTTCAGTGGTTTGGATGGTGCCAAGGGAGA

[NM_000088.3](http://www.ncbi.nlm.nih.gov/entrez/viewer.fcgi?db=nucleotide&id=NM_000088.3): c.814G>T; [NP_000079.2](http://www.ncbi.nlm.nih.gov/entrez/viewer.fcgi?db=nucleotide&id=NP_000079.2): p.G272C

tctacctctttcctgctttagGGTTTCAGT(G/T)GTTTGGATGGTGCCAAGGGAGATGCTGGTC

[NM_000088.3](http://www.ncbi.nlm.nih.gov/entrez/viewer.fcgi?db=nucleotide&id=NM_000088.3): c.823G>C; [NP_000079.2](http://www.ncbi.nlm.nih.gov/entrez/viewer.fcgi?db=nucleotide&id=NP_000079.2): p.G275R

ttcctgctttagGGTTTCAGTGGTTTGGAT(G/C)GTGCCAAGGGAGATGCTGGTCCTGCTGGTC

[NM_000088.3](http://www.ncbi.nlm.nih.gov/entrez/viewer.fcgi?db=nucleotide&id=NM_000088.3): c.824G>A; [NP_000079.2](http://www.ncbi.nlm.nih.gov/entrez/viewer.fcgi?db=nucleotide&id=NP_000079.2): p.G275D

tcctgctttagGGTTTCAGTGGTTTGGATG(G/A)TGCCAAGGGAGATGCTGGTCCTGCTGGTCC

[NM_000088.3](http://www.ncbi.nlm.nih.gov/entrez/viewer.fcgi?db=nucleotide&id=NM_000088.3): c.842G>A; [NP_000079.2](http://www.ncbi.nlm.nih.gov/entrez/viewer.fcgi?db=nucleotide&id=NP_000079.2): p.G281D

GTGGTTTGGATGGTGCCAAGGGAGATGCTG(G/A)TCCTGCTGGTCCTAAGgtaagaggctgtct

[NM_000088.3](http://www.ncbi.nlm.nih.gov/entrez/viewer.fcgi?db=nucleotide&id=NM_000088.3): c.841G>A; [NP_000079.2](http://www.ncbi.nlm.nih.gov/entrez/viewer.fcgi?db=nucleotide&id=NP_000079.2): p.G281S

AGTGGTTTGGATGGTGCCAAGGGAGATGCT(G/A)GTCCTGCTGGTCCTAAGgtaagaggctgtc

[NM_000088.3](http://www.ncbi.nlm.nih.gov/entrez/viewer.fcgi?db=nucleotide&id=NM_000088.3): c.851G>C; [NP_000079.2](http://www.ncbi.nlm.nih.gov/entrez/viewer.fcgi?db=nucleotide&id=NP_000079.2): p.G284A

ATGGTGCCAAGGGAGATGCTGGTCCTGCTG(G/C)TCCTAAGgtaagaggctgtctgaacatcat

[NM_000088.3](http://www.ncbi.nlm.nih.gov/entrez/viewer.fcgi?db=nucleotide&id=NM_000088.3): c.850G>A; [NP_000079.2](http://www.ncbi.nlm.nih.gov/entrez/viewer.fcgi?db=nucleotide&id=NP_000079.2): p.G284S

GATGGTGCCAAGGGAGATGCTGGTCCTGCT(G/A)GTCCTAAGgtaagaggctgtctgaacatca

[NM_000088.3](http://www.ncbi.nlm.nih.gov/entrez/viewer.fcgi?db=nucleotide&id=NM_000088.3): c.859G>A; [NP_000079.2](http://www.ncbi.nlm.nih.gov/entrez/viewer.fcgi?db=nucleotide&id=NP_000079.2): p.G287S

atttctcactcattattctctgatctacag(G/A)GTGAGCCTGGCAGCCCTGGTGAAAATGGAG

[NM_000088.3](http://www.ncbi.nlm.nih.gov/entrez/viewer.fcgi?db=nucleotide&id=NM_000088.3): c.863A>C; [NP_000079.2](http://www.ncbi.nlm.nih.gov/entrez/viewer.fcgi?db=nucleotide&id=NP_000079.2): p.E288A

ctcactcattattctctgatctacagGGTG(A/C)GCCTGGCAGCCCTGGTGAAAATGGAGCTCC

[NM_000088.3](http://www.ncbi.nlm.nih.gov/entrez/viewer.fcgi?db=nucleotide&id=NM_000088.3): c.862G>A; [NP_000079.2](http://www.ncbi.nlm.nih.gov/entrez/viewer.fcgi?db=nucleotide&id=NP_000079.2): p.E288K

tctcactcattattctctgatctacagGGT(G/A)AGCCTGGCAGCCCTGGTGAAAATGGAGCTC

[NM_000088.3](http://www.ncbi.nlm.nih.gov/entrez/viewer.fcgi?db=nucleotide&id=NM_000088.3): c.896G>A; [NP_000079.2](http://www.ncbi.nlm.nih.gov/entrez/viewer.fcgi?db=nucleotide&id=NP_000079.2): p.G299D

CTGGCAGCCCTGGTGAAAATGGAGCTCCTG(G/A)TCAGATGgtgagtgtgcccagttccagagg

[NM_000088.3](http://www.ncbi.nlm.nih.gov/entrez/viewer.fcgi?db=nucleotide&id=NM_000088.3): c.895G>A; [NP_000079.2](http://www.ncbi.nlm.nih.gov/entrez/viewer.fcgi?db=nucleotide&id=NP_000079.2): p.G299S

CCTGGCAGCCCTGGTGAAAATGGAGCTCCT(G/A)GTCAGATGgtgagtgtgcccagttccagag

[NM_000088.3](http://www.ncbi.nlm.nih.gov/entrez/viewer.fcgi?db=nucleotide&id=NM_000088.3): c.913G>C; [NP_000079.2](http://www.ncbi.nlm.nih.gov/entrez/viewer.fcgi?db=nucleotide&id=NP_000079.2): p.G305R

ttctcttggtttggttgtcagGGCCCCCGT(G/C)GCCTGCCTGGTGAGAGAGGTCGCCCTGGAG

[NM_000088.3](http://www.ncbi.nlm.nih.gov/entrez/viewer.fcgi?db=nucleotide&id=NM_000088.3): c.913G>A; [NP_000079.2](http://www.ncbi.nlm.nih.gov/entrez/viewer.fcgi?db=nucleotide&id=NP_000079.2): p.G305S

ttctcttggtttggttgtcagGGCCCCCGT(G/A)GCCTGCCTGGTGAGAGAGGTCGCCCTGGAG

[NM_000088.3](http://www.ncbi.nlm.nih.gov/entrez/viewer.fcgi?db=nucleotide&id=NM_000088.3): c.931G>C; [NP_000079.2](http://www.ncbi.nlm.nih.gov/entrez/viewer.fcgi?db=nucleotide&id=NP_000079.2): p.G311R

cagGGCCCCCGTGGCCTGCCTGGTGAGAGA(G/C)GTCGCCCTGGAGCCCCTGGCCCTGCTgtaa

[NM_000088.3](http://www.ncbi.nlm.nih.gov/entrez/viewer.fcgi?db=nucleotide&id=NM_000088.3): c.932G>A; [NP_000079.2](http://www.ncbi.nlm.nih.gov/entrez/viewer.fcgi?db=nucleotide&id=NP_000079.2): p.G311D

agGGCCCCCGTGGCCTGCCTGGTGAGAGAG(G/A)TCGCCCTGGAGCCCCTGGCCCTGCTgtaag

[NM_000088.3](http://www.ncbi.nlm.nih.gov/entrez/viewer.fcgi?db=nucleotide&id=NM_000088.3): c.934C>T; [NP_000079.2](http://www.ncbi.nlm.nih.gov/entrez/viewer.fcgi?db=nucleotide&id=NP_000079.2): p.R312C

GGCCCCCGTGGCCTGCCTGGTGAGAGAGGT(C/T)GCCCTGGAGCCCCTGGCCCTGCTgtaagta

[NM_000088.3](http://www.ncbi.nlm.nih.gov/entrez/viewer.fcgi?db=nucleotide&id=NM_000088.3): c.940G>A; [NP_000079.2](http://www.ncbi.nlm.nih.gov/entrez/viewer.fcgi?db=nucleotide&id=NP_000079.2): p.G314R

CGTGGCCTGCCTGGTGAGAGAGGTCGCCCT(G/A)GAGCCCCTGGCCCTGCTgtaagtactcctg

[NM_000088.3](http://www.ncbi.nlm.nih.gov/entrez/viewer.fcgi?db=nucleotide&id=NM_000088.3): c.959G>T; [NP_000079.2](http://www.ncbi.nlm.nih.gov/entrez/viewer.fcgi?db=nucleotide&id=NP_000079.2): p.G320V

cttaacggggcttcccctctctcctgcagG(G/T)TGCTCGTGGAAATGATGGTGCTACTGGTGC

[NM_000088.3](http://www.ncbi.nlm.nih.gov/entrez/viewer.fcgi?db=nucleotide&id=NM_000088.3): c.968G>A; [NP_000079.2](http://www.ncbi.nlm.nih.gov/entrez/viewer.fcgi?db=nucleotide&id=NP_000079.2): p.G323E

gcttcccctctctcctgcagGGTGCTCGTG(G/A)AAATGATGGTGCTACTGGTGCTGCCGGGCC

[NM_000088.3](http://www.ncbi.nlm.nih.gov/entrez/viewer.fcgi?db=nucleotide&id=NM_000088.3): c.977G>A; [NP_000079.2](http://www.ncbi.nlm.nih.gov/entrez/viewer.fcgi?db=nucleotide&id=NP_000079.2): p.G326D

ctctcctgcagGGTGCTCGTGGAAATGATG(G/A)TGCTACTGGTGCTGCCGGGCCCCCTgtgag

[NM_000088.3](http://www.ncbi.nlm.nih.gov/entrez/viewer.fcgi?db=nucleotide&id=NM_000088.3): c.995G>C; [NP_000079.2](http://www.ncbi.nlm.nih.gov/entrez/viewer.fcgi?db=nucleotide&id=NP_000079.2): p.G332A

GTGGAAATGATGGTGCTACTGGTGCTGCCG(G/C)GCCCCCTgtgagtgtggcctgtaggcctca

[NM_000088.3](http://www.ncbi.nlm.nih.gov/entrez/viewer.fcgi?db=nucleotide&id=NM_000088.3): c.994G>A; [NP_000079.2](http://www.ncbi.nlm.nih.gov/entrez/viewer.fcgi?db=nucleotide&id=NP_000079.2): p.G332R

CGTGGAAATGATGGTGCTACTGGTGCTGCC(G/A)GGCCCCCTgtgagtgtggcctgtaggcctc

[NM_000088.3](http://www.ncbi.nlm.nih.gov/entrez/viewer.fcgi?db=nucleotide&id=NM_000088.3): c.1012G>T; [NP_000079.2](http://www.ncbi.nlm.nih.gov/entrez/viewer.fcgi?db=nucleotide&id=NP_000079.2): p.G338C

acatctccatttcattcacagGGTCCCACC(G/T)GCCCCGCTGGTCCTCCTGGCTTCCCTGGTG

[NM_000088.3](http://www.ncbi.nlm.nih.gov/entrez/viewer.fcgi?db=nucleotide&id=NM_000088.3): c.1012G>A; [NP_000079.2](http://www.ncbi.nlm.nih.gov/entrez/viewer.fcgi?db=nucleotide&id=NP_000079.2): p.G338S

acatctccatttcattcacagGGTCCCACC(G/A)GCCCCGCTGGTCCTCCTGGCTTCCCTGGTG

[NM_000088.3](http://www.ncbi.nlm.nih.gov/entrez/viewer.fcgi?db=nucleotide&id=NM_000088.3): c.1045G>T; [NP_000079.2](http://www.ncbi.nlm.nih.gov/entrez/viewer.fcgi?db=nucleotide&id=NP_000079.2): p.V349F

CCCGCTGGTCCTCCTGGCTTCCCTGGTGCT(G/T)TTGGTGCTAAGgtgagaccccccactctcc

[NM_000088.3](http://www.ncbi.nlm.nih.gov/entrez/viewer.fcgi?db=nucleotide&id=NM_000088.3): c.1048G>C; [NP_000079.2](http://www.ncbi.nlm.nih.gov/entrez/viewer.fcgi?db=nucleotide&id=NP_000079.2): p.G350R

GCTGGTCCTCCTGGCTTCCCTGGTGCTGTT(G/C)GTGCTAAGgtgagaccccccactctcctct

[NM_000088.3](http://www.ncbi.nlm.nih.gov/entrez/viewer.fcgi?db=nucleotide&id=NM_000088.3): c.1058G>A; [NP_000079.2](http://www.ncbi.nlm.nih.gov/entrez/viewer.fcgi?db=nucleotide&id=NP_000079.2): p.G353D

cttctgatatgtcctttcttctgattcagG(G/A)TGAAGCTGGTCCCCAAGGGCCCCGAGGCTC

[NM_000088.3](http://www.ncbi.nlm.nih.gov/entrez/viewer.fcgi?db=nucleotide&id=NM_000088.3): c.1057G>T; [NP_000079.2](http://www.ncbi.nlm.nih.gov/entrez/viewer.fcgi?db=nucleotide&id=NP_000079.2): p.G353C

gcttctgatatgtcctttcttctgattcag(G/T)GTGAAGCTGGTCCCCAAGGGCCCCGAGGCT

[NM_000088.3](http://www.ncbi.nlm.nih.gov/entrez/viewer.fcgi?db=nucleotide&id=NM_000088.3): c.1057G>A; [NP_000079.2](http://www.ncbi.nlm.nih.gov/entrez/viewer.fcgi?db=nucleotide&id=NP_000079.2): p.G353S

gcttctgatatgtcctttcttctgattcag(G/A)GTGAAGCTGGTCCCCAAGGGCCCCGAGGCT

[NM_000088.3](http://www.ncbi.nlm.nih.gov/entrez/viewer.fcgi?db=nucleotide&id=NM_000088.3): c.1066G>T; [NP_000079.2](http://www.ncbi.nlm.nih.gov/entrez/viewer.fcgi?db=nucleotide&id=NP_000079.2): p.G356C

atgtcctttcttctgattcagGGTGAAGCT(G/T)GTCCCCAAGGGCCCCGAGGCTCTGAAGGTC

[NM_000088.3](http://www.ncbi.nlm.nih.gov/entrez/viewer.fcgi?db=nucleotide&id=NM_000088.3): c.1081C>T; [NP_000079.2](http://www.ncbi.nlm.nih.gov/entrez/viewer.fcgi?db=nucleotide&id=NP_000079.2): p.R361X

attcagGGTGAAGCTGGTCCCCAAGGGCCC(C/T)GAGGCTCTGAAGGTCCCCAGGGTGTGCGTG

[NM_000088.3](http://www.ncbi.nlm.nih.gov/entrez/viewer.fcgi?db=nucleotide&id=NM_000088.3): c.1094G>C; [NP_000079.2](http://www.ncbi.nlm.nih.gov/entrez/viewer.fcgi?db=nucleotide&id=NP_000079.2): p.G365A

CTGGTCCCCAAGGGCCCCGAGGCTCTGAAG(G/C)TCCCCAGGGTGTGCGTGGTGAGCCTGGCCC

[NM_000088.3](http://www.ncbi.nlm.nih.gov/entrez/viewer.fcgi?db=nucleotide&id=NM_000088.3): c.1094G>T; [NP_000079.2](http://www.ncbi.nlm.nih.gov/entrez/viewer.fcgi?db=nucleotide&id=NP_000079.2): p.G365V

CTGGTCCCCAAGGGCCCCGAGGCTCTGAAG(G/T)TCCCCAGGGTGTGCGTGGTGAGCCTGGCCC

[NM_000088.3](http://www.ncbi.nlm.nih.gov/entrez/viewer.fcgi?db=nucleotide&id=NM_000088.3): c.1102G>A; [NP_000079.2](http://www.ncbi.nlm.nih.gov/entrez/viewer.fcgi?db=nucleotide&id=NP_000079.2): p.G368S

CAAGGGCCCCGAGGCTCTGAAGGTCCCCAG(G/A)GTGTGCGTGGTGAGCCTGGCCCCCCTGGCC

[NM_000088.3](http://www.ncbi.nlm.nih.gov/entrez/viewer.fcgi?db=nucleotide&id=NM_000088.3): c.1103G>T; [NP_000079.2](http://www.ncbi.nlm.nih.gov/entrez/viewer.fcgi?db=nucleotide&id=NP_000079.2): p.G368V

AAGGGCCCCGAGGCTCTGAAGGTCCCCAGG(G/T)TGTGCGTGGTGAGCCTGGCCCCCCTGGCCC

[NM_000088.3](http://www.ncbi.nlm.nih.gov/entrez/viewer.fcgi?db=nucleotide&id=NM_000088.3): c.1111G>A; [NP_000079.2](http://www.ncbi.nlm.nih.gov/entrez/viewer.fcgi?db=nucleotide&id=NP_000079.2): p.G371S

CGAGGCTCTGAAGGTCCCCAGGGTGTGCGT(G/A)GTGAGCCTGGCCCCCCTGGCCCTGCTGGTG

[NM_000088.3](http://www.ncbi.nlm.nih.gov/entrez/viewer.fcgi?db=nucleotide&id=NM_000088.3): c.1130G>C; [NP_000079.2](http://www.ncbi.nlm.nih.gov/entrez/viewer.fcgi?db=nucleotide&id=NP_000079.2): p.G377A

AGGGTGTGCGTGGTGAGCCTGGCCCCCCTG(G/C)CCCTGCTGGTGCTGCTGGCCCTGCTgtaag

[NM_000088.3](http://www.ncbi.nlm.nih.gov/entrez/viewer.fcgi?db=nucleotide&id=NM_000088.3): c.1138G>T; [NP_000079.2](http://www.ncbi.nlm.nih.gov/entrez/viewer.fcgi?db=nucleotide&id=NP_000079.2): p.G380C

CGTGGTGAGCCTGGCCCCCCTGGCCCTGCT(G/T)GTGCTGCTGGCCCTGCTgtaagtgtccccg

[NM_000088.3](http://www.ncbi.nlm.nih.gov/entrez/viewer.fcgi?db=nucleotide&id=NM_000088.3): c.1139G>T; [NP_000079.2](http://www.ncbi.nlm.nih.gov/entrez/viewer.fcgi?db=nucleotide&id=NP_000079.2): p.G380V

GTGGTGAGCCTGGCCCCCCTGGCCCTGCTG(G/T)TGCTGCTGGCCCTGCTgtaagtgtccccga

[NM_000088.3](http://www.ncbi.nlm.nih.gov/entrez/viewer.fcgi?db=nucleotide&id=NM_000088.3): c.1147G>T; [NP_000079.2](http://www.ncbi.nlm.nih.gov/entrez/viewer.fcgi?db=nucleotide&id=NP_000079.2): p.G383C

CCTGGCCCCCCTGGCCCTGCTGGTGCTGCT(G/T)GCCCTGCTgtaagtgtccccgactcagtgt

[NM_000088.3](http://www.ncbi.nlm.nih.gov/entrez/viewer.fcgi?db=nucleotide&id=NM_000088.3): c.1147G>A; [NP_000079.2](http://www.ncbi.nlm.nih.gov/entrez/viewer.fcgi?db=nucleotide&id=NP_000079.2): p.G383S

CCTGGCCCCCCTGGCCCTGCTGGTGCTGCT(G/A)GCCCTGCTgtaagtgtccccgactcagtgt

[NM_000088.3](http://www.ncbi.nlm.nih.gov/entrez/viewer.fcgi?db=nucleotide&id=NM_000088.3): c.1165G>C; [NP_000079.2](http://www.ncbi.nlm.nih.gov/entrez/viewer.fcgi?db=nucleotide&id=NP_000079.2): p.G389R

actcctttctctgtgccacagGGAAACCCT(G/C)GTGCTGATGGACAGCCTGGTGCTAAAGGTG

[NM_000088.3](http://www.ncbi.nlm.nih.gov/entrez/viewer.fcgi?db=nucleotide&id=NM_000088.3): c.1165G>T; [NP_000079.2](http://www.ncbi.nlm.nih.gov/entrez/viewer.fcgi?db=nucleotide&id=NP_000079.2): p.G389C

actcctttctctgtgccacagGGAAACCCT(G/T)GTGCTGATGGACAGCCTGGTGCTAAAGGTG

[NM_000088.3](http://www.ncbi.nlm.nih.gov/entrez/viewer.fcgi?db=nucleotide&id=NM_000088.3): c.1165G>A; [NP_000079.2](http://www.ncbi.nlm.nih.gov/entrez/viewer.fcgi?db=nucleotide&id=NP_000079.2): p.G389S

actcctttctctgtgccacagGGAAACCCT(G/A)GTGCTGATGGACAGCCTGGTGCTAAAGGTG

[NM_000088.3](http://www.ncbi.nlm.nih.gov/entrez/viewer.fcgi?db=nucleotide&id=NM_000088.3): c.1168G>A; [NP_000079.2](http://www.ncbi.nlm.nih.gov/entrez/viewer.fcgi?db=nucleotide&id=NP_000079.2): p.A390T

cctttctctgtgccacagGGAAACCCTGGT(G/A)CTGATGGACAGCCTGGTGCTAAAGGTGCCA

[NM_000088.3](http://www.ncbi.nlm.nih.gov/entrez/viewer.fcgi?db=nucleotide&id=NM_000088.3): c.1175G>A; [NP_000079.2](http://www.ncbi.nlm.nih.gov/entrez/viewer.fcgi?db=nucleotide&id=NP_000079.2): p.G392E

ctgtgccacagGGAAACCCTGGTGCTGATG(G/A)ACAGCCTGGTGCTAAAGGTGCCAATgtaag

[NM_000088.3](http://www.ncbi.nlm.nih.gov/entrez/viewer.fcgi?db=nucleotide&id=NM_000088.3): c.1183G>A; [NP_000079.2](http://www.ncbi.nlm.nih.gov/entrez/viewer.fcgi?db=nucleotide&id=NP_000079.2): p.G395S

cagGGAAACCCTGGTGCTGATGGACAGCCT(G/A)GTGCTAAAGGTGCCAATgtaagtatcctgc

[NM_000088.3](http://www.ncbi.nlm.nih.gov/entrez/viewer.fcgi?db=nucleotide&id=NM_000088.3): c.1193G>C; [NP_000079.2](http://www.ncbi.nlm.nih.gov/entrez/viewer.fcgi?db=nucleotide&id=NP_000079.2): p.G398A

CTGGTGCTGATGGACAGCCTGGTGCTAAAG(G/C)TGCCAATgtaagtatcctgccaggcttcag

[NM_000088.3](http://www.ncbi.nlm.nih.gov/entrez/viewer.fcgi?db=nucleotide&id=NM_000088.3): c.1193G>A; [NP_000079.2](http://www.ncbi.nlm.nih.gov/entrez/viewer.fcgi?db=nucleotide&id=NP_000079.2): p.G398D

CTGGTGCTGATGGACAGCCTGGTGCTAAAG(G/A)TGCCAATgtaagtatcctgccaggcttcag

[NM_000088.3](http://www.ncbi.nlm.nih.gov/entrez/viewer.fcgi?db=nucleotide&id=NM_000088.3): c.1192G>T; [NP_000079.2](http://www.ncbi.nlm.nih.gov/entrez/viewer.fcgi?db=nucleotide&id=NP_000079.2): p.G398C

CCTGGTGCTGATGGACAGCCTGGTGCTAAA(G/T)GTGCCAATgtaagtatcctgccaggcttca

[NM_000088.3](http://www.ncbi.nlm.nih.gov/entrez/viewer.fcgi?db=nucleotide&id=NM_000088.3): c.1202G>A; [NP_000079.2](http://www.ncbi.nlm.nih.gov/entrez/viewer.fcgi?db=nucleotide&id=NP_000079.2): p.G401D

ctcacgccctggctgtctgcctcccacagG(G/A)TGCTCCTGGTATTGCTGGTGCTCCTGGCTT

[NM_000088.3](http://www.ncbi.nlm.nih.gov/entrez/viewer.fcgi?db=nucleotide&id=NM_000088.3): c.1201G>T; [NP_000079.2](http://www.ncbi.nlm.nih.gov/entrez/viewer.fcgi?db=nucleotide&id=NP_000079.2): p.G401C

gctcacgccctggctgtctgcctcccacag(G/T)GTGCTCCTGGTATTGCTGGTGCTCCTGGCT

[NM_000088.3](http://www.ncbi.nlm.nih.gov/entrez/viewer.fcgi?db=nucleotide&id=NM_000088.3): c.1210G>T; [NP_000079.2](http://www.ncbi.nlm.nih.gov/entrez/viewer.fcgi?db=nucleotide&id=NP_000079.2): p.G404C

ctggctgtctgcctcccacagGGTGCTCCT(G/T)GTATTGCTGGTGCTCCTGGCTTCCCTGGTG

[NM_000088.3](http://www.ncbi.nlm.nih.gov/entrez/viewer.fcgi?db=nucleotide&id=NM_000088.3): c.1210G>A; [NP_000079.2](http://www.ncbi.nlm.nih.gov/entrez/viewer.fcgi?db=nucleotide&id=NP_000079.2): p.G404S

ctggctgtctgcctcccacagGGTGCTCCT(G/A)GTATTGCTGGTGCTCCTGGCTTCCCTGGTG

[NM_000088.3](http://www.ncbi.nlm.nih.gov/entrez/viewer.fcgi?db=nucleotide&id=NM_000088.3): c.1219G>T; [NP_000079.2](http://www.ncbi.nlm.nih.gov/entrez/viewer.fcgi?db=nucleotide&id=NP_000079.2): p.G407C

tgcctcccacagGGTGCTCCTGGTATTGCT(G/T)GTGCTCCTGGCTTCCCTGGTGCCCGAGGCC

[NM_000088.3](http://www.ncbi.nlm.nih.gov/entrez/viewer.fcgi?db=nucleotide&id=NM_000088.3): c.1219G>A; [NP_000079.2](http://www.ncbi.nlm.nih.gov/entrez/viewer.fcgi?db=nucleotide&id=NP_000079.2): p.G407S

tgcctcccacagGGTGCTCCTGGTATTGCT(G/A)GTGCTCCTGGCTTCCCTGGTGCCCGAGGCC

[NM_000088.3](http://www.ncbi.nlm.nih.gov/entrez/viewer.fcgi?db=nucleotide&id=NM_000088.3): c.1228G>A; [NP_000079.2](http://www.ncbi.nlm.nih.gov/entrez/viewer.fcgi?db=nucleotide&id=NP_000079.2): p.G410S

cagGGTGCTCCTGGTATTGCTGGTGCTCCT(G/A)GCTTCCCTGGTGCCCGAGGCCCCTCTGGAC

[NM_000088.3](http://www.ncbi.nlm.nih.gov/entrez/viewer.fcgi?db=nucleotide&id=NM_000088.3): c.1238G>C; [NP_000079.2](http://www.ncbi.nlm.nih.gov/entrez/viewer.fcgi?db=nucleotide&id=NP_000079.2): p.G413A

CTGGTATTGCTGGTGCTCCTGGCTTCCCTG(G/C)TGCCCGAGGCCCCTCTGGACCCCAGGGCCC

[NM_000088.3](http://www.ncbi.nlm.nih.gov/entrez/viewer.fcgi?db=nucleotide&id=NM_000088.3): c.1243C>T; [NP_000079.2](http://www.ncbi.nlm.nih.gov/entrez/viewer.fcgi?db=nucleotide&id=NP_000079.2): p.R415X

ATTGCTGGTGCTCCTGGCTTCCCTGGTGCC(C/T)GAGGCCCCTCTGGACCCCAGGGCCCCGGCG

[NM_000088.3](http://www.ncbi.nlm.nih.gov/entrez/viewer.fcgi?db=nucleotide&id=NM_000088.3): c.1249C>G; [NP_000079.2](http://www.ncbi.nlm.nih.gov/entrez/viewer.fcgi?db=nucleotide&id=NP_000079.2): p.P417A

GGTGCTCCTGGCTTCCCTGGTGCCCGAGGC(C/G)CCTCTGGACCCCAGGGCCCCGGCGGCCCTC

[NM_000088.3](http://www.ncbi.nlm.nih.gov/entrez/viewer.fcgi?db=nucleotide&id=NM_000088.3): c.1265G>C; [NP_000079.2](http://www.ncbi.nlm.nih.gov/entrez/viewer.fcgi?db=nucleotide&id=NP_000079.2): p.G422A

CTGGTGCCCGAGGCCCCTCTGGACCCCAGG(G/C)CCCCGGCGGCCCTCCTGGTCCCAAGGGTAA

[NM_000088.3](http://www.ncbi.nlm.nih.gov/entrez/viewer.fcgi?db=nucleotide&id=NM_000088.3): c.1264G>T; [NP_000079.2](http://www.ncbi.nlm.nih.gov/entrez/viewer.fcgi?db=nucleotide&id=NP_000079.2): p.G422C

CCTGGTGCCCGAGGCCCCTCTGGACCCCAG(G/T)GCCCCGGCGGCCCTCCTGGTCCCAAGGGTA

[NM_000088.3](http://www.ncbi.nlm.nih.gov/entrez/viewer.fcgi?db=nucleotide&id=NM_000088.3): c.1273G>A; [NP_000079.2](http://www.ncbi.nlm.nih.gov/entrez/viewer.fcgi?db=nucleotide&id=NP_000079.2): p.G425S

CGAGGCCCCTCTGGACCCCAGGGCCCCGGC(G/A)GCCCTCCTGGTCCCAAGGGTAACAGCgtga

[NM_000088.3](http://www.ncbi.nlm.nih.gov/entrez/viewer.fcgi?db=nucleotide&id=NM_000088.3): c.1282G>T; [NP_000079.2](http://www.ncbi.nlm.nih.gov/entrez/viewer.fcgi?db=nucleotide&id=NP_000079.2): p.G428C

TCTGGACCCCAGGGCCCCGGCGGCCCTCCT(G/T)GTCCCAAGGGTAACAGCgtgagtaccaaac

[NM_000088.3](http://www.ncbi.nlm.nih.gov/entrez/viewer.fcgi?db=nucleotide&id=NM_000088.3): c.1292G>T; [NP_000079.2](http://www.ncbi.nlm.nih.gov/entrez/viewer.fcgi?db=nucleotide&id=NP_000079.2): p.G431V

AGGGCCCCGGCGGCCCTCCTGGTCCCAAGG(G/T)TAACAGCgtgagtaccaaactctcccttct

[NM_000088.3](http://www.ncbi.nlm.nih.gov/entrez/viewer.fcgi?db=nucleotide&id=NM_000088.3): c.1301G>T; [NP_000079.2](http://www.ncbi.nlm.nih.gov/entrez/viewer.fcgi?db=nucleotide&id=NP_000079.2): p.G434V

tcagctcatcctcttctccccccttgcagG(G/T)TGAACCTGGTGCTCCTGGCAGCAAAGGAGA

[NM_000088.3](http://www.ncbi.nlm.nih.gov/entrez/viewer.fcgi?db=nucleotide&id=NM_000088.3): c.1310G>A; [NP_000079.2](http://www.ncbi.nlm.nih.gov/entrez/viewer.fcgi?db=nucleotide&id=NP_000079.2): p.G437D

cctcttctccccccttgcagGGTGAACCTG(G/A)TGCTCCTGGCAGCAAAGGAGACACTGGTGC

[NM_000088.3](http://www.ncbi.nlm.nih.gov/entrez/viewer.fcgi?db=nucleotide&id=NM_000088.3): c.1364G>A; [NP_000079.2](http://www.ncbi.nlm.nih.gov/entrez/viewer.fcgi?db=nucleotide&id=NP_000079.2): p.G455D

ctcctctcggccctctccagGGCCCTGTTG(G/A)TGTTCAAGGACCCCCTGGCCCTGCTGGAGA

[NM_000088.3](http://www.ncbi.nlm.nih.gov/entrez/viewer.fcgi?db=nucleotide&id=NM_000088.3): c.1363G>T; [NP_000079.2](http://www.ncbi.nlm.nih.gov/entrez/viewer.fcgi?db=nucleotide&id=NP_000079.2): p.G455C

cctcctctcggccctctccagGGCCCTGTT(G/T)GTGTTCAAGGACCCCCTGGCCCTGCTGGAG

[NM_000088.3](http://www.ncbi.nlm.nih.gov/entrez/viewer.fcgi?db=nucleotide&id=NM_000088.3): c.1381G>A; [NP_000079.2](http://www.ncbi.nlm.nih.gov/entrez/viewer.fcgi?db=nucleotide&id=NP_000079.2): p.G461S

cagGGCCCTGTTGGTGTTCAAGGACCCCCT(G/A)GCCCTGCTGGAGAGGAAGGAAAGCGAGGAG

[NM_000088.3](http://www.ncbi.nlm.nih.gov/entrez/viewer.fcgi?db=nucleotide&id=NM_000088.3): c.1390G>A; [NP_000079.2](http://www.ncbi.nlm.nih.gov/entrez/viewer.fcgi?db=nucleotide&id=NP_000079.2): p.G464R

GTTGGTGTTCAAGGACCCCCTGGCCCTGCT(G/A)GAGAGGAAGGAAAGCGAGGAGCTCGAGGTG

[NM_000088.3](http://www.ncbi.nlm.nih.gov/entrez/viewer.fcgi?db=nucleotide&id=NM_000088.3): c.1391G>A; [NP_000079.2](http://www.ncbi.nlm.nih.gov/entrez/viewer.fcgi?db=nucleotide&id=NP_000079.2): p.G464E

TTGGTGTTCAAGGACCCCCTGGCCCTGCTG(G/A)AGAGGAAGGAAAGCGAGGAGCTCGAGGTGA

[NM_000088.3](http://www.ncbi.nlm.nih.gov/entrez/viewer.fcgi?db=nucleotide&id=NM_000088.3): c.1405C>T; [NP_000079.2](http://www.ncbi.nlm.nih.gov/entrez/viewer.fcgi?db=nucleotide&id=NP_000079.2): p.R469X

CCCCCTGGCCCTGCTGGAGAGGAAGGAAAG(C/T)GAGGAGCTCGAGGTGAACCCGGACCCACTG

[NM_000088.3](http://www.ncbi.nlm.nih.gov/entrez/viewer.fcgi?db=nucleotide&id=NM_000088.3): c.1409G>C; [NP_000079.2](http://www.ncbi.nlm.nih.gov/entrez/viewer.fcgi?db=nucleotide&id=NP_000079.2): p.G470A

CTGGCCCTGCTGGAGAGGAAGGAAAGCGAG(G/C)AGCTCGAGGTGAACCCGGACCCACTGGCCT

[NM_000088.3](http://www.ncbi.nlm.nih.gov/entrez/viewer.fcgi?db=nucleotide&id=NM_000088.3): c.1409G>T; [NP_000079.2](http://www.ncbi.nlm.nih.gov/entrez/viewer.fcgi?db=nucleotide&id=NP_000079.2): p.G470V

CTGGCCCTGCTGGAGAGGAAGGAAAGCGAG(G/T)AGCTCGAGGTGAACCCGGACCCACTGGCCT

[NM_000088.3](http://www.ncbi.nlm.nih.gov/entrez/viewer.fcgi?db=nucleotide&id=NM_000088.3): c.1414C>T; [NP_000079.2](http://www.ncbi.nlm.nih.gov/entrez/viewer.fcgi?db=nucleotide&id=NP_000079.2): p.R472X

CCTGCTGGAGAGGAAGGAAAGCGAGGAGCT(C/T)GAGGTGAACCCGGACCCACTGGCCTGCCCG

[NM_000088.3](http://www.ncbi.nlm.nih.gov/entrez/viewer.fcgi?db=nucleotide&id=NM_000088.3): c.1426G>A; [NP_000079.2](http://www.ncbi.nlm.nih.gov/entrez/viewer.fcgi?db=nucleotide&id=NP_000079.2): p.G476R

GAAGGAAAGCGAGGAGCTCGAGGTGAACCC(G/A)GACCCACTGGCCTGCCCGGACCCCCTGGCG

[NM_000088.3](http://www.ncbi.nlm.nih.gov/entrez/viewer.fcgi?db=nucleotide&id=NM_000088.3): c.1426G>C; [NP_000079.2](http://www.ncbi.nlm.nih.gov/entrez/viewer.fcgi?db=nucleotide&id=NP_000079.2): p.G476R

GAAGGAAAGCGAGGAGCTCGAGGTGAACCC(G/C)GACCCACTGGCCTGCCCGGACCCCCTGGCG

[NM_000088.3](http://www.ncbi.nlm.nih.gov/entrez/viewer.fcgi?db=nucleotide&id=NM_000088.3): c.1445G>C; [NP_000079.2](http://www.ncbi.nlm.nih.gov/entrez/viewer.fcgi?db=nucleotide&id=NP_000079.2): p.G482A

GAGGTGAACCCGGACCCACTGGCCTGCCCG(G/C)ACCCCCTGGCGAGCGTgtaagtgtccctgc

[NM_000088.3](http://www.ncbi.nlm.nih.gov/entrez/viewer.fcgi?db=nucleotide&id=NM_000088.3): c.1444G>A; [NP_000079.2](http://www.ncbi.nlm.nih.gov/entrez/viewer.fcgi?db=nucleotide&id=NP_000079.2): p.G482R

CGAGGTGAACCCGGACCCACTGGCCTGCCC(G/A)GACCCCCTGGCGAGCGTgtaagtgtccctg

[NM_000088.3](http://www.ncbi.nlm.nih.gov/entrez/viewer.fcgi?db=nucleotide&id=NM_000088.3): c.1471G>T; [NP_000079.2](http://www.ncbi.nlm.nih.gov/entrez/viewer.fcgi?db=nucleotide&id=NP_000079.2): p.G491C

tggcctcctctcctcctgcagGGTGGACCT(G/T)GTAGCCGTGGTTTCCCTGGCGCAGATGGTG

[NM_000088.3](http://www.ncbi.nlm.nih.gov/entrez/viewer.fcgi?db=nucleotide&id=NM_000088.3): c.1507G>T; [NP_000079.2](http://www.ncbi.nlm.nih.gov/entrez/viewer.fcgi?db=nucleotide&id=NP_000079.2): p.G503C

CGTGGTTTCCCTGGCGCAGATGGTGTTGCT(G/T)GTCCCAAGgtaacctctccttgcggccggg

[NM_000088.3](http://www.ncbi.nlm.nih.gov/entrez/viewer.fcgi?db=nucleotide&id=NM_000088.3): c.1526G>T; [NP_000079.2](http://www.ncbi.nlm.nih.gov/entrez/viewer.fcgi?db=nucleotide&id=NP_000079.2): p.G509V

ctgtgacttcccccaaccagGGTCCCGCTG(G/T)TGAACGTGGTTCTCCTGGCCCTGCTGGCCC

[NM_000088.3](http://www.ncbi.nlm.nih.gov/entrez/viewer.fcgi?db=nucleotide&id=NM_000088.3): c.1580G>C; [NP_000079.2](http://www.ncbi.nlm.nih.gov/entrez/viewer.fcgi?db=nucleotide&id=NP_000079.2): p.G527A

CTGGCCCCAAAGGATCTCCTGGTGAAGCTG(G/C)TCGTCCCGGTGAAGCTGGTCTGCCTGGTGC

[NM_000088.3](http://www.ncbi.nlm.nih.gov/entrez/viewer.fcgi?db=nucleotide&id=NM_000088.3): c.1579G>T; [NP_000079.2](http://www.ncbi.nlm.nih.gov/entrez/viewer.fcgi?db=nucleotide&id=NP_000079.2): p.G527C

GCTGGCCCCAAAGGATCTCCTGGTGAAGCT(G/T)GTCGTCCCGGTGAAGCTGGTCTGCCTGGTG

[NM_000088.3](http://www.ncbi.nlm.nih.gov/entrez/viewer.fcgi?db=nucleotide&id=NM_000088.3): c.1588G>T; [NP_000079.2](http://www.ncbi.nlm.nih.gov/entrez/viewer.fcgi?db=nucleotide&id=NP_000079.2): p.G530C

AAAGGATCTCCTGGTGAAGCTGGTCGTCCC(G/T)GTGAAGCTGGTCTGCCTGGTGCCAAGgtga

[NM_000088.3](http://www.ncbi.nlm.nih.gov/entrez/viewer.fcgi?db=nucleotide&id=NM_000088.3): c.1588G>A; [NP_000079.2](http://www.ncbi.nlm.nih.gov/entrez/viewer.fcgi?db=nucleotide&id=NP_000079.2): p.G530S

AAAGGATCTCCTGGTGAAGCTGGTCGTCCC(G/A)GTGAAGCTGGTCTGCCTGGTGCCAAGgtga

[NM_000088.3](http://www.ncbi.nlm.nih.gov/entrez/viewer.fcgi?db=nucleotide&id=NM_000088.3): c.1598G>A; [NP_000079.2](http://www.ncbi.nlm.nih.gov/entrez/viewer.fcgi?db=nucleotide&id=NP_000079.2): p.G533D

CTGGTGAAGCTGGTCGTCCCGGTGAAGCTG(G/A)TCTGCCTGGTGCCAAGgtgaggccccaggc

[NM_000088.3](http://www.ncbi.nlm.nih.gov/entrez/viewer.fcgi?db=nucleotide&id=NM_000088.3): c.1597G>T; [NP_000079.2](http://www.ncbi.nlm.nih.gov/entrez/viewer.fcgi?db=nucleotide&id=NP_000079.2): p.G533C

CCTGGTGAAGCTGGTCGTCCCGGTGAAGCT(G/T)GTCTGCCTGGTGCCAAGgtgaggccccagg

[NM_000088.3](http://www.ncbi.nlm.nih.gov/entrez/viewer.fcgi?db=nucleotide&id=NM_000088.3): c.1643G>C; [NP_000079.2](http://www.ncbi.nlm.nih.gov/entrez/viewer.fcgi?db=nucleotide&id=NP_000079.2): p.G548A

agGGTCTGACTGGAAGCCCTGGCAGCCCTG(G/C)TCCTGATGGCAAAACTGGCCCCCCTgtaag

[NM_000088.3](http://www.ncbi.nlm.nih.gov/entrez/viewer.fcgi?db=nucleotide&id=NM_000088.3): c.1661G>C; [NP_000079.2](http://www.ncbi.nlm.nih.gov/entrez/viewer.fcgi?db=nucleotide&id=NP_000079.2): p.G554A

CTGGCAGCCCTGGTCCTGATGGCAAAACTG(G/C)CCCCCCTgtaagtatcactccccctgaacc

[NM_000088.3](http://www.ncbi.nlm.nih.gov/entrez/viewer.fcgi?db=nucleotide&id=NM_000088.3): c.1664C>G; [NP_000079.2](http://www.ncbi.nlm.nih.gov/entrez/viewer.fcgi?db=nucleotide&id=NP_000079.2): p.P555R

GCAGCCCTGGTCCTGATGGCAAAACTGGCC(C/G)CCCTgtaagtatcactccccctgaaccccc

[NM_000088.3](http://www.ncbi.nlm.nih.gov/entrez/viewer.fcgi?db=nucleotide&id=NM_000088.3): c.1678G>C; [NP_000079.2](http://www.ncbi.nlm.nih.gov/entrez/viewer.fcgi?db=nucleotide&id=NP_000079.2): p.G560R

tttcgtgcctcccatccttagGGTCCCGCC(G/C)GTCAAGATGGTCGCCCCGGACCCCCAGGCC

[NM_000088.3](http://www.ncbi.nlm.nih.gov/entrez/viewer.fcgi?db=nucleotide&id=NM_000088.3): c.1678G>T; [NP_000079.2](http://www.ncbi.nlm.nih.gov/entrez/viewer.fcgi?db=nucleotide&id=NP_000079.2): p.G560C

tttcgtgcctcccatccttagGGTCCCGCC(G/T)GTCAAGATGGTCGCCCCGGACCCCCAGGCC

[NM_000088.3](http://www.ncbi.nlm.nih.gov/entrez/viewer.fcgi?db=nucleotide&id=NM_000088.3): c.1678G>A; [NP_000079.2](http://www.ncbi.nlm.nih.gov/entrez/viewer.fcgi?db=nucleotide&id=NP_000079.2): p.G560S

tttcgtgcctcccatccttagGGTCCCGCC(G/A)GTCAAGATGGTCGCCCCGGACCCCCAGGCC

[NM_000088.3](http://www.ncbi.nlm.nih.gov/entrez/viewer.fcgi?db=nucleotide&id=NM_000088.3): c.1696G>A; [NP_000079.2](http://www.ncbi.nlm.nih.gov/entrez/viewer.fcgi?db=nucleotide&id=NP_000079.2): p.G566R

tagGGTCCCGCCGGTCAAGATGGTCGCCCC(G/A)GACCCCCAGGCCCACCTGGTGCCCGTGGTC

[NM_000088.3](http://www.ncbi.nlm.nih.gov/entrez/viewer.fcgi?db=nucleotide&id=NM_000088.3): c.1705G>C; [NP_000079.2](http://www.ncbi.nlm.nih.gov/entrez/viewer.fcgi?db=nucleotide&id=NP_000079.2): p.G569R

GCCGGTCAAGATGGTCGCCCCGGACCCCCA(G/C)GCCCACCTGGTGCCCGTGGTCAGGCTGGTG

[NM_000088.3](http://www.ncbi.nlm.nih.gov/entrez/viewer.fcgi?db=nucleotide&id=NM_000088.3): c.1720C>T; [NP_000079.2](http://www.ncbi.nlm.nih.gov/entrez/viewer.fcgi?db=nucleotide&id=NP_000079.2): p.R574C

CGCCCCGGACCCCCAGGCCCACCTGGTGCC(C/T)GTGGTCAGGCTGGTGTGATGGGATTCCCTG

[NM_000088.3](http://www.ncbi.nlm.nih.gov/entrez/viewer.fcgi?db=nucleotide&id=NM_000088.3): c.1741G>A; [NP_000079.2](http://www.ncbi.nlm.nih.gov/entrez/viewer.fcgi?db=nucleotide&id=NP_000079.2): p.G581R

CCTGGTGCCCGTGGTCAGGCTGGTGTGATG(G/A)GATTCCCTGGACCTAAAGGTGCTGCTgtga

[NM_000088.3](http://www.ncbi.nlm.nih.gov/entrez/viewer.fcgi?db=nucleotide&id=NM_000088.3): c.1760G>C; [NP_000079.2](http://www.ncbi.nlm.nih.gov/entrez/viewer.fcgi?db=nucleotide&id=NP_000079.2): p.G587A

CTGGTGTGATGGGATTCCCTGGACCTAAAG(G/C)TGCTGCTgtgagtattaagtgaggatccat

[NM_000088.3](http://www.ncbi.nlm.nih.gov/entrez/viewer.fcgi?db=nucleotide&id=NM_000088.3): c.1759G>A; [NP_000079.2](http://www.ncbi.nlm.nih.gov/entrez/viewer.fcgi?db=nucleotide&id=NP_000079.2): p.G587S

GCTGGTGTGATGGGATTCCCTGGACCTAAA(G/A)GTGCTGCTgtgagtattaagtgaggatcca

[NM_000088.3](http://www.ncbi.nlm.nih.gov/entrez/viewer.fcgi?db=nucleotide&id=NM_000088.3): c.1778G>A; [NP_000079.2](http://www.ncbi.nlm.nih.gov/entrez/viewer.fcgi?db=nucleotide&id=NP_000079.2): p.G593D

ctaactccctttctccacagGGAGAGCCCG(G/A)CAAGGCTGGAGAGCGAGGTGTTCCCGGACC

[NM_000088.3](http://www.ncbi.nlm.nih.gov/entrez/viewer.fcgi?db=nucleotide&id=NM_000088.3): c.1777G>T; [NP_000079.2](http://www.ncbi.nlm.nih.gov/entrez/viewer.fcgi?db=nucleotide&id=NP_000079.2): p.G593C

cctaactccctttctccacagGGAGAGCCC(G/T)GCAAGGCTGGAGAGCGAGGTGTTCCCGGAC

[NM_000088.3](http://www.ncbi.nlm.nih.gov/entrez/viewer.fcgi?db=nucleotide&id=NM_000088.3): c.1777G>A; [NP_000079.2](http://www.ncbi.nlm.nih.gov/entrez/viewer.fcgi?db=nucleotide&id=NP_000079.2): p.G593S

cctaactccctttctccacagGGAGAGCCC(G/A)GCAAGGCTGGAGAGCGAGGTGTTCCCGGAC

[NM_000088.3](http://www.ncbi.nlm.nih.gov/entrez/viewer.fcgi?db=nucleotide&id=NM_000088.3): c.1792C>T; [NP_000079.2](http://www.ncbi.nlm.nih.gov/entrez/viewer.fcgi?db=nucleotide&id=NP_000079.2): p.R598X

ccacagGGAGAGCCCGGCAAGGCTGGAGAG(C/T)GAGGTGTTCCCGGACCCCCTGGCGCTGTCg

[NM_000088.3](http://www.ncbi.nlm.nih.gov/entrez/viewer.fcgi?db=nucleotide&id=NM_000088.3): c.1804G>A; [NP_000079.2](http://www.ncbi.nlm.nih.gov/entrez/viewer.fcgi?db=nucleotide&id=NP_000079.2): p.G602R

CCCGGCAAGGCTGGAGAGCGAGGTGTTCCC(G/A)GACCCCCTGGCGCTGTCgtaagtatctcct

[NM_000088.3](http://www.ncbi.nlm.nih.gov/entrez/viewer.fcgi?db=nucleotide&id=NM_000088.3): c.1814G>A; [NP_000079.2](http://www.ncbi.nlm.nih.gov/entrez/viewer.fcgi?db=nucleotide&id=NP_000079.2): p.G605D

CTGGAGAGCGAGGTGTTCCCGGACCCCCTG(G/A)CGCTGTCgtaagtatctcctttccatccct

[NM_000088.3](http://www.ncbi.nlm.nih.gov/entrez/viewer.fcgi?db=nucleotide&id=NM_000088.3): c.1840G>C; [NP_000079.2](http://www.ncbi.nlm.nih.gov/entrez/viewer.fcgi?db=nucleotide&id=NP_000079.2): p.G614R

ctctccctgcagGGTCCTGCTGGCAAAGAT(G/C)GAGAGGCTGGAGCTCAGGGACCCCCTGGCC

[NM_000088.3](http://www.ncbi.nlm.nih.gov/entrez/viewer.fcgi?db=nucleotide&id=NM_000088.3): c.1849G>A; [NP_000079.2](http://www.ncbi.nlm.nih.gov/entrez/viewer.fcgi?db=nucleotide&id=NP_000079.2): p.G617R

cagGGTCCTGCTGGCAAAGATGGAGAGGCT(G/A)GAGCTCAGGGACCCCCTGGCCCTGCTgtga

[NM_000088.3](http://www.ncbi.nlm.nih.gov/entrez/viewer.fcgi?db=nucleotide&id=NM_000088.3): c.1876G>T; [NP_000079.2](http://www.ncbi.nlm.nih.gov/entrez/viewer.fcgi?db=nucleotide&id=NP_000079.2): p.G626C

ccccgtgactgtagtgttctctctgtgcag(G/T)GTCCCGCTGGCGAGAGAGGTGAACAAGGCC

[NM_000088.3](http://www.ncbi.nlm.nih.gov/entrez/viewer.fcgi?db=nucleotide&id=NM_000088.3): c.1876G>A; [NP_000079.2](http://www.ncbi.nlm.nih.gov/entrez/viewer.fcgi?db=nucleotide&id=NP_000079.2): p.G626S

ccccgtgactgtagtgttctctctgtgcag(G/A)GTCCCGCTGGCGAGAGAGGTGAACAAGGCC

[NM_000088.3](http://www.ncbi.nlm.nih.gov/entrez/viewer.fcgi?db=nucleotide&id=NM_000088.3): c.1885G>A; [NP_000079.2](http://www.ncbi.nlm.nih.gov/entrez/viewer.fcgi?db=nucleotide&id=NP_000079.2): p.G629S

tgtagtgttctctctgtgcagGGTCCCGCT(G/A)GCGAGAGAGGTGAACAAGGCCCTGCTGGCT

[NM_000088.3](http://www.ncbi.nlm.nih.gov/entrez/viewer.fcgi?db=nucleotide&id=NM_000088.3): c.1903G>A; [NP_000079.2](http://www.ncbi.nlm.nih.gov/entrez/viewer.fcgi?db=nucleotide&id=NP_000079.2): p.G635S

cagGGTCCCGCTGGCGAGAGAGGTGAACAA(G/A)GCCCTGCTGGCTCCCCCGGATTCCAGgtga

[NM_000088.3](http://www.ncbi.nlm.nih.gov/entrez/viewer.fcgi?db=nucleotide&id=NM_000088.3): c.1913G>T; [NP_000079.2](http://www.ncbi.nlm.nih.gov/entrez/viewer.fcgi?db=nucleotide&id=NP_000079.2): p.G638V

CTGGCGAGAGAGGTGAACAAGGCCCTGCTG(G/T)CTCCCCCGGATTCCAGgtgaggcctcatgg

[NM_000088.3](http://www.ncbi.nlm.nih.gov/entrez/viewer.fcgi?db=nucleotide&id=NM_000088.3): c.1939G>A; [NP_000079.2](http://www.ncbi.nlm.nih.gov/entrez/viewer.fcgi?db=nucleotide&id=NP_000079.2): p.G647S

tcccccatttcccacctacagGGTCTCCCT(G/A)GTCCTGCTGGTCCTCCAGGTGAAGCAGGCA

[NM_000088.3](http://www.ncbi.nlm.nih.gov/entrez/viewer.fcgi?db=nucleotide&id=NM_000088.3): c.1948G>C; [NP_000079.2](http://www.ncbi.nlm.nih.gov/entrez/viewer.fcgi?db=nucleotide&id=NP_000079.2): p.G650R

tcccacctacagGGTCTCCCTGGTCCTGCT(G/C)GTCCTCCAGGTGAAGCAGGCAAACCTGGTG

[NM_000088.3](http://www.ncbi.nlm.nih.gov/entrez/viewer.fcgi?db=nucleotide&id=NM_000088.3): c.1966G>A; [NP_000079.2](http://www.ncbi.nlm.nih.gov/entrez/viewer.fcgi?db=nucleotide&id=NP_000079.2): p.G656S

CCTGGTCCTGCTGGTCCTCCAGGTGAAGCA(G/A)GCAAACCTGGTGAACAGgtaagagggagca

[NM_000088.3](http://www.ncbi.nlm.nih.gov/entrez/viewer.fcgi?db=nucleotide&id=NM_000088.3): c.1976G>C; [NP_000079.2](http://www.ncbi.nlm.nih.gov/entrez/viewer.fcgi?db=nucleotide&id=NP_000079.2): p.G659A

CTGGTCCTCCAGGTGAAGCAGGCAAACCTG(G/C)TGAACAGgtaagagggagcagccggccaga

[NM_000088.3](http://www.ncbi.nlm.nih.gov/entrez/viewer.fcgi?db=nucleotide&id=NM_000088.3): c.2002G>A; [NP_000079.2](http://www.ncbi.nlm.nih.gov/entrez/viewer.fcgi?db=nucleotide&id=NP_000079.2): p.G668S

cctccccctcagGGTGTTCCTGGAGACCTT(G/A)GCGCCCCTGGCCCCTCTGGAGCAAGAgtaa

[NM_000088.3](http://www.ncbi.nlm.nih.gov/entrez/viewer.fcgi?db=nucleotide&id=NM_000088.3): c.2032G>T; [NP_000079.2](http://www.ncbi.nlm.nih.gov/entrez/viewer.fcgi?db=nucleotide&id=NP_000079.2): p.E678X

ttcacatgccctgtccttcccttctagGGC(G/T)AGAGAGGTTTCCCTGGCGAGCGTGGTGTGC

[NM_000088.3](http://www.ncbi.nlm.nih.gov/entrez/viewer.fcgi?db=nucleotide&id=NM_000088.3): c.2047G>A; [NP_000079.2](http://www.ncbi.nlm.nih.gov/entrez/viewer.fcgi?db=nucleotide&id=NP_000079.2): p.G683S

cttcccttctagGGCGAGAGAGGTTTCCCT(G/A)GCGAGCGTGGTGTGCAAGGTCCCCCTGGTC

[NM_000088.3](http://www.ncbi.nlm.nih.gov/entrez/viewer.fcgi?db=nucleotide&id=NM_000088.3): c.2057G>C; [NP_000079.2](http://www.ncbi.nlm.nih.gov/entrez/viewer.fcgi?db=nucleotide&id=NP_000079.2): p.G686A

agGGCGAGAGAGGTTTCCCTGGCGAGCGTG(G/C)TGTGCAAGGTCCCCCTGGTCCTGCTGGTCC

[NM_000088.3](http://www.ncbi.nlm.nih.gov/entrez/viewer.fcgi?db=nucleotide&id=NM_000088.3): c.2057G>T; [NP_000079.2](http://www.ncbi.nlm.nih.gov/entrez/viewer.fcgi?db=nucleotide&id=NP_000079.2): p.G686V

agGGCGAGAGAGGTTTCCCTGGCGAGCGTG(G/T)TGTGCAAGGTCCCCCTGGTCCTGCTGGTCC

[NM_000088.3](http://www.ncbi.nlm.nih.gov/entrez/viewer.fcgi?db=nucleotide&id=NM_000088.3): c.2065G>A; [NP_000079.2](http://www.ncbi.nlm.nih.gov/entrez/viewer.fcgi?db=nucleotide&id=NP_000079.2): p.G689S

AGAGGTTTCCCTGGCGAGCGTGGTGTGCAA(G/A)GTCCCCCTGGTCCTGCTGGTCCCCGAGGGG

[NM_000088.3](http://www.ncbi.nlm.nih.gov/entrez/viewer.fcgi?db=nucleotide&id=NM_000088.3): c.2089C>T; [NP_000079.2](http://www.ncbi.nlm.nih.gov/entrez/viewer.fcgi?db=nucleotide&id=NP_000079.2): p.R697X

GTGCAAGGTCCCCCTGGTCCTGCTGGTCCC(C/T)GAGGGGCCAACGGTGCTCCCGGCAACGATG

[NM_000088.3](http://www.ncbi.nlm.nih.gov/entrez/viewer.fcgi?db=nucleotide&id=NM_000088.3): c.2101G>T; [NP_000079.2](http://www.ncbi.nlm.nih.gov/entrez/viewer.fcgi?db=nucleotide&id=NP_000079.2): p.G701C

CCTGGTCCTGCTGGTCCCCGAGGGGCCAAC(G/T)GTGCTCCCGGCAACGATGGTGCTAAGgtga

[NM_000088.3](http://www.ncbi.nlm.nih.gov/entrez/viewer.fcgi?db=nucleotide&id=NM_000088.3): c.2101G>A; [NP_000079.2](http://www.ncbi.nlm.nih.gov/entrez/viewer.fcgi?db=nucleotide&id=NP_000079.2): p.G701S

CCTGGTCCTGCTGGTCCCCGAGGGGCCAAC(G/A)GTGCTCCCGGCAACGATGGTGCTAAGgtga

[NM_000088.3](http://www.ncbi.nlm.nih.gov/entrez/viewer.fcgi?db=nucleotide&id=NM_000088.3): c.2110G>T; [NP_000079.2](http://www.ncbi.nlm.nih.gov/entrez/viewer.fcgi?db=nucleotide&id=NP_000079.2): p.G704C

GCTGGTCCCCGAGGGGCCAACGGTGCTCCC(G/T)GCAACGATGGTGCTAAGgtgagggcagcgt

[NM_000088.3](http://www.ncbi.nlm.nih.gov/entrez/viewer.fcgi?db=nucleotide&id=NM_000088.3): c.2110G>A; [NP_000079.2](http://www.ncbi.nlm.nih.gov/entrez/viewer.fcgi?db=nucleotide&id=NP_000079.2): p.G704S

GCTGGTCCCCGAGGGGCCAACGGTGCTCCC(G/A)GCAACGATGGTGCTAAGgtgagggcagcgt

[NM_000088.3](http://www.ncbi.nlm.nih.gov/entrez/viewer.fcgi?db=nucleotide&id=NM_000088.3): c.2156G>A; [NP_000079.2](http://www.ncbi.nlm.nih.gov/entrez/viewer.fcgi?db=nucleotide&id=NP_000079.2): p.G719D

agGGTGATGCTGGTGCCCCTGGAGCTCCCG(G/A)TAGCCAGGGCGCCCCTGGCCTTCAGGGAAT

[NM_000088.3](http://www.ncbi.nlm.nih.gov/entrez/viewer.fcgi?db=nucleotide&id=NM_000088.3): c.2155G>A; [NP_000079.2](http://www.ncbi.nlm.nih.gov/entrez/viewer.fcgi?db=nucleotide&id=NP_000079.2): p.G719S

tagGGTGATGCTGGTGCCCCTGGAGCTCCC(G/A)GTAGCCAGGGCGCCCCTGGCCTTCAGGGAA

[NM_000088.3](http://www.ncbi.nlm.nih.gov/entrez/viewer.fcgi?db=nucleotide&id=NM_000088.3): c.2164G>A; [NP_000079.2](http://www.ncbi.nlm.nih.gov/entrez/viewer.fcgi?db=nucleotide&id=NP_000079.2): p.G722S

GCTGGTGCCCCTGGAGCTCCCGGTAGCCAG(G/A)GCGCCCCTGGCCTTCAGGGAATGCCTGGTG

[NM_000088.3](http://www.ncbi.nlm.nih.gov/entrez/viewer.fcgi?db=nucleotide&id=NM_000088.3): c.2182G>A; [NP_000079.2](http://www.ncbi.nlm.nih.gov/entrez/viewer.fcgi?db=nucleotide&id=NP_000079.2): p.G728R

CCCGGTAGCCAGGGCGCCCCTGGCCTTCAG(G/A)GAATGCCTGGTGAACGTGGTGCAGCTGGTC

[NM_000088.3](http://www.ncbi.nlm.nih.gov/entrez/viewer.fcgi?db=nucleotide&id=NM_000088.3): c.2192G>T; [NP_000079.2](http://www.ncbi.nlm.nih.gov/entrez/viewer.fcgi?db=nucleotide&id=NP_000079.2): p.G731V

AGGGCGCCCCTGGCCTTCAGGGAATGCCTG(G/T)TGAACGTGGTGCAGCTGGTCTTCCAGGGCC

[NM_000088.3](http://www.ncbi.nlm.nih.gov/entrez/viewer.fcgi?db=nucleotide&id=NM_000088.3): c.2201G>T; [NP_000079.2](http://www.ncbi.nlm.nih.gov/entrez/viewer.fcgi?db=nucleotide&id=NP_000079.2): p.G734V

CTGGCCTTCAGGGAATGCCTGGTGAACGTG(G/T)TGCAGCTGGTCTTCCAGGGCCTAAGGGTGA

[NM_000088.3](http://www.ncbi.nlm.nih.gov/entrez/viewer.fcgi?db=nucleotide&id=NM_000088.3): c.2210G>A; [NP_000079.2](http://www.ncbi.nlm.nih.gov/entrez/viewer.fcgi?db=nucleotide&id=NP_000079.2): p.G737D

AGGGAATGCCTGGTGAACGTGGTGCAGCTG(G/A)TCTTCCAGGGCCTAAGGGTGACAGAgtaag

[NM_000088.3](http://www.ncbi.nlm.nih.gov/entrez/viewer.fcgi?db=nucleotide&id=NM_000088.3): c.2209G>T; [NP_000079.2](http://www.ncbi.nlm.nih.gov/entrez/viewer.fcgi?db=nucleotide&id=NP_000079.2): p.G737C

CAGGGAATGCCTGGTGAACGTGGTGCAGCT(G/T)GTCTTCCAGGGCCTAAGGGTGACAGAgtaa

[NM_000088.3](http://www.ncbi.nlm.nih.gov/entrez/viewer.fcgi?db=nucleotide&id=NM_000088.3): c.2218G>C; [NP_000079.2](http://www.ncbi.nlm.nih.gov/entrez/viewer.fcgi?db=nucleotide&id=NP_000079.2): p.G740R

CCTGGTGAACGTGGTGCAGCTGGTCTTCCA(G/C)GGCCTAAGGGTGACAGAgtaagttcaacct

[NM_000088.3](http://www.ncbi.nlm.nih.gov/entrez/viewer.fcgi?db=nucleotide&id=NM_000088.3): c.2228G>A; [NP_000079.2](http://www.ncbi.nlm.nih.gov/entrez/viewer.fcgi?db=nucleotide&id=NP_000079.2): p.G743D

GTGGTGCAGCTGGTCTTCCAGGGCCTAAGG(G/A)TGACAGAgtaagttcaaccttccccctccc

[NM_000088.3](http://www.ncbi.nlm.nih.gov/entrez/viewer.fcgi?db=nucleotide&id=NM_000088.3): c.2227G>A; [NP_000079.2](http://www.ncbi.nlm.nih.gov/entrez/viewer.fcgi?db=nucleotide&id=NP_000079.2): p.G743S

CGTGGTGCAGCTGGTCTTCCAGGGCCTAAG(G/A)GTGACAGAgtaagttcaaccttccccctcc

[NM_000088.3](http://www.ncbi.nlm.nih.gov/entrez/viewer.fcgi?db=nucleotide&id=NM_000088.3): c.2228G>T; [NP_000079.2](http://www.ncbi.nlm.nih.gov/entrez/viewer.fcgi?db=nucleotide&id=NP_000079.2): p.G743V

GTGGTGCAGCTGGTCTTCCAGGGCCTAAGG(G/T)TGACAGAgtaagttcaaccttccccctccc

[NM_000088.3](http://www.ncbi.nlm.nih.gov/entrez/viewer.fcgi?db=nucleotide&id=NM_000088.3): c.2263G>A; [NP_000079.2](http://www.ncbi.nlm.nih.gov/entrez/viewer.fcgi?db=nucleotide&id=NP_000079.2): p.G755S

tagGGTGATGCTGGTCCCAAAGGTGCTGAT(G/A)GCTCTCCTGGCAAAGATGGCGTCCGTGGTC

[NM_000088.3](http://www.ncbi.nlm.nih.gov/entrez/viewer.fcgi?db=nucleotide&id=NM_000088.3): c.2272G>T; [NP_000079.2](http://www.ncbi.nlm.nih.gov/entrez/viewer.fcgi?db=nucleotide&id=NP_000079.2): p.G758C

GCTGGTCCCAAAGGTGCTGATGGCTCTCCT(G/T)GCAAAGATGGCGTCCGTGGTCTGACTGGCC

[NM_000088.3](http://www.ncbi.nlm.nih.gov/entrez/viewer.fcgi?db=nucleotide&id=NM_000088.3): c.2290G>A; [NP_000079.2](http://www.ncbi.nlm.nih.gov/entrez/viewer.fcgi?db=nucleotide&id=NP_000079.2): p.G764S

GATGGCTCTCCTGGCAAAGATGGCGTCCGT(G/A)GTCTGACTGGCCCCATTGGTCCTCCTGGCC

[NM_000088.3](http://www.ncbi.nlm.nih.gov/entrez/viewer.fcgi?db=nucleotide&id=NM_000088.3): c.2291G>T; [NP_000079.2](http://www.ncbi.nlm.nih.gov/entrez/viewer.fcgi?db=nucleotide&id=NP_000079.2): p.G764V

ATGGCTCTCCTGGCAAAGATGGCGTCCGTG(G/T)TCTGACTGGCCCCATTGGTCCTCCTGGCCC

[NM_000088.3](http://www.ncbi.nlm.nih.gov/entrez/viewer.fcgi?db=nucleotide&id=NM_000088.3): c.2299G>A; [NP_000079.2](http://www.ncbi.nlm.nih.gov/entrez/viewer.fcgi?db=nucleotide&id=NP_000079.2): p.G767S

CCTGGCAAAGATGGCGTCCGTGGTCTGACT(G/A)GCCCCATTGGTCCTCCTGGCCCTGCTGGTG

[NM_000088.3](http://www.ncbi.nlm.nih.gov/entrez/viewer.fcgi?db=nucleotide&id=NM_000088.3): c.2308G>T; [NP_000079.2](http://www.ncbi.nlm.nih.gov/entrez/viewer.fcgi?db=nucleotide&id=NP_000079.2): p.G770C

GATGGCGTCCGTGGTCTGACTGGCCCCATT(G/T)GTCCTCCTGGCCCTGCTGGTGCCCCTGGTG

[NM_000088.3](http://www.ncbi.nlm.nih.gov/entrez/viewer.fcgi?db=nucleotide&id=NM_000088.3): c.2308G>A; [NP_000079.2](http://www.ncbi.nlm.nih.gov/entrez/viewer.fcgi?db=nucleotide&id=NP_000079.2): p.G770S

GATGGCGTCCGTGGTCTGACTGGCCCCATT(G/A)GTCCTCCTGGCCCTGCTGGTGCCCCTGGTG

[NM_000088.3](http://www.ncbi.nlm.nih.gov/entrez/viewer.fcgi?db=nucleotide&id=NM_000088.3): c.2317G>A; [NP_000079.2](http://www.ncbi.nlm.nih.gov/entrez/viewer.fcgi?db=nucleotide&id=NP_000079.2): p.G773S

CGTGGTCTGACTGGCCCCATTGGTCCTCCT(G/A)GCCCTGCTGGTGCCCCTGGTGACAAGgtga

[NM_000088.3](http://www.ncbi.nlm.nih.gov/entrez/viewer.fcgi?db=nucleotide&id=NM_000088.3): c.2326G>A; [NP_000079.2](http://www.ncbi.nlm.nih.gov/entrez/viewer.fcgi?db=nucleotide&id=NP_000079.2): p.G776S

ACTGGCCCCATTGGTCCTCCTGGCCCTGCT(G/A)GTGCCCCTGGTGACAAGgtgaggtggccgc

[NM_000088.3](http://www.ncbi.nlm.nih.gov/entrez/viewer.fcgi?db=nucleotide&id=NM_000088.3): c.2335G>A; [NP_000079.2](http://www.ncbi.nlm.nih.gov/entrez/viewer.fcgi?db=nucleotide&id=NP_000079.2): p.G779S

ATTGGTCCTCCTGGCCCTGCTGGTGCCCCT(G/A)GTGACAAGgtgaggtggccgcctccccacc

[NM_000088.3](http://www.ncbi.nlm.nih.gov/entrez/viewer.fcgi?db=nucleotide&id=NM_000088.3): c.2362G>T; [NP_000079.2](http://www.ncbi.nlm.nih.gov/entrez/viewer.fcgi?db=nucleotide&id=NP_000079.2): p.G788C

ccttgctctcagGGTGAAAGTGGTCCCAGC(G/T)GCCCTGCTGGTCCCACTGGAGCTCGTGGTG

[NM_000088.3](http://www.ncbi.nlm.nih.gov/entrez/viewer.fcgi?db=nucleotide&id=NM_000088.3): c.2362G>A; [NP_000079.2](http://www.ncbi.nlm.nih.gov/entrez/viewer.fcgi?db=nucleotide&id=NP_000079.2): p.G788S

ccttgctctcagGGTGAAAGTGGTCCCAGC(G/A)GCCCTGCTGGTCCCACTGGAGCTCGTGGTG

[NM_000088.3](http://www.ncbi.nlm.nih.gov/entrez/viewer.fcgi?db=nucleotide&id=NM_000088.3): c.2387G>A; [NP_000079.2](http://www.ncbi.nlm.nih.gov/entrez/viewer.fcgi?db=nucleotide&id=NP_000079.2): p.R796H

CCAGCGGCCCTGCTGGTCCCACTGGAGCTC(G/A)TGGTGCCCCCgtaagtacagaagacctgtt

[NM_000088.3](http://www.ncbi.nlm.nih.gov/entrez/viewer.fcgi?db=nucleotide&id=NM_000088.3): c.2425G>A; [NP_000079.2](http://www.ncbi.nlm.nih.gov/entrez/viewer.fcgi?db=nucleotide&id=NP_000079.2): p.G809S

tagGGAGACCGTGGTGAGCCTGGTCCCCCC(G/A)GCCCTGCTGGCTTTGCTGGCCCCCCTgtga

[NM_000088.3](http://www.ncbi.nlm.nih.gov/entrez/viewer.fcgi?db=nucleotide&id=NM_000088.3): c.2444G>C; [NP_000079.2](http://www.ncbi.nlm.nih.gov/entrez/viewer.fcgi?db=nucleotide&id=NP_000079.2): p.G815A

CTGGTCCCCCCGGCCCTGCTGGCTTTGCTG(G/C)CCCCCCTgtgagtaccaagacccccatcat

[NM_000088.3](http://www.ncbi.nlm.nih.gov/entrez/viewer.fcgi?db=nucleotide&id=NM_000088.3): c.2444G>T; [NP_000079.2](http://www.ncbi.nlm.nih.gov/entrez/viewer.fcgi?db=nucleotide&id=NP_000079.2): p.G815V

CTGGTCCCCCCGGCCCTGCTGGCTTTGCTG(G/T)CCCCCCTgtgagtaccaagacccccatcat

[NM_000088.3](http://www.ncbi.nlm.nih.gov/entrez/viewer.fcgi?db=nucleotide&id=NM_000088.3): c.2461G>C; [NP_000079.2](http://www.ncbi.nlm.nih.gov/entrez/viewer.fcgi?db=nucleotide&id=NP_000079.2): p.G821R

ccgccctgcttcctcccccagGGTGCTGAC(G/C)GCCAACCTGGTGCTAAAGGCGAACCTGGTG

[NM_000088.3](http://www.ncbi.nlm.nih.gov/entrez/viewer.fcgi?db=nucleotide&id=NM_000088.3): c.2461G>A; [NP_000079.2](http://www.ncbi.nlm.nih.gov/entrez/viewer.fcgi?db=nucleotide&id=NP_000079.2): p.G821S

ccgccctgcttcctcccccagGGTGCTGAC(G/A)GCCAACCTGGTGCTAAAGGCGAACCTGGTG

[NM_000088.3](http://www.ncbi.nlm.nih.gov/entrez/viewer.fcgi?db=nucleotide&id=NM_000088.3): c.2464C>T; [NP_000079.2](http://www.ncbi.nlm.nih.gov/entrez/viewer.fcgi?db=nucleotide&id=NP_000079.2): p.Q822X

ccctgcttcctcccccagGGTGCTGACGGC(C/T)AACCTGGTGCTAAAGGCGAACCTGGTGATG

[NM_000088.3](http://www.ncbi.nlm.nih.gov/entrez/viewer.fcgi?db=nucleotide&id=NM_000088.3): c.2471G>C; [NP_000079.2](http://www.ncbi.nlm.nih.gov/entrez/viewer.fcgi?db=nucleotide&id=NP_000079.2): p.G824A

tcctcccccagGGTGCTGACGGCCAACCTG(G/C)TGCTAAAGGCGAACCTGGTGATGCTGGTGC

[NM_000088.3](http://www.ncbi.nlm.nih.gov/entrez/viewer.fcgi?db=nucleotide&id=NM_000088.3): c.2470G>C; [NP_000079.2](http://www.ncbi.nlm.nih.gov/entrez/viewer.fcgi?db=nucleotide&id=NP_000079.2): p.G824R

ttcctcccccagGGTGCTGACGGCCAACCT(G/C)GTGCTAAAGGCGAACCTGGTGATGCTGGTG

[NM_000088.3](http://www.ncbi.nlm.nih.gov/entrez/viewer.fcgi?db=nucleotide&id=NM_000088.3): c.2470G>T; [NP_000079.2](http://www.ncbi.nlm.nih.gov/entrez/viewer.fcgi?db=nucleotide&id=NP_000079.2): p.G824C

ttcctcccccagGGTGCTGACGGCCAACCT(G/T)GTGCTAAAGGCGAACCTGGTGATGCTGGTG

[NM_000088.3](http://www.ncbi.nlm.nih.gov/entrez/viewer.fcgi?db=nucleotide&id=NM_000088.3): c.2480G>A; [NP_000079.2](http://www.ncbi.nlm.nih.gov/entrez/viewer.fcgi?db=nucleotide&id=NP_000079.2): p.G827D

agGGTGCTGACGGCCAACCTGGTGCTAAAG(G/A)CGAACCTGGTGATGCTGGTGCTAAAGGCGA

[NM_000088.3](http://www.ncbi.nlm.nih.gov/entrez/viewer.fcgi?db=nucleotide&id=NM_000088.3): c.2498G>A; [NP_000079.2](http://www.ncbi.nlm.nih.gov/entrez/viewer.fcgi?db=nucleotide&id=NP_000079.2): p.G833D

CTGGTGCTAAAGGCGAACCTGGTGATGCTG(G/A)TGCTAAAGGCGATGCTGGTCCCCCTGGCCC

[NM_000088.3](http://www.ncbi.nlm.nih.gov/entrez/viewer.fcgi?db=nucleotide&id=NM_000088.3): c.2498G>T; [NP_000079.2](http://www.ncbi.nlm.nih.gov/entrez/viewer.fcgi?db=nucleotide&id=NP_000079.2): p.G833V

CTGGTGCTAAAGGCGAACCTGGTGATGCTG(G/T)TGCTAAAGGCGATGCTGGTCCCCCTGGCCC

[NM_000088.3](http://www.ncbi.nlm.nih.gov/entrez/viewer.fcgi?db=nucleotide&id=NM_000088.3): c.2507G>C; [NP_000079.2](http://www.ncbi.nlm.nih.gov/entrez/viewer.fcgi?db=nucleotide&id=NP_000079.2): p.G836A

AAGGCGAACCTGGTGATGCTGGTGCTAAAG(G/C)CGATGCTGGTCCCCCTGGCCCTGCCGGACC

[NM_000088.3](http://www.ncbi.nlm.nih.gov/entrez/viewer.fcgi?db=nucleotide&id=NM_000088.3): c.2515G>A; [NP_000079.2](http://www.ncbi.nlm.nih.gov/entrez/viewer.fcgi?db=nucleotide&id=NP_000079.2): p.G839S

CCTGGTGATGCTGGTGCTAAAGGCGATGCT(G/A)GTCCCCCTGGCCCTGCCGGACCCGCTGGAC

[NM_000088.3](http://www.ncbi.nlm.nih.gov/entrez/viewer.fcgi?db=nucleotide&id=NM_000088.3): c.2524G>C; [NP_000079.2](http://www.ncbi.nlm.nih.gov/entrez/viewer.fcgi?db=nucleotide&id=NP_000079.2): p.G842R

GCTGGTGCTAAAGGCGATGCTGGTCCCCCT(G/C)GCCCTGCCGGACCCGCTGGACCCCCTGGCC

[NM_000088.3](http://www.ncbi.nlm.nih.gov/entrez/viewer.fcgi?db=nucleotide&id=NM_000088.3): c.2525G>A; [NP_000079.2](http://www.ncbi.nlm.nih.gov/entrez/viewer.fcgi?db=nucleotide&id=NP_000079.2): p.G842D

CTGGTGCTAAAGGCGATGCTGGTCCCCCTG(G/A)CCCTGCCGGACCCGCTGGACCCCCTGGCCC

[NM_000088.3](http://www.ncbi.nlm.nih.gov/entrez/viewer.fcgi?db=nucleotide&id=NM_000088.3): c.2533G>A; [NP_000079.2](http://www.ncbi.nlm.nih.gov/entrez/viewer.fcgi?db=nucleotide&id=NP_000079.2): p.G845R

AAAGGCGATGCTGGTCCCCCTGGCCCTGCC(G/A)GACCCGCTGGACCCCCTGGCCCCATTgtga

[NM_000088.3](http://www.ncbi.nlm.nih.gov/entrez/viewer.fcgi?db=nucleotide&id=NM_000088.3): c.2533G>C; [NP_000079.2](http://www.ncbi.nlm.nih.gov/entrez/viewer.fcgi?db=nucleotide&id=NP_000079.2): p.G845R

AAAGGCGATGCTGGTCCCCCTGGCCCTGCC(G/C)GACCCGCTGGACCCCCTGGCCCCATTgtga

[NM_000088.3](http://www.ncbi.nlm.nih.gov/entrez/viewer.fcgi?db=nucleotide&id=NM_000088.3): c.2542G>C; [NP_000079.2](http://www.ncbi.nlm.nih.gov/entrez/viewer.fcgi?db=nucleotide&id=NP_000079.2): p.G848R

GCTGGTCCCCCTGGCCCTGCCGGACCCGCT(G/C)GACCCCCTGGCCCCATTgtgagtggcttgg

[NM_000088.3](http://www.ncbi.nlm.nih.gov/entrez/viewer.fcgi?db=nucleotide&id=NM_000088.3): c.2552G>A; [NP_000079.2](http://www.ncbi.nlm.nih.gov/entrez/viewer.fcgi?db=nucleotide&id=NP_000079.2): p.G851D

CTGGCCCTGCCGGACCCGCTGGACCCCCTG(G/A)CCCCATTgtgagtggcttggccctctgtgc

[NM_000088.3](http://www.ncbi.nlm.nih.gov/entrez/viewer.fcgi?db=nucleotide&id=NM_000088.3): c.2560G>A; [NP_000079.2](http://www.ncbi.nlm.nih.gov/entrez/viewer.fcgi?db=nucleotide&id=NP_000079.2): p.G854S

caggcttgatgcctctgtgctctcctacag(G/A)GTAATGTTGGTGCTCCTGGAGCCAAAGGTG

[NM_000088.3](http://www.ncbi.nlm.nih.gov/entrez/viewer.fcgi?db=nucleotide&id=NM_000088.3): c.2563A>C; [NP_000079.2](http://www.ncbi.nlm.nih.gov/entrez/viewer.fcgi?db=nucleotide&id=NP_000079.2): p.N855H

gcttgatgcctctgtgctctcctacagGGT(A/C)ATGTTGGTGCTCCTGGAGCCAAAGGTGCTC

[NM_000088.3](http://www.ncbi.nlm.nih.gov/entrez/viewer.fcgi?db=nucleotide&id=NM_000088.3): c.2569G>T; [NP_000079.2](http://www.ncbi.nlm.nih.gov/entrez/viewer.fcgi?db=nucleotide&id=NP_000079.2): p.G857C

tgcctctgtgctctcctacagGGTAATGTT(G/T)GTGCTCCTGGAGCCAAAGGTGCTCGCGGCA

[NM_000088.3](http://www.ncbi.nlm.nih.gov/entrez/viewer.fcgi?db=nucleotide&id=NM_000088.3): c.2579G>C; [NP_000079.2](http://www.ncbi.nlm.nih.gov/entrez/viewer.fcgi?db=nucleotide&id=NP_000079.2): p.G860A

ctctcctacagGGTAATGTTGGTGCTCCTG(G/C)AGCCAAAGGTGCTCGCGGCAGCGCTGGTCC

[NM_000088.3](http://www.ncbi.nlm.nih.gov/entrez/viewer.fcgi?db=nucleotide&id=NM_000088.3): c.2588G>T; [NP_000079.2](http://www.ncbi.nlm.nih.gov/entrez/viewer.fcgi?db=nucleotide&id=NP_000079.2): p.G863V

agGGTAATGTTGGTGCTCCTGGAGCCAAAG(G/T)TGCTCGCGGCAGCGCTGGTCCCCCTgtgag

[NM_000088.3](http://www.ncbi.nlm.nih.gov/entrez/viewer.fcgi?db=nucleotide&id=NM_000088.3): c.2596G>T; [NP_000079.2](http://www.ncbi.nlm.nih.gov/entrez/viewer.fcgi?db=nucleotide&id=NP_000079.2): p.G866C

GTTGGTGCTCCTGGAGCCAAAGGTGCTCGC(G/T)GCAGCGCTGGTCCCCCTgtgagtatcaccc

[NM_000088.3](http://www.ncbi.nlm.nih.gov/entrez/viewer.fcgi?db=nucleotide&id=NM_000088.3): c.2596G>A; [NP_000079.2](http://www.ncbi.nlm.nih.gov/entrez/viewer.fcgi?db=nucleotide&id=NP_000079.2): p.G866S

GTTGGTGCTCCTGGAGCCAAAGGTGCTCGC(G/A)GCAGCGCTGGTCCCCCTgtgagtatcaccc

[NM_000088.3](http://www.ncbi.nlm.nih.gov/entrez/viewer.fcgi?db=nucleotide&id=NM_000088.3): c.2605G>T; [NP_000079.2](http://www.ncbi.nlm.nih.gov/entrez/viewer.fcgi?db=nucleotide&id=NP_000079.2): p.G869C

CCTGGAGCCAAAGGTGCTCGCGGCAGCGCT(G/T)GTCCCCCTgtgagtatcacccgcctctctg

[NM_000088.3](http://www.ncbi.nlm.nih.gov/entrez/viewer.fcgi?db=nucleotide&id=NM_000088.3): c.2623G>A; [NP_000079.2](http://www.ncbi.nlm.nih.gov/entrez/viewer.fcgi?db=nucleotide&id=NP_000079.2): p.G875S

tcctatgttctctccttccagGGTGCTACT(G/A)GTTTCCCTGGTGCTGCTGGCCGAGTCGGTC

[NM_000088.3](http://www.ncbi.nlm.nih.gov/entrez/viewer.fcgi?db=nucleotide&id=NM_000088.3): c.2632G>A; [NP_000079.2](http://www.ncbi.nlm.nih.gov/entrez/viewer.fcgi?db=nucleotide&id=NP_000079.2): p.G878S

ctctccttccagGGTGCTACTGGTTTCCCT(G/A)GTGCTGCTGGCCGAGTCGGTCCTCCTGGCC

[NM_000088.3](http://www.ncbi.nlm.nih.gov/entrez/viewer.fcgi?db=nucleotide&id=NM_000088.3): c.2644C>T; [NP_000079.2](http://www.ncbi.nlm.nih.gov/entrez/viewer.fcgi?db=nucleotide&id=NP_000079.2): p.R882X

GGTGCTACTGGTTTCCCTGGTGCTGCTGGC(C/T)GAGTCGGTCCTCCTGGCCCCTCTgtaagtc

[NM_000088.3](http://www.ncbi.nlm.nih.gov/entrez/viewer.fcgi?db=nucleotide&id=NM_000088.3): c.2650G>A; [NP_000079.2](http://www.ncbi.nlm.nih.gov/entrez/viewer.fcgi?db=nucleotide&id=NP_000079.2): p.G884S

ACTGGTTTCCCTGGTGCTGCTGGCCGAGTC(G/A)GTCCTCCTGGCCCCTCTgtaagtctctgca

[NM_000088.3](http://www.ncbi.nlm.nih.gov/entrez/viewer.fcgi?db=nucleotide&id=NM_000088.3): c.2659G>A; [NP_000079.2](http://www.ncbi.nlm.nih.gov/entrez/viewer.fcgi?db=nucleotide&id=NP_000079.2): p.G887S

CCTGGTGCTGCTGGCCGAGTCGGTCCTCCT(G/A)GCCCCTCTgtaagtctctgcagcagagtcc

[NM_000088.3](http://www.ncbi.nlm.nih.gov/entrez/viewer.fcgi?db=nucleotide&id=NM_000088.3): c.2687G>A; [NP_000079.2](http://www.ncbi.nlm.nih.gov/entrez/viewer.fcgi?db=nucleotide&id=NP_000079.2): p.G896D

tcctcatccagGGAAATGCTGGACCCCCTG(G/A)CCCTCCTGGTCCTGCTGGCAAAGAAGGCGG

[NM_000088.3](http://www.ncbi.nlm.nih.gov/entrez/viewer.fcgi?db=nucleotide&id=NM_000088.3): c.2686G>T; [NP_000079.2](http://www.ncbi.nlm.nih.gov/entrez/viewer.fcgi?db=nucleotide&id=NP_000079.2): p.G896C

atcctcatccagGGAAATGCTGGACCCCCT(G/T)GCCCTCCTGGTCCTGCTGGCAAAGAAGGCG

[NM_000088.3](http://www.ncbi.nlm.nih.gov/entrez/viewer.fcgi?db=nucleotide&id=NM_000088.3): c.2750G>A; [NP_000079.2](http://www.ncbi.nlm.nih.gov/entrez/viewer.fcgi?db=nucleotide&id=NP_000079.2): p.G917E

AAGGTCCCCGTGGTGAGACTGGCCCTGCTG(G/A)ACGTCCTGGTGAAGTTGGTCCCCCTGGTCC

[NM_000088.3](http://www.ncbi.nlm.nih.gov/entrez/viewer.fcgi?db=nucleotide&id=NM_000088.3): c.2776G>T; [NP_000079.2](http://www.ncbi.nlm.nih.gov/entrez/viewer.fcgi?db=nucleotide&id=NP_000079.2): p.G926C

GCTGGACGTCCTGGTGAAGTTGGTCCCCCT(G/T)GTCCCCCTGGCCCTGCTGGCGAGAAAGGAT

[NM_000088.3](http://www.ncbi.nlm.nih.gov/entrez/viewer.fcgi?db=nucleotide&id=NM_000088.3): c.2830G>T; [NP_000079.2](http://www.ncbi.nlm.nih.gov/entrez/viewer.fcgi?db=nucleotide&id=NP_000079.2): p.G944C

tggcacctcacccctgtttgcctcccaaag(G/T)GTGCTCCTGGTACTCCCGGGCCTCAAGGTA

[NM_000088.3](http://www.ncbi.nlm.nih.gov/entrez/viewer.fcgi?db=nucleotide&id=NM_000088.3): c.2839G>T; [NP_000079.2](http://www.ncbi.nlm.nih.gov/entrez/viewer.fcgi?db=nucleotide&id=NP_000079.2): p.G947C

acccctgtttgcctcccaaagGGTGCTCCT(G/T)GTACTCCCGGGCCTCAAGGTATTGCTGGAC

[NM_000088.3](http://www.ncbi.nlm.nih.gov/entrez/viewer.fcgi?db=nucleotide&id=NM_000088.3): c.2848G>A; [NP_000079.2](http://www.ncbi.nlm.nih.gov/entrez/viewer.fcgi?db=nucleotide&id=NP_000079.2): p.G950R

tgcctcccaaagGGTGCTCCTGGTACTCCC(G/A)GGCCTCAAGGTATTGCTGGACAGCGTGGTG

[NM_000088.3](http://www.ncbi.nlm.nih.gov/entrez/viewer.fcgi?db=nucleotide&id=NM_000088.3): c.2869C>T; [NP_000079.2](http://www.ncbi.nlm.nih.gov/entrez/viewer.fcgi?db=nucleotide&id=NP_000079.2): p.Q957X

GGTACTCCCGGGCCTCAAGGTATTGCTGGA(C/T)AGCGTGGTGTGGTCGGCCTGCCTGGTCAGA

[NM_000088.3](http://www.ncbi.nlm.nih.gov/entrez/viewer.fcgi?db=nucleotide&id=NM_000088.3): c.2885G>A; [NP_000079.2](http://www.ncbi.nlm.nih.gov/entrez/viewer.fcgi?db=nucleotide&id=NP_000079.2): p.G962D

AAGGTATTGCTGGACAGCGTGGTGTGGTCG(G/A)CCTGCCTGGTCAGAGAGGAGAGAGAGGCTT

[NM_000088.3](http://www.ncbi.nlm.nih.gov/entrez/viewer.fcgi?db=nucleotide&id=NM_000088.3): c.2921G>C; [NP_000079.2](http://www.ncbi.nlm.nih.gov/entrez/viewer.fcgi?db=nucleotide&id=NP_000079.2): p.G974A

CTGGTCAGAGAGGAGAGAGAGGCTTCCCTG(G/C)TCTTCCTGGCCCCTCTgtaagtgcccccct

[NM_000088.3](http://www.ncbi.nlm.nih.gov/entrez/viewer.fcgi?db=nucleotide&id=NM_000088.3): c.2930G>A; [NP_000079.2](http://www.ncbi.nlm.nih.gov/entrez/viewer.fcgi?db=nucleotide&id=NP_000079.2): p.G977D

GAGGAGAGAGAGGCTTCCCTGGTCTTCCTG(G/A)CCCCTCTgtaagtgcccccctcaccttggg

[NM_000088.3](http://www.ncbi.nlm.nih.gov/entrez/viewer.fcgi?db=nucleotide&id=NM_000088.3): c.2939G>T; [NP_000079.2](http://www.ncbi.nlm.nih.gov/entrez/viewer.fcgi?db=nucleotide&id=NP_000079.2): p.G980V

ctctggcctgactctttcttctcccttagG(G/T)TGAACCTGGCAAACAAGGTCCCTCTGGAGC

[NM_000088.3](http://www.ncbi.nlm.nih.gov/entrez/viewer.fcgi?db=nucleotide&id=NM_000088.3): c.3001G>T; [NP_000079.2](http://www.ncbi.nlm.nih.gov/entrez/viewer.fcgi?db=nucleotide&id=NP_000079.2): p.G1001C

AGTGGTGAACGTGGTCCCCCTGGTCCCATG(G/T)GCCCCCCTGGATTGGCTGGACCCCCTGGTG

[NM_000088.3](http://www.ncbi.nlm.nih.gov/entrez/viewer.fcgi?db=nucleotide&id=NM_000088.3): c.3028G>A; [NP_000079.2](http://www.ncbi.nlm.nih.gov/entrez/viewer.fcgi?db=nucleotide&id=NP_000079.2): p.G1010S

ATGGGCCCCCCTGGATTGGCTGGACCCCCT(G/A)GTGAATCTGGACGTGAGgtgagcagtcccc

[NM_000088.3](http://www.ncbi.nlm.nih.gov/entrez/viewer.fcgi?db=nucleotide&id=NM_000088.3): c.3040C>T; [NP_000079.2](http://www.ncbi.nlm.nih.gov/entrez/viewer.fcgi?db=nucleotide&id=NP_000079.2): p.R1014C

GGATTGGCTGGACCCCCTGGTGAATCTGGA(C/T)GTGAGgtgagcagtccccagcccccatgcc

[NM_000088.3](http://www.ncbi.nlm.nih.gov/entrez/viewer.fcgi?db=nucleotide&id=NM_000088.3): c.3065G>C; [NP_000079.2](http://www.ncbi.nlm.nih.gov/entrez/viewer.fcgi?db=nucleotide&id=NP_000079.2): p.G1022A

tctctctgcagGGGGCTCCTGGTGCCGAAG(G/C)TTCCCCTGGACGAGACGGTTCTCCTGGCGC

[NM_000088.3](http://www.ncbi.nlm.nih.gov/entrez/viewer.fcgi?db=nucleotide&id=NM_000088.3): c.3064G>A; [NP_000079.2](http://www.ncbi.nlm.nih.gov/entrez/viewer.fcgi?db=nucleotide&id=NP_000079.2): p.G1022S

ctctctctgcagGGGGCTCCTGGTGCCGAA(G/A)GTTCCCCTGGACGAGACGGTTCTCCTGGCG

[NM_000088.3](http://www.ncbi.nlm.nih.gov/entrez/viewer.fcgi?db=nucleotide&id=NM_000088.3): c.3065G>T; [NP_000079.2](http://www.ncbi.nlm.nih.gov/entrez/viewer.fcgi?db=nucleotide&id=NP_000079.2): p.G1022V

tctctctgcagGGGGCTCCTGGTGCCGAAG(G/T)TTCCCCTGGACGAGACGGTTCTCCTGGCGC

[NM_000088.3](http://www.ncbi.nlm.nih.gov/entrez/viewer.fcgi?db=nucleotide&id=NM_000088.3): c.3073G>A; [NP_000079.2](http://www.ncbi.nlm.nih.gov/entrez/viewer.fcgi?db=nucleotide&id=NP_000079.2): p.G1025R

cagGGGGCTCCTGGTGCCGAAGGTTCCCCT(G/A)GACGAGACGGTTCTCCTGGCGCCAAGgtaa

[NM_000088.3](http://www.ncbi.nlm.nih.gov/entrez/viewer.fcgi?db=nucleotide&id=NM_000088.3): c.3076C>T; [NP_000079.2](http://www.ncbi.nlm.nih.gov/entrez/viewer.fcgi?db=nucleotide&id=NP_000079.2): p.R1026X

GGGGCTCCTGGTGCCGAAGGTTCCCCTGGA(C/T)GAGACGGTTCTCCTGGCGCCAAGgtaagat

[NM_000088.3](http://www.ncbi.nlm.nih.gov/entrez/viewer.fcgi?db=nucleotide&id=NM_000088.3): c.3082G>A; [NP_000079.2](http://www.ncbi.nlm.nih.gov/entrez/viewer.fcgi?db=nucleotide&id=NP_000079.2): p.G1028S

CCTGGTGCCGAAGGTTCCCCTGGACGAGAC(G/A)GTTCTCCTGGCGCCAAGgtaagatggcaac

[NM_000088.3](http://www.ncbi.nlm.nih.gov/entrez/viewer.fcgi?db=nucleotide&id=NM_000088.3): c.3106C>T; [NP_000079.2](http://www.ncbi.nlm.nih.gov/entrez/viewer.fcgi?db=nucleotide&id=NP_000079.2): p.R1036C

atattcccctgctctccccgccagGGTGAC(C/T)GTGGTGAGACCGGCCCCGCTGGACCCCCTG

[NM_000088.3](http://www.ncbi.nlm.nih.gov/entrez/viewer.fcgi?db=nucleotide&id=NM_000088.3): c.3118G>A; [NP_000079.2](http://www.ncbi.nlm.nih.gov/entrez/viewer.fcgi?db=nucleotide&id=NP_000079.2): p.G1040S

tctccccgccagGGTGACCGTGGTGAGACC(G/A)GCCCCGCTGGACCCCCTGGTGCTCCTGGTG

[NM_000088.3](http://www.ncbi.nlm.nih.gov/entrez/viewer.fcgi?db=nucleotide&id=NM_000088.3): c.3136G>T; [NP_000079.2](http://www.ncbi.nlm.nih.gov/entrez/viewer.fcgi?db=nucleotide&id=NP_000079.2): p.G1046C

CGTGGTGAGACCGGCCCCGCTGGACCCCCT(G/T)GTGCTCCTGGTGCTCCTGGTGCCCCTGGCC

[NM_000088.3](http://www.ncbi.nlm.nih.gov/entrez/viewer.fcgi?db=nucleotide&id=NM_000088.3): c.3136G>A; [NP_000079.2](http://www.ncbi.nlm.nih.gov/entrez/viewer.fcgi?db=nucleotide&id=NP_000079.2): p.G1046S

CGTGGTGAGACCGGCCCCGCTGGACCCCCT(G/A)GTGCTCCTGGTGCTCCTGGTGCCCCTGGCC

[NM_000088.3](http://www.ncbi.nlm.nih.gov/entrez/viewer.fcgi?db=nucleotide&id=NM_000088.3): c.3145G>A; [NP_000079.2](http://www.ncbi.nlm.nih.gov/entrez/viewer.fcgi?db=nucleotide&id=NP_000079.2): p.G1049S

ACCGGCCCCGCTGGACCCCCTGGTGCTCCT(G/A)GTGCTCCTGGTGCCCCTGGCCCCGTTGGCC

[NM_000088.3](http://www.ncbi.nlm.nih.gov/entrez/viewer.fcgi?db=nucleotide&id=NM_000088.3): c.3154G>A; [NP_000079.2](http://www.ncbi.nlm.nih.gov/entrez/viewer.fcgi?db=nucleotide&id=NP_000079.2): p.G1052S

GCTGGACCCCCTGGTGCTCCTGGTGCTCCT(G/A)GTGCCCCTGGCCCCGTTGGCCCTGCTGGCA

[NM_000088.3](http://www.ncbi.nlm.nih.gov/entrez/viewer.fcgi?db=nucleotide&id=NM_000088.3): c.3164G>A; [NP_000079.2](http://www.ncbi.nlm.nih.gov/entrez/viewer.fcgi?db=nucleotide&id=NP_000079.2): p.G1055D

CTGGTGCTCCTGGTGCTCCTGGTGCCCCTG(G/A)CCCCGTTGGCCCTGCTGGCAAGAGTGGTGA

[NM_000088.3](http://www.ncbi.nlm.nih.gov/entrez/viewer.fcgi?db=nucleotide&id=NM_000088.3): c.3172G>A; [NP_000079.2](http://www.ncbi.nlm.nih.gov/entrez/viewer.fcgi?db=nucleotide&id=NP_000079.2): p.G1058S

CCTGGTGCTCCTGGTGCCCCTGGCCCCGTT(G/A)GCCCTGCTGGCAAGAGTGGTGATCGTGGTG

[NM_000088.3](http://www.ncbi.nlm.nih.gov/entrez/viewer.fcgi?db=nucleotide&id=NM_000088.3): c.3182G>A; [NP_000079.2](http://www.ncbi.nlm.nih.gov/entrez/viewer.fcgi?db=nucleotide&id=NP_000079.2): p.G1061D

CTGGTGCCCCTGGCCCCGTTGGCCCTGCTG(G/A)CAAGAGTGGTGATCGTGGTGAGACTgtaag

[NM_000088.3](http://www.ncbi.nlm.nih.gov/entrez/viewer.fcgi?db=nucleotide&id=NM_000088.3): c.3181G>A; [NP_000079.2](http://www.ncbi.nlm.nih.gov/entrez/viewer.fcgi?db=nucleotide&id=NP_000079.2): p.G1061S

CCTGGTGCCCCTGGCCCCGTTGGCCCTGCT(G/A)GCAAGAGTGGTGATCGTGGTGAGACTgtaa

[NM_000088.3](http://www.ncbi.nlm.nih.gov/entrez/viewer.fcgi?db=nucleotide&id=NM_000088.3): c.3191G>C; [NP_000079.2](http://www.ncbi.nlm.nih.gov/entrez/viewer.fcgi?db=nucleotide&id=NP_000079.2): p.G1064A

CTGGCCCCGTTGGCCCTGCTGGCAAGAGTG(G/C)TGATCGTGGTGAGACTgtaagtagctgggc

[NM_000088.3](http://www.ncbi.nlm.nih.gov/entrez/viewer.fcgi?db=nucleotide&id=NM_000088.3): c.3196C>T; [NP_000079.2](http://www.ncbi.nlm.nih.gov/entrez/viewer.fcgi?db=nucleotide&id=NP_000079.2): p.R1066C

CCCGTTGGCCCTGCTGGCAAGAGTGGTGAT(C/T)GTGGTGAGACTgtaagtagctgggctccag

[NM_000088.3](http://www.ncbi.nlm.nih.gov/entrez/viewer.fcgi?db=nucleotide&id=NM_000088.3): c.3226G>T; [NP_000079.2](http://www.ncbi.nlm.nih.gov/entrez/viewer.fcgi?db=nucleotide&id=NP_000079.2): p.G1076C

tgctctccccagGGTCCTGCTGGTCCCACC(G/T)GTCCTGTCGGCCCTGTTGGCGCCCGTGGCC

[NM_000088.3](http://www.ncbi.nlm.nih.gov/entrez/viewer.fcgi?db=nucleotide&id=NM_000088.3): c.3226G>A; [NP_000079.2](http://www.ncbi.nlm.nih.gov/entrez/viewer.fcgi?db=nucleotide&id=NP_000079.2): p.G1076S

tgctctccccagGGTCCTGCTGGTCCCACC(G/A)GTCCTGTCGGCCCTGTTGGCGCCCGTGGCC

[NM_000088.3](http://www.ncbi.nlm.nih.gov/entrez/viewer.fcgi?db=nucleotide&id=NM_000088.3): c.3235G>A; [NP_000079.2](http://www.ncbi.nlm.nih.gov/entrez/viewer.fcgi?db=nucleotide&id=NP_000079.2): p.G1079S

cagGGTCCTGCTGGTCCCACCGGTCCTGTC(G/A)GCCCTGTTGGCGCCCGTGGCCCCGCCgtaa

[NM_000088.3](http://www.ncbi.nlm.nih.gov/entrez/viewer.fcgi?db=nucleotide&id=NM_000088.3): c.3244G>T; [NP_000079.2](http://www.ncbi.nlm.nih.gov/entrez/viewer.fcgi?db=nucleotide&id=NP_000079.2): p.G1082C

GCTGGTCCCACCGGTCCTGTCGGCCCTGTT(G/T)GCGCCCGTGGCCCCGCCgtaagtaccctgc

[NM_000088.3](http://www.ncbi.nlm.nih.gov/entrez/viewer.fcgi?db=nucleotide&id=NM_000088.3): c.3253G>A; [NP_000079.2](http://www.ncbi.nlm.nih.gov/entrez/viewer.fcgi?db=nucleotide&id=NP_000079.2): p.G1085S

ACCGGTCCTGTCGGCCCTGTTGGCGCCCGT(G/A)GCCCCGCCgtaagtaccctgctgtgtcccc

[NM_000088.3](http://www.ncbi.nlm.nih.gov/entrez/viewer.fcgi?db=nucleotide&id=NM_000088.3): c.3263G>C; [NP_000079.2](http://www.ncbi.nlm.nih.gov/entrez/viewer.fcgi?db=nucleotide&id=NP_000079.2): p.G1088A

ctgacagtttgtccctttctctcttctagG(G/C)ACCCCAAGGCCCCCGTGGTGACAAGGGTGA

[NM_000088.3](http://www.ncbi.nlm.nih.gov/entrez/viewer.fcgi?db=nucleotide&id=NM_000088.3): c.3271G>A; [NP_000079.2](http://www.ncbi.nlm.nih.gov/entrez/viewer.fcgi?db=nucleotide&id=NP_000079.2): p.G1091S

ttgtccctttctctcttctagGGACCCCAA(G/A)GCCCCCGTGGTGACAAGGGTGAGACAGGCG

[NM_000088.3](http://www.ncbi.nlm.nih.gov/entrez/viewer.fcgi?db=nucleotide&id=NM_000088.3): c.3277C>T; [NP_000079.2](http://www.ncbi.nlm.nih.gov/entrez/viewer.fcgi?db=nucleotide&id=NP_000079.2): p.R1093C

ctttctctcttctagGGACCCCAAGGCCCC(C/T)GTGGTGACAAGGGTGAGACAGGCGAACAGG

[NM_000088.3](http://www.ncbi.nlm.nih.gov/entrez/viewer.fcgi?db=nucleotide&id=NM_000088.3): c.3280G>A; [NP_000079.2](http://www.ncbi.nlm.nih.gov/entrez/viewer.fcgi?db=nucleotide&id=NP_000079.2): p.G1094S

tctctcttctagGGACCCCAAGGCCCCCGT(G/A)GTGACAAGGGTGAGACAGGCGAACAGGGCG

[NM_000088.3](http://www.ncbi.nlm.nih.gov/entrez/viewer.fcgi?db=nucleotide&id=NM_000088.3): c.3299G>A; [NP_000079.2](http://www.ncbi.nlm.nih.gov/entrez/viewer.fcgi?db=nucleotide&id=NP_000079.2): p.G1100D

AAGGCCCCCGTGGTGACAAGGGTGAGACAG(G/A)CGAACAGGGCGACAGAGGCATAAAGGGTCA

[NM_000088.3](http://www.ncbi.nlm.nih.gov/entrez/viewer.fcgi?db=nucleotide&id=NM_000088.3): c.3317G>C; [NP_000079.2](http://www.ncbi.nlm.nih.gov/entrez/viewer.fcgi?db=nucleotide&id=NP_000079.2): p.G1106A

AGGGTGAGACAGGCGAACAGGGCGACAGAG(G/C)CATAAAGGGTCACCGTGGCTTCTCTGGCCT

[NM_000088.3](http://www.ncbi.nlm.nih.gov/entrez/viewer.fcgi?db=nucleotide&id=NM_000088.3): c.3325G>A; [NP_000079.2](http://www.ncbi.nlm.nih.gov/entrez/viewer.fcgi?db=nucleotide&id=NP_000079.2): p.G1109S

ACAGGCGAACAGGGCGACAGAGGCATAAAG(G/A)GTCACCGTGGCTTCTCTGGCCTCCAGGGTC

[NM_000088.3](http://www.ncbi.nlm.nih.gov/entrez/viewer.fcgi?db=nucleotide&id=NM_000088.3): c.3349C>T; [NP_000079.2](http://www.ncbi.nlm.nih.gov/entrez/viewer.fcgi?db=nucleotide&id=NP_000079.2): p.Q1117X

ATAAAGGGTCACCGTGGCTTCTCTGGCCTC(C/T)AGGGTCCCCCTGGCCCTCCTgtaagtatgc

[NM_000088.3](http://www.ncbi.nlm.nih.gov/entrez/viewer.fcgi?db=nucleotide&id=NM_000088.3): c.3370G>T; [NP_000079.2](http://www.ncbi.nlm.nih.gov/entrez/viewer.fcgi?db=nucleotide&id=NP_000079.2): p.G1124C

cctgaactgccttttttatctgtccttcag(G/T)GCTCTCCTGGTGAACAAGGTCCCTCTGGAG

[NM_000088.3](http://www.ncbi.nlm.nih.gov/entrez/viewer.fcgi?db=nucleotide&id=NM_000088.3): c.3421C>T; [NP_000079.2](http://www.ncbi.nlm.nih.gov/entrez/viewer.fcgi?db=nucleotide&id=NP_000079.2): p.R1141X

CCCTCTGGAGCCTCTGGTCCTGCTGGTCCC(C/T)GAgtaagtcatgccttctctctcctcttcc

[NM_000088.3](http://www.ncbi.nlm.nih.gov/entrez/viewer.fcgi?db=nucleotide&id=NM_000088.3): c.3424G>A; [NP_000079.2](http://www.ncbi.nlm.nih.gov/entrez/viewer.fcgi?db=nucleotide&id=NP_000079.2): p.G1142S

gcctttctcaaaccatttcccccactccag(G/A)GTCCCCCTGGCTCTGCTGGTGCTCCTGGCA

[NM_000088.3](http://www.ncbi.nlm.nih.gov/entrez/viewer.fcgi?db=nucleotide&id=NM_000088.3): c.3434G>A; [NP_000079.2](http://www.ncbi.nlm.nih.gov/entrez/viewer.fcgi?db=nucleotide&id=NP_000079.2): p.G1145D

aaccatttcccccactccagGGTCCCCCTG(G/A)CTCTGCTGGTGCTCCTGGCAAAGATGGACT

[NM_000088.3](http://www.ncbi.nlm.nih.gov/entrez/viewer.fcgi?db=nucleotide&id=NM_000088.3): c.3433G>T; [NP_000079.2](http://www.ncbi.nlm.nih.gov/entrez/viewer.fcgi?db=nucleotide&id=NP_000079.2): p.G1145C

aaaccatttcccccactccagGGTCCCCCT(G/T)GCTCTGCTGGTGCTCCTGGCAAAGATGGAC

[NM_000088.3](http://www.ncbi.nlm.nih.gov/entrez/viewer.fcgi?db=nucleotide&id=NM_000088.3): c.3451G>A; [NP_000079.2](http://www.ncbi.nlm.nih.gov/entrez/viewer.fcgi?db=nucleotide&id=NP_000079.2): p.G1151S

cagGGTCCCCCTGGCTCTGCTGGTGCTCCT(G/A)GCAAAGATGGACTCAACGGTCTCCCTGGCC

[NM_000088.3](http://www.ncbi.nlm.nih.gov/entrez/viewer.fcgi?db=nucleotide&id=NM_000088.3): c.3452G>T; [NP_000079.2](http://www.ncbi.nlm.nih.gov/entrez/viewer.fcgi?db=nucleotide&id=NP_000079.2): p.G1151V

agGGTCCCCCTGGCTCTGCTGGTGCTCCTG(G/T)CAAAGATGGACTCAACGGTCTCCCTGGCCC

[NM_000088.3](http://www.ncbi.nlm.nih.gov/entrez/viewer.fcgi?db=nucleotide&id=NM_000088.3): c.3460G>A; [NP_000079.2](http://www.ncbi.nlm.nih.gov/entrez/viewer.fcgi?db=nucleotide&id=NP_000079.2): p.G1154R

CCTGGCTCTGCTGGTGCTCCTGGCAAAGAT(G/A)GACTCAACGGTCTCCCTGGCCCCATTGGGC

[NM_000088.3](http://www.ncbi.nlm.nih.gov/entrez/viewer.fcgi?db=nucleotide&id=NM_000088.3): c.3470G>A; [NP_000079.2](http://www.ncbi.nlm.nih.gov/entrez/viewer.fcgi?db=nucleotide&id=NP_000079.2): p.G1157D

CTGGTGCTCCTGGCAAAGATGGACTCAACG(G/A)TCTCCCTGGCCCCATTGGGCCCCCTGGTCC

[NM_000088.3](http://www.ncbi.nlm.nih.gov/entrez/viewer.fcgi?db=nucleotide&id=NM_000088.3): c.3496G>T; [NP_000079.2](http://www.ncbi.nlm.nih.gov/entrez/viewer.fcgi?db=nucleotide&id=NP_000079.2): p.G1166C

AACGGTCTCCCTGGCCCCATTGGGCCCCCT(G/T)GTCCTCGCGGTCGCACTGGTGATGCTGGTC

[NM_000088.3](http://www.ncbi.nlm.nih.gov/entrez/viewer.fcgi?db=nucleotide&id=NM_000088.3): c.3505G>T; [NP_000079.2](http://www.ncbi.nlm.nih.gov/entrez/viewer.fcgi?db=nucleotide&id=NP_000079.2): p.G1169C

CCTGGCCCCATTGGGCCCCCTGGTCCTCGC(G/T)GTCGCACTGGTGATGCTGGTCCTGTTgtat

[NM_000088.3](http://www.ncbi.nlm.nih.gov/entrez/viewer.fcgi?db=nucleotide&id=NM_000088.3): c.3505G>A; [NP_000079.2](http://www.ncbi.nlm.nih.gov/entrez/viewer.fcgi?db=nucleotide&id=NP_000079.2): p.G1169S

CCTGGCCCCATTGGGCCCCCTGGTCCTCGC(G/A)GTCGCACTGGTGATGCTGGTCCTGTTgtat

[NM_000088.3](http://www.ncbi.nlm.nih.gov/entrez/viewer.fcgi?db=nucleotide&id=NM_000088.3): c.3515G>A; [NP_000079.2](http://www.ncbi.nlm.nih.gov/entrez/viewer.fcgi?db=nucleotide&id=NP_000079.2): p.G1172D

TTGGGCCCCCTGGTCCTCGCGGTCGCACTG(G/A)TGATGCTGGTCCTGTTgtatgtagcccctc

[NM_000088.3](http://www.ncbi.nlm.nih.gov/entrez/viewer.fcgi?db=nucleotide&id=NM_000088.3): c.3521C>T; [NP_000079.2](http://www.ncbi.nlm.nih.gov/entrez/viewer.fcgi?db=nucleotide&id=NP_000079.2): p.A1174V

CCCCTGGTCCTCGCGGTCGCACTGGTGATG(C/T)TGGTCCTGTTgtatgtagcccctcatcccc

[NM_000088.3](http://www.ncbi.nlm.nih.gov/entrez/viewer.fcgi?db=nucleotide&id=NM_000088.3): c.3523G>T; [NP_000079.2](http://www.ncbi.nlm.nih.gov/entrez/viewer.fcgi?db=nucleotide&id=NP_000079.2): p.G1175C

CCTGGTCCTCGCGGTCGCACTGGTGATGCT(G/T)GTCCTGTTgtatgtagcccctcatcccctc

[NM_000088.3](http://www.ncbi.nlm.nih.gov/entrez/viewer.fcgi?db=nucleotide&id=NM_000088.3): c.3523G>A; [NP_000079.2](http://www.ncbi.nlm.nih.gov/entrez/viewer.fcgi?db=nucleotide&id=NP_000079.2): p.G1175S

CCTGGTCCTCGCGGTCGCACTGGTGATGCT(G/A)GTCCTGTTgtatgtagcccctcatcccctc

[NM_000088.3](http://www.ncbi.nlm.nih.gov/entrez/viewer.fcgi?db=nucleotide&id=NM_000088.3): c.3533G>T; [NP_000079.2](http://www.ncbi.nlm.nih.gov/entrez/viewer.fcgi?db=nucleotide&id=NP_000079.2): p.G1178V

aatccccactctcttccctctctgtgcagG(G/T)TCCCCCCGGCCCTCCTGGACCTCCTGGTCC

[NM_000088.3](http://www.ncbi.nlm.nih.gov/entrez/viewer.fcgi?db=nucleotide&id=NM_000088.3): c.3541G>A; [NP_000079.2](http://www.ncbi.nlm.nih.gov/entrez/viewer.fcgi?db=nucleotide&id=NP_000079.2): p.G1181S

ctctcttccctctctgtgcagGGTCCCCCC(G/A)GCCCTCCTGGACCTCCTGGTCCCCCTGGTC

[NM_000088.3](http://www.ncbi.nlm.nih.gov/entrez/viewer.fcgi?db=nucleotide&id=NM_000088.3): c.3551G>T; [NP_000079.2](http://www.ncbi.nlm.nih.gov/entrez/viewer.fcgi?db=nucleotide&id=NP_000079.2): p.G1184V

tctctgtgcagGGTCCCCCCGGCCCTCCTG(G/T)ACCTCCTGGTCCCCCTGGTCCTCCCAGCGC

[NM_000088.3](http://www.ncbi.nlm.nih.gov/entrez/viewer.fcgi?db=nucleotide&id=NM_000088.3): c.3559G>A; [NP_000079.2](http://www.ncbi.nlm.nih.gov/entrez/viewer.fcgi?db=nucleotide&id=NP_000079.2): p.G1187S

cagGGTCCCCCCGGCCCTCCTGGACCTCCT(G/A)GTCCCCCTGGTCCTCCCAGCGCTGGTTTCG

[NM_000088.3](http://www.ncbi.nlm.nih.gov/entrez/viewer.fcgi?db=nucleotide&id=NM_000088.3): c.3560G>T; [NP_000079.2](http://www.ncbi.nlm.nih.gov/entrez/viewer.fcgi?db=nucleotide&id=NP_000079.2): p.G1187V

agGGTCCCCCCGGCCCTCCTGGACCTCCTG(G/T)TCCCCCTGGTCCTCCCAGCGCTGGTTTCGA

[NM_000088.3](http://www.ncbi.nlm.nih.gov/entrez/viewer.fcgi?db=nucleotide&id=NM_000088.3): c.3583G>T; [NP_000079.2](http://www.ncbi.nlm.nih.gov/entrez/viewer.fcgi?db=nucleotide&id=NP_000079.2): p.G1195C

CCTCCTGGTCCCCCTGGTCCTCCCAGCGCT(G/T)GTTTCGACTTCAGCTTCCTGCCCCAGCCAC

[NM_000088.3](http://www.ncbi.nlm.nih.gov/entrez/viewer.fcgi?db=nucleotide&id=NM_000088.3): c.3595A>G; [NP_000079.2](http://www.ncbi.nlm.nih.gov/entrez/viewer.fcgi?db=nucleotide&id=NP_000079.2): p.S1199G

CCTGGTCCTCCCAGCGCTGGTTTCGACTTC(A/G)GCTTCCTGCCCCAGCCACCTCAAGAGAAGG

[NM_000088.3](http://www.ncbi.nlm.nih.gov/entrez/viewer.fcgi?db=nucleotide&id=NM_000088.3): c.3652G>A; [NP_000079.2](http://www.ncbi.nlm.nih.gov/entrez/viewer.fcgi?db=nucleotide&id=NP_000079.2): p.A1218T

AAGGCTCACGATGGTGGCCGCTACTACCGG(G/A)CTGATGATGCCAATGTGGTTCGTGACCGTG

[NM_000088.3](http://www.ncbi.nlm.nih.gov/entrez/viewer.fcgi?db=nucleotide&id=NM_000088.3): c.3655G>A; [NP_000079.2](http://www.ncbi.nlm.nih.gov/entrez/viewer.fcgi?db=nucleotide&id=NP_000079.2): p.D1219N

GCTCACGATGGTGGCCGCTACTACCGGGCT(G/A)ATGATGCCAATGTGGTTCGTGACCGTGACC

[NM_000088.3](http://www.ncbi.nlm.nih.gov/entrez/viewer.fcgi?db=nucleotide&id=NM_000088.3): c.3657T>G; [NP_000079.2](http://www.ncbi.nlm.nih.gov/entrez/viewer.fcgi?db=nucleotide&id=NP_000079.2): p.D1219E

TCACGATGGTGGCCGCTACTACCGGGCTGA(T/G)GATGCCAATGTGGTTCGTGACCGTGACCTC

[NM_000088.3](http://www.ncbi.nlm.nih.gov/entrez/viewer.fcgi?db=nucleotide&id=NM_000088.3): c.3766G>A; [NP_000079.2](http://www.ncbi.nlm.nih.gov/entrez/viewer.fcgi?db=nucleotide&id=NP_000079.2): p.A1256T

CGGAGCCCAGAGGGCAGCCGCAAGAACCCC(G/A)CCCGCACCTGCCGTGACCTCAAGATGTGCC

[NM_000088.3](http://www.ncbi.nlm.nih.gov/entrez/viewer.fcgi?db=nucleotide&id=NM_000088.3): c.3790A>G; [NP_000079.2](http://www.ncbi.nlm.nih.gov/entrez/viewer.fcgi?db=nucleotide&id=NP_000079.2): p.M1264V

AACCCCGCCCGCACCTGCCGTGACCTCAAG(A/G)TGTGCCACTCTGACTGGAAGAGTGgtgagg

[NM_000088.3](http://www.ncbi.nlm.nih.gov/entrez/viewer.fcgi?db=nucleotide&id=NM_000088.3): c.3806G>A; [NP_000079.2](http://www.ncbi.nlm.nih.gov/entrez/viewer.fcgi?db=nucleotide&id=NP_000079.2): p.W1269X

GCCGTGACCTCAAGATGTGCCACTCTGACT(G/A)GAAGAGTGgtgagggcctgccctagcctct

[NM_000088.3](http://www.ncbi.nlm.nih.gov/entrez/viewer.fcgi?db=nucleotide&id=NM_000088.3): c.3831C>G; [NP_000079.2](http://www.ncbi.nlm.nih.gov/entrez/viewer.fcgi?db=nucleotide&id=NP_000079.2): p.D1277E

ctgccctaccacagGAGAGTACTGGATTGA(C/G)CCCAACCAAGGCTGCAACCTGGATGCCATC

[NM_000088.3](http://www.ncbi.nlm.nih.gov/entrez/viewer.fcgi?db=nucleotide&id=NM_000088.3): c.3829G>C; [NP_000079.2](http://www.ncbi.nlm.nih.gov/entrez/viewer.fcgi?db=nucleotide&id=NP_000079.2): p.D1277H

ccctgccctaccacagGAGAGTACTGGATT(G/C)ACCCCAACCAAGGCTGCAACCTGGATGCCA

[NM_000088.3](http://www.ncbi.nlm.nih.gov/entrez/viewer.fcgi?db=nucleotide&id=NM_000088.3): c.3893C>T; [NP_000079.2](http://www.ncbi.nlm.nih.gov/entrez/viewer.fcgi?db=nucleotide&id=NP_000079.2): p.T1298I

AAGTCTTCTGCAACATGGAGACTGGTGAGA(C/T)CTGCGTGTACCCCACTCAGCCCAGTGTGGC

[NM_000088.3](http://www.ncbi.nlm.nih.gov/entrez/viewer.fcgi?db=nucleotide&id=NM_000088.3): c.3897C>G; [NP_000079.2](http://www.ncbi.nlm.nih.gov/entrez/viewer.fcgi?db=nucleotide&id=NP_000079.2): p.C1299W

CTTCTGCAACATGGAGACTGGTGAGACCTG(C/G)GTGTACCCCACTCAGCCCAGTGTGGCCCAG

[NM_000088.3](http://www.ncbi.nlm.nih.gov/entrez/viewer.fcgi?db=nucleotide&id=NM_000088.3): c.3910C>T; [NP_000079.2](http://www.ncbi.nlm.nih.gov/entrez/viewer.fcgi?db=nucleotide&id=NP_000079.2): p.Q1304X

GAGACTGGTGAGACCTGCGTGTACCCCACT(C/T)AGCCCAGTGTGGCCCAGAAGAACTGGTACA

[NM_000088.3](http://www.ncbi.nlm.nih.gov/entrez/viewer.fcgi?db=nucleotide&id=NM_000088.3): c.3925C>T; [NP_000079.2](http://www.ncbi.nlm.nih.gov/entrez/viewer.fcgi?db=nucleotide&id=NP_000079.2): p.Q1309X

TGCGTGTACCCCACTCAGCCCAGTGTGGCC(C/T)AGAAGAACTGGTACATCAGCAAGAACCCCA

[NM_000088.3](http://www.ncbi.nlm.nih.gov/entrez/viewer.fcgi?db=nucleotide&id=NM_000088.3): c.3936G>T; [NP_000079.2](http://www.ncbi.nlm.nih.gov/entrez/viewer.fcgi?db=nucleotide&id=NP_000079.2): p.W1312C

CACTCAGCCCAGTGTGGCCCAGAAGAACTG(G/T)TACATCAGCAAGAACCCCAAGGACAAGAGG

[NM_000088.3](http://www.ncbi.nlm.nih.gov/entrez/viewer.fcgi?db=nucleotide&id=NM_000088.3): c.3967C>T; [NP_000079.2](http://www.ncbi.nlm.nih.gov/entrez/viewer.fcgi?db=nucleotide&id=NP_000079.2): p.H1323Y

TACATCAGCAAGAACCCCAAGGACAAGAGG(C/T)ATGTCTGGTTCGGCGAGAGCATGACCGATG

[NM_000088.3](http://www.ncbi.nlm.nih.gov/entrez/viewer.fcgi?db=nucleotide&id=NM_000088.3): c.3974G>A; [NP_000079.2](http://www.ncbi.nlm.nih.gov/entrez/viewer.fcgi?db=nucleotide&id=NP_000079.2): p.W1325X

GCAAGAACCCCAAGGACAAGAGGCATGTCT(G/A)GTTCGGCGAGAGCATGACCGATGGATTCCA

[NM_000088.3](http://www.ncbi.nlm.nih.gov/entrez/viewer.fcgi?db=nucleotide&id=NM_000088.3): c.4160C>T; [NP_000079.2](http://www.ncbi.nlm.nih.gov/entrez/viewer.fcgi?db=nucleotide&id=NP_000079.2): p.A1387V

TGGACCAGCAGACTGGCAACCTCAAGAAGG(C/T)CCTGCTCCTCCAGGGCTCCAACGAGATCGA

[NM_000088.3](http://www.ncbi.nlm.nih.gov/entrez/viewer.fcgi?db=nucleotide&id=NM_000088.3): c.4163T>G; [NP_000079.2](http://www.ncbi.nlm.nih.gov/entrez/viewer.fcgi?db=nucleotide&id=NP_000079.2): p.L1388R

ACCAGCAGACTGGCAACCTCAAGAAGGCCC(T/G)GCTCCTCCAGGGCTCCAACGAGATCGAGAT

[NM_000088.3](http://www.ncbi.nlm.nih.gov/entrez/viewer.fcgi?db=nucleotide&id=NM_000088.3): c.4237G>A; [NP_000079.2](http://www.ncbi.nlm.nih.gov/entrez/viewer.fcgi?db=nucleotide&id=NP_000079.2): p.D1413N

AACAGCCGCTTCACCTACAGCGTCACTGTC(G/A)ATGGCTGCACGgtgagtgcccagaatcccc

[NM_000088.3](http://www.ncbi.nlm.nih.gov/entrez/viewer.fcgi?db=nucleotide&id=NM_000088.3): c.4295C>T; [NP_000079.2](http://www.ncbi.nlm.nih.gov/entrez/viewer.fcgi?db=nucleotide&id=NP_000079.2): p.T1432I

GGGGCAAGACAGTGATTGAATACAAAACCA(C/T)CAAGACCTCCCGCCTGCCCATCATCGATGT

[NM_000088.3](http://www.ncbi.nlm.nih.gov/entrez/viewer.fcgi?db=nucleotide&id=NM_000088.3): c.4321G>C; [NP_000079.2](http://www.ncbi.nlm.nih.gov/entrez/viewer.fcgi?db=nucleotide&id=NP_000079.2): p.D1441H

ACCACCAAGACCTCCCGCCTGCCCATCATC(G/C)ATGTGGCCCCCTTGGACGTTGGTGCCCCAG

[NM_000088.3](http://www.ncbi.nlm.nih.gov/entrez/viewer.fcgi?db=nucleotide&id=NM_000088.3): c.4321G>T; [NP_000079.2](http://www.ncbi.nlm.nih.gov/entrez/viewer.fcgi?db=nucleotide&id=NP_000079.2): p.D1441Y

ACCACCAAGACCTCCCGCCTGCCCATCATC(G/T)ATGTGGCCCCCTTGGACGTTGGTGCCCCAG

[NM_000088.3](http://www.ncbi.nlm.nih.gov/entrez/viewer.fcgi?db=nucleotide&id=NM_000088.3): c.4391T>C; [NP_000079.2](http://www.ncbi.nlm.nih.gov/entrez/viewer.fcgi?db=nucleotide&id=NP_000079.2): p.L1464P

TCGGCTTCGACGTTGGCCCTGTCTGCTTCC(T/C)GTAAactccctccatcccaacctggctccc

**Splicing**

[NM_000088.3](http://www.ncbi.nlm.nih.gov/entrez/viewer.fcgi?db=nucleotide&id=NM_000088.3): c.104-441G>T

gcccccgccaccccacctgcccagggaatg(g/t)gggcgggatgagggctggacctcccttctc

[NM_000088.3](http://www.ncbi.nlm.nih.gov/entrez/viewer.fcgi?db=nucleotide&id=NM_000088.3): c.104-3C>G

ctactgacaacgcccctcttccgcctgtcc(c/g)agTCCCACCAATCACCTGCGTACAGAACGG

[NM_000088.3](http://www.ncbi.nlm.nih.gov/entrez/viewer.fcgi?db=nucleotide&id=NM_000088.3): c.103+1G>A

AAGGCCAAGTCGAGGGCCAAGACGAAGACA(g/a)taagtcccaaacttttgggagtgcaaggat

[NM_000088.3](http://www.ncbi.nlm.nih.gov/entrez/viewer.fcgi?db=nucleotide&id=NM_000088.3): c.334-9A>G

ctaacctggcctcttgtttcttctccccca(a/g)tcccacagGGACCCAAGGGAGACACTGGCC

[NM_000088.3](http://www.ncbi.nlm.nih.gov/entrez/viewer.fcgi?db=nucleotide&id=NM_000088.3): c.334-2A>G

ggcctcttgtttcttctcccccaatcccac(a/g)gGGACCCAAGGGAGACACTGGCCCCCGAGG

[NM_000088.3](http://www.ncbi.nlm.nih.gov/entrez/viewer.fcgi?db=nucleotide&id=NM_000088.3): c.333+2T>C

CCACCGACCAAGAAACCACCGGCGTCGAGg(t/c)aatctcctgccctcgaattttgcccctgcg

[NM_000088.3](http://www.ncbi.nlm.nih.gov/entrez/viewer.fcgi?db=nucleotide&id=NM_000088.3): c.370-1G>A

ctcaccccggccccgccctctccccctgca(g/a)GGACCCGCAGGCCCCCCTGGCCGAGATGGC

[NM_000088.3](http://www.ncbi.nlm.nih.gov/entrez/viewer.fcgi?db=nucleotide&id=NM_000088.3): c.472-2A>G

gtcctcactctgacttctcttgtttgttct(a/g)gAACTTTGCTCCCCAGCTGTCTTATGGCTA

[NM_000088.3](http://www.ncbi.nlm.nih.gov/entrez/viewer.fcgi?db=nucleotide&id=NM_000088.3): c.472-1G>A

tcctcactctgacttctcttgtttgttcta(g/a)AACTTTGCTCCCCAGCTGTCTTATGGCTAT

[NM_000088.3](http://www.ncbi.nlm.nih.gov/entrez/viewer.fcgi?db=nucleotide&id=NM_000088.3): c.471+1G>A

GGACCTCCCGGACCCCCTGGCCTCGGAGGA(g/a)taagtggagaggccttgtgtgtccactctc

[NM_000088.3](http://www.ncbi.nlm.nih.gov/entrez/viewer.fcgi?db=nucleotide&id=NM_000088.3): c.543G>A

AACCGGAGGAATTTCCGTGCCTGGCCCCAT(G/A)gtgagccagcagggggagcatggatgacag

[NM_000088.3](http://www.ncbi.nlm.nih.gov/entrez/viewer.fcgi?db=nucleotide&id=NM_000088.3): c.543+2T>C

CCGGAGGAATTTCCGTGCCTGGCCCCATGg(t/c)gagccagcagggggagcatggatgacagaa

[NM_000088.3](http://www.ncbi.nlm.nih.gov/entrez/viewer.fcgi?db=nucleotide&id=NM_000088.3): c.589-2A>G

tcttctaacccatctttttccttcttctca(a/g)gGGTCCCCAAGGCTTCCAAGGTCCCCCTGG

[NM_000088.3](http://www.ncbi.nlm.nih.gov/entrez/viewer.fcgi?db=nucleotide&id=NM_000088.3): c.588+1G>A

CGTGGTCTCCCTGGCCCCCCTGGTGCACCT(g/a)tgagtatccaggacgtcttcatatgcctcc

[NM_000088.3](http://www.ncbi.nlm.nih.gov/entrez/viewer.fcgi?db=nucleotide&id=NM_000088.3): c.588+4A>T

GGTCTCCCTGGCCCCCCTGGTGCACCTgtg(a/t)gtatccaggacgtcttcatatgcctccttg

[NM_000088.3](http://www.ncbi.nlm.nih.gov/entrez/viewer.fcgi?db=nucleotide&id=NM_000088.3): c.643-2A>G

catattctaaccacctcttctatcttttct(a/g)gGGTCCCATGGGTCCCCGAGGTCCCCCAGG

[NM_000088.3](http://www.ncbi.nlm.nih.gov/entrez/viewer.fcgi?db=nucleotide&id=NM_000088.3): c.642+1G>A

CCTGGTGAGCCTGGCGAGCCTGGAGCTTCA(g/a)taagcactctctatacagattcatactcct

[NM_000088.3](http://www.ncbi.nlm.nih.gov/entrez/viewer.fcgi?db=nucleotide&id=NM_000088.3): c.642+5G>A

GTGAGCCTGGCGAGCCTGGAGCTTCAgtaa(g/a)cactctctatacagattcatactccttcta

[NM_000088.3](http://www.ncbi.nlm.nih.gov/entrez/viewer.fcgi?db=nucleotide&id=NM_000088.3): c.697-2A>G

agaggcatgatggtcttttctctccctctc(a/g)gGGGGAAGCTGGAAAACCTGGTCGTCCTGG

[NM_000088.3](http://www.ncbi.nlm.nih.gov/entrez/viewer.fcgi?db=nucleotide&id=NM_000088.3): c.697-2A>T

agaggcatgatggtcttttctctccctctc(a/t)gGGGGAAGCTGGAAAACCTGGTCGTCCTGG

[NM_000088.3](http://www.ncbi.nlm.nih.gov/entrez/viewer.fcgi?db=nucleotide&id=NM_000088.3): c.697-1G>T

gaggcatgatggtcttttctctccctctca(g/t)GGGGAAGCTGGAAAACCTGGTCGTCCTGGT

[NM_000088.3](http://www.ncbi.nlm.nih.gov/entrez/viewer.fcgi?db=nucleotide&id=NM_000088.3): c.696+2T>G

CAGGTCCCCCTGGAAAGAATGGAGATGATg(t/g)aagtatccccagcaagaagataccatctga

[NM_000088.3](http://www.ncbi.nlm.nih.gov/entrez/viewer.fcgi?db=nucleotide&id=NM_000088.3): c.804+5G>C

GCCTCCCTGGAATGAAGGGACACAGAgtga(g/c)tcacctttgagtcatttaagctccccaagt

[NM_000088.3](http://www.ncbi.nlm.nih.gov/entrez/viewer.fcgi?db=nucleotide&id=NM_000088.3): c.859-2A>C

gaatttctcactcattattctctgatctac(a/c)gGGTGAGCCTGGCAGCCCTGGTGAAAATGG

[NM_000088.3](http://www.ncbi.nlm.nih.gov/entrez/viewer.fcgi?db=nucleotide&id=NM_000088.3): c.859-1G>A

aatttctcactcattattctctgatctaca(g/a)GGTGAGCCTGGCAGCCCTGGTGAAAATGGA

[NM_000088.3](http://www.ncbi.nlm.nih.gov/entrez/viewer.fcgi?db=nucleotide&id=NM_000088.3): c.858+1G>A

AAGGGAGATGCTGGTCCTGCTGGTCCTAAG(g/a)taagaggctgtctgaacatcatggtcctcc

[NM_000088.3](http://www.ncbi.nlm.nih.gov/entrez/viewer.fcgi?db=nucleotide&id=NM_000088.3): c.858+1G>T

AAGGGAGATGCTGGTCCTGCTGGTCCTAAG(g/t)taagaggctgtctgaacatcatggtcctcc

[NM_000088.3](http://www.ncbi.nlm.nih.gov/entrez/viewer.fcgi?db=nucleotide&id=NM_000088.3): c.904-10T>A

gtctcccatctcatctgacttctcttggtt(t/a)ggttgtcagGGCCCCCGTGGCCTGCCTGGT

[NM_000088.3](http://www.ncbi.nlm.nih.gov/entrez/viewer.fcgi?db=nucleotide&id=NM_000088.3): c.958-1G>A

gacttaacggggcttcccctctctcctgca(g/a)GGTGCTCGTGGAAATGATGGTGCTACTGGT

[NM_000088.3](http://www.ncbi.nlm.nih.gov/entrez/viewer.fcgi?db=nucleotide&id=NM_000088.3): c.957+2T>A

[NM_000088.3](http://www.ncbi.nlm.nih.gov/entrez/viewer.fcgi?db=nucleotide&id=NM_000088.3): c.957+5G>A

GTCGCCCTGGAGCCCCTGGCCCTGCTgtaa(g/a)tactcctggccccttgggggatccctgagc

[NM_000088.3](http://www.ncbi.nlm.nih.gov/entrez/viewer.fcgi?db=nucleotide&id=NM_000088.3): c.1003-2A>G

ccttctcgctgacatctccatttcattcac(a/g)gGGTCCCACCGGCCCCGCTGGTCCTCCTGG

[NM_000088.3](http://www.ncbi.nlm.nih.gov/entrez/viewer.fcgi?db=nucleotide&id=NM_000088.3): c.1003-1G>C

cttctcgctgacatctccatttcattcaca(g/c)GGTCCCACCGGCCCCGCTGGTCCTCCTGGC

[NM_000088.3](http://www.ncbi.nlm.nih.gov/entrez/viewer.fcgi?db=nucleotide&id=NM_000088.3): c.1057-2A>C

tggcttctgatatgtcctttcttctgattc(a/c)gGGTGAAGCTGGTCCCCAAGGGCCCCGAGG

[NM_000088.3](http://www.ncbi.nlm.nih.gov/entrez/viewer.fcgi?db=nucleotide&id=NM_000088.3): c.1057-2A>G

tggcttctgatatgtcctttcttctgattc(a/g)gGGTGAAGCTGGTCCCCAAGGGCCCCGAGG

[NM_000088.3](http://www.ncbi.nlm.nih.gov/entrez/viewer.fcgi?db=nucleotide&id=NM_000088.3): c.1056+1G>A

CCTGGCTTCCCTGGTGCTGTTGGTGCTAAG(g/a)tgagaccccccactctcctctaagcatgac

[NM_000088.3](http://www.ncbi.nlm.nih.gov/entrez/viewer.fcgi?db=nucleotide&id=NM_000088.3): c.1155+1G>A

CCTGGCCCTGCTGGTGCTGCTGGCCCTGCT(g/a)taagtgtccccgactcagtgtcccctttgc

[NM_000088.3](http://www.ncbi.nlm.nih.gov/entrez/viewer.fcgi?db=nucleotide&id=NM_000088.3): c.1155+1G>C

CCTGGCCCTGCTGGTGCTGCTGGCCCTGCT(g/c)taagtgtccccgactcagtgtcccctttgc

[NM_000088.3](http://www.ncbi.nlm.nih.gov/entrez/viewer.fcgi?db=nucleotide&id=NM_000088.3): c.1201-1G>A

ggctcacgccctggctgtctgcctcccaca(g/a)GGTGCTCCTGGTATTGCTGGTGCTCCTGGC

[NM_000088.3](http://www.ncbi.nlm.nih.gov/entrez/viewer.fcgi?db=nucleotide&id=NM_000088.3): c.1200+1G>A

GATGGACAGCCTGGTGCTAAAGGTGCCAAT(g/a)taagtatcctgccaggcttcagtcccactc

[NM_000088.3](http://www.ncbi.nlm.nih.gov/entrez/viewer.fcgi?db=nucleotide&id=NM_000088.3): c.1299+1G>A

GGCGGCCCTCCTGGTCCCAAGGGTAACAGC(g/a)tgagtaccaaactctcccttctgcccaccc

[NM_000088.3](http://www.ncbi.nlm.nih.gov/entrez/viewer.fcgi?db=nucleotide&id=NM_000088.3): c.1299+1G>C

GGCGGCCCTCCTGGTCCCAAGGGTAACAGC(g/c)tgagtaccaaactctcccttctgcccaccc

[NM_000088.3](http://www.ncbi.nlm.nih.gov/entrez/viewer.fcgi?db=nucleotide&id=NM_000088.3): c.1354-12G>A

gcccctcaccacaggccgcctcctcctctc(g/a)gccctctccagGGCCCTGTTGGTGTTCAAG

[NM_000088.3](http://www.ncbi.nlm.nih.gov/entrez/viewer.fcgi?db=nucleotide&id=NM_000088.3): c.1354-2A>G

acaggccgcctcctcctctcggccctctcc(a/g)gGGCCCTGTTGGTGTTCAAGGACCCCCTGG

[NM_000088.3](http://www.ncbi.nlm.nih.gov/entrez/viewer.fcgi?db=nucleotide&id=NM_000088.3): c.1353+1G>C

AAAGGAGACACTGGTGCTAAGGGAGAGCCT(g/c)taagtctccccgccatccttcttgcagccc

[NM_000088.3](http://www.ncbi.nlm.nih.gov/entrez/viewer.fcgi?db=nucleotide&id=NM_000088.3): c.1353+2T>C

AAGGAGACACTGGTGCTAAGGGAGAGCCTg(t/c)aagtctccccgccatccttcttgcagccca

[NM_000088.3](http://www.ncbi.nlm.nih.gov/entrez/viewer.fcgi?db=nucleotide&id=NM_000088.3): c.1353+5G>C

GAGACACTGGTGCTAAGGGAGAGCCTgtaa(g/c)tctccccgccatccttcttgcagcccagcc

[NM_000088.3](http://www.ncbi.nlm.nih.gov/entrez/viewer.fcgi?db=nucleotide&id=NM_000088.3): c.1461+1G>C

ACTGGCCTGCCCGGACCCCCTGGCGAGCGT(g/c)taagtgtccctgcccgccccctcccgctcc

[NM_000088.3](http://www.ncbi.nlm.nih.gov/entrez/viewer.fcgi?db=nucleotide&id=NM_000088.3): c.1516-1G>T

cctaactgttcctgtgacttcccccaacca(g/t)GGTCCCGCTGGTGAACGTGGTTCTCCTGGC

[NM_000088.3](http://www.ncbi.nlm.nih.gov/entrez/viewer.fcgi?db=nucleotide&id=NM_000088.3): c.1515+3A>G

TGGCGCAGATGGTGTTGCTGGTCCCAAGgt(a/g)acctctccttgcggccggggggctgaccct

[NM_000088.3](http://www.ncbi.nlm.nih.gov/entrez/viewer.fcgi?db=nucleotide&id=NM_000088.3): c.1615-4C>A

cccaagtcccactcatacctctctgcctcc(c/a)tagGGTCTGACTGGAAGCCCTGGCAGCCCT

[NM_000088.3](http://www.ncbi.nlm.nih.gov/entrez/viewer.fcgi?db=nucleotide&id=NM_000088.3): c.1614+1G>A

CCCGGTGAAGCTGGTCTGCCTGGTGCCAAG(g/a)tgaggccccaggctttcagcctggcttggc

[NM_000088.3](http://www.ncbi.nlm.nih.gov/entrez/viewer.fcgi?db=nucleotide&id=NM_000088.3): c.1669-2A>G

ctcactgctgctttcgtgcctcccatcctt(a/g)gGGTCCCGCCGGTCAAGATGGTCGCCCCGG

[NM_000088.3](http://www.ncbi.nlm.nih.gov/entrez/viewer.fcgi?db=nucleotide&id=NM_000088.3): c.1768-1G>A

ccccctcaaccctaactccctttctccaca(g/a)GGAGAGCCCGGCAAGGCTGGAGAGCGAGGT

[NM_000088.3](http://www.ncbi.nlm.nih.gov/entrez/viewer.fcgi?db=nucleotide&id=NM_000088.3): c.1767+5G>C

GATTCCCTGGACCTAAAGGTGCTGCTgtga(g/c)tattaagtgaggatccatgaagagccaggg

[NM_000088.3](http://www.ncbi.nlm.nih.gov/entrez/viewer.fcgi?db=nucleotide&id=NM_000088.3): c.1822-2A>C

accctgagttcccctttgctctctccctgc(a/c)gGGTCCTGCTGGCAAAGATGGAGAGGCTGG

[NM_000088.3](http://www.ncbi.nlm.nih.gov/entrez/viewer.fcgi?db=nucleotide&id=NM_000088.3): c.1821+1G>A

CGAGGTGTTCCCGGACCCCCTGGCGCTGTC(g/a)taagtatctcctttccatccctacctcctt

[NM_000088.3](http://www.ncbi.nlm.nih.gov/entrez/viewer.fcgi?db=nucleotide&id=NM_000088.3): c.1821+1G>C

CGAGGTGTTCCCGGACCCCCTGGCGCTGTC(g/c)taagtatctcctttccatccctacctcctt

[NM_000088.3](http://www.ncbi.nlm.nih.gov/entrez/viewer.fcgi?db=nucleotide&id=NM_000088.3): c.1821+1G>T

CGAGGTGTTCCCGGACCCCCTGGCGCTGTC(g/t)taagtatctcctttccatccctacctcctt

[NM_000088.3](http://www.ncbi.nlm.nih.gov/entrez/viewer.fcgi?db=nucleotide&id=NM_000088.3): c.1876-2A>G

ctccccgtgactgtagtgttctctctgtgc(a/g)gGGTCCCGCTGGCGAGAGAGGTGAACAAGG

[NM_000088.3](http://www.ncbi.nlm.nih.gov/entrez/viewer.fcgi?db=nucleotide&id=NM_000088.3): c.1876-1G>C

tccccgtgactgtagtgttctctctgtgca(g/c)GGTCCCGCTGGCGAGAGAGGTGAACAAGGC

[NM_000088.3](http://www.ncbi.nlm.nih.gov/entrez/viewer.fcgi?db=nucleotide&id=NM_000088.3): c.1875+1G>A

GCTGGAGCTCAGGGACCCCCTGGCCCTGCT(g/a)tgagtgtccctgatggggagatctggggag

[NM_000088.3](http://www.ncbi.nlm.nih.gov/entrez/viewer.fcgi?db=nucleotide&id=NM_000088.3): c.1875+1G>C

GCTGGAGCTCAGGGACCCCCTGGCCCTGCT(g/c)tgagtgtccctgatggggagatctggggag

[NM_000088.3](http://www.ncbi.nlm.nih.gov/entrez/viewer.fcgi?db=nucleotide&id=NM_000088.3): c.1984-5C>A

tgctcactgtctgttcctctctccctcccc(c/a)tcagGGTGTTCCTGGAGACCTTGGCGCCCC

[NM_000088.3](http://www.ncbi.nlm.nih.gov/entrez/viewer.fcgi?db=nucleotide&id=NM_000088.3): c.1984-2A>C

tcactgtctgttcctctctccctccccctc(a/c)gGGTGTTCCTGGAGACCTTGGCGCCCCTGG

[NM_000088.3](http://www.ncbi.nlm.nih.gov/entrez/viewer.fcgi?db=nucleotide&id=NM_000088.3): c.2028+1G>A

CTTGGCGCCCCTGGCCCCTCTGGAGCAAGA(g/a)taagtaggcctctctcgctgcatccgtcaa

[NM_000088.3](http://www.ncbi.nlm.nih.gov/entrez/viewer.fcgi?db=nucleotide&id=NM_000088.3): c.2028+2T>G

TTGGCGCCCCTGGCCCCTCTGGAGCAAGAg(t/g)aagtaggcctctctcgctgcatccgtcaag

[NM_000088.3](http://www.ncbi.nlm.nih.gov/entrez/viewer.fcgi?db=nucleotide&id=NM_000088.3): c.2028+4A>G

GGCGCCCCTGGCCCCTCTGGAGCAAGAgta(a/g)gtaggcctctctcgctgcatccgtcaaggt

[NM_000088.3](http://www.ncbi.nlm.nih.gov/entrez/viewer.fcgi?db=nucleotide&id=NM_000088.3): c.2128-3T>G

ggcctcactccagtcttcttggttgtcaca(t/g)agGGTGATGCTGGTGCCCCTGGAGCTCCCG

[NM_000088.3](http://www.ncbi.nlm.nih.gov/entrez/viewer.fcgi?db=nucleotide&id=NM_000088.3): c.2128-1G>A

cctcactccagtcttcttggttgtcacata(g/a)GGTGATGCTGGTGCCCCTGGAGCTCCCGGT

[NM_000088.3](http://www.ncbi.nlm.nih.gov/entrez/viewer.fcgi?db=nucleotide&id=NM_000088.3): c.2128-1G>C

cctcactccagtcttcttggttgtcacata(g/c)GGTGATGCTGGTGCCCCTGGAGCTCCCGGT

[NM_000088.3](http://www.ncbi.nlm.nih.gov/entrez/viewer.fcgi?db=nucleotide&id=NM_000088.3): c.2127+2T>G

ACGGTGCTCCCGGCAACGATGGTGCTAAGg(t/g)gagggcagcgtggaaggggctctggcaagt

[NM_000088.3](http://www.ncbi.nlm.nih.gov/entrez/viewer.fcgi?db=nucleotide&id=NM_000088.3): c.2452-2A>G

ccctccattcaccgccctgcttcctccccc(a/g)gGGTGCTGACGGCCAACCTGGTGCTAAAGG

[NM_000088.3](http://www.ncbi.nlm.nih.gov/entrez/viewer.fcgi?db=nucleotide&id=NM_000088.3): c.2452-1G>A

cctccattcaccgccctgcttcctccccca(g/a)GGTGCTGACGGCCAACCTGGTGCTAAAGGC

[NM_000088.3](http://www.ncbi.nlm.nih.gov/entrez/viewer.fcgi?db=nucleotide&id=NM_000088.3): c.2451+94G>T

atgaggacaaaggcgtcagccatcctcagg(g/t)gagaagggtggagacgggattgtttcccac

[NM_000088.3](http://www.ncbi.nlm.nih.gov/entrez/viewer.fcgi?db=nucleotide&id=NM_000088.3): c.2560-1G>A

ccaggcttgatgcctctgtgctctcctaca(g/a)GGTAATGTTGGTGCTCCTGGAGCCAAAGGT

[NM_000088.3](http://www.ncbi.nlm.nih.gov/entrez/viewer.fcgi?db=nucleotide&id=NM_000088.3): c.2559+1G>A

GCCGGACCCGCTGGACCCCCTGGCCCCATT(g/a)tgagtggcttggccctctgtgcccacgagg

[NM_000088.3](http://www.ncbi.nlm.nih.gov/entrez/viewer.fcgi?db=nucleotide&id=NM_000088.3): c.2559+5G>A

GACCCGCTGGACCCCCTGGCCCCATTgtga(g/a)tggcttggccctctgtgcccacgaggctgg

[NM_000088.3](http://www.ncbi.nlm.nih.gov/entrez/viewer.fcgi?db=nucleotide&id=NM_000088.3): c.2667+1G>A

GCTGGCCGAGTCGGTCCTCCTGGCCCCTCT(g/a)taagtctctgcagcagagtccactgctcta

[NM_000088.3](http://www.ncbi.nlm.nih.gov/entrez/viewer.fcgi?db=nucleotide&id=NM_000088.3): c.2829+1G>A

AAAGGATCCCCTGGTGCTGATGGTCCTGCT(g/a)taagtgccagctcagatctctgcagctccg

[NM_000088.3](http://www.ncbi.nlm.nih.gov/entrez/viewer.fcgi?db=nucleotide&id=NM_000088.3): c.2829+3A>C

AGGATCCCCTGGTGCTGATGGTCCTGCTgt(a/c)agtgccagctcagatctctgcagctccgga

[NM_000088.3](http://www.ncbi.nlm.nih.gov/entrez/viewer.fcgi?db=nucleotide&id=NM_000088.3): c.2937+4A>C

GGCTTCCCTGGTCTTCCTGGCCCCTCTgta(a/c)gtgcccccctcaccttggggggccctgaga

[NM_000088.3](http://www.ncbi.nlm.nih.gov/entrez/viewer.fcgi?db=nucleotide&id=NM_000088.3): c.3046-2A>G

tgactctcacttctctctctctctctctgc(a/g)gGGGGCTCCTGGTGCCGAAGGTTCCCCTGG

[NM_000088.3](http://www.ncbi.nlm.nih.gov/entrez/viewer.fcgi?db=nucleotide&id=NM_000088.3): c.3046-1G>A

gactctcacttctctctctctctctctgca(g/a)GGGGCTCCTGGTGCCGAAGGTTCCCCTGGA

[NM_000088.3](http://www.ncbi.nlm.nih.gov/entrez/viewer.fcgi?db=nucleotide&id=NM_000088.3): c.3046-1G>T

gactctcacttctctctctctctctctgca(g/t)GGGGCTCCTGGTGCCGAAGGTTCCCCTGGA

[NM_000088.3](http://www.ncbi.nlm.nih.gov/entrez/viewer.fcgi?db=nucleotide&id=NM_000088.3): c.3100-2A>G

gctgacccatattcccctgctctccccgcc(a/g)gGGTGACCGTGGTGAGACCGGCCCCGCTGG

[NM_000088.3](http://www.ncbi.nlm.nih.gov/entrez/viewer.fcgi?db=nucleotide&id=NM_000088.3): c.3100-1G>A

ctgacccatattcccctgctctccccgcca(g/a)GGTGACCGTGGTGAGACCGGCCCCGCTGGA

[NM_000088.3](http://www.ncbi.nlm.nih.gov/entrez/viewer.fcgi?db=nucleotide&id=NM_000088.3): c.3208-2A>C

ccaagagctcatgctttccttgctctcccc(a/c)gGGTCCTGCTGGTCCCACCGGTCCTGTCGG

[NM_000088.3](http://www.ncbi.nlm.nih.gov/entrez/viewer.fcgi?db=nucleotide&id=NM_000088.3): c.3207+1G>A

GCTGGCAAGAGTGGTGATCGTGGTGAGACT(g/a)taagtagctgggctccagttccctgtacct

[NM_000088.3](http://www.ncbi.nlm.nih.gov/entrez/viewer.fcgi?db=nucleotide&id=NM_000088.3): c.3207+5G>A

GCAAGAGTGGTGATCGTGGTGAGACTgtaa(g/a)tagctgggctccagttccctgtacctggtc

[NM_000088.3](http://www.ncbi.nlm.nih.gov/entrez/viewer.fcgi?db=nucleotide&id=NM_000088.3): c.3207+5G>T

GCAAGAGTGGTGATCGTGGTGAGACTgtaa(g/t)tagctgggctccagttccctgtacctggtc

[NM_000088.3](http://www.ncbi.nlm.nih.gov/entrez/viewer.fcgi?db=nucleotide&id=NM_000088.3): c.3261+1G>A

GTCGGCCCTGTTGGCGCCCGTGGCCCCGCC(g/a)taagtaccctgctgtgtcccccatgccttt

[NM_000088.3](http://www.ncbi.nlm.nih.gov/entrez/viewer.fcgi?db=nucleotide&id=NM_000088.3): c.3424-3C>G

gcagcctttctcaaaccatttcccccactc(c/g)agGGTCCCCCTGGCTCTGCTGGTGCTCCTG

[NM_000088.3](http://www.ncbi.nlm.nih.gov/entrez/viewer.fcgi?db=nucleotide&id=NM_000088.3): c.3424-1G>C

agcctttctcaaaccatttcccccactcca(g/c)GGTCCCCCTGGCTCTGCTGGTGCTCCTGGC

[NM_000088.3](http://www.ncbi.nlm.nih.gov/entrez/viewer.fcgi?db=nucleotide&id=NM_000088.3): c.3423+1G>A

TCTGGAGCCTCTGGTCCTGCTGGTCCCCGA(g/a)taagtcatgccttctctctcctcttcctga

[NM_000088.3](http://www.ncbi.nlm.nih.gov/entrez/viewer.fcgi?db=nucleotide&id=NM_000088.3): c.3423+2T>A

CTGGAGCCTCTGGTCCTGCTGGTCCCCGAg(t/a)aagtcatgccttctctctcctcttcctgag

[NM_000088.3](http://www.ncbi.nlm.nih.gov/entrez/viewer.fcgi?db=nucleotide&id=NM_000088.3): c.3532-2A>G

ggtaatccccactctcttccctctctgtgc(a/g)gGGTCCCCCCGGCCCTCCTGGACCTCCTGG

[NM_000088.3](http://www.ncbi.nlm.nih.gov/entrez/viewer.fcgi?db=nucleotide&id=NM_000088.3): c.3531+1G>A

CGCGGTCGCACTGGTGATGCTGGTCCTGTT(g/a)tatgtagcccctcatcccctctgctcatgg

[NM_000088.3](http://www.ncbi.nlm.nih.gov/entrez/viewer.fcgi?db=nucleotide&id=NM_000088.3): c.3531+5G>A

GTCGCACTGGTGATGCTGGTCCTGTTgtat(g/a)tagcccctcatcccctctgctcatggccct

[NM_000088.3](http://www.ncbi.nlm.nih.gov/entrez/viewer.fcgi?db=nucleotide&id=NM_000088.3): c.3790A>G

AACCCCGCCCGCACCTGCCGTGACCTCAAG(A/G)TGTGCCACTCTGACTGGAAGAGTGgtgagg

**Regulatory**

ACAGGACCCCTGTCGCCTATTAGGGAGGGG(G-T)TCTCTTGGAACTGACCCACAGTAGGGGGCA
-1990 relative to transcription initiation site

CCTCTCCGAGGGGCAGGGTTCCTCCCTGCT(C-T)TCCATCAGGACAGTATAAAAGGGGCCCGGG
-161 relative to initiation codon

GCCCCCGCCACCCCACCTGCCCAGGGAATG(G-T)GGGCGGGATGAGGGCTGGACCTCCCTTCTC
+1252 relative to transcription initiation site

**Small deletions**

[NM_000088.3](http://www.ncbi.nlm.nih.gov/entrez/viewer.fcgi?db=nucleotide&id=NM_000088.3): c.246delC

CGTGATCTGTGACGAGACCAAGAACTGCCC(C/-)GGCGCCGAAGTCCCCGAGGGCGAGTGCTGT

.[NM_000088.3](http://www.ncbi.nlm.nih.gov/entrez/viewer.fcgi?db=nucleotide&id=NM_000088.3): c.314_324delAAGAAACCACC

ctcttcgtcccctagAGTCACCCACCGACC(AAGAAACCACC/-)GGCGTCGAGgtaatctcctgccctcgaatt

[NM_000088.3](http://www.ncbi.nlm.nih.gov/entrez/viewer.fcgi?db=nucleotide&id=NM_000088.3): c.386delC

ctctccccctgcagGGACCCGCAGGCCCCC(C/-)TGGCCGAGATGGCATCCCTGGACAGCCTGG

[NM_000088.3](http://www.ncbi.nlm.nih.gov/entrez/viewer.fcgi?db=nucleotide&id=NM_000088.3): c.423delC

AGATGGCATCCCTGGACAGCCTGGACTTCC(C/-)GGACCCCCCGGACCCCCCGGACCTCCCGGA

[NM_000088.3](http://www.ncbi.nlm.nih.gov/entrez/viewer.fcgi?db=nucleotide&id=NM_000088.3): c.432delC

CCCTGGACAGCCTGGACTTCCCGGACCCCC(C/-)GGACCCCCCGGACCTCCCGGACCCCCTGGC

[NM_000088.3](http://www.ncbi.nlm.nih.gov/entrez/viewer.fcgi?db=nucleotide&id=NM_000088.3): c.441delC

GCCTGGACTTCCCGGACCCCCCGGACCCCC(C/-)GGACCTCCCGGACCCCCTGGCCTCGGAGGA

[NM_000088.3](http://www.ncbi.nlm.nih.gov/entrez/viewer.fcgi?db=nucleotide&id=NM_000088.3): c.459delT

CCCCGGACCCCCCGGACCTCCCGGACCCCC(T/-)GGCCTCGGAGGAgtaagtggagaggccttg

[NM_000088.3](http://www.ncbi.nlm.nih.gov/entrez/viewer.fcgi?db=nucleotide&id=NM_000088.3): c.484delC

ttctcttgtttgttctagAACTTTGCTCCC(C/-)AGCTGTCTTATGGCTATGATGAGAAATCAA

[NM_000088.3](http://www.ncbi.nlm.nih.gov/entrez/viewer.fcgi?db=nucleotide&id=NM_000088.3): c.533delC

ATGAGAAATCAACCGGAGGAATTTCCGTGC(C/-)TGGCCCCATGgtgagccagcagggggagca

[NM_000088.3](http://www.ncbi.nlm.nih.gov/entrez/viewer.fcgi?db=nucleotide&id=NM_000088.3): c.579delT

CTCTGGTCCTCGTGGTCTCCCTGGCCCCCC(T/-)GGTGCACCTgtgagtatccaggacgtcttc

[NM_000088.3](http://www.ncbi.nlm.nih.gov/entrez/viewer.fcgi?db=nucleotide&id=NM_000088.3): c.614delC

tcaagGGTCCCCAAGGCTTCCAAGGTCCCC(C/-)TGGTGAGCCTGGCGAGCCTGGAGCTTCAgt

[NM_000088.3](http://www.ncbi.nlm.nih.gov/entrez/viewer.fcgi?db=nucleotide&id=NM_000088.3): c.669delA

ctagGGTCCCATGGGTCCCCGAGGTCCCCC(A/-)GGTCCCCCTGGAAAGAATGGAGATGATgta

[NM_000088.3](http://www.ncbi.nlm.nih.gov/entrez/viewer.fcgi?db=nucleotide&id=NM_000088.3): c.671delG

agGGTCCCATGGGTCCCCGAGGTCCCCCAG(G/-)TCCCCCTGGAAAGAATGGAGATGATgtaag

[NM_000088.3](http://www.ncbi.nlm.nih.gov/entrez/viewer.fcgi?db=nucleotide&id=NM_000088.3): c.697-2delA

agaggcatgatggtcttttctctccctctc(a/-)gGGGGAAGCTGGAAAACCTGGTCGTCCTGG

[NM_000088.3](http://www.ncbi.nlm.nih.gov/entrez/viewer.fcgi?db=nucleotide&id=NM_000088.3): c.697-2_697-1delAG

agaggcatgatggtcttttctctccctctc(ag/-)GGGGAAGCTGGAAAACCTGGTCGTCCTGGT

[NM_000088.3](http://www.ncbi.nlm.nih.gov/entrez/viewer.fcgi?db=nucleotide&id=NM_000088.3): c.775delG

ctccagGGTGCTCGAGGATTGCCCGGAACA(G/-)CTGGCCTCCCTGGAATGAAGGGACACAGAg

[NM_000088.3](http://www.ncbi.nlm.nih.gov/entrez/viewer.fcgi?db=nucleotide&id=NM_000088.3): c.804+3_804+6delGAGT

TGGCCTCCCTGGAATGAAGGGACACAGAgt(gagt/-)cacctttgagtcatttaagctccccaagtc

[NM_000088.3](http://www.ncbi.nlm.nih.gov/entrez/viewer.fcgi?db=nucleotide&id=NM_000088.3): c.804+2_804+3delTG

CTGGCCTCCCTGGAATGAAGGGACACAGAg(tg/-)agtcacctttgagtcatttaagctccccaa

[NM_000088.3](http://www.ncbi.nlm.nih.gov/entrez/viewer.fcgi?db=nucleotide&id=NM_000088.3): c.809delT

taccttctacctctttcctgctttagGGTT(T/-)CAGTGGTTTGGATGGTGCCAAGGGAGATGC

[NM_000088.3](http://www.ncbi.nlm.nih.gov/entrez/viewer.fcgi?db=nucleotide&id=NM_000088.3): c.858+1_858+5delGTAAG

AAGGGAGATGCTGGTCCTGCTGGTCCTAAG(gtaag/-)aggctgtctgaacatcatggtcctccacat

[NM_000088.3](http://www.ncbi.nlm.nih.gov/entrez/viewer.fcgi?db=nucleotide&id=NM_000088.3): c.879_880delTG

tgatctacagGGTGAGCCTGGCAGCCCTGG(TG/-)AAAATGGAGCTCCTGGTCAGATGgtgagtg

[NM_000088.3](http://www.ncbi.nlm.nih.gov/entrez/viewer.fcgi?db=nucleotide&id=NM_000088.3): c.1065delT

tatgtcctttcttctgattcagGGTGAAGC(T/-)GGTCCCCAAGGGCCCCGAGGCTCTGAAGGT

[NM_000088.3](http://www.ncbi.nlm.nih.gov/entrez/viewer.fcgi?db=nucleotide&id=NM_000088.3): c.1127delC

CCCAGGGTGTGCGTGGTGAGCCTGGCCCCC(C/-)TGGCCCTGCTGGTGCTGCTGGCCCTGCTgt

[NM_000088.3](http://www.ncbi.nlm.nih.gov/entrez/viewer.fcgi?db=nucleotide&id=NM_000088.3): c.1128delT

CCAGGGTGTGCGTGGTGAGCCTGGCCCCCC(T/-)GGCCCTGCTGGTGCTGCTGGCCCTGCTgta

[NM_000088.3](http://www.ncbi.nlm.nih.gov/entrez/viewer.fcgi?db=nucleotide&id=NM_000088.3): c.1155+3_1155+6delAAGT

TGGCCCTGCTGGTGCTGCTGGCCCTGCTgt(aagt/-)gtccccgactcagtgtcccctttgccactt

[NM_000088.3](http://www.ncbi.nlm.nih.gov/entrez/viewer.fcgi?db=nucleotide&id=NM_000088.3): c.1452delT

ACCCGGACCCACTGGCCTGCCCGGACCCCC(T/-)GGCGAGCGTgtaagtgtccctgcccgcccc

[NM_000088.3](http://www.ncbi.nlm.nih.gov/entrez/viewer.fcgi?db=nucleotide&id=NM_000088.3): c.1486_1487delCC

ctgcagGGTGGACCTGGTAGCCGTGGTTTC(CC/-)TGGCGCAGATGGTGTTGCTGGTCCCAAGgt

[NM_000088.3](http://www.ncbi.nlm.nih.gov/entrez/viewer.fcgi?db=nucleotide&id=NM_000088.3): c.1668delT

CCCTGGTCCTGATGGCAAAACTGGCCCCCC(T/-)gtaagtatcactccccctgaaccccctgcc

[NM_000088.3](http://www.ncbi.nlm.nih.gov/entrez/viewer.fcgi?db=nucleotide&id=NM_000088.3): c.1812delT

GGCTGGAGAGCGAGGTGTTCCCGGACCCCC(T/-)GGCGCTGTCgtaagtatctcctttccatcc

[NM_000088.3](http://www.ncbi.nlm.nih.gov/entrez/viewer.fcgi?db=nucleotide&id=NM_000088.3): c.1814delG

CTGGAGAGCGAGGTGTTCCCGGACCCCCTG(G/-)CGCTGTCgtaagtatctcctttccatccct

[NM_000088.3](http://www.ncbi.nlm.nih.gov/entrez/viewer.fcgi?db=nucleotide&id=NM_000088.3): c.1845_1847delGGC

cctgcagGGTCCTGCTGGCAAAGATGGAGA(GGC/-)TGGAGCTCAGGGACCCCCTGGCCCTGCTgt

[NM_000088.3](http://www.ncbi.nlm.nih.gov/entrez/viewer.fcgi?db=nucleotide&id=NM_000088.3): c.1865delC

AAGATGGAGAGGCTGGAGCTCAGGGACCCC(C/-)TGGCCCTGCTgtgagtgtccctgatgggga

[NM_000088.3](http://www.ncbi.nlm.nih.gov/entrez/viewer.fcgi?db=nucleotide&id=NM_000088.3): c.1866delT

AGATGGAGAGGCTGGAGCTCAGGGACCCCC(T/-)GGCCCTGCTgtgagtgtccctgatggggag

[NM_000088.3](http://www.ncbi.nlm.nih.gov/entrez/viewer.fcgi?db=nucleotide&id=NM_000088.3): c.1876-2delA

ctccccgtgactgtagtgttctctctgtgc(a/-)gGGTCCCGCTGGCGAGAGAGGTGAACAAGG

[NM_000088.3](http://www.ncbi.nlm.nih.gov/entrez/viewer.fcgi?db=nucleotide&id=NM_000088.3): c.1886delG

gtagtgttctctctgtgcagGGTCCCGCTG(G/-)CGAGAGAGGTGAACAAGGCCCTGCTGGCTC

[NM_000088.3](http://www.ncbi.nlm.nih.gov/entrez/viewer.fcgi?db=nucleotide&id=NM_000088.3): c.1893_1894delAG

tctctctgtgcagGGTCCCGCTGGCGAGAG(AG/-)GTGAACAAGGCCCTGCTGGCTCCCCCGGAT

[NM_000088.3](http://www.ncbi.nlm.nih.gov/entrez/viewer.fcgi?db=nucleotide&id=NM_000088.3): c.1944delT

catttcccacctacagGGTCTCCCTGGTCC(T/-)GCTGGTCCTCCAGGTGAAGCAGGCAAACCT

[NM_000088.3](http://www.ncbi.nlm.nih.gov/entrez/viewer.fcgi?db=nucleotide&id=NM_000088.3): c.1984-5delC

tgctcactgtctgttcctctctccctcccc(c/-)tcagGGTGTTCCTGGAGACCTTGGCGCCCC

[NM_000088.3](http://www.ncbi.nlm.nih.gov/entrez/viewer.fcgi?db=nucleotide&id=NM_000088.3): c.1994delG

cctctctccctccccctcagGGTGTTCCTG(G/-)AGACCTTGGCGCCCCTGGCCCCTCTGGAGC

[NM_000088.3](http://www.ncbi.nlm.nih.gov/entrez/viewer.fcgi?db=nucleotide&id=NM_000088.3): c.2010delT

tcagGGTGTTCCTGGAGACCTTGGCGCCCC(T/-)GGCCCCTCTGGAGCAAGAgtaagtaggcct

[NM_000088.3](http://www.ncbi.nlm.nih.gov/entrez/viewer.fcgi?db=nucleotide&id=NM_000088.3): c.2072delC

TCCCTGGCGAGCGTGGTGTGCAAGGTCCCC(C/-)TGGTCCTGCTGGTCCCCGAGGGGCCAACGG

[NM_000088.3](http://www.ncbi.nlm.nih.gov/entrez/viewer.fcgi?db=nucleotide&id=NM_000088.3): c.2073delT

CCCTGGCGAGCGTGGTGTGCAAGGTCCCCC(T/-)GGTCCTGCTGGTCCCCGAGGGGCCAACGGT

[NM_000088.3](http://www.ncbi.nlm.nih.gov/entrez/viewer.fcgi?db=nucleotide&id=NM_000088.3): c.2097delC

TCCCCCTGGTCCTGCTGGTCCCCGAGGGGC(C/-)AACGGTGCTCCCGGCAACGATGGTGCTAAG

[NM_000088.3](http://www.ncbi.nlm.nih.gov/entrez/viewer.fcgi?db=nucleotide&id=NM_000088.3): c.2235_2235+1delAG

AGCTGGTCTTCCAGGGCCTAAGGGTGACAG(Ag/-)taagttcaaccttccccctcccctgagccc

[NM_000088.3](http://www.ncbi.nlm.nih.gov/entrez/viewer.fcgi?db=nucleotide&id=NM_000088.3): c.2268_2269delTC

TGATGCTGGTCCCAAAGGTGCTGATGGCTC(TC/-)CTGGCAAAGATGGCGTCCGTGGTCTGACTG

[NM_000088.3](http://www.ncbi.nlm.nih.gov/entrez/viewer.fcgi?db=nucleotide&id=NM_000088.3): c.2366delC

gctctcagGGTGAAAGTGGTCCCAGCGGCC(C/-)TGCTGGTCCCACTGGAGCTCGTGGTGCCCC

[NM_000088.3](http://www.ncbi.nlm.nih.gov/entrez/viewer.fcgi?db=nucleotide&id=NM_000088.3): c.2424delC

ctagGGAGACCGTGGTGAGCCTGGTCCCCC(C/-)GGCCCTGCTGGCTTTGCTGGCCCCCCTgtg

[NM_000088.3](http://www.ncbi.nlm.nih.gov/entrez/viewer.fcgi?db=nucleotide&id=NM_000088.3): c.2444delG

CTGGTCCCCCCGGCCCTGCTGGCTTTGCTG(G/-)CCCCCCTgtgagtaccaagacccccatcat

[NM_000088.3](http://www.ncbi.nlm.nih.gov/entrez/viewer.fcgi?db=nucleotide&id=NM_000088.3): c.2451delT

CCCCGGCCCTGCTGGCTTTGCTGGCCCCCC(T/-)gtgagtaccaagacccccatcatttttcat

[NM_000088.3](http://www.ncbi.nlm.nih.gov/entrez/viewer.fcgi?db=nucleotide&id=NM_000088.3): c.2510_2525delATGCTGGTCCCCCTGG

GCGAACCTGGTGATGCTGGTGCTAAAGGCG(ATGCTGGTCCCCCTGG/-)CCCTGCCGGACCCGCTGGACCCCCTGGCCC

[NM_000088.3](http://www.ncbi.nlm.nih.gov/entrez/viewer.fcgi?db=nucleotide&id=NM_000088.3): c.2523delT

TGCTGGTGCTAAAGGCGATGCTGGTCCCCC(T/-)GGCCCTGCCGGACCCGCTGGACCCCCTGGC

[NM_000088.3](http://www.ncbi.nlm.nih.gov/entrez/viewer.fcgi?db=nucleotide&id=NM_000088.3): c.2613delT

CAAAGGTGCTCGCGGCAGCGCTGGTCCCCC(T/-)gtgagtatcacccgcctctctgttgagcct

[NM_000088.3](http://www.ncbi.nlm.nih.gov/entrez/viewer.fcgi?db=nucleotide&id=NM_000088.3): c.2646_2647delAG

TGCTACTGGTTTCCCTGGTGCTGCTGGCCG(AG/-)TCGGTCCTCCTGGCCCCTCTgtaagtctct

[NM_000088.3](http://www.ncbi.nlm.nih.gov/entrez/viewer.fcgi?db=nucleotide&id=NM_000088.3): c.2655_2663delTCCTGGCCC

TTTCCCTGGTGCTGCTGGCCGAGTCGGTCC(TCCTGGCCC/-)CTCTgtaagtctctgcagcagagtccactg

[NM_000088.3](http://www.ncbi.nlm.nih.gov/entrez/viewer.fcgi?db=nucleotide&id=NM_000088.3): c.2684delC

ctatcctcatccagGGAAATGCTGGACCCC(C/-)TGGCCCTCCTGGTCCTGCTGGCAAAGAAGG

[NM_000088.3](http://www.ncbi.nlm.nih.gov/entrez/viewer.fcgi?db=nucleotide&id=NM_000088.3): c.2725_2733delCCCCGTGGT

GGTCCTGCTGGCAAAGAAGGCGGCAAAGGT(CCCCGTGGT/-)GAGACTGGCCCTGCTGGACGTCCTGGTGAA

[NM_000088.3](http://www.ncbi.nlm.nih.gov/entrez/viewer.fcgi?db=nucleotide&id=NM_000088.3): c.2784delT

TCCTGGTGAAGTTGGTCCCCCTGGTCCCCC(T/-)GGCCCTGCTGGCGAGAAAGGATCCCCTGGT

[NM_000088.3](http://www.ncbi.nlm.nih.gov/entrez/viewer.fcgi?db=nucleotide&id=NM_000088.3): c.2991delT

CTCTGGAGCAAGTGGTGAACGTGGTCCCCC(T/-)GGTCCCATGGGCCCCCCTGGATTGGCTGGA

[NM_000088.3](http://www.ncbi.nlm.nih.gov/entrez/viewer.fcgi?db=nucleotide&id=NM_000088.3): c.3008delC

AACGTGGTCCCCCTGGTCCCATGGGCCCCC(C/-)TGGATTGGCTGGACCCCCTGGTGAATCTGG

[NM_000088.3](http://www.ncbi.nlm.nih.gov/entrez/viewer.fcgi?db=nucleotide&id=NM_000088.3): c.3027delT

CATGGGCCCCCCTGGATTGGCTGGACCCCC(T/-)GGTGAATCTGGACGTGAGgtgagcagtccc

[NM_000088.3](http://www.ncbi.nlm.nih.gov/entrez/viewer.fcgi?db=nucleotide&id=NM_000088.3): c.3065delG

tctctctgcagGGGGCTCCTGGTGCCGAAG(G/-)TTCCCCTGGACGAGACGGTTCTCCTGGCGC

[NM_000088.3](http://www.ncbi.nlm.nih.gov/entrez/viewer.fcgi?db=nucleotide&id=NM_000088.3): c.3079delG

GCTCCTGGTGCCGAAGGTTCCCCTGGACGA(G/-)ACGGTTCTCCTGGCGCCAAGgtaagatggc

[NM_000088.3](http://www.ncbi.nlm.nih.gov/entrez/viewer.fcgi?db=nucleotide&id=NM_000088.3): c.3114delG

ctgctctccccgccagGGTGACCGTGGTGA(G/-)ACCGGCCCCGCTGGACCCCCTGGTGCTCCT

[NM_000088.3](http://www.ncbi.nlm.nih.gov/entrez/viewer.fcgi?db=nucleotide&id=NM_000088.3): c.3129_3137delACCCCCTGG

gGGTGACCGTGGTGAGACCGGCCCCGCTGG(ACCCCCTGG/-)TGCTCCTGGTGCTCCTGGTGCCCCTGGCCC

[NM_000088.3](http://www.ncbi.nlm.nih.gov/entrez/viewer.fcgi?db=nucleotide&id=NM_000088.3): c.3132_3140delCCCTGGTGC

TGACCGTGGTGAGACCGGCCCCGCTGGACC(CCCTGGTGC/-)TCCTGGTGCTCCTGGTGCCCCTGGCCCCGT

[NM_000088.3](http://www.ncbi.nlm.nih.gov/entrez/viewer.fcgi?db=nucleotide&id=NM_000088.3): c.3150_3158delTCCTGGTGC

CCCCGCTGGACCCCCTGGTGCTCCTGGTGC(TCCTGGTGC/-)CCCTGGCCCCGTTGGCCCTGCTGGCAAGAG

[NM_000088.3](http://www.ncbi.nlm.nih.gov/entrez/viewer.fcgi?db=nucleotide&id=NM_000088.3): c.3156_3164delTGCCCCTGG

TGGACCCCCTGGTGCTCCTGGTGCTCCTGG(TGCCCCTGG/-)CCCCGTTGGCCCTGCTGGCAAGAGTGGTGA

[NM_000088.3](http://www.ncbi.nlm.nih.gov/entrez/viewer.fcgi?db=nucleotide&id=NM_000088.3): c.3162delT

CCCTGGTGCTCCTGGTGCTCCTGGTGCCCC(T/-)GGCCCCGTTGGCCCTGCTGGCAAGAGTGGT

[NM_000088.3](http://www.ncbi.nlm.nih.gov/entrez/viewer.fcgi?db=nucleotide&id=NM_000088.3): c.3201delT

TGGCCCTGCTGGCAAGAGTGGTGATCGTGG(T/-)GAGACTgtaagtagctgggctccagttccc

[NM_000088.3](http://www.ncbi.nlm.nih.gov/entrez/viewer.fcgi?db=nucleotide&id=NM_000088.3): c.3241delG

CCTGCTGGTCCCACCGGTCCTGTCGGCCCT(G/-)TTGGCGCCCGTGGCCCCGCCgtaagtaccc

[NM_000088.3](http://www.ncbi.nlm.nih.gov/entrez/viewer.fcgi?db=nucleotide&id=NM_000088.3): c.3258delC

TCCTGTCGGCCCTGTTGGCGCCCGTGGCCC(C/-)GCCgtaagtaccctgctgtgtcccccatgc

[NM_000088.3](http://www.ncbi.nlm.nih.gov/entrez/viewer.fcgi?db=nucleotide&id=NM_000088.3): c.3307_3314delGGCGACAG

CGTGGTGACAAGGGTGAGACAGGCGAACAG(GGCGACAG/-)AGGCATAAAGGGTCACCGTGGCTTCTCTGG

[NM_000088.3](http://www.ncbi.nlm.nih.gov/entrez/viewer.fcgi?db=nucleotide&id=NM_000088.3): c.3313delA

GACAAGGGTGAGACAGGCGAACAGGGCGAC(A/-)GAGGCATAAAGGGTCACCGTGGCTTCTCTG

[NM_000088.3](http://www.ncbi.nlm.nih.gov/entrez/viewer.fcgi?db=nucleotide&id=NM_000088.3): c.3371delG

ctgaactgccttttttatctgtccttcagG(G/-)CTCTCCTGGTGAACAAGGTCCCTCTGGAGC

[NM_000088.3](http://www.ncbi.nlm.nih.gov/entrez/viewer.fcgi?db=nucleotide&id=NM_000088.3): c.3402delC

CTCTCCTGGTGAACAAGGTCCCTCTGGAGC(C/-)TCTGGTCCTGCTGGTCCCCGAgtaagtcat

[NM_000088.3](http://www.ncbi.nlm.nih.gov/entrez/viewer.fcgi?db=nucleotide&id=NM_000088.3): c.3477delT

TCCTGGCAAAGATGGACTCAACGGTCTCCC(T/-)GGCCCCATTGGGCCCCCTGGTCCTCGCGGT

[NM_000088.3](http://www.ncbi.nlm.nih.gov/entrez/viewer.fcgi?db=nucleotide&id=NM_000088.3): c.3480_3497delCCCCATTGGGCCCCCTGG

TGGCAAAGATGGACTCAACGGTCTCCCTGG(CCCCATTGGGCCCCCTGG/-)TCCTCGCGGTCGCACTGGTGATGCTGGTCC

[NM_000088.3](http://www.ncbi.nlm.nih.gov/entrez/viewer.fcgi?db=nucleotide&id=NM_000088.3): c.3495delT

CAACGGTCTCCCTGGCCCCATTGGGCCCCC(T/-)GGTCCTCGCGGTCGCACTGGTGATGCTGGT

[NM_000088.3](http://www.ncbi.nlm.nih.gov/entrez/viewer.fcgi?db=nucleotide&id=NM_000088.3): c.3532-3_3532-1delCAG

cggtaatccccactctcttccctctctgtg(cag/-)GGTCCCCCCGGCCCTCCTGGACCTCCTGGT

[NM_000088.3](http://www.ncbi.nlm.nih.gov/entrez/viewer.fcgi?db=nucleotide&id=NM_000088.3): c.3540delC

actctcttccctctctgtgcagGGTCCCCC(C/-)GGCCCTCCTGGACCTCCTGGTCCCCCTGGT

[NM_000088.3](http://www.ncbi.nlm.nih.gov/entrez/viewer.fcgi?db=nucleotide&id=NM_000088.3): c.3560delG

agGGTCCCCCCGGCCCTCCTGGACCTCCTG(G/-)TCCCCCTGGTCCTCCCAGCGCTGGTTTCGA

[NM_000088.3](http://www.ncbi.nlm.nih.gov/entrez/viewer.fcgi?db=nucleotide&id=NM_000088.3): c.3580_3581delGC

GGACCTCCTGGTCCCCCTGGTCCTCCCAGC(GC/-)TGGTTTCGACTTCAGCTTCCTGCCCCAGCC

[NM_000088.3](http://www.ncbi.nlm.nih.gov/entrez/viewer.fcgi?db=nucleotide&id=NM_000088.3): c.3584delG

CTCCTGGTCCCCCTGGTCCTCCCAGCGCTG(G/-)TTTCGACTTCAGCTTCCTGCCCCAGCCACC

[NM_000088.3](http://www.ncbi.nlm.nih.gov/entrez/viewer.fcgi?db=nucleotide&id=NM_000088.3): c.3589_3590delGA

GGTCCCCCTGGTCCTCCCAGCGCTGGTTTC(GA/-)CTTCAGCTTCCTGCCCCAGCCACCTCAAGA

[NM_000088.3](http://www.ncbi.nlm.nih.gov/entrez/viewer.fcgi?db=nucleotide&id=NM_000088.3): c.3638delG

AGCCACCTCAAGAGAAGGCTCACGATGGTG(G/-)CCGCTACTACCGGGCTGATGATGCCAATGT

[NM_000088.3](http://www.ncbi.nlm.nih.gov/entrez/viewer.fcgi?db=nucleotide&id=NM_000088.3): c.3765delC

CCGGAGCCCAGAGGGCAGCCGCAAGAACCC(C/-)GCCCGCACCTGCCGTGACCTCAAGATGTGC

[NM_000088.3](http://www.ncbi.nlm.nih.gov/entrez/viewer.fcgi?db=nucleotide&id=NM_000088.3): c.3782_3794delACCTCAAGATGTG

GCCGCAAGAACCCCGCCCGCACCTGCCGTG(ACCTCAAGATGTG/-)CCACTCTGACTGGAAGAGTGgtgagggcct

[NM_000088.3](http://www.ncbi.nlm.nih.gov/entrez/viewer.fcgi?db=nucleotide&id=NM_000088.3): c.4008_4013delCGAGTA

actcttccctctctcccctcccctgcagTT(CGAGTA/-)TGGCGGCCAGGGCTCCGACCCTGCCGATGT

[NM_000088.3](http://www.ncbi.nlm.nih.gov/entrez/viewer.fcgi?db=nucleotide&id=NM_000088.3): c.4054delC

GGCTCCGACCCTGCCGATGTGGCCATCCAG(C/-)TGACCTTCCTGCGCCTGATGTCCACCGAGG

[NM_000088.3](http://www.ncbi.nlm.nih.gov/entrez/viewer.fcgi?db=nucleotide&id=NM_000088.3): c.4247delC

TCACCTACAGCGTCACTGTCGATGGCTGCA(C/-)Ggtgagtgcccagaatccccaggcagggcc

[NM_000088.3](http://www.ncbi.nlm.nih.gov/entrez/viewer.fcgi?db=nucleotide&id=NM_000088.3): c.4357_4361delGAATT

GCCCCCTTGGACGTTGGTGCCCCAGACCAG(GAATT/-)CGGCTTCGACGTTGGCCCTGTCTGCTTCCT

**Small insertions**

[NM_000088.3](http://www.ncbi.nlm.nih.gov/entrez/viewer.fcgi?db=nucleotide&id=NM_000088.3): c.300_301insC

ctctaacgcgcgtctcttcgtcccctagAG(-/c)TCACCCACCGACCAAGAAACCACCGGCGTC

[NM_000088.3](http://www.ncbi.nlm.nih.gov/entrez/viewer.fcgi?db=nucleotide&id=NM_000088.3): c.386dupC

tctccccctgcagGGACCCGCAGGCCCCCC(-/c)TGGCCGAGATGGCATCCCTGGACAGCCTGG

[NM_000088.3](http://www.ncbi.nlm.nih.gov/entrez/viewer.fcgi?db=nucleotide&id=NM_000088.3): c.390_391insA

cccctgcagGGACCCGCAGGCCCCCCTGGC(-/a)CGAGATGGCATCCCTGGACAGCCTGGACTT

[NM_000088.3](http://www.ncbi.nlm.nih.gov/entrez/viewer.fcgi?db=nucleotide&id=NM_000088.3): c.588+2_588+3insTT

TGGTCTCCCTGGCCCCCCTGGTGCACCTgt(-/tt)gagtatccaggacgtcttcatatgcctcct

[NM_000088.3](http://www.ncbi.nlm.nih.gov/entrez/viewer.fcgi?db=nucleotide&id=NM_000088.3): c.904-1_909dupGGGCCCC

gacttctcttggtttggttgtcagGGCCCC(-/gggcccc)CGTGGCCTGCCTGGTGAGAGAGGTCGCCCT

[NM_000088.3](http://www.ncbi.nlm.nih.gov/entrez/viewer.fcgi?db=nucleotide&id=NM_000088.3): c.1001dupC

TGATGGTGCTACTGGTGCTGCCGGGCCCCC(-/c)Tgtgagtgtggcctgtaggcctcagggcct

[NM_000088.3](http://www.ncbi.nlm.nih.gov/entrez/viewer.fcgi?db=nucleotide&id=NM_000088.3): c.1127dupC

CCAGGGTGTGCGTGGTGAGCCTGGCCCCCC(-/c)TGGCCCTGCTGGTGCTGCTGGCCCTGCTgt

[NM_000088.3](http://www.ncbi.nlm.nih.gov/entrez/viewer.fcgi?db=nucleotide&id=NM_000088.3): c.1379dupC

ccagGGCCCTGTTGGTGTTCAAGGACCCCC(-/c)TGGCCCTGCTGGAGAGGAAGGAAAGCGAGG

[NM_000088.3](http://www.ncbi.nlm.nih.gov/entrez/viewer.fcgi?db=nucleotide&id=NM_000088.3): c.1667dupC

CCCTGGTCCTGATGGCAAAACTGGCCCCCC(-/c)Tgtaagtatcactccccctgaaccccctgc

[NM_000088.3](http://www.ncbi.nlm.nih.gov/entrez/viewer.fcgi?db=nucleotide&id=NM_000088.3): c.1718_1719insAC

TCGCCCCGGACCCCCAGGCCCACCTGGTGC(-/ac)CCGTGGTCAGGCTGGTGTGATGGGATTCCC

[NM_000088.3](http://www.ncbi.nlm.nih.gov/entrez/viewer.fcgi?db=nucleotide&id=NM_000088.3): c.1920dupC

AGAGGTGAACAAGGCCCTGCTGGCTCCCCC(-/c)GGATTCCAGgtgaggcctcatggctgtcag

[NM_000088.3](http://www.ncbi.nlm.nih.gov/entrez/viewer.fcgi?db=nucleotide&id=NM_000088.3): c.2016dupC

GTTCCTGGAGACCTTGGCGCCCCTGGCCCC(-/c)TCTGGAGCAAGAgtaagtaggcctctctcg

[NM_000088.3](http://www.ncbi.nlm.nih.gov/entrez/viewer.fcgi?db=nucleotide&id=NM_000088.3): c.2046_2047insCTCTCTAG

cttcccttctagGGCGAGAGAGGTTTCCCT(-/ctctctag)GGCGAGCGTGGTGTGCAAGGTCCCCCTGGT

[NM_000088.3](http://www.ncbi.nlm.nih.gov/entrez/viewer.fcgi?db=nucleotide&id=NM_000088.3): c.2451dupT

CCCGGCCCTGCTGGCTTTGCTGGCCCCCCT(-/t)gtgagtaccaagacccccatcatttttcat

[NM_000088.3](http://www.ncbi.nlm.nih.gov/entrez/viewer.fcgi?db=nucleotide&id=NM_000088.3): c.2522dupC

TGCTGGTGCTAAAGGCGATGCTGGTCCCCC(-/c)TGGCCCTGCCGGACCCGCTGGACCCCCTGG

[NM_000088.3](http://www.ncbi.nlm.nih.gov/entrez/viewer.fcgi?db=nucleotide&id=NM_000088.3): c.2550dupT

CCTGGCCCTGCCGGACCCGCTGGACCCCCT(-/t)GGCCCCATTgtgagtggcttggccctctgt

[NM_000088.3](http://www.ncbi.nlm.nih.gov/entrez/viewer.fcgi?db=nucleotide&id=NM_000088.3): c.2563_2565dupAAT

tgatgcctctgtgctctcctacagGGTAAT(-/aat)GTTGGTGCTCCTGGAGCCAAAGGTGCTCGC

[NM_000088.3](http://www.ncbi.nlm.nih.gov/entrez/viewer.fcgi?db=nucleotide&id=NM_000088.3): c.3150_3158dupTCCTGGTGC

ACCCCCTGGTGCTCCTGGTGCTCCTGGTGC(-/tcctggtgc)CCCTGGCCCCGTTGGCCCTGCTGGCAAGAG

[NM_000088.3](http://www.ncbi.nlm.nih.gov/entrez/viewer.fcgi?db=nucleotide&id=NM_000088.3): c.3158_3159insTCCTGGTCC

ACCCCCTGGTGCTCCTGGTGCTCCTGGTGC(-/tcctggtcc)CCCTGGCCCCGTTGGCCCTGCTGGCAAGAG

[NM_000088.3](http://www.ncbi.nlm.nih.gov/entrez/viewer.fcgi?db=nucleotide&id=NM_000088.3): c.3285dupC

ttctagGGACCCCAAGGCCCCCGTGGTGAC(-/c)AAGGGTGAGACAGGCGAACAGGGCGACAGA

[NM_000088.3](http://www.ncbi.nlm.nih.gov/entrez/viewer.fcgi?db=nucleotide&id=NM_000088.3): c.3564_3572dupCCCTGGTCC

CCCTCCTGGACCTCCTGGTCCCCCTGGTCC(-/ccctggtcc)TCCCAGCGCTGGTTTCGACTTCAGCTTCCT

[NM_000088.3](http://www.ncbi.nlm.nih.gov/entrez/viewer.fcgi?db=nucleotide&id=NM_000088.3): c.3969dupT

ATCAGCAAGAACCCCAAGGACAAGAGGCAT(-/t)GTCTGGTTCGGCGAGAGCATGACCGATGGA

[NM_000088.3](http://www.ncbi.nlm.nih.gov/entrez/viewer.fcgi?db=nucleotide&id=NM_000088.3): c.4238_4248dupATGGCTGCACG

ACCTACAGCGTCACTGTCGATGGCTGCACG(-/atggctgcacg)gtgagtgcccagaatccccaggcagggccc

[NM_000088.3](http://www.ncbi.nlm.nih.gov/entrez/viewer.fcgi?db=nucleotide&id=NM_000088.3): c.4338dupC

CTGCCCATCATCGATGTGGCCCCCTTGGAC(-/c)GTTGGTGCCCCAGACCAGGAATTCGGCTTC

**Small indels**

[NM_000088.3](http://www.ncbi.nlm.nih.gov/entrez/viewer.fcgi?db=nucleotide&id=NM_000088.3): c.333_333+1delGGinsTC

ACCCACCGACCAAGAAACCACCGGCGTCGA(Gg/tc)taatctcctgccctcgaattttgcccctgc

[NM_000088.3](http://www.ncbi.nlm.nih.gov/entrez/viewer.fcgi?db=nucleotide&id=NM_000088.3): c.672_673delTCinsA

gGGTCCCATGGGTCCCCGAGGTCCCCCAGG(TC/a)CCCCTGGAAAGAATGGAGATGATgtaagta

[NM_000088.3](http://www.ncbi.nlm.nih.gov/entrez/viewer.fcgi?db=nucleotide&id=NM_000088.3): c.997_998delCCinsG

GGAAATGATGGTGCTACTGGTGCTGCCGGG(CC/g)CCCTgtgagtgtggcctgtaggcctcaggg

[NM_000088.3](http://www.ncbi.nlm.nih.gov/entrez/viewer.fcgi?db=nucleotide&id=NM_000088.3): c.1614+2_1614+3delTGinsC

CCGGTGAAGCTGGTCTGCCTGGTGCCAAGg(tg/c)aggccccaggctttcagcctggcttggcca

[NM_000088.3](http://www.ncbi.nlm.nih.gov/entrez/viewer.fcgi?db=nucleotide&id=NM_000088.3): c.1876-2_1876-1delAGinsCT

ctccccgtgactgtagtgttctctctgtgc(ag/ct)GGTCCCGCTGGCGAGAGAGGTGAACAAGGC

[NM_000088.3](http://www.ncbi.nlm.nih.gov/entrez/viewer.fcgi?db=nucleotide&id=NM_000088.3): c.1982_1983delAGinsTT

CTCCAGGTGAAGCAGGCAAACCTGGTGAAC(AG/tt)gtaagagggagcagccggccagaggggtgg
