# Supplementary material for: Development of a High-Throughput Resequencing Array for the Detection of Pathogenic Mutations in Osteogenesis Imperfecta
Source: PLoS One. 2015 Mar 5;10(3):e0119553. doi: 10.1371/journal.pone.0119553 (PMC4350936; doi:10.1371/journal.pone.0119553)
Supplement: S3 Datasets — According to the selected mutations, we have made them into the probes to tiles on the chip. (ZIP) [file pone.0119553.s003.zip › COL1A2.doc]

**Missense/nonsense**

[NM_000089.3](http://www.ncbi.nlm.nih.gov/entrez/viewer.fcgi?db=nucleotide&id=NM_000089.3): c.353G>A; [NP_000080.2](http://www.ncbi.nlm.nih.gov/entrez/viewer.fcgi?db=nucleotide&id=NP_000080.2): p.G118D

agGGCCCTCAAGGTTTCCAAGGACCTGCTG(G/A)TGAGCCTGGTGAACCTGGTCAAACTgtgag

[NM_000089.3](http://www.ncbi.nlm.nih.gov/entrez/viewer.fcgi?db=nucleotide&id=NM_000089.3): c.371G>A; [NP_000080.2](http://www.ncbi.nlm.nih.gov/entrez/viewer.fcgi?db=nucleotide&id=NP_000080.2): p.G124D

AAGGACCTGCTGGTGAGCCTGGTGAACCTG(G/A)TCAAACTgtgagtacatttttccacctttg

[NM_000089.3](http://www.ncbi.nlm.nih.gov/entrez/viewer.fcgi?db=nucleotide&id=NM_000089.3): c.389G>A; [NP_000080.2](http://www.ncbi.nlm.nih.gov/entrez/viewer.fcgi?db=nucleotide&id=NP_000080.2): p.G130D

tgggtctcccattttcttagGGTCCTGCAG(G/A)TGCTCGTGGTCCAGCTGGCCCTCCTGGCAA

[NM_000089.3](http://www.ncbi.nlm.nih.gov/entrez/viewer.fcgi?db=nucleotide&id=NM_000089.3): c.397G>A; [NP_000080.2](http://www.ncbi.nlm.nih.gov/entrez/viewer.fcgi?db=nucleotide&id=NP_000080.2): p.G133S

ccattttcttagGGTCCTGCAGGTGCTCGT(G/A)GTCCAGCTGGCCCTCCTGGCAAGGCTGGTG

[NM_000089.3](http://www.ncbi.nlm.nih.gov/entrez/viewer.fcgi?db=nucleotide&id=NM_000089.3): c.497G>T; [NP_000080.2](http://www.ncbi.nlm.nih.gov/entrez/viewer.fcgi?db=nucleotide&id=NP_000080.2): p.G166V

cttctgtatttctttctaagGGTGCTCGTG(G/T)TTTCCCTGGAACTCCTGGACTTCCTGGCTT

[NM_000089.3](http://www.ncbi.nlm.nih.gov/entrez/viewer.fcgi?db=nucleotide&id=NM_000089.3): c.544C>G; [NP_000080.2](http://www.ncbi.nlm.nih.gov/entrez/viewer.fcgi?db=nucleotide&id=NP_000080.2): p.H182D

cattttataataaggctttcctttcagGGA(C/G)ACAATGGTCTGGATGGATTGAAGGGACAGC

[NM_000089.3](http://www.ncbi.nlm.nih.gov/entrez/viewer.fcgi?db=nucleotide&id=NM_000089.3): c.569G>T; [NP_000080.2](http://www.ncbi.nlm.nih.gov/entrez/viewer.fcgi?db=nucleotide&id=NP_000080.2): p.G190V

agGGACACAATGGTCTGGATGGATTGAAGG(G/T)ACAGCCCGGTGCTCCTGGTGTGAAGgtaaa

[NM_000089.3](http://www.ncbi.nlm.nih.gov/entrez/viewer.fcgi?db=nucleotide&id=NM_000089.3): c.578G>A; [NP_000080.2](http://www.ncbi.nlm.nih.gov/entrez/viewer.fcgi?db=nucleotide&id=NP_000080.2): p.G193D

ATGGTCTGGATGGATTGAAGGGACAGCCCG(G/A)TGCTCCTGGTGTGAAGgtaaatattaaatt

[NM_000089.3](http://www.ncbi.nlm.nih.gov/entrez/viewer.fcgi?db=nucleotide&id=NM_000089.3): c.577G>A; [NP_000080.2](http://www.ncbi.nlm.nih.gov/entrez/viewer.fcgi?db=nucleotide&id=NP_000080.2): p.G193S

AATGGTCTGGATGGATTGAAGGGACAGCCC(G/A)GTGCTCCTGGTGTGAAGgtaaatattaaat

[NM_000089.3](http://www.ncbi.nlm.nih.gov/entrez/viewer.fcgi?db=nucleotide&id=NM_000089.3): c.586G>T; [NP_000080.2](http://www.ncbi.nlm.nih.gov/entrez/viewer.fcgi?db=nucleotide&id=NP_000080.2): p.G196C

GATGGATTGAAGGGACAGCCCGGTGCTCCT(G/T)GTGTGAAGgtaaatattaaattagaagcac

[NM_000089.3](http://www.ncbi.nlm.nih.gov/entrez/viewer.fcgi?db=nucleotide&id=NM_000089.3): c.604G>C; [NP_000080.2](http://www.ncbi.nlm.nih.gov/entrez/viewer.fcgi?db=nucleotide&id=NP_000080.2): p.G202R

actcaatccttctccatgtagGGTGAACCT(G/C)GTGCCCCTGGTGAAAATGGAACTCCAGGTC

[NM_000089.3](http://www.ncbi.nlm.nih.gov/entrez/viewer.fcgi?db=nucleotide&id=NM_000089.3): c.605G>A; [NP_000080.2](http://www.ncbi.nlm.nih.gov/entrez/viewer.fcgi?db=nucleotide&id=NP_000080.2): p.G202D

ctcaatccttctccatgtagGGTGAACCTG(G/A)TGCCCCTGGTGAAAATGGAACTCCAGGTCA

[NM_000089.3](http://www.ncbi.nlm.nih.gov/entrez/viewer.fcgi?db=nucleotide&id=NM_000089.3): c.632G>A; [NP_000080.2](http://www.ncbi.nlm.nih.gov/entrez/viewer.fcgi?db=nucleotide&id=NP_000080.2): p.G211D

CTGGTGCCCCTGGTGAAAATGGAACTCCAG(G/A)TCAAACAgtaagtattgactacttcattgt

[NM_000089.3](http://www.ncbi.nlm.nih.gov/entrez/viewer.fcgi?db=nucleotide&id=NM_000089.3): c.650G>T; [NP_000080.2](http://www.ncbi.nlm.nih.gov/entrez/viewer.fcgi?db=nucleotide&id=NP_000080.2): p.G217V

gaaaatatttctgcttctagGGAGCCCGTG(G/T)GCTTCCTGGTGAGAGAGGACGTGTTGGTGC

[NM_000089.3](http://www.ncbi.nlm.nih.gov/entrez/viewer.fcgi?db=nucleotide&id=NM_000089.3): c.700C>T; [NP_000080.2](http://www.ncbi.nlm.nih.gov/entrez/viewer.fcgi?db=nucleotide&id=NP_000080.2): p.R234C

atcttccctttattttcttcttagGGTGCC(C/T)GTGGCAGTGATGGAAGTGTGGGTCCCGTGG

[NM_000089.3](http://www.ncbi.nlm.nih.gov/entrez/viewer.fcgi?db=nucleotide&id=NM_000089.3): c.739G>C; [NP_000080.2](http://www.ncbi.nlm.nih.gov/entrez/viewer.fcgi?db=nucleotide&id=NP_000080.2): p.G247R

atttcttaccaccttctgctttgatttcag(G/C)GTCCCATTGGGTCTGCTGGCCCTCCAGGCT

[NM_000089.3](http://www.ncbi.nlm.nih.gov/entrez/viewer.fcgi?db=nucleotide&id=NM_000089.3): c.740G>A; [NP_000080.2](http://www.ncbi.nlm.nih.gov/entrez/viewer.fcgi?db=nucleotide&id=NP_000080.2): p.G247D

tttcttaccaccttctgctttgatttcagG(G/A)TCCCATTGGGTCTGCTGGCCCTCCAGGCTT

[NM_000089.3](http://www.ncbi.nlm.nih.gov/entrez/viewer.fcgi?db=nucleotide&id=NM_000089.3): c.758G>A; [NP_000080.2](http://www.ncbi.nlm.nih.gov/entrez/viewer.fcgi?db=nucleotide&id=NP_000080.2): p.G253D

tttgatttcagGGTCCCATTGGGTCTGCTG(G/A)CCCTCCAGGCTTCCCAGGTGCCCCTGGCCC

[NM_000089.3](http://www.ncbi.nlm.nih.gov/entrez/viewer.fcgi?db=nucleotide&id=NM_000089.3): c.767G>A; [NP_000080.2](http://www.ncbi.nlm.nih.gov/entrez/viewer.fcgi?db=nucleotide&id=NP_000080.2): p.G256D

agGGTCCCATTGGGTCTGCTGGCCCTCCAG(G/A)CTTCCCAGGTGCCCCTGGCCCCAAGgtaaa

[NM_000089.3](http://www.ncbi.nlm.nih.gov/entrez/viewer.fcgi?db=nucleotide&id=NM_000089.3): c.767G>T; [NP_000080.2](http://www.ncbi.nlm.nih.gov/entrez/viewer.fcgi?db=nucleotide&id=NP_000080.2): p.G256V

agGGTCCCATTGGGTCTGCTGGCCCTCCAG(G/T)CTTCCCAGGTGCCCCTGGCCCCAAGgtaaa

[NM_000089.3](http://www.ncbi.nlm.nih.gov/entrez/viewer.fcgi?db=nucleotide&id=NM_000089.3): c.794G>A; [NP_000080.2](http://www.ncbi.nlm.nih.gov/entrez/viewer.fcgi?db=nucleotide&id=NP_000080.2): p.G265D

taattccttggtttaatttcctcttttagG(G/A)TGAAATTGGAGCTGTTGGTAACGCTGGTCC

[NM_000089.3](http://www.ncbi.nlm.nih.gov/entrez/viewer.fcgi?db=nucleotide&id=NM_000089.3): c.821G>A; [NP_000080.2](http://www.ncbi.nlm.nih.gov/entrez/viewer.fcgi?db=nucleotide&id=NP_000080.2): p.G274D

agGGTGAAATTGGAGCTGTTGGTAACGCTG(G/A)TCCTGCTGGTCCCGCCGGTCCCCGTGGTGA

[NM_000089.3](http://www.ncbi.nlm.nih.gov/entrez/viewer.fcgi?db=nucleotide&id=NM_000089.3): c.821G>T; [NP_000080.2](http://www.ncbi.nlm.nih.gov/entrez/viewer.fcgi?db=nucleotide&id=NP_000080.2): p.G274V

agGGTGAAATTGGAGCTGTTGGTAACGCTG(G/T)TCCTGCTGGTCCCGCCGGTCCCCGTGGTGA

[NM_000089.3](http://www.ncbi.nlm.nih.gov/entrez/viewer.fcgi?db=nucleotide&id=NM_000089.3): c.838G>A; [NP_000080.2](http://www.ncbi.nlm.nih.gov/entrez/viewer.fcgi?db=nucleotide&id=NP_000080.2): p.G280S

GTTGGTAACGCTGGTCCTGCTGGTCCCGCC(G/A)GTCCCCGTGGTGAAGTGGGTCTTCCAGGCC

[NM_000089.3](http://www.ncbi.nlm.nih.gov/entrez/viewer.fcgi?db=nucleotide&id=NM_000089.3): c.839G>T; [NP_000080.2](http://www.ncbi.nlm.nih.gov/entrez/viewer.fcgi?db=nucleotide&id=NP_000080.2): p.G280V

TTGGTAACGCTGGTCCTGCTGGTCCCGCCG(G/T)TCCCCGTGGTGAAGTGGGTCTTCCAGGCCT

[NM_000089.3](http://www.ncbi.nlm.nih.gov/entrez/viewer.fcgi?db=nucleotide&id=NM_000089.3): c.847G>C; [NP_000080.2](http://www.ncbi.nlm.nih.gov/entrez/viewer.fcgi?db=nucleotide&id=NP_000080.2): p.G283R

GCTGGTCCTGCTGGTCCCGCCGGTCCCCGT(G/C)GTGAAGTGGGTCTTCCAGGCCTCTCCGGCC

[NM_000089.3](http://www.ncbi.nlm.nih.gov/entrez/viewer.fcgi?db=nucleotide&id=NM_000089.3): c.857G>C; [NP_000080.2](http://www.ncbi.nlm.nih.gov/entrez/viewer.fcgi?db=nucleotide&id=NP_000080.2): p.G286A

CTGGTCCCGCCGGTCCCCGTGGTGAAGTGG(G/C)TCTTCCAGGCCTCTCCGGCCCCGTTGGACC

[NM_000089.3](http://www.ncbi.nlm.nih.gov/entrez/viewer.fcgi?db=nucleotide&id=NM_000089.3): c.857G>T; [NP_000080.2](http://www.ncbi.nlm.nih.gov/entrez/viewer.fcgi?db=nucleotide&id=NP_000080.2): p.G286V

CTGGTCCCGCCGGTCCCCGTGGTGAAGTGG(G/T)TCTTCCAGGCCTCTCCGGCCCCGTTGGACC

[NM_000089.3](http://www.ncbi.nlm.nih.gov/entrez/viewer.fcgi?db=nucleotide&id=NM_000089.3): c.866G>C; [NP_000080.2](http://www.ncbi.nlm.nih.gov/entrez/viewer.fcgi?db=nucleotide&id=NP_000080.2): p.G289A

CCGGTCCCCGTGGTGAAGTGGGTCTTCCAG(G/C)CCTCTCCGGCCCCGTTGGACCTCCTgtaag

[NM_000089.3](http://www.ncbi.nlm.nih.gov/entrez/viewer.fcgi?db=nucleotide&id=NM_000089.3): c.866G>T; [NP_000080.2](http://www.ncbi.nlm.nih.gov/entrez/viewer.fcgi?db=nucleotide&id=NP_000080.2): p.G289V

CCGGTCCCCGTGGTGAAGTGGGTCTTCCAG(G/T)CCTCTCCGGCCCCGTTGGACCTCCTgtaag

[NM_000089.3](http://www.ncbi.nlm.nih.gov/entrez/viewer.fcgi?db=nucleotide&id=NM_000089.3): c.884G>T; [NP_000080.2](http://www.ncbi.nlm.nih.gov/entrez/viewer.fcgi?db=nucleotide&id=NP_000080.2): p.G295V

TGGGTCTTCCAGGCCTCTCCGGCCCCGTTG(G/T)ACCTCCTgtaagtagccactgtctttaaac

[NM_000089.3](http://www.ncbi.nlm.nih.gov/entrez/viewer.fcgi?db=nucleotide&id=NM_000089.3): c.892G>C; [NP_000080.2](http://www.ncbi.nlm.nih.gov/entrez/viewer.fcgi?db=nucleotide&id=NP_000080.2): p.G298R

gatatccttctcctttccttttcctcatag(G/C)GTAATCCTGGAGCAAACGGCCTTACTGGTG

[NM_000089.3](http://www.ncbi.nlm.nih.gov/entrez/viewer.fcgi?db=nucleotide&id=NM_000089.3): c.920G>A; [NP_000080.2](http://www.ncbi.nlm.nih.gov/entrez/viewer.fcgi?db=nucleotide&id=NP_000080.2): p.G307D

agGGTAATCCTGGAGCAAACGGCCTTACTG(G/A)TGCCAAGGGTGCTGCTgtgagtatacctgc

[NM_000089.3](http://www.ncbi.nlm.nih.gov/entrez/viewer.fcgi?db=nucleotide&id=NM_000089.3): c.928G>C; [NP_000080.2](http://www.ncbi.nlm.nih.gov/entrez/viewer.fcgi?db=nucleotide&id=NP_000080.2): p.G310R

CCTGGAGCAAACGGCCTTACTGGTGCCAAG(G/C)GTGCTGCTgtgagtatacctgcgtagctaa

[NM_000089.3](http://www.ncbi.nlm.nih.gov/entrez/viewer.fcgi?db=nucleotide&id=NM_000089.3): c.946G>C; [NP_000080.2](http://www.ncbi.nlm.nih.gov/entrez/viewer.fcgi?db=nucleotide&id=NP_000080.2): p.G316R

acactgcattttccttcacagGGCCTTCCC(G/C)GCGTTGCTGGGGCTCCCGGCCTCCCTGGAC

[NM_000089.3](http://www.ncbi.nlm.nih.gov/entrez/viewer.fcgi?db=nucleotide&id=NM_000089.3): c.955G>A; [NP_000080.2](http://www.ncbi.nlm.nih.gov/entrez/viewer.fcgi?db=nucleotide&id=NP_000080.2): p.G319R

tttccttcacagGGCCTTCCCGGCGTTGCT(G/A)GGGCTCCCGGCCTCCCTGGACCCCGCGGTA

[NM_000089.3](http://www.ncbi.nlm.nih.gov/entrez/viewer.fcgi?db=nucleotide&id=NM_000089.3): c.964G>A; [NP_000080.2](http://www.ncbi.nlm.nih.gov/entrez/viewer.fcgi?db=nucleotide&id=NP_000080.2): p.G322S

cagGGCCTTCCCGGCGTTGCTGGGGCTCCC(G/A)GCCTCCCTGGACCCCGCGGTATTCCTGGCC

[NM_000089.3](http://www.ncbi.nlm.nih.gov/entrez/viewer.fcgi?db=nucleotide&id=NM_000089.3): c.974G>A; [NP_000080.2](http://www.ncbi.nlm.nih.gov/entrez/viewer.fcgi?db=nucleotide&id=NP_000080.2): p.G325E

CCGGCGTTGCTGGGGCTCCCGGCCTCCCTG(G/A)ACCCCGCGGTATTCCTGGCCCTGTTGGTGC

[NM_000089.3](http://www.ncbi.nlm.nih.gov/entrez/viewer.fcgi?db=nucleotide&id=NM_000089.3): c.983G>A; [NP_000080.2](http://www.ncbi.nlm.nih.gov/entrez/viewer.fcgi?db=nucleotide&id=NP_000080.2): p.G328D

CTGGGGCTCCCGGCCTCCCTGGACCCCGCG(G/A)TATTCCTGGCCCTGTTGGTGCTGCCGGTGC

[NM_000089.3](http://www.ncbi.nlm.nih.gov/entrez/viewer.fcgi?db=nucleotide&id=NM_000089.3): c.982G>T; [NP_000080.2](http://www.ncbi.nlm.nih.gov/entrez/viewer.fcgi?db=nucleotide&id=NP_000080.2): p.G328C

GCTGGGGCTCCCGGCCTCCCTGGACCCCGC(G/T)GTATTCCTGGCCCTGTTGGTGCTGCCGGTG

[NM_000089.3](http://www.ncbi.nlm.nih.gov/entrez/viewer.fcgi?db=nucleotide&id=NM_000089.3): c.982G>A; [NP_000080.2](http://www.ncbi.nlm.nih.gov/entrez/viewer.fcgi?db=nucleotide&id=NP_000080.2): p.G328S

GCTGGGGCTCCCGGCCTCCCTGGACCCCGC(G/A)GTATTCCTGGCCCTGTTGGTGCTGCCGGTG

[NM_000089.3](http://www.ncbi.nlm.nih.gov/entrez/viewer.fcgi?db=nucleotide&id=NM_000089.3): c.992G>A; [NP_000080.2](http://www.ncbi.nlm.nih.gov/entrez/viewer.fcgi?db=nucleotide&id=NP_000080.2): p.G331D

CCGGCCTCCCTGGACCCCGCGGTATTCCTG(G/A)CCCTGTTGGTGCTGCCGGTGCTACTGGTGC

[NM_000089.3](http://www.ncbi.nlm.nih.gov/entrez/viewer.fcgi?db=nucleotide&id=NM_000089.3): c.992G>T; [NP_000080.2](http://www.ncbi.nlm.nih.gov/entrez/viewer.fcgi?db=nucleotide&id=NP_000080.2): p.G331V

CCGGCCTCCCTGGACCCCGCGGTATTCCTG(G/T)CCCTGTTGGTGCTGCCGGTGCTACTGGTGC

[NM_000089.3](http://www.ncbi.nlm.nih.gov/entrez/viewer.fcgi?db=nucleotide&id=NM_000089.3): c.1000G>A; [NP_000080.2](http://www.ncbi.nlm.nih.gov/entrez/viewer.fcgi?db=nucleotide&id=NP_000080.2): p.G334S

CCTGGACCCCGCGGTATTCCTGGCCCTGTT(G/A)GTGCTGCCGGTGCTACTGGTGCCAGAGGAC

[NM_000089.3](http://www.ncbi.nlm.nih.gov/entrez/viewer.fcgi?db=nucleotide&id=NM_000089.3): c.1009G>C; [NP_000080.2](http://www.ncbi.nlm.nih.gov/entrez/viewer.fcgi?db=nucleotide&id=NP_000080.2): p.G337R

CGCGGTATTCCTGGCCCTGTTGGTGCTGCC(G/C)GTGCTACTGGTGCCAGAGGACTTGTTgtaa

[NM_000089.3](http://www.ncbi.nlm.nih.gov/entrez/viewer.fcgi?db=nucleotide&id=NM_000089.3): c.1009G>T; [NP_000080.2](http://www.ncbi.nlm.nih.gov/entrez/viewer.fcgi?db=nucleotide&id=NP_000080.2): p.G337C

CGCGGTATTCCTGGCCCTGTTGGTGCTGCC(G/T)GTGCTACTGGTGCCAGAGGACTTGTTgtaa

[NM_000089.3](http://www.ncbi.nlm.nih.gov/entrez/viewer.fcgi?db=nucleotide&id=NM_000089.3): c.1009G>A; [NP_000080.2](http://www.ncbi.nlm.nih.gov/entrez/viewer.fcgi?db=nucleotide&id=NP_000080.2): p.G337S

CGCGGTATTCCTGGCCCTGTTGGTGCTGCC(G/A)GTGCTACTGGTGCCAGAGGACTTGTTgtaa

[NM_000089.3](http://www.ncbi.nlm.nih.gov/entrez/viewer.fcgi?db=nucleotide&id=NM_000089.3): c.1018G>C; [NP_000080.2](http://www.ncbi.nlm.nih.gov/entrez/viewer.fcgi?db=nucleotide&id=NP_000080.2): p.G340R

CCTGGCCCTGTTGGTGCTGCCGGTGCTACT(G/C)GTGCCAGAGGACTTGTTgtaagtggtcatg

[NM_000089.3](http://www.ncbi.nlm.nih.gov/entrez/viewer.fcgi?db=nucleotide&id=NM_000089.3): c.1018G>A; [NP_000080.2](http://www.ncbi.nlm.nih.gov/entrez/viewer.fcgi?db=nucleotide&id=NP_000080.2): p.G340S

CCTGGCCCTGTTGGTGCTGCCGGTGCTACT(G/A)GTGCCAGAGGACTTGTTgtaagtggtcatg

[NM_000089.3](http://www.ncbi.nlm.nih.gov/entrez/viewer.fcgi?db=nucleotide&id=NM_000089.3): c.1036G>T; [NP_000080.2](http://www.ncbi.nlm.nih.gov/entrez/viewer.fcgi?db=nucleotide&id=NP_000080.2): p.G346C

ctttgaccactgttctgtattgaaccctag(G/T)GTGAGCCTGGTCCAGCTGGCTCCAAAGGAG

[NM_000089.3](http://www.ncbi.nlm.nih.gov/entrez/viewer.fcgi?db=nucleotide&id=NM_000089.3): c.1036G>A; [NP_000080.2](http://www.ncbi.nlm.nih.gov/entrez/viewer.fcgi?db=nucleotide&id=NP_000080.2): p.G346S

ctttgaccactgttctgtattgaaccctag(G/A)GTGAGCCTGGTCCAGCTGGCTCCAAAGGAG

[NM_000089.3](http://www.ncbi.nlm.nih.gov/entrez/viewer.fcgi?db=nucleotide&id=NM_000089.3): c.1037G>T; [NP_000080.2](http://www.ncbi.nlm.nih.gov/entrez/viewer.fcgi?db=nucleotide&id=NP_000080.2): p.G346V

tttgaccactgttctgtattgaaccctagG(G/T)TGAGCCTGGTCCAGCTGGCTCCAAAGGAGA

[NM_000089.3](http://www.ncbi.nlm.nih.gov/entrez/viewer.fcgi?db=nucleotide&id=NM_000089.3): c.1045G>T; [NP_000080.2](http://www.ncbi.nlm.nih.gov/entrez/viewer.fcgi?db=nucleotide&id=NP_000080.2): p.G349C

ctgttctgtattgaaccctagGGTGAGCCT(G/T)GTCCAGCTGGCTCCAAAGGAGAGAGCGGTA

[NM_000089.3](http://www.ncbi.nlm.nih.gov/entrez/viewer.fcgi?db=nucleotide&id=NM_000089.3): c.1045G>A; [NP_000080.2](http://www.ncbi.nlm.nih.gov/entrez/viewer.fcgi?db=nucleotide&id=NP_000080.2): p.G349S

ctgttctgtattgaaccctagGGTGAGCCT(G/A)GTCCAGCTGGCTCCAAAGGAGAGAGCGGTA

[NM_000089.3](http://www.ncbi.nlm.nih.gov/entrez/viewer.fcgi?db=nucleotide&id=NM_000089.3): c.1072G>T; [NP_000080.2](http://www.ncbi.nlm.nih.gov/entrez/viewer.fcgi?db=nucleotide&id=NP_000080.2): p.G358C

CCTGGTCCAGCTGGCTCCAAAGGAGAGAGC(G/T)GTAACAAGGGTGAGCCCgtaagtagctcta

[NM_000089.3](http://www.ncbi.nlm.nih.gov/entrez/viewer.fcgi?db=nucleotide&id=NM_000089.3): c.1072G>A; [NP_000080.2](http://www.ncbi.nlm.nih.gov/entrez/viewer.fcgi?db=nucleotide&id=NP_000080.2): p.G358S

CCTGGTCCAGCTGGCTCCAAAGGAGAGAGC(G/A)GTAACAAGGGTGAGCCCgtaagtagctcta

[NM_000089.3](http://www.ncbi.nlm.nih.gov/entrez/viewer.fcgi?db=nucleotide&id=NM_000089.3): c.1090G>A; [NP_000080.2](http://www.ncbi.nlm.nih.gov/entrez/viewer.fcgi?db=nucleotide&id=NP_000080.2): p.G364S

ccctcttcttttgttcttttcattaaacag(G/A)GCTCTGCTGGGCCCCAAGGTCCTCCTGGTC

[NM_000089.3](http://www.ncbi.nlm.nih.gov/entrez/viewer.fcgi?db=nucleotide&id=NM_000089.3): c.1099G>T; [NP_000080.2](http://www.ncbi.nlm.nih.gov/entrez/viewer.fcgi?db=nucleotide&id=NP_000080.2): p.G367W

tttgttcttttcattaaacagGGCTCTGCT(G/T)GGCCCCAAGGTCCTCCTGGTCCCAGTGGTG

[NM_000089.3](http://www.ncbi.nlm.nih.gov/entrez/viewer.fcgi?db=nucleotide&id=NM_000089.3): c.1109G>C; [NP_000080.2](http://www.ncbi.nlm.nih.gov/entrez/viewer.fcgi?db=nucleotide&id=NP_000080.2): p.G370A

tcattaaacagGGCTCTGCTGGGCCCCAAG(G/C)TCCTCCTGGTCCCAGTGGTGAAGAAGGAAA

[NM_000089.3](http://www.ncbi.nlm.nih.gov/entrez/viewer.fcgi?db=nucleotide&id=NM_000089.3): c.1108G>A; [NP_000080.2](http://www.ncbi.nlm.nih.gov/entrez/viewer.fcgi?db=nucleotide&id=NP_000080.2): p.G370S

ttcattaaacagGGCTCTGCTGGGCCCCAA(G/A)GTCCTCCTGGTCCCAGTGGTGAAGAAGGAA

[NM_000089.3](http://www.ncbi.nlm.nih.gov/entrez/viewer.fcgi?db=nucleotide&id=NM_000089.3): c.1127G>A; [NP_000080.2](http://www.ncbi.nlm.nih.gov/entrez/viewer.fcgi?db=nucleotide&id=NP_000080.2): p.G376D

CTGGGCCCCAAGGTCCTCCTGGTCCCAGTG(G/A)TGAAGAAGGAAAGAGAGGCCCTAATGGGGA

[NM_000089.3](http://www.ncbi.nlm.nih.gov/entrez/viewer.fcgi?db=nucleotide&id=NM_000089.3): c.1127G>T; [NP_000080.2](http://www.ncbi.nlm.nih.gov/entrez/viewer.fcgi?db=nucleotide&id=NP_000080.2): p.G376V

CTGGGCCCCAAGGTCCTCCTGGTCCCAGTG(G/T)TGAAGAAGGAAAGAGAGGCCCTAATGGGGA

[NM_000089.3](http://www.ncbi.nlm.nih.gov/entrez/viewer.fcgi?db=nucleotide&id=NM_000089.3): c.1154G>A; [NP_000080.2](http://www.ncbi.nlm.nih.gov/entrez/viewer.fcgi?db=nucleotide&id=NP_000080.2): p.G385E

GTGGTGAAGAAGGAAAGAGAGGCCCTAATG(G/A)GGAAGCTGGATCTGCCGGCCCTCCAGGACC

[NM_000089.3](http://www.ncbi.nlm.nih.gov/entrez/viewer.fcgi?db=nucleotide&id=NM_000089.3): c.1163G>T; [NP_000080.2](http://www.ncbi.nlm.nih.gov/entrez/viewer.fcgi?db=nucleotide&id=NP_000080.2): p.G388V

AAGGAAAGAGAGGCCCTAATGGGGAAGCTG(G/T)ATCTGCCGGCCCTCCAGGACCTCCTGGGCT

[NM_000089.3](http://www.ncbi.nlm.nih.gov/entrez/viewer.fcgi?db=nucleotide&id=NM_000089.3): c.1171G>T; [NP_000080.2](http://www.ncbi.nlm.nih.gov/entrez/viewer.fcgi?db=nucleotide&id=NP_000080.2): p.G391C

AGAGGCCCTAATGGGGAAGCTGGATCTGCC(G/T)GCCCTCCAGGACCTCCTGGGCTGAGAgtag

[NM_000089.3](http://www.ncbi.nlm.nih.gov/entrez/viewer.fcgi?db=nucleotide&id=NM_000089.3): c.1171G>A; [NP_000080.2](http://www.ncbi.nlm.nih.gov/entrez/viewer.fcgi?db=nucleotide&id=NP_000080.2): p.G391S

AGAGGCCCTAATGGGGAAGCTGGATCTGCC(G/A)GCCCTCCAGGACCTCCTGGGCTGAGAgtag

[NM_000089.3](http://www.ncbi.nlm.nih.gov/entrez/viewer.fcgi?db=nucleotide&id=NM_000089.3): c.1190G>A; [NP_000080.2](http://www.ncbi.nlm.nih.gov/entrez/viewer.fcgi?db=nucleotide&id=NP_000080.2): p.G397E

CTGGATCTGCCGGCCCTCCAGGACCTCCTG(G/A)GCTGAGAgtaggtttcaaatgctcccaaca

[NM_000089.3](http://www.ncbi.nlm.nih.gov/entrez/viewer.fcgi?db=nucleotide&id=NM_000089.3): c.1216G>A; [NP_000080.2](http://www.ncbi.nlm.nih.gov/entrez/viewer.fcgi?db=nucleotide&id=NP_000080.2): p.G406S

actttcttgcagGGTAGTCCTGGTTCTCGT(G/A)GTCTTCCTGGAGCTGATGGCAGAGCTGGCG

[NM_000089.3](http://www.ncbi.nlm.nih.gov/entrez/viewer.fcgi?db=nucleotide&id=NM_000089.3): c.1226G>T; [NP_000080.2](http://www.ncbi.nlm.nih.gov/entrez/viewer.fcgi?db=nucleotide&id=NP_000080.2): p.G409V

agGGTAGTCCTGGTTCTCGTGGTCTTCCTG(G/T)AGCTGATGGCAGAGCTGGCGTCATGgtaag

[NM_000089.3](http://www.ncbi.nlm.nih.gov/entrez/viewer.fcgi?db=nucleotide&id=NM_000089.3): c.1253G>A; [NP_000080.2](http://www.ncbi.nlm.nih.gov/entrez/viewer.fcgi?db=nucleotide&id=NP_000080.2): p.G418D

ctatctgttttttttttttttttgaatagG(G/A)CCCTCCTGGTAGTCGTGGTGCAAGTGGCCC

[NM_000089.3](http://www.ncbi.nlm.nih.gov/entrez/viewer.fcgi?db=nucleotide&id=NM_000089.3): c.1252G>A; [NP_000080.2](http://www.ncbi.nlm.nih.gov/entrez/viewer.fcgi?db=nucleotide&id=NP_000080.2): p.G418S

tctatctgttttttttttttttttgaatag(G/A)GCCCTCCTGGTAGTCGTGGTGCAAGTGGCC

[NM_000089.3](http://www.ncbi.nlm.nih.gov/entrez/viewer.fcgi?db=nucleotide&id=NM_000089.3): c.1270G>A; [NP_000080.2](http://www.ncbi.nlm.nih.gov/entrez/viewer.fcgi?db=nucleotide&id=NP_000080.2): p.G424S

ttttttgaatagGGCCCTCCTGGTAGTCGT(G/A)GTGCAAGTGGCCCTGCTGGAGTCCGAGGAC

[NM_000089.3](http://www.ncbi.nlm.nih.gov/entrez/viewer.fcgi?db=nucleotide&id=NM_000089.3): c.1279G>A; [NP_000080.2](http://www.ncbi.nlm.nih.gov/entrez/viewer.fcgi?db=nucleotide&id=NP_000080.2): p.G427S

tagGGCCCTCCTGGTAGTCGTGGTGCAAGT(G/A)GCCCTGCTGGAGTCCGAGGACCTAATGGAG

[NM_000089.3](http://www.ncbi.nlm.nih.gov/entrez/viewer.fcgi?db=nucleotide&id=NM_000089.3): c.1298G>A; [NP_000080.2](http://www.ncbi.nlm.nih.gov/entrez/viewer.fcgi?db=nucleotide&id=NP_000080.2): p.G433E

GTGGTGCAAGTGGCCCTGCTGGAGTCCGAG(G/A)ACCTAATGGAGATGCTGGTCGCCCTGGGGA

[NM_000089.3](http://www.ncbi.nlm.nih.gov/entrez/viewer.fcgi?db=nucleotide&id=NM_000089.3): c.1333G>C; [NP_000080.2](http://www.ncbi.nlm.nih.gov/entrez/viewer.fcgi?db=nucleotide&id=NP_000080.2): p.G445R

AATGGAGATGCTGGTCGCCCTGGGGAGCCT(G/C)GTCTCATGGGACCCAGAgtaagtttcaaac

[NM_000089.3](http://www.ncbi.nlm.nih.gov/entrez/viewer.fcgi?db=nucleotide&id=NM_000089.3): c.1343G>A; [NP_000080.2](http://www.ncbi.nlm.nih.gov/entrez/viewer.fcgi?db=nucleotide&id=NP_000080.2): p.G448E

CTGGTCGCCCTGGGGAGCCTGGTCTCATGG(G/A)ACCCAGAgtaagtttcaaactgattctgag

[NM_000089.3](http://www.ncbi.nlm.nih.gov/entrez/viewer.fcgi?db=nucleotide&id=NM_000089.3): c.1360G>T; [NP_000080.2](http://www.ncbi.nlm.nih.gov/entrez/viewer.fcgi?db=nucleotide&id=NP_000080.2): p.G454C

acatcctcatttattttatagGGTCTTCCT(G/T)GTTCCCCTGGAAATATCGGCCCCGCTGGAA

[NM_000089.3](http://www.ncbi.nlm.nih.gov/entrez/viewer.fcgi?db=nucleotide&id=NM_000089.3): c.1378G>A; [NP_000080.2](http://www.ncbi.nlm.nih.gov/entrez/viewer.fcgi?db=nucleotide&id=NP_000080.2): p.G460S

tagGGTCTTCCTGGTTCCCCTGGAAATATC(G/A)GCCCCGCTGGAAAAGAAGGTCCTGTCgtaa

[NM_000089.3](http://www.ncbi.nlm.nih.gov/entrez/viewer.fcgi?db=nucleotide&id=NM_000089.3): c.1406G>C; [NP_000080.2](http://www.ncbi.nlm.nih.gov/entrez/viewer.fcgi?db=nucleotide&id=NP_000080.2): p.G469A

gagtaaacttgaaataactctgctttcagG(G/C)CCTCCCTGGCATCGACGGCAGGCCTGGCCC

[NM_000089.3](http://www.ncbi.nlm.nih.gov/entrez/viewer.fcgi?db=nucleotide&id=NM_000089.3): c.1433G>A; [NP_000080.2](http://www.ncbi.nlm.nih.gov/entrez/viewer.fcgi?db=nucleotide&id=NP_000080.2): p.G478D

agGGCCTCCCTGGCATCGACGGCAGGCCTG(G/A)CCCAATTGGCCCAGCTGGAGCAAGAGGAGA

[NM_000089.3](http://www.ncbi.nlm.nih.gov/entrez/viewer.fcgi?db=nucleotide&id=NM_000089.3): c.1433G>T; [NP_000080.2](http://www.ncbi.nlm.nih.gov/entrez/viewer.fcgi?db=nucleotide&id=NP_000080.2): p.G478V

agGGCCTCCCTGGCATCGACGGCAGGCCTG(G/T)CCCAATTGGCCCAGCTGGAGCAAGAGGAGA

[NM_000089.3](http://www.ncbi.nlm.nih.gov/entrez/viewer.fcgi?db=nucleotide&id=NM_000089.3): c.1451G>A; [NP_000080.2](http://www.ncbi.nlm.nih.gov/entrez/viewer.fcgi?db=nucleotide&id=NP_000080.2): p.G484E

ACGGCAGGCCTGGCCCAATTGGCCCAGCTG(G/A)AGCAAGAGGAGAGCCTGGCAACATTGGATT

[NM_000089.3](http://www.ncbi.nlm.nih.gov/entrez/viewer.fcgi?db=nucleotide&id=NM_000089.3): c.1451G>T; [NP_000080.2](http://www.ncbi.nlm.nih.gov/entrez/viewer.fcgi?db=nucleotide&id=NP_000080.2): p.G484V

ACGGCAGGCCTGGCCCAATTGGCCCAGCTG(G/T)AGCAAGAGGAGAGCCTGGCAACATTGGATT

[NM_000089.3](http://www.ncbi.nlm.nih.gov/entrez/viewer.fcgi?db=nucleotide&id=NM_000089.3): c.1478G>A; [NP_000080.2](http://www.ncbi.nlm.nih.gov/entrez/viewer.fcgi?db=nucleotide&id=NP_000080.2): p.G493E

CTGGAGCAAGAGGAGAGCCTGGCAACATTG(G/A)ATTCCCTGGACCCAAAGGCCCCACTgtaag

[NM_000089.3](http://www.ncbi.nlm.nih.gov/entrez/viewer.fcgi?db=nucleotide&id=NM_000089.3): c.1496G>T; [NP_000080.2](http://www.ncbi.nlm.nih.gov/entrez/viewer.fcgi?db=nucleotide&id=NP_000080.2): p.G499V

CTGGCAACATTGGATTCCCTGGACCCAAAG(G/T)CCCCACTgtaagaatcaccacaactttctt

[NM_000089.3](http://www.ncbi.nlm.nih.gov/entrez/viewer.fcgi?db=nucleotide&id=NM_000089.3): c.1523G>A; [NP_000080.2](http://www.ncbi.nlm.nih.gov/entrez/viewer.fcgi?db=nucleotide&id=NP_000080.2): p.G508D

ttttactctagGGTGATCCTGGCAAAAACG(G/A)TGATAAAGGTCATGCTGGTCTTGCTGGTGC

[NM_000089.3](http://www.ncbi.nlm.nih.gov/entrez/viewer.fcgi?db=nucleotide&id=NM_000089.3): c.1532G>A; [NP_000080.2](http://www.ncbi.nlm.nih.gov/entrez/viewer.fcgi?db=nucleotide&id=NP_000080.2): p.G511D

agGGTGATCCTGGCAAAAACGGTGATAAAG(G/A)TCATGCTGGTCTTGCTGGTGCTCGGgtagg[NM_000089.3](http://www.ncbi.nlm.nih.gov/entrez/viewer.fcgi?db=nucleotide&id=NM_000089.3): c.1531G>A; [NP_000080.2](http://www.ncbi.nlm.nih.gov/entrez/viewer.fcgi?db=nucleotide&id=NP_000080.2): p.G511S

tagGGTGATCCTGGCAAAAACGGTGATAAA(G/A)GTCATGCTGGTCTTGCTGGTGCTCGGgtag

[NM_000089.3](http://www.ncbi.nlm.nih.gov/entrez/viewer.fcgi?db=nucleotide&id=NM_000089.3): c.1532G>T; [NP_000080.2](http://www.ncbi.nlm.nih.gov/entrez/viewer.fcgi?db=nucleotide&id=NP_000080.2): p.G511V

agGGTGATCCTGGCAAAAACGGTGATAAAG(G/T)TCATGCTGGTCTTGCTGGTGCTCGGgtagg

[NM_000089.3](http://www.ncbi.nlm.nih.gov/entrez/viewer.fcgi?db=nucleotide&id=NM_000089.3): c.1549G>C; [NP_000080.2](http://www.ncbi.nlm.nih.gov/entrez/viewer.fcgi?db=nucleotide&id=NP_000080.2): p.G517R

AACGGTGATAAAGGTCATGCTGGTCTTGCT(G/C)GTGCTCGGgtaggtgctaacttgtgtacag

[NM_000089.3](http://www.ncbi.nlm.nih.gov/entrez/viewer.fcgi?db=nucleotide&id=NM_000089.3): c.1558G>A; [NP_000080.2](http://www.ncbi.nlm.nih.gov/entrez/viewer.fcgi?db=nucleotide&id=NP_000080.2): p.G520S

accatcagcctttctgttaaatatttttag(G/A)GTGCTCCAGGTCCTGATGGAAACAATGGTG

[NM_000089.3](http://www.ncbi.nlm.nih.gov/entrez/viewer.fcgi?db=nucleotide&id=NM_000089.3): c.1576G>A; [NP_000080.2](http://www.ncbi.nlm.nih.gov/entrez/viewer.fcgi?db=nucleotide&id=NP_000080.2): p.G526R

aaatatttttagGGTGCTCCAGGTCCTGAT(G/A)GAAACAATGGTGCTCAGGGACCTCCTGGAC

[NM_000089.3](http://www.ncbi.nlm.nih.gov/entrez/viewer.fcgi?db=nucleotide&id=NM_000089.3): c.1577G>A; [NP_000080.2](http://www.ncbi.nlm.nih.gov/entrez/viewer.fcgi?db=nucleotide&id=NP_000080.2): p.G526E

aatatttttagGGTGCTCCAGGTCCTGATG(G/A)AAACAATGGTGCTCAGGGACCTCCTGGACC

[NM_000089.3](http://www.ncbi.nlm.nih.gov/entrez/viewer.fcgi?db=nucleotide&id=NM_000089.3): c.1585G>T; [NP_000080.2](http://www.ncbi.nlm.nih.gov/entrez/viewer.fcgi?db=nucleotide&id=NP_000080.2): p.G529C

tagGGTGCTCCAGGTCCTGATGGAAACAAT(G/T)GTGCTCAGGGACCTCCTGGACCACAGgtga

[NM_000089.3](http://www.ncbi.nlm.nih.gov/entrez/viewer.fcgi?db=nucleotide&id=NM_000089.3): c.1595G>A; [NP_000080.2](http://www.ncbi.nlm.nih.gov/entrez/viewer.fcgi?db=nucleotide&id=NP_000080.2): p.G532E

CAGGTCCTGATGGAAACAATGGTGCTCAGG(G/A)ACCTCCTGGACCACAGgtgagtatttctcc

[NM_000089.3](http://www.ncbi.nlm.nih.gov/entrez/viewer.fcgi?db=nucleotide&id=NM_000089.3): c.1621G>T; [NP_000080.2](http://www.ncbi.nlm.nih.gov/entrez/viewer.fcgi?db=nucleotide&id=NP_000080.2): p.G541C

tctctttcctgtcactttcagGGTGTTCAA(G/T)GTGGAAAAGGTGAACAGGGTCCCCCTGGTC

[NM_000089.3](http://www.ncbi.nlm.nih.gov/entrez/viewer.fcgi?db=nucleotide&id=NM_000089.3): c.1630G>A; [NP_000080.2](http://www.ncbi.nlm.nih.gov/entrez/viewer.fcgi?db=nucleotide&id=NP_000080.2): p.G544S

tgtcactttcagGGTGTTCAAGGTGGAAAA(G/A)GTGAACAGGGTCCCCCTGGTCCTCCAGGCT

[NM_000089.3](http://www.ncbi.nlm.nih.gov/entrez/viewer.fcgi?db=nucleotide&id=NM_000089.3): c.1631G>T; [NP_000080.2](http://www.ncbi.nlm.nih.gov/entrez/viewer.fcgi?db=nucleotide&id=NP_000080.2): p.G544V

gtcactttcagGGTGTTCAAGGTGGAAAAG(G/T)TGAACAGGGTCCCCCTGGTCCTCCAGGCTT

[NM_000089.3](http://www.ncbi.nlm.nih.gov/entrez/viewer.fcgi?db=nucleotide&id=NM_000089.3): c.1639G>C; [NP_000080.2](http://www.ncbi.nlm.nih.gov/entrez/viewer.fcgi?db=nucleotide&id=NP_000080.2): p.G547R

cagGGTGTTCAAGGTGGAAAAGGTGAACAG(G/C)GTCCCCCTGGTCCTCCAGGCTTCCAGgtaa

[NM_000089.3](http://www.ncbi.nlm.nih.gov/entrez/viewer.fcgi?db=nucleotide&id=NM_000089.3): c.1645G>C; [NP_000080.2](http://www.ncbi.nlm.nih.gov/entrez/viewer.fcgi?db=nucleotide&id=NP_000080.2): p.A549P

GTTCAAGGTGGAAAAGGTGAACAGGGTCCC(G/C)CTGGTCCTCCAGGCTTCCAGgtaagtcaac

[NM_000089.3](http://www.ncbi.nlm.nih.gov/entrez/viewer.fcgi?db=nucleotide&id=NM_000089.3): c.1648G>A; [NP_000080.2](http://www.ncbi.nlm.nih.gov/entrez/viewer.fcgi?db=nucleotide&id=NP_000080.2): p.G550S

CAAGGTGGAAAAGGTGAACAGGGTCCCCCT(G/A)GTCCTCCAGGCTTCCAGgtaagtcaactca

[NM_000089.3](http://www.ncbi.nlm.nih.gov/entrez/viewer.fcgi?db=nucleotide&id=NM_000089.3): c.1658G>A; [NP_000080.2](http://www.ncbi.nlm.nih.gov/entrez/viewer.fcgi?db=nucleotide&id=NP_000080.2): p.G553D

AAGGTGAACAGGGTCCCCCTGGTCCTCCAG(G/A)CTTCCAGgtaagtcaactcaaacatataca

[NM_000089.3](http://www.ncbi.nlm.nih.gov/entrez/viewer.fcgi?db=nucleotide&id=NM_000089.3): c.1684G>T; [NP_000080.2](http://www.ncbi.nlm.nih.gov/entrez/viewer.fcgi?db=nucleotide&id=NP_000080.2): p.G562C

tttcatttttagGGTCTGCCTGGCCCCTCA(G/T)GTCCCGCTGGTGAAGTTGGCAAACCAGGAG

[NM_000089.3](http://www.ncbi.nlm.nih.gov/entrez/viewer.fcgi?db=nucleotide&id=NM_000089.3): c.1685G>T; [NP_000080.2](http://www.ncbi.nlm.nih.gov/entrez/viewer.fcgi?db=nucleotide&id=NP_000080.2): p.G562V

ttcatttttagGGTCTGCCTGGCCCCTCAG(G/T)TCCCGCTGGTGAAGTTGGCAAACCAGGAGA

[NM_000089.3](http://www.ncbi.nlm.nih.gov/entrez/viewer.fcgi?db=nucleotide&id=NM_000089.3): c.1711G>A; [NP_000080.2](http://www.ncbi.nlm.nih.gov/entrez/viewer.fcgi?db=nucleotide&id=NP_000080.2): p.G571R

TCAGGTCCCGCTGGTGAAGTTGGCAAACCA(G/A)GAGAAAGGgtgagtaaaacaagtaatagta

[NM_000089.3](http://www.ncbi.nlm.nih.gov/entrez/viewer.fcgi?db=nucleotide&id=NM_000089.3): c.1756G>C; [NP_000080.2](http://www.ncbi.nlm.nih.gov/entrez/viewer.fcgi?db=nucleotide&id=NP_000080.2): p.G586R

CATGGTGAGTTTGGTCTCCCTGGTCCTGCT(G/C)GTCCAAGAgtaagtgttacttcattaactt

[NM_000089.3](http://www.ncbi.nlm.nih.gov/entrez/viewer.fcgi?db=nucleotide&id=NM_000089.3): c.1774G>A; [NP_000080.2](http://www.ncbi.nlm.nih.gov/entrez/viewer.fcgi?db=nucleotide&id=NP_000080.2): p.G592S

tcttctaatcacttttttcagGGGGAACGC(G/A)GTCCCCCAGGTGAGAGTGGTGCTGCCGGTC

[NM_000089.3](http://www.ncbi.nlm.nih.gov/entrez/viewer.fcgi?db=nucleotide&id=NM_000089.3): c.1783G>T; [NP_000080.2](http://www.ncbi.nlm.nih.gov/entrez/viewer.fcgi?db=nucleotide&id=NP_000080.2): p.G595C

cacttttttcagGGGGAACGCGGTCCCCCA(G/T)GTGAGAGTGGTGCTGCCGGTCCTACTGGTC

[NM_000089.3](http://www.ncbi.nlm.nih.gov/entrez/viewer.fcgi?db=nucleotide&id=NM_000089.3): c.1783G>A; [NP_000080.2](http://www.ncbi.nlm.nih.gov/entrez/viewer.fcgi?db=nucleotide&id=NP_000080.2): p.G595S

cacttttttcagGGGGAACGCGGTCCCCCA(G/A)GTGAGAGTGGTGCTGCCGGTCCTACTGGTC

[NM_000089.3](http://www.ncbi.nlm.nih.gov/entrez/viewer.fcgi?db=nucleotide&id=NM_000089.3): c.1793G>T; [NP_000080.2](http://www.ncbi.nlm.nih.gov/entrez/viewer.fcgi?db=nucleotide&id=NP_000080.2): p.G598V

agGGGGAACGCGGTCCCCCAGGTGAGAGTG(G/T)TGCTGCCGGTCCTACTGGTCCTATTGGAAG

[NM_000089.3](http://www.ncbi.nlm.nih.gov/entrez/viewer.fcgi?db=nucleotide&id=NM_000089.3): c.1802G>A; [NP_000080.2](http://www.ncbi.nlm.nih.gov/entrez/viewer.fcgi?db=nucleotide&id=NP_000080.2): p.G601D

GCGGTCCCCCAGGTGAGAGTGGTGCTGCCG(G/A)TCCTACTGGTCCTATTGGAAGCCGAGGTCC

[NM_000089.3](http://www.ncbi.nlm.nih.gov/entrez/viewer.fcgi?db=nucleotide&id=NM_000089.3): c.1801G>A; [NP_000080.2](http://www.ncbi.nlm.nih.gov/entrez/viewer.fcgi?db=nucleotide&id=NP_000080.2): p.G601S

CGCGGTCCCCCAGGTGAGAGTGGTGCTGCC(G/A)GTCCTACTGGTCCTATTGGAAGCCGAGGTC

[NM_000089.3](http://www.ncbi.nlm.nih.gov/entrez/viewer.fcgi?db=nucleotide&id=NM_000089.3): c.1874G>A; [NP_000080.2](http://www.ncbi.nlm.nih.gov/entrez/viewer.fcgi?db=nucleotide&id=NP_000080.2): p.G625D

tcacatgtgtttgactcaagGGTGAACCTG(G/A)TGTGGTTGGTGCTGTGGGCACTGCTGGTCC

[NM_000089.3](http://www.ncbi.nlm.nih.gov/entrez/viewer.fcgi?db=nucleotide&id=NM_000089.3): c.1882G>C; [NP_000080.2](http://www.ncbi.nlm.nih.gov/entrez/viewer.fcgi?db=nucleotide&id=NP_000080.2): p.G628R

gtttgactcaagGGTGAACCTGGTGTGGTT(G/C)GTGCTGTGGGCACTGCTGGTCCATCTGGTC

[NM_000089.3](http://www.ncbi.nlm.nih.gov/entrez/viewer.fcgi?db=nucleotide&id=NM_000089.3): c.1901G>T; [NP_000080.2](http://www.ncbi.nlm.nih.gov/entrez/viewer.fcgi?db=nucleotide&id=NP_000080.2): p.G634V

CTGGTGTGGTTGGTGCTGTGGGCACTGCTG(G/T)TCCATCTGGTCCTAGTGGACTCCCAGGAGA

[NM_000089.3](http://www.ncbi.nlm.nih.gov/entrez/viewer.fcgi?db=nucleotide&id=NM_000089.3): c.1910G>A; [NP_000080.2](http://www.ncbi.nlm.nih.gov/entrez/viewer.fcgi?db=nucleotide&id=NP_000080.2): p.G637D

TTGGTGCTGTGGGCACTGCTGGTCCATCTG(G/A)TCCTAGTGGACTCCCAGGAGAGAGGGGTGC

[NM_000089.3](http://www.ncbi.nlm.nih.gov/entrez/viewer.fcgi?db=nucleotide&id=NM_000089.3): c.1919G>A; [NP_000080.2](http://www.ncbi.nlm.nih.gov/entrez/viewer.fcgi?db=nucleotide&id=NP_000080.2): p.G640E

TGGGCACTGCTGGTCCATCTGGTCCTAGTG(G/A)ACTCCCAGGAGAGAGGGGTGCTGCTGGCAT

[NM_000089.3](http://www.ncbi.nlm.nih.gov/entrez/viewer.fcgi?db=nucleotide&id=NM_000089.3): c.1936G>T; [NP_000080.2](http://www.ncbi.nlm.nih.gov/entrez/viewer.fcgi?db=nucleotide&id=NP_000080.2): p.G646C

TCTGGTCCTAGTGGACTCCCAGGAGAGAGG(G/T)GTGCTGCTGGCATACCTGGAGGCAAGGGAG

[NM_000089.3](http://www.ncbi.nlm.nih.gov/entrez/viewer.fcgi?db=nucleotide&id=NM_000089.3): c.1937G>T; [NP_000080.2](http://www.ncbi.nlm.nih.gov/entrez/viewer.fcgi?db=nucleotide&id=NP_000080.2): p.G646V

CTGGTCCTAGTGGACTCCCAGGAGAGAGGG(G/T)TGCTGCTGGCATACCTGGAGGCAAGGGAGA

[NM_000089.3](http://www.ncbi.nlm.nih.gov/entrez/viewer.fcgi?db=nucleotide&id=NM_000089.3): c.1981G>T; [NP_000080.2](http://www.ncbi.nlm.nih.gov/entrez/viewer.fcgi?db=nucleotide&id=NP_000080.2): p.G661C

gctcttgctttatactttcagGGTGAACCT(G/T)GTCTCAGAGGTGAAATTGGTAACCCTGGCA

[NM_000089.3](http://www.ncbi.nlm.nih.gov/entrez/viewer.fcgi?db=nucleotide&id=NM_000089.3): c.1981G>A; [NP_000080.2](http://www.ncbi.nlm.nih.gov/entrez/viewer.fcgi?db=nucleotide&id=NP_000080.2): p.G661S

gctcttgctttatactttcagGGTGAACCT(G/A)GTCTCAGAGGTGAAATTGGTAACCCTGGCA

[NM_000089.3](http://www.ncbi.nlm.nih.gov/entrez/viewer.fcgi?db=nucleotide&id=NM_000089.3): c.1982G>T; [NP_000080.2](http://www.ncbi.nlm.nih.gov/entrez/viewer.fcgi?db=nucleotide&id=NP_000080.2): p.G661V

ctcttgctttatactttcagGGTGAACCTG(G/T)TCTCAGAGGTGAAATTGGTAACCCTGGCAG

[NM_000089.3](http://www.ncbi.nlm.nih.gov/entrez/viewer.fcgi?db=nucleotide&id=NM_000089.3): c.1991G>A; [NP_000080.2](http://www.ncbi.nlm.nih.gov/entrez/viewer.fcgi?db=nucleotide&id=NP_000080.2): p.G664D

tatactttcagGGTGAACCTGGTCTCAGAG(G/A)TGAAATTGGTAACCCTGGCAGAGATGGTGC

[NM_000089.3](http://www.ncbi.nlm.nih.gov/entrez/viewer.fcgi?db=nucleotide&id=NM_000089.3): c.2009G>A; [NP_000080.2](http://www.ncbi.nlm.nih.gov/entrez/viewer.fcgi?db=nucleotide&id=NP_000080.2): p.G670D

CTGGTCTCAGAGGTGAAATTGGTAACCCTG(G/A)CAGAGATGGTGCTCGTgtgagtagaatttt

[NM_000089.3](http://www.ncbi.nlm.nih.gov/entrez/viewer.fcgi?db=nucleotide&id=NM_000089.3): c.2026G>C; [NP_000080.2](http://www.ncbi.nlm.nih.gov/entrez/viewer.fcgi?db=nucleotide&id=NP_000080.2): p.G676R

taataagacatgtttcctttttggtactag(G/C)GTGCTCCTGGTGCTGTAGGTGCCCCTGGTC

[NM_000089.3](http://www.ncbi.nlm.nih.gov/entrez/viewer.fcgi?db=nucleotide&id=NM_000089.3): c.2027G>A; [NP_000080.2](http://www.ncbi.nlm.nih.gov/entrez/viewer.fcgi?db=nucleotide&id=NP_000080.2): p.G676D

aataagacatgtttcctttttggtactagG(G/A)TGCTCCTGGTGCTGTAGGTGCCCCTGGTCC

[NM_000089.3](http://www.ncbi.nlm.nih.gov/entrez/viewer.fcgi?db=nucleotide&id=NM_000089.3): c.2027G>T; [NP_000080.2](http://www.ncbi.nlm.nih.gov/entrez/viewer.fcgi?db=nucleotide&id=NP_000080.2): p.G676V

aataagacatgtttcctttttggtactagG(G/T)TGCTCCTGGTGCTGTAGGTGCCCCTGGTCC

[NM_000089.3](http://www.ncbi.nlm.nih.gov/entrez/viewer.fcgi?db=nucleotide&id=NM_000089.3): c.2045G>A; [NP_000080.2](http://www.ncbi.nlm.nih.gov/entrez/viewer.fcgi?db=nucleotide&id=NP_000080.2): p.G682D

tttggtactagGGTGCTCCTGGTGCTGTAG(G/A)TGCCCCTGGTCCTGCTGGAGCCACAGGTGA

[NM_000089.3](http://www.ncbi.nlm.nih.gov/entrez/viewer.fcgi?db=nucleotide&id=NM_000089.3): c.2099G>A; [NP_000080.2](http://www.ncbi.nlm.nih.gov/entrez/viewer.fcgi?db=nucleotide&id=NP_000080.2): p.G700D

tcccattatagGGCGAAGCTGGGGCTGCTG(G/A)TCCTGCTGGTCCTGCTGGTCCTCGGGGAAG

[NM_000089.3](http://www.ncbi.nlm.nih.gov/entrez/viewer.fcgi?db=nucleotide&id=NM_000089.3): c.2098G>T; [NP_000080.2](http://www.ncbi.nlm.nih.gov/entrez/viewer.fcgi?db=nucleotide&id=NP_000080.2): p.G700C

gtcccattatagGGCGAAGCTGGGGCTGCT(G/T)GTCCTGCTGGTCCTGCTGGTCCTCGGGGAA

[NM_000089.3](http://www.ncbi.nlm.nih.gov/entrez/viewer.fcgi?db=nucleotide&id=NM_000089.3): c.2123G>A; [NP_000080.2](http://www.ncbi.nlm.nih.gov/entrez/viewer.fcgi?db=nucleotide&id=NP_000080.2): p.R708Q

CTGCTGGTCCTGCTGGTCCTGCTGGTCCTC(G/A)GGGAAGCCCTgtaagtaagaacctgggtca

[NM_000089.3](http://www.ncbi.nlm.nih.gov/entrez/viewer.fcgi?db=nucleotide&id=NM_000089.3): c.2135G>A; [NP_000080.2](http://www.ncbi.nlm.nih.gov/entrez/viewer.fcgi?db=nucleotide&id=NP_000080.2): p.G712D

cacatttttaaatcccttctcccacctagG(G/A)TGAACGTGGTGAGGTCGGTCCTGCTGGCCC

[NM_000089.3](http://www.ncbi.nlm.nih.gov/entrez/viewer.fcgi?db=nucleotide&id=NM_000089.3): c.2134G>A; [NP_000080.2](http://www.ncbi.nlm.nih.gov/entrez/viewer.fcgi?db=nucleotide&id=NP_000080.2): p.G712S

acacatttttaaatcccttctcccacctag(G/A)GTGAACGTGGTGAGGTCGGTCCTGCTGGCC

[NM_000089.3](http://www.ncbi.nlm.nih.gov/entrez/viewer.fcgi?db=nucleotide&id=NM_000089.3): c.2144G>A; [NP_000080.2](http://www.ncbi.nlm.nih.gov/entrez/viewer.fcgi?db=nucleotide&id=NP_000080.2): p.G715D

aaatcccttctcccacctagGGTGAACGTG(G/A)TGAGGTCGGTCCTGCTGGCCCCAATGGATT

[NM_000089.3](http://www.ncbi.nlm.nih.gov/entrez/viewer.fcgi?db=nucleotide&id=NM_000089.3): c.2152G>A; [NP_000080.2](http://www.ncbi.nlm.nih.gov/entrez/viewer.fcgi?db=nucleotide&id=NP_000080.2): p.G718S

tctcccacctagGGTGAACGTGGTGAGGTC(G/A)GTCCTGCTGGCCCCAATGGATTTGCTGGTC

[NM_000089.3](http://www.ncbi.nlm.nih.gov/entrez/viewer.fcgi?db=nucleotide&id=NM_000089.3): c.2161G>A; [NP_000080.2](http://www.ncbi.nlm.nih.gov/entrez/viewer.fcgi?db=nucleotide&id=NP_000080.2): p.G721S

tagGGTGAACGTGGTGAGGTCGGTCCTGCT(G/A)GCCCCAATGGATTTGCTGGTCCTGCTgtga

[NM_000089.3](http://www.ncbi.nlm.nih.gov/entrez/viewer.fcgi?db=nucleotide&id=NM_000089.3): c.2188G>T; [NP_000080.2](http://www.ncbi.nlm.nih.gov/entrez/viewer.fcgi?db=nucleotide&id=NP_000080.2): p.G730C

accagagctgtaactgtttatttccaacag(G/T)GTGCTGCTGGTCAACCTGGTGCTAAAGGAG

[NM_000089.3](http://www.ncbi.nlm.nih.gov/entrez/viewer.fcgi?db=nucleotide&id=NM_000089.3): c.2197G>T; [NP_000080.2](http://www.ncbi.nlm.nih.gov/entrez/viewer.fcgi?db=nucleotide&id=NP_000080.2): p.G733C

gtaactgtttatttccaacagGGTGCTGCT(G/T)GTCAACCTGGTGCTAAAGGAGAAAGAGGAG

[NM_000089.3](http://www.ncbi.nlm.nih.gov/entrez/viewer.fcgi?db=nucleotide&id=NM_000089.3): c.2206G>T; [NP_000080.2](http://www.ncbi.nlm.nih.gov/entrez/viewer.fcgi?db=nucleotide&id=NP_000080.2): p.G736C

tatttccaacagGGTGCTGCTGGTCAACCT(G/T)GTGCTAAAGGAGAAAGAGGAGCCAAAGGGC

[NM_000089.3](http://www.ncbi.nlm.nih.gov/entrez/viewer.fcgi?db=nucleotide&id=NM_000089.3): c.2215G>A; [NP_000080.2](http://www.ncbi.nlm.nih.gov/entrez/viewer.fcgi?db=nucleotide&id=NP_000080.2): p.G739R

cagGGTGCTGCTGGTCAACCTGGTGCTAAA(G/A)GAGAAAGAGGAGCCAAAGGGCCTAAGGGTG

[NM_000089.3](http://www.ncbi.nlm.nih.gov/entrez/viewer.fcgi?db=nucleotide&id=NM_000089.3): c.2215G>C; [NP_000080.2](http://www.ncbi.nlm.nih.gov/entrez/viewer.fcgi?db=nucleotide&id=NP_000080.2): p.G739R

cagGGTGCTGCTGGTCAACCTGGTGCTAAA(G/C)GAGAAAGAGGAGCCAAAGGGCCTAAGGGTG

[NM_000089.3](http://www.ncbi.nlm.nih.gov/entrez/viewer.fcgi?db=nucleotide&id=NM_000089.3): c.2234G>A; [NP_000080.2](http://www.ncbi.nlm.nih.gov/entrez/viewer.fcgi?db=nucleotide&id=NP_000080.2): p.G745E

CTGGTGCTAAAGGAGAAAGAGGAGCCAAAG(G/A)GCCTAAGGGTGAAAACGGTGTTGTTGGTCC

[NM_000089.3](http://www.ncbi.nlm.nih.gov/entrez/viewer.fcgi?db=nucleotide&id=NM_000089.3): c.2243G>T; [NP_000080.2](http://www.ncbi.nlm.nih.gov/entrez/viewer.fcgi?db=nucleotide&id=NP_000080.2): p.G748V

AAGGAGAAAGAGGAGCCAAAGGGCCTAAGG(G/T)TGAAAACGGTGTTGTTGGTCCCACAGGCCC

[NM_000089.3](http://www.ncbi.nlm.nih.gov/entrez/viewer.fcgi?db=nucleotide&id=NM_000089.3): c.2251G>A; [NP_000080.2](http://www.ncbi.nlm.nih.gov/entrez/viewer.fcgi?db=nucleotide&id=NP_000080.2): p.G751S

AGAGGAGCCAAAGGGCCTAAGGGTGAAAAC(G/A)GTGTTGTTGGTCCCACAGGCCCCGTTGGAG

[NM_000089.3](http://www.ncbi.nlm.nih.gov/entrez/viewer.fcgi?db=nucleotide&id=NM_000089.3): c.2260G>T; [NP_000080.2](http://www.ncbi.nlm.nih.gov/entrez/viewer.fcgi?db=nucleotide&id=NP_000080.2): p.G754C

AAAGGGCCTAAGGGTGAAAACGGTGTTGTT(G/T)GTCCCACAGGCCCCGTTGGAGCTGCTGGCC

[NM_000089.3](http://www.ncbi.nlm.nih.gov/entrez/viewer.fcgi?db=nucleotide&id=NM_000089.3): c.2270G>T; [NP_000080.2](http://www.ncbi.nlm.nih.gov/entrez/viewer.fcgi?db=nucleotide&id=NP_000080.2): p.G757V

AGGGTGAAAACGGTGTTGTTGGTCCCACAG(G/T)CCCCGTTGGAGCTGCTGGCCCAGCTgtaag

[NM_000089.3](http://www.ncbi.nlm.nih.gov/entrez/viewer.fcgi?db=nucleotide&id=NM_000089.3): c.2288G>A; [NP_000080.2](http://www.ncbi.nlm.nih.gov/entrez/viewer.fcgi?db=nucleotide&id=NP_000080.2): p.G763D

TTGGTCCCACAGGCCCCGTTGGAGCTGCTG(G/A)CCCAGCTgtaagttgaattcactggtggtc

[NM_000089.3](http://www.ncbi.nlm.nih.gov/entrez/viewer.fcgi?db=nucleotide&id=NM_000089.3): c.2288G>T; [NP_000080.2](http://www.ncbi.nlm.nih.gov/entrez/viewer.fcgi?db=nucleotide&id=NP_000080.2): p.G763V

TTGGTCCCACAGGCCCCGTTGGAGCTGCTG(G/T)CCCAGCTgtaagttgaattcactggtggtc

[NM_000089.3](http://www.ncbi.nlm.nih.gov/entrez/viewer.fcgi?db=nucleotide&id=NM_000089.3): c.2296G>A; [NP_000080.2](http://www.ncbi.nlm.nih.gov/entrez/viewer.fcgi?db=nucleotide&id=NP_000080.2): p.G766S

atttgactccatctttttgtttgcatttag(G/A)GTCCAAATGGTCCCCCCGGTCCTGCTGGAA

[NM_000089.3](http://www.ncbi.nlm.nih.gov/entrez/viewer.fcgi?db=nucleotide&id=NM_000089.3): c.2297G>T; [NP_000080.2](http://www.ncbi.nlm.nih.gov/entrez/viewer.fcgi?db=nucleotide&id=NP_000080.2): p.G766V

tttgactccatctttttgtttgcatttagG(G/T)TCCAAATGGTCCCCCCGGTCCTGCTGGAAG

[NM_000089.3](http://www.ncbi.nlm.nih.gov/entrez/viewer.fcgi?db=nucleotide&id=NM_000089.3): c.2314G>A; [NP_000080.2](http://www.ncbi.nlm.nih.gov/entrez/viewer.fcgi?db=nucleotide&id=NP_000080.2): p.G772S

gtttgcatttagGGTCCAAATGGTCCCCCC(G/A)GTCCTGCTGGAAGTCGTGGTGATGGAGGCC

[NM_000089.3](http://www.ncbi.nlm.nih.gov/entrez/viewer.fcgi?db=nucleotide&id=NM_000089.3): c.2332G>A; [NP_000080.2](http://www.ncbi.nlm.nih.gov/entrez/viewer.fcgi?db=nucleotide&id=NP_000080.2): p.G778S

AATGGTCCCCCCGGTCCTGCTGGAAGTCGT(G/A)GTGATGGAGGCCCCCCTgtgagtatttaca

[NM_000089.3](http://www.ncbi.nlm.nih.gov/entrez/viewer.fcgi?db=nucleotide&id=NM_000089.3): c.2350G>C; [NP_000080.2](http://www.ncbi.nlm.nih.gov/entrez/viewer.fcgi?db=nucleotide&id=NP_000080.2): p.G784R

ctgtggaattctaatgtgcttggctcttag(G/C)GTATGACTGGTTTCCCTGGTGCTGCTGGAC

[NM_000089.3](http://www.ncbi.nlm.nih.gov/entrez/viewer.fcgi?db=nucleotide&id=NM_000089.3): c.2350G>A; [NP_000080.2](http://www.ncbi.nlm.nih.gov/entrez/viewer.fcgi?db=nucleotide&id=NP_000080.2): p.G784S

ctgtggaattctaatgtgcttggctcttag(G/A)GTATGACTGGTTTCCCTGGTGCTGCTGGAC

[NM_000089.3](http://www.ncbi.nlm.nih.gov/entrez/viewer.fcgi?db=nucleotide&id=NM_000089.3): c.2360G>A; [NP_000080.2](http://www.ncbi.nlm.nih.gov/entrez/viewer.fcgi?db=nucleotide&id=NP_000080.2): p.G787D

ctaatgtgcttggctcttagGGTATGACTG(G/A)TTTCCCTGGTGCTGCTGGACGGACTGGTCC

[NM_000089.3](http://www.ncbi.nlm.nih.gov/entrez/viewer.fcgi?db=nucleotide&id=NM_000089.3): c.2359G>T; [NP_000080.2](http://www.ncbi.nlm.nih.gov/entrez/viewer.fcgi?db=nucleotide&id=NP_000080.2): p.G787C

tctaatgtgcttggctcttagGGTATGACT(G/T)GTTTCCCTGGTGCTGCTGGACGGACTGGTC

[NM_000089.3](http://www.ncbi.nlm.nih.gov/entrez/viewer.fcgi?db=nucleotide&id=NM_000089.3): c.2369G>A; [NP_000080.2](http://www.ncbi.nlm.nih.gov/entrez/viewer.fcgi?db=nucleotide&id=NP_000080.2): p.G790D

ttggctcttagGGTATGACTGGTTTCCCTG(G/A)TGCTGCTGGACGGACTGGTCCCCCAGGACC

[NM_000089.3](http://www.ncbi.nlm.nih.gov/entrez/viewer.fcgi?db=nucleotide&id=NM_000089.3): c.2377G>A; [NP_000080.2](http://www.ncbi.nlm.nih.gov/entrez/viewer.fcgi?db=nucleotide&id=NP_000080.2): p.G793R

tagGGTATGACTGGTTTCCCTGGTGCTGCT(G/A)GACGGACTGGTCCCCCAGGACCCTCTgtaa

[NM_000089.3](http://www.ncbi.nlm.nih.gov/entrez/viewer.fcgi?db=nucleotide&id=NM_000089.3): c.2377G>C; [NP_000080.2](http://www.ncbi.nlm.nih.gov/entrez/viewer.fcgi?db=nucleotide&id=NP_000080.2): p.G793R

tagGGTATGACTGGTTTCCCTGGTGCTGCT(G/C)GACGGACTGGTCCCCCAGGACCCTCTgtaa

[NM_000089.3](http://www.ncbi.nlm.nih.gov/entrez/viewer.fcgi?db=nucleotide&id=NM_000089.3): c.2387G>C; [NP_000080.2](http://www.ncbi.nlm.nih.gov/entrez/viewer.fcgi?db=nucleotide&id=NP_000080.2): p.G796A

CTGGTTTCCCTGGTGCTGCTGGACGGACTG(G/C)TCCCCCAGGACCCTCTgtaagtaaatcact

[NM_000089.3](http://www.ncbi.nlm.nih.gov/entrez/viewer.fcgi?db=nucleotide&id=NM_000089.3): c.2386G>T; [NP_000080.2](http://www.ncbi.nlm.nih.gov/entrez/viewer.fcgi?db=nucleotide&id=NP_000080.2): p.G796C

ACTGGTTTCCCTGGTGCTGCTGGACGGACT(G/T)GTCCCCCAGGACCCTCTgtaagtaaatcac

[NM_000089.3](http://www.ncbi.nlm.nih.gov/entrez/viewer.fcgi?db=nucleotide&id=NM_000089.3): c.2386G>A; [NP_000080.2](http://www.ncbi.nlm.nih.gov/entrez/viewer.fcgi?db=nucleotide&id=NP_000080.2): p.G796S

ACTGGTTTCCCTGGTGCTGCTGGACGGACT(G/A)GTCCCCCAGGACCCTCTgtaagtaaatcac

[NM_000089.3](http://www.ncbi.nlm.nih.gov/entrez/viewer.fcgi?db=nucleotide&id=NM_000089.3): c.2413G>C; [NP_000080.2](http://www.ncbi.nlm.nih.gov/entrez/viewer.fcgi?db=nucleotide&id=NP_000080.2): p.G805R

accttgtacatttgctcatagGGTATTTCT(G/C)GCCCTCCTGGTCCCCCTGGTCCTGCTGGGA

[NM_000089.3](http://www.ncbi.nlm.nih.gov/entrez/viewer.fcgi?db=nucleotide&id=NM_000089.3): c.2413G>T; [NP_000080.2](http://www.ncbi.nlm.nih.gov/entrez/viewer.fcgi?db=nucleotide&id=NP_000080.2): p.G805C

accttgtacatttgctcatagGGTATTTCT(G/T)GCCCTCCTGGTCCCCCTGGTCCTGCTGGGA

[NM_000089.3](http://www.ncbi.nlm.nih.gov/entrez/viewer.fcgi?db=nucleotide&id=NM_000089.3): c.2428C>T; [NP_000080.2](http://www.ncbi.nlm.nih.gov/entrez/viewer.fcgi?db=nucleotide&id=NP_000080.2): p.P810S

tcatagGGTATTTCTGGCCCTCCTGGTCCC(C/T)CTGGTCCTGCTGGGAAAGAAGGGCTTCGTG

[NM_000089.3](http://www.ncbi.nlm.nih.gov/entrez/viewer.fcgi?db=nucleotide&id=NM_000089.3): c.2432G>C; [NP_000080.2](http://www.ncbi.nlm.nih.gov/entrez/viewer.fcgi?db=nucleotide&id=NP_000080.2): p.G811A

agGGTATTTCTGGCCCTCCTGGTCCCCCTG(G/C)TCCTGCTGGGAAAGAAGGGCTTCGTGGTCC

[NM_000089.3](http://www.ncbi.nlm.nih.gov/entrez/viewer.fcgi?db=nucleotide&id=NM_000089.3): c.2450G>T; [NP_000080.2](http://www.ncbi.nlm.nih.gov/entrez/viewer.fcgi?db=nucleotide&id=NP_000080.2): p.G817V

CTGGTCCCCCTGGTCCTGCTGGGAAAGAAG(G/T)GCTTCGTGGTCCTCGTGGTGACCAAGGTCC

[NM_000089.3](http://www.ncbi.nlm.nih.gov/entrez/viewer.fcgi?db=nucleotide&id=NM_000089.3): c.2458G>A; [NP_000080.2](http://www.ncbi.nlm.nih.gov/entrez/viewer.fcgi?db=nucleotide&id=NP_000080.2): p.G820S

CCTGGTCCTGCTGGGAAAGAAGGGCTTCGT(G/A)GTCCTCGTGGTGACCAAGGTCCAGTTGGCC

[NM_000089.3](http://www.ncbi.nlm.nih.gov/entrez/viewer.fcgi?db=nucleotide&id=NM_000089.3): c.2467G>T; [NP_000080.2](http://www.ncbi.nlm.nih.gov/entrez/viewer.fcgi?db=nucleotide&id=NP_000080.2): p.G823C

GCTGGGAAAGAAGGGCTTCGTGGTCCTCGT(G/T)GTGACCAAGGTCCAGTTGGCCGAACTGGAG

[NM_000089.3](http://www.ncbi.nlm.nih.gov/entrez/viewer.fcgi?db=nucleotide&id=NM_000089.3): c.2485G>A; [NP_000080.2](http://www.ncbi.nlm.nih.gov/entrez/viewer.fcgi?db=nucleotide&id=NP_000080.2): p.G829S

CGTGGTCCTCGTGGTGACCAAGGTCCAGTT(G/A)GCCGAACTGGAGAAGTAGGTGCAGTTGGTC

[NM_000089.3](http://www.ncbi.nlm.nih.gov/entrez/viewer.fcgi?db=nucleotide&id=NM_000089.3): c.2503G>A; [NP_000080.2](http://www.ncbi.nlm.nih.gov/entrez/viewer.fcgi?db=nucleotide&id=NP_000080.2): p.G835S

CAAGGTCCAGTTGGCCGAACTGGAGAAGTA(G/A)GTGCAGTTGGTCCCCCTGGCTTCGCTGGTG

[NM_000089.3](http://www.ncbi.nlm.nih.gov/entrez/viewer.fcgi?db=nucleotide&id=NM_000089.3): c.2521G>A; [NP_000080.2](http://www.ncbi.nlm.nih.gov/entrez/viewer.fcgi?db=nucleotide&id=NP_000080.2): p.G841S

ACTGGAGAAGTAGGTGCAGTTGGTCCCCCT(G/A)GCTTCGCTGGTGAGAAGGGTCCCTCTGGAG

[NM_000089.3](http://www.ncbi.nlm.nih.gov/entrez/viewer.fcgi?db=nucleotide&id=NM_000089.3): c.2539G>C; [NP_000080.2](http://www.ncbi.nlm.nih.gov/entrez/viewer.fcgi?db=nucleotide&id=NP_000080.2): p.G847R

GTTGGTCCCCCTGGCTTCGCTGGTGAGAAG(G/C)GTCCCTCTGGAGAGGCTGGTACTGCTgtaa

[NM_000089.3](http://www.ncbi.nlm.nih.gov/entrez/viewer.fcgi?db=nucleotide&id=NM_000089.3): c.2558G>A; [NP_000080.2](http://www.ncbi.nlm.nih.gov/entrez/viewer.fcgi?db=nucleotide&id=NP_000080.2): p.G853D

CTGGTGAGAAGGGTCCCTCTGGAGAGGCTG(G/A)TACTGCTgtaagtgatttccaactcctctt

[NM_000089.3](http://www.ncbi.nlm.nih.gov/entrez/viewer.fcgi?db=nucleotide&id=NM_000089.3): c.2558G>T; [NP_000080.2](http://www.ncbi.nlm.nih.gov/entrez/viewer.fcgi?db=nucleotide&id=NP_000080.2): p.G853V

CTGGTGAGAAGGGTCCCTCTGGAGAGGCTG(G/T)TACTGCTgtaagtgatttccaactcctctt

[NM_000089.3](http://www.ncbi.nlm.nih.gov/entrez/viewer.fcgi?db=nucleotide&id=NM_000089.3): c.2566G>A; [NP_000080.2](http://www.ncbi.nlm.nih.gov/entrez/viewer.fcgi?db=nucleotide&id=NP_000080.2): p.G856R

cataatactcagtattttttctctatttag(G/A)GACCTCCTGGCACTCCAGGTCCTCAGGGTC

[NM_000089.3](http://www.ncbi.nlm.nih.gov/entrez/viewer.fcgi?db=nucleotide&id=NM_000089.3): c.2567G>T; [NP_000080.2](http://www.ncbi.nlm.nih.gov/entrez/viewer.fcgi?db=nucleotide&id=NP_000080.2): p.G856V

ataatactcagtattttttctctatttagG(G/T)ACCTCCTGGCACTCCAGGTCCTCAGGGTCT

[NM_000089.3](http://www.ncbi.nlm.nih.gov/entrez/viewer.fcgi?db=nucleotide&id=NM_000089.3): c.2575G>A; [NP_000080.2](http://www.ncbi.nlm.nih.gov/entrez/viewer.fcgi?db=nucleotide&id=NP_000080.2): p.G859S

cagtattttttctctatttagGGACCTCCT(G/A)GCACTCCAGGTCCTCAGGGTCTTCTTGGTG

[NM_000089.3](http://www.ncbi.nlm.nih.gov/entrez/viewer.fcgi?db=nucleotide&id=NM_000089.3): c.2629G>T; [NP_000080.2](http://www.ncbi.nlm.nih.gov/entrez/viewer.fcgi?db=nucleotide&id=NP_000080.2): p.G877C

CTTGGTGCTCCTGGTATTCTGGGTCTCCCT(G/T)GCTCGAGAGGTGAACGTGGTCTACCAGGTG

[NM_000089.3](http://www.ncbi.nlm.nih.gov/entrez/viewer.fcgi?db=nucleotide&id=NM_000089.3): c.2638G>A; [NP_000080.2](http://www.ncbi.nlm.nih.gov/entrez/viewer.fcgi?db=nucleotide&id=NP_000080.2): p.G880S

CCTGGTATTCTGGGTCTCCCTGGCTCGAGA(G/A)GTGAACGTGGTCTACCAGGTGTTGCTGGTG

[NM_000089.3](http://www.ncbi.nlm.nih.gov/entrez/viewer.fcgi?db=nucleotide&id=NM_000089.3): c.2675G>A; [NP_000080.2](http://www.ncbi.nlm.nih.gov/entrez/viewer.fcgi?db=nucleotide&id=NP_000080.2): p.G892D

atgtcattttatcttctctgcctgtttagG(G/A)TGAACCTGGTCCTCTTGGCATTGCCGGCCC

[NM_000089.3](http://www.ncbi.nlm.nih.gov/entrez/viewer.fcgi?db=nucleotide&id=NM_000089.3): c.2684G>A; [NP_000080.2](http://www.ncbi.nlm.nih.gov/entrez/viewer.fcgi?db=nucleotide&id=NP_000080.2): p.G895D

tatcttctctgcctgtttagGGTGAACCTG(G/A)TCCTCTTGGCATTGCCGGCCCTCCTGGGGC

[NM_000089.3](http://www.ncbi.nlm.nih.gov/entrez/viewer.fcgi?db=nucleotide&id=NM_000089.3): c.2701G>A; [NP_000080.2](http://www.ncbi.nlm.nih.gov/entrez/viewer.fcgi?db=nucleotide&id=NP_000080.2): p.G901S

tagGGTGAACCTGGTCCTCTTGGCATTGCC(G/A)GCCCTCCTGGGGCCCGTGGTCCTCCTGGTG

[NM_000089.3](http://www.ncbi.nlm.nih.gov/entrez/viewer.fcgi?db=nucleotide&id=NM_000089.3): c.2710G>C; [NP_000080.2](http://www.ncbi.nlm.nih.gov/entrez/viewer.fcgi?db=nucleotide&id=NP_000080.2): p.G904R

CCTGGTCCTCTTGGCATTGCCGGCCCTCCT(G/C)GGGCCCGTGGTCCTCCTGGTGCTGTGGGTA

[NM_000089.3](http://www.ncbi.nlm.nih.gov/entrez/viewer.fcgi?db=nucleotide&id=NM_000089.3): c.2746G>A; [NP_000080.2](http://www.ncbi.nlm.nih.gov/entrez/viewer.fcgi?db=nucleotide&id=NP_000080.2): p.G916R

CGTGGTCCTCCTGGTGCTGTGGGTAGTCCT(G/A)GAGTCAACGGTGCTCCTGGTGAAGCTGGTC

[NM_000089.3](http://www.ncbi.nlm.nih.gov/entrez/viewer.fcgi?db=nucleotide&id=NM_000089.3): c.2746G>C; [NP_000080.2](http://www.ncbi.nlm.nih.gov/entrez/viewer.fcgi?db=nucleotide&id=NP_000080.2): p.G916R

CGTGGTCCTCCTGGTGCTGTGGGTAGTCCT(G/C)GAGTCAACGGTGCTCCTGGTGAAGCTGGTC

[NM_000089.3](http://www.ncbi.nlm.nih.gov/entrez/viewer.fcgi?db=nucleotide&id=NM_000089.3): c.2773G>T; [NP_000080.2](http://www.ncbi.nlm.nih.gov/entrez/viewer.fcgi?db=nucleotide&id=NP_000080.2): p.G925C

CCTGGAGTCAACGGTGCTCCTGGTGAAGCT(G/T)GTCGTGATgtgagtccaacacttggtttgt

[NM_000089.3](http://www.ncbi.nlm.nih.gov/entrez/viewer.fcgi?db=nucleotide&id=NM_000089.3): c.2801G>A; [NP_000080.2](http://www.ncbi.nlm.nih.gov/entrez/viewer.fcgi?db=nucleotide&id=NP_000080.2): p.G934D

tgtcttcacagGGCAACCCTGGGAACGATG(G/A)TCCCCCAGGTCGCGATGGTCAACCCGGACA

[NM_000089.3](http://www.ncbi.nlm.nih.gov/entrez/viewer.fcgi?db=nucleotide&id=NM_000089.3): c.2809G>T; [NP_000080.2](http://www.ncbi.nlm.nih.gov/entrez/viewer.fcgi?db=nucleotide&id=NP_000080.2): p.G937C

cagGGCAACCCTGGGAACGATGGTCCCCCA(G/T)GTCGCGATGGTCAACCCGGACACAAGgtca

[NM_000089.3](http://www.ncbi.nlm.nih.gov/entrez/viewer.fcgi?db=nucleotide&id=NM_000089.3): c.2836G>A; [NP_000080.2](http://www.ncbi.nlm.nih.gov/entrez/viewer.fcgi?db=nucleotide&id=NP_000080.2): p.G946R

tgatgctaagcttcattttgcctttggtag(G/A)GAGAGCGCGGTTACCCTGGCAATATTGGTC

[NM_000089.3](http://www.ncbi.nlm.nih.gov/entrez/viewer.fcgi?db=nucleotide&id=NM_000089.3): c.2845G>A; [NP_000080.2](http://www.ncbi.nlm.nih.gov/entrez/viewer.fcgi?db=nucleotide&id=NP_000080.2): p.G949S

gcttcattttgcctttggtagGGAGAGCGC(G/A)GTTACCCTGGCAATATTGGTCCCGTTGGTG

[NM_000089.3](http://www.ncbi.nlm.nih.gov/entrez/viewer.fcgi?db=nucleotide&id=NM_000089.3): c.2864G>A; [NP_000080.2](http://www.ncbi.nlm.nih.gov/entrez/viewer.fcgi?db=nucleotide&id=NP_000080.2): p.G955D

agGGAGAGCGCGGTTACCCTGGCAATATTG(G/A)TCCCGTTGGTGCTGCAGGTGCACCTGGTCC

[NM_000089.3](http://www.ncbi.nlm.nih.gov/entrez/viewer.fcgi?db=nucleotide&id=NM_000089.3): c.2863G>T; [NP_000080.2](http://www.ncbi.nlm.nih.gov/entrez/viewer.fcgi?db=nucleotide&id=NP_000080.2): p.G955C

tagGGAGAGCGCGGTTACCCTGGCAATATT(G/T)GTCCCGTTGGTGCTGCAGGTGCACCTGGTC

[NM_000089.3](http://www.ncbi.nlm.nih.gov/entrez/viewer.fcgi?db=nucleotide&id=NM_000089.3): c.2863G>A; [NP_000080.2](http://www.ncbi.nlm.nih.gov/entrez/viewer.fcgi?db=nucleotide&id=NP_000080.2): p.G955S

tagGGAGAGCGCGGTTACCCTGGCAATATT(G/A)GTCCCGTTGGTGCTGCAGGTGCACCTGGTC

[NM_000089.3](http://www.ncbi.nlm.nih.gov/entrez/viewer.fcgi?db=nucleotide&id=NM_000089.3): c.2909G>A; [NP_000080.2](http://www.ncbi.nlm.nih.gov/entrez/viewer.fcgi?db=nucleotide&id=NP_000080.2): p.G970D

CAGGTGCACCTGGTCCTCATGGCCCCGTGG(G/A)TCCTGCTGGCAAACATGGAAACCGTGGTGA

[NM_000089.3](http://www.ncbi.nlm.nih.gov/entrez/viewer.fcgi?db=nucleotide&id=NM_000089.3): c.2918G>A; [NP_000080.2](http://www.ncbi.nlm.nih.gov/entrez/viewer.fcgi?db=nucleotide&id=NP_000080.2): p.G973D

CTGGTCCTCATGGCCCCGTGGGTCCTGCTG(G/A)CAAACATGGAAACCGTGGTGAAACTgtaag

[NM_000089.3](http://www.ncbi.nlm.nih.gov/entrez/viewer.fcgi?db=nucleotide&id=NM_000089.3): c.2918G>T; [NP_000080.2](http://www.ncbi.nlm.nih.gov/entrez/viewer.fcgi?db=nucleotide&id=NP_000080.2): p.G973V

CTGGTCCTCATGGCCCCGTGGGTCCTGCTG(G/T)CAAACATGGAAACCGTGGTGAAACTgtaag

[NM_000089.3](http://www.ncbi.nlm.nih.gov/entrez/viewer.fcgi?db=nucleotide&id=NM_000089.3): c.2945G>A; [NP_000080.2](http://www.ncbi.nlm.nih.gov/entrez/viewer.fcgi?db=nucleotide&id=NP_000080.2): p.G982D

aagttgtgtttttctttttcatttcacagG(G/A)TCCTTCTGGTCCTGTTGGTCCTGCTGGTGC

[NM_000089.3](http://www.ncbi.nlm.nih.gov/entrez/viewer.fcgi?db=nucleotide&id=NM_000089.3): c.2945G>T; [NP_000080.2](http://www.ncbi.nlm.nih.gov/entrez/viewer.fcgi?db=nucleotide&id=NP_000080.2): p.G982V

aagttgtgtttttctttttcatttcacagG(G/T)TCCTTCTGGTCCTGTTGGTCCTGCTGGTGC

[NM_000089.3](http://www.ncbi.nlm.nih.gov/entrez/viewer.fcgi?db=nucleotide&id=NM_000089.3): c.2963G>T; [NP_000080.2](http://www.ncbi.nlm.nih.gov/entrez/viewer.fcgi?db=nucleotide&id=NP_000080.2): p.G988V

tcatttcacagGGTCCTTCTGGTCCTGTTG(G/T)TCCTGCTGGTGCTGTTGGCCCAAGAGGTCC

[NM_000089.3](http://www.ncbi.nlm.nih.gov/entrez/viewer.fcgi?db=nucleotide&id=NM_000089.3): c.2972G>T; [NP_000080.2](http://www.ncbi.nlm.nih.gov/entrez/viewer.fcgi?db=nucleotide&id=NP_000080.2): p.G991V

agGGTCCTTCTGGTCCTGTTGGTCCTGCTG(G/T)TGCTGTTGGCCCAAGAGGTCCTAGTgtatg

[NM_000089.3](http://www.ncbi.nlm.nih.gov/entrez/viewer.fcgi?db=nucleotide&id=NM_000089.3): c.2981G>A; [NP_000080.2](http://www.ncbi.nlm.nih.gov/entrez/viewer.fcgi?db=nucleotide&id=NP_000080.2): p.G994D

CTGGTCCTGTTGGTCCTGCTGGTGCTGTTG(G/A)CCCAAGAGGTCCTAGTgtatgtacatgctg

[NM_000089.3](http://www.ncbi.nlm.nih.gov/entrez/viewer.fcgi?db=nucleotide&id=NM_000089.3): c.2990G>A; [NP_000080.2](http://www.ncbi.nlm.nih.gov/entrez/viewer.fcgi?db=nucleotide&id=NP_000080.2): p.G997D

TTGGTCCTGCTGGTGCTGTTGGCCCAAGAG(G/A)TCCTAGTgtatgtacatgctgaagatttct

[NM_000089.3](http://www.ncbi.nlm.nih.gov/entrez/viewer.fcgi?db=nucleotide&id=NM_000089.3): c.3008G>A; [NP_000080.2](http://www.ncbi.nlm.nih.gov/entrez/viewer.fcgi?db=nucleotide&id=NP_000080.2): p.G1003D

atccttctgtttctttatagGGCCCACAAG(G/A)CATTCGTGGCGATAAGGGAGAGCCCGGTGA

[NM_000089.3](http://www.ncbi.nlm.nih.gov/entrez/viewer.fcgi?db=nucleotide&id=NM_000089.3): c.3016G>A; [NP_000080.2](http://www.ncbi.nlm.nih.gov/entrez/viewer.fcgi?db=nucleotide&id=NP_000080.2): p.G1006S

gtttctttatagGGCCCACAAGGCATTCGT(G/A)GCGATAAGGGAGAGCCCGGTGAAAAGGGGC

[NM_000089.3](http://www.ncbi.nlm.nih.gov/entrez/viewer.fcgi?db=nucleotide&id=NM_000089.3): c.3034G>A; [NP_000080.2](http://www.ncbi.nlm.nih.gov/entrez/viewer.fcgi?db=nucleotide&id=NP_000080.2): p.G1012S

CAAGGCATTCGTGGCGATAAGGGAGAGCCC(G/A)GTGAAAAGGGGCCCAGAGGTCTTCCTGGCT

[NM_000089.3](http://www.ncbi.nlm.nih.gov/entrez/viewer.fcgi?db=nucleotide&id=NM_000089.3): c.3043G>A; [NP_000080.2](http://www.ncbi.nlm.nih.gov/entrez/viewer.fcgi?db=nucleotide&id=NP_000080.2): p.G1015R

CGTGGCGATAAGGGAGAGCCCGGTGAAAAG(G/A)GGCCCAGAGGTCTTCCTGGCTTAAAGGGAC

[NM_000089.3](http://www.ncbi.nlm.nih.gov/entrez/viewer.fcgi?db=nucleotide&id=NM_000089.3): c.3043G>C; [NP_000080.2](http://www.ncbi.nlm.nih.gov/entrez/viewer.fcgi?db=nucleotide&id=NP_000080.2): p.G1015R

CGTGGCGATAAGGGAGAGCCCGGTGAAAAG(G/C)GGCCCAGAGGTCTTCCTGGCTTAAAGGGAC

[NM_000089.3](http://www.ncbi.nlm.nih.gov/entrez/viewer.fcgi?db=nucleotide&id=NM_000089.3): c.3070G>C; [NP_000080.2](http://www.ncbi.nlm.nih.gov/entrez/viewer.fcgi?db=nucleotide&id=NP_000080.2): p.G1024R

AAGGGGCCCAGAGGTCTTCCTGGCTTAAAG(G/C)GACACAATGGATTGCAAGGTCTGCCTGGTA

[NM_000089.3](http://www.ncbi.nlm.nih.gov/entrez/viewer.fcgi?db=nucleotide&id=NM_000089.3): c.3079G>A; [NP_000080.2](http://www.ncbi.nlm.nih.gov/entrez/viewer.fcgi?db=nucleotide&id=NP_000080.2): p.G1027R

AGAGGTCTTCCTGGCTTAAAGGGACACAAT(G/A)GATTGCAAGGTCTGCCTGGTATCGCTgtaa

[NM_000089.3](http://www.ncbi.nlm.nih.gov/entrez/viewer.fcgi?db=nucleotide&id=NM_000089.3): c.3080G>A; [NP_000080.2](http://www.ncbi.nlm.nih.gov/entrez/viewer.fcgi?db=nucleotide&id=NP_000080.2): p.G1027E

GAGGTCTTCCTGGCTTAAAGGGACACAATG(G/A)ATTGCAAGGTCTGCCTGGTATCGCTgtaag

[NM_000089.3](http://www.ncbi.nlm.nih.gov/entrez/viewer.fcgi?db=nucleotide&id=NM_000089.3): c.3106G>C; [NP_000080.2](http://www.ncbi.nlm.nih.gov/entrez/viewer.fcgi?db=nucleotide&id=NP_000080.2): p.G1036R

aagtgtgattttcctcttctgtctttaaag(G/C)GTCACCATGGTGATCAAGGTGCTCCTGGCT

[NM_000089.3](http://www.ncbi.nlm.nih.gov/entrez/viewer.fcgi?db=nucleotide&id=NM_000089.3): c.3116G>A; [NP_000080.2](http://www.ncbi.nlm.nih.gov/entrez/viewer.fcgi?db=nucleotide&id=NP_000080.2): p.G1039D

ttcctcttctgtctttaaagGGTCACCATG(G/A)TGATCAAGGTGCTCCTGGCTCCGTGGGTCC

[NM_000089.3](http://www.ncbi.nlm.nih.gov/entrez/viewer.fcgi?db=nucleotide&id=NM_000089.3): c.3124G>A; [NP_000080.2](http://www.ncbi.nlm.nih.gov/entrez/viewer.fcgi?db=nucleotide&id=NP_000080.2): p.G1042S

ctgtctttaaagGGTCACCATGGTGATCAA(G/A)GTGCTCCTGGCTCCGTGGGTCCTGCTGGTC

[NM_000089.3](http://www.ncbi.nlm.nih.gov/entrez/viewer.fcgi?db=nucleotide&id=NM_000089.3): c.3151G>A; [NP_000080.2](http://www.ncbi.nlm.nih.gov/entrez/viewer.fcgi?db=nucleotide&id=NP_000080.2): p.G1051S

CAAGGTGCTCCTGGCTCCGTGGGTCCTGCT(G/A)GTCCTAGGgtaggtggactcaagagaagac

[NM_000089.3](http://www.ncbi.nlm.nih.gov/entrez/viewer.fcgi?db=nucleotide&id=NM_000089.3): c.3169G>T; [NP_000080.2](http://www.ncbi.nlm.nih.gov/entrez/viewer.fcgi?db=nucleotide&id=NP_000080.2): p.G1057C

cctggtgtctgtcttccttagGGCCCTGCT(G/T)GTCCTTCTGGCCCTGCTGGAAAAGATGGTC

[NM_000089.3](http://www.ncbi.nlm.nih.gov/entrez/viewer.fcgi?db=nucleotide&id=NM_000089.3): c.3197G>A; [NP_000080.2](http://www.ncbi.nlm.nih.gov/entrez/viewer.fcgi?db=nucleotide&id=NP_000080.2): p.G1066D

CTGGTCCTTCTGGCCCTGCTGGAAAAGATG(G/A)TCGCACTGGACATCCTGGTACAGTTGGACC

[NM_000089.3](http://www.ncbi.nlm.nih.gov/entrez/viewer.fcgi?db=nucleotide&id=NM_000089.3): c.3233G>A; [NP_000080.2](http://www.ncbi.nlm.nih.gov/entrez/viewer.fcgi?db=nucleotide&id=NP_000080.2): p.G1078D

CTGGACATCCTGGTACAGTTGGACCTGCTG(G/A)CATTCGAGGCCCTCAGGGTCACCAAGGCCC

[NM_000089.3](http://www.ncbi.nlm.nih.gov/entrez/viewer.fcgi?db=nucleotide&id=NM_000089.3): c.3242G>T; [NP_000080.2](http://www.ncbi.nlm.nih.gov/entrez/viewer.fcgi?db=nucleotide&id=NP_000080.2): p.G1081V

CTGGTACAGTTGGACCTGCTGGCATTCGAG(G/T)CCCTCAGGGTCACCAAGGCCCTGCTgtaag

[NM_000089.3](http://www.ncbi.nlm.nih.gov/entrez/viewer.fcgi?db=nucleotide&id=NM_000089.3): c.3251G>T; [NP_000080.2](http://www.ncbi.nlm.nih.gov/entrez/viewer.fcgi?db=nucleotide&id=NP_000080.2): p.G1084V

TTGGACCTGCTGGCATTCGAGGCCCTCAGG(G/T)TCACCAAGGCCCTGCTgtaagtatgatttg

[NM_000089.3](http://www.ncbi.nlm.nih.gov/entrez/viewer.fcgi?db=nucleotide&id=NM_000089.3): c.3260G>A; [NP_000080.2](http://www.ncbi.nlm.nih.gov/entrez/viewer.fcgi?db=nucleotide&id=NP_000080.2): p.G1087D

CTGGCATTCGAGGCCCTCAGGGTCACCAAG(G/A)CCCTGCTgtaagtatgatttggggaaataa

[NM_000089.3](http://www.ncbi.nlm.nih.gov/entrez/viewer.fcgi?db=nucleotide&id=NM_000089.3): c.3269G>A; [NP_000080.2](http://www.ncbi.nlm.nih.gov/entrez/viewer.fcgi?db=nucleotide&id=NP_000080.2): p.G1090D

cctctcctatctcactttcacctttgcagG(G/A)CCCCCCTGGTCCCCCTGGCCCTCCTGGACC

[NM_000089.3](http://www.ncbi.nlm.nih.gov/entrez/viewer.fcgi?db=nucleotide&id=NM_000089.3): c.3268G>T; [NP_000080.2](http://www.ncbi.nlm.nih.gov/entrez/viewer.fcgi?db=nucleotide&id=NP_000080.2): p.G1090C

gcctctcctatctcactttcacctttgcag(G/T)GCCCCCCTGGTCCCCCTGGCCCTCCTGGAC

[NM_000089.3](http://www.ncbi.nlm.nih.gov/entrez/viewer.fcgi?db=nucleotide&id=NM_000089.3): c.3287G>C; [NP_000080.2](http://www.ncbi.nlm.nih.gov/entrez/viewer.fcgi?db=nucleotide&id=NP_000080.2): p.G1096A

cacctttgcagGGCCCCCCTGGTCCCCCTG(G/C)CCCTCCTGGACCTCCAGGTGTAAGCGGTGG

[NM_000089.3](http://www.ncbi.nlm.nih.gov/entrez/viewer.fcgi?db=nucleotide&id=NM_000089.3): c.3295G>A; [NP_000080.2](http://www.ncbi.nlm.nih.gov/entrez/viewer.fcgi?db=nucleotide&id=NP_000080.2): p.G1099R

cagGGCCCCCCTGGTCCCCCTGGCCCTCCT(G/A)GACCTCCAGGTGTAAGCGGTGGTGGTTATG

[NM_000089.3](http://www.ncbi.nlm.nih.gov/entrez/viewer.fcgi?db=nucleotide&id=NM_000089.3): c.3305G>C; [NP_000080.2](http://www.ncbi.nlm.nih.gov/entrez/viewer.fcgi?db=nucleotide&id=NP_000080.2): p.G1102A

CTGGTCCCCCTGGCCCTCCTGGACCTCCAG(G/C)TGTAAGCGGTGGTGGTTATGACTTTGGTTA

[NM_000089.3](http://www.ncbi.nlm.nih.gov/entrez/viewer.fcgi?db=nucleotide&id=NM_000089.3): c.3304G>C; [NP_000080.2](http://www.ncbi.nlm.nih.gov/entrez/viewer.fcgi?db=nucleotide&id=NP_000080.2): p.G1102R

CCTGGTCCCCCTGGCCCTCCTGGACCTCCA(G/C)GTGTAAGCGGTGGTGGTTATGACTTTGGTT

[NM_000089.3](http://www.ncbi.nlm.nih.gov/entrez/viewer.fcgi?db=nucleotide&id=NM_000089.3): c.3305G>A; [NP_000080.2](http://www.ncbi.nlm.nih.gov/entrez/viewer.fcgi?db=nucleotide&id=NP_000080.2): p.G1102D

CTGGTCCCCCTGGCCCTCCTGGACCTCCAG(G/A)TGTAAGCGGTGGTGGTTATGACTTTGGTTA

[NM_000089.3](http://www.ncbi.nlm.nih.gov/entrez/viewer.fcgi?db=nucleotide&id=NM_000089.3): c.3304G>A; [NP_000080.2](http://www.ncbi.nlm.nih.gov/entrez/viewer.fcgi?db=nucleotide&id=NP_000080.2): p.G1102S

CCTGGTCCCCCTGGCCCTCCTGGACCTCCA(G/A)GTGTAAGCGGTGGTGGTTATGACTTTGGTT

[NM_000089.3](http://www.ncbi.nlm.nih.gov/entrez/viewer.fcgi?db=nucleotide&id=NM_000089.3): c.3305G>T; [NP_000080.2](http://www.ncbi.nlm.nih.gov/entrez/viewer.fcgi?db=nucleotide&id=NP_000080.2): p.G1102V

CTGGTCCCCCTGGCCCTCCTGGACCTCCAG(G/T)TGTAAGCGGTGGTGGTTATGACTTTGGTTA

[NM_000089.3](http://www.ncbi.nlm.nih.gov/entrez/viewer.fcgi?db=nucleotide&id=NM_000089.3): c.3313G>A; [NP_000080.2](http://www.ncbi.nlm.nih.gov/entrez/viewer.fcgi?db=nucleotide&id=NP_000080.2): p.G1105S

CCTGGCCCTCCTGGACCTCCAGGTGTAAGC(G/A)GTGGTGGTTATGACTTTGGTTACGATGGAG

[NM_000089.3](http://www.ncbi.nlm.nih.gov/entrez/viewer.fcgi?db=nucleotide&id=NM_000089.3): c.3332G>A; [NP_000080.2](http://www.ncbi.nlm.nih.gov/entrez/viewer.fcgi?db=nucleotide&id=NP_000080.2): p.G1111D

CAGGTGTAAGCGGTGGTGGTTATGACTTTG(G/A)TTACGATGGAGACTTCTACAGGGCTGACCA

[NM_000089.3](http://www.ncbi.nlm.nih.gov/entrez/viewer.fcgi?db=nucleotide&id=NM_000089.3): c.3350A>G; [NP_000080.2](http://www.ncbi.nlm.nih.gov/entrez/viewer.fcgi?db=nucleotide&id=NP_000080.2): p.Y1117C

GTTATGACTTTGGTTACGATGGAGACTTCT(A/G)CAGGGCTGACCAGCCTCGCTCAGCACCTTC

[NM_000089.3](http://www.ncbi.nlm.nih.gov/entrez/viewer.fcgi?db=nucleotide&id=NM_000089.3): c.3355G>A; [NP_000080.2](http://www.ncbi.nlm.nih.gov/entrez/viewer.fcgi?db=nucleotide&id=NP_000080.2): p.A1119T

GACTTTGGTTACGATGGAGACTTCTACAGG(G/A)CTGACCAGCCTCGCTCAGCACCTTCTCTCA

[NM_000089.3](http://www.ncbi.nlm.nih.gov/entrez/viewer.fcgi?db=nucleotide&id=NM_000089.3): c.3359A>C; [NP_000080.2](http://www.ncbi.nlm.nih.gov/entrez/viewer.fcgi?db=nucleotide&id=NP_000080.2): p.D1120A

TTGGTTACGATGGAGACTTCTACAGGGCTG(A/C)CCAGCCTCGCTCAGCACCTTCTCTCAGACC

[NM_000089.3](http://www.ncbi.nlm.nih.gov/entrez/viewer.fcgi?db=nucleotide&id=NM_000089.3): c.3442A>C; [NP_000080.2](http://www.ncbi.nlm.nih.gov/entrez/viewer.fcgi?db=nucleotide&id=NP_000080.2): p.T1148P

ACTCTGAAGTCTCTCAACAACCAGATTGAG(A/C)CCCTTCTTACTCCTGAAGGCTCTAGAAAGA

[NM_000089.3](http://www.ncbi.nlm.nih.gov/entrez/viewer.fcgi?db=nucleotide&id=NM_000089.3): c.3584G>A; [NP_000080.2](http://www.ncbi.nlm.nih.gov/entrez/viewer.fcgi?db=nucleotide&id=NP_000080.2): p.C1195Y

GATGCACTATGGATGCTATCAAAGTATACT(G/A)TGATTTCTCTACTGGCGAAACCTGTATCCG

[NM_000089.3](http://www.ncbi.nlm.nih.gov/entrez/viewer.fcgi?db=nucleotide&id=NM_000089.3): c.3601G>T; [NP_000080.2](http://www.ncbi.nlm.nih.gov/entrez/viewer.fcgi?db=nucleotide&id=NP_000080.2): p.E1201X

ATCAAAGTATACTGTGATTTCTCTACTGGC(G/T)AAACCTGTATCCGGGCCCAACCTGAAAACA

[NM_000089.3](http://www.ncbi.nlm.nih.gov/entrez/viewer.fcgi?db=nucleotide&id=NM_000089.3): c.4095C>A; [NP_000080.2](http://www.ncbi.nlm.nih.gov/entrez/viewer.fcgi?db=nucleotide&id=NP_000080.2): p.F1365L

ATTCTTTGTGGACATTGGCCCAGTCTGTTT(C/A)AAATAAatgaactcaatctaaattaaaaaa

[NM_000089.3](http://www.ncbi.nlm.nih.gov/entrez/viewer.fcgi?db=nucleotide&id=NM_000089.3): c.4099T>C; [NP_000080.2](http://www.ncbi.nlm.nih.gov/entrez/viewer.fcgi?db=nucleotide&id=NP_000080.2): p.X1367Q

TTTGTGGACATTGGCCCAGTCTGTTTCAAA(T/C)AAatgaactcaatctaaattaaaaaagaaa

**Splicing**

[NM_000089.3](http://www.ncbi.nlm.nih.gov/entrez/viewer.fcgi?db=nucleotide&id=NM_000089.3): c.70+717A>G

acttggagtgggtacattctgaaaagtaat(a/g)taagtgtctcaattcactttctagtcatgg

[NM_000089.3](http://www.ncbi.nlm.nih.gov/entrez/viewer.fcgi?db=nucleotide&id=NM_000089.3): c.226-2A>G

atatatataattttttttttttacttctct(a/g)gAACTTTGCTGCTCAGTATGATGGAAAAGG

[NM_000089.3](http://www.ncbi.nlm.nih.gov/entrez/viewer.fcgi?db=nucleotide&id=NM_000089.3): c.226-1G>A

tatatataattttttttttttacttctcta(g/a)AACTTTGCTGCTCAGTATGATGGAAAAGGA

[NM_000089.3](http://www.ncbi.nlm.nih.gov/entrez/viewer.fcgi?db=nucleotide&id=NM_000089.3): c.226-1G>C

tatatataattttttttttttacttctcta(g/c)AACTTTGCTGCTCAGTATGATGGAAAAGGA

[NM_000089.3](http://www.ncbi.nlm.nih.gov/entrez/viewer.fcgi?db=nucleotide&id=NM_000089.3): c.279G>A

AAAAGGAGTTGGACTTGGCCCTGGACCAAT(G/A)gtatgcttatctgtttatcttagccaaaaa

[NM_000089.3](http://www.ncbi.nlm.nih.gov/entrez/viewer.fcgi?db=nucleotide&id=NM_000089.3): c.279+1G>A

AAAGGAGTTGGACTTGGCCCTGGACCAATG(g/a)tatgcttatctgtttatcttagccaaaaaa

[NM_000089.3](http://www.ncbi.nlm.nih.gov/entrez/viewer.fcgi?db=nucleotide&id=NM_000089.3): c.279+1G>T

AAAGGAGTTGGACTTGGCCCTGGACCAATG(g/t)tatgcttatctgtttatcttagccaaaaaa

[NM_000089.3](http://www.ncbi.nlm.nih.gov/entrez/viewer.fcgi?db=nucleotide&id=NM_000089.3): c.279+2T>C

AAGGAGTTGGACTTGGCCCTGGACCAATGg(t/c)atgcttatctgtttatcttagccaaaaaaa

[NM_000089.3](http://www.ncbi.nlm.nih.gov/entrez/viewer.fcgi?db=nucleotide&id=NM_000089.3): c.433-1G>C

gaagttgactctacaatgttttcatgttta(g/c)GGTCACCCTGGAAAACCCGGACGACCTGGT

[NM_000089.3](http://www.ncbi.nlm.nih.gov/entrez/viewer.fcgi?db=nucleotide&id=NM_000089.3): c.432+1G>T

GCTGGCCCTCCTGGCAAGGCTGGTGAAGAT(g/t)taagtatttactcttaagcactttcaaaat

[NM_000089.3](http://www.ncbi.nlm.nih.gov/entrez/viewer.fcgi?db=nucleotide&id=NM_000089.3): c.432+5G>A

GCCCTCCTGGCAAGGCTGGTGAAGATgtaa(g/a)tatttactcttaagcactttcaaaatgcta

[NM_000089.3](http://www.ncbi.nlm.nih.gov/entrez/viewer.fcgi?db=nucleotide&id=NM_000089.3): c.486+1G>C

CCTGGTGAGAGAGGAGTTGTTGGACCACAG(g/c)tgagactttttacattggtagatagcacaa

[NM_000089.3](http://www.ncbi.nlm.nih.gov/entrez/viewer.fcgi?db=nucleotide&id=NM_000089.3): c.540+5G>A

GACTTCCTGGCTTCAAAGGCATTAGGgtga(g/a)cacattctttactcagaagagagaaaatgc

[NM_000089.3](http://www.ncbi.nlm.nih.gov/entrez/viewer.fcgi?db=nucleotide&id=NM_000089.3): c.595-3T>A

taatgaacaaaaactcaatccttctccatg(t/a)agGGTGAACCTGGTGCCCCTGGTGAAAATG

[NM_000089.3](http://www.ncbi.nlm.nih.gov/entrez/viewer.fcgi?db=nucleotide&id=NM_000089.3): c.595-2A>G

aatgaacaaaaactcaatccttctccatgt(a/g)gGGTGAACCTGGTGCCCCTGGTGAAAATGG

[NM_000089.3](http://www.ncbi.nlm.nih.gov/entrez/viewer.fcgi?db=nucleotide&id=NM_000089.3): c.594+2T>G

AGGGACAGCCCGGTGCTCCTGGTGTGAAGg(t/g)aaatattaaattagaagcactgtttttaag

[NM_000089.3](http://www.ncbi.nlm.nih.gov/entrez/viewer.fcgi?db=nucleotide&id=NM_000089.3): c.693+1G>A

AGAGGACGTGTTGGTGCCCCTGGCCCAGCT(g/a)taagtgcttccatttttgttcagtttcatc

[NM_000089.3](http://www.ncbi.nlm.nih.gov/entrez/viewer.fcgi?db=nucleotide&id=NM_000089.3): c.693+2T>A

GAGGACGTGTTGGTGCCCCTGGCCCAGCTg(t/a)aagtgcttccatttttgttcagtttcatcc

[NM_000089.3](http://www.ncbi.nlm.nih.gov/entrez/viewer.fcgi?db=nucleotide&id=NM_000089.3): c.693+2T>C

GAGGACGTGTTGGTGCCCCTGGCCCAGCTg(t/c)aagtgcttccatttttgttcagtttcatcc

[NM_000089.3](http://www.ncbi.nlm.nih.gov/entrez/viewer.fcgi?db=nucleotide&id=NM_000089.3): c.739-2A>G

aaatttcttaccaccttctgctttgatttc(a/g)gGGTCCCATTGGGTCTGCTGGCCCTCCAGG

[NM_000089.3](http://www.ncbi.nlm.nih.gov/entrez/viewer.fcgi?db=nucleotide&id=NM_000089.3): c.792+1G>A

CCAGGCTTCCCAGGTGCCCCTGGCCCCAAG(g/a)taaaaacactggtgaccattgtcactactt

[NM_000089.3](http://www.ncbi.nlm.nih.gov/entrez/viewer.fcgi?db=nucleotide&id=NM_000089.3): c.792+1G>T

CCAGGCTTCCCAGGTGCCCCTGGCCCCAAG(g/t)taaaaacactggtgaccattgtcactactt

[NM_000089.3](http://www.ncbi.nlm.nih.gov/entrez/viewer.fcgi?db=nucleotide&id=NM_000089.3): c.792+2T>A

CAGGCTTCCCAGGTGCCCCTGGCCCCAAGg(t/a)aaaaacactggtgaccattgtcactacttt

[NM_000089.3](http://www.ncbi.nlm.nih.gov/entrez/viewer.fcgi?db=nucleotide&id=NM_000089.3): c.792+2T>C

CAGGCTTCCCAGGTGCCCCTGGCCCCAAGg(t/c)aaaaacactggtgaccattgtcactacttt

[NM_000089.3](http://www.ncbi.nlm.nih.gov/entrez/viewer.fcgi?db=nucleotide&id=NM_000089.3): c.1036-1G>C

cctttgaccactgttctgtattgaacccta(g/c)GGTGAGCCTGGTCCAGCTGGCTCCAAAGGA

[NM_000089.3](http://www.ncbi.nlm.nih.gov/entrez/viewer.fcgi?db=nucleotide&id=NM_000089.3): c.1197+5G>A

GCCCTCCAGGACCTCCTGGGCTGAGAgtag(g/a)tttcaaatgctcccaacaccctaacacacc

[NM_000089.3](http://www.ncbi.nlm.nih.gov/entrez/viewer.fcgi?db=nucleotide&id=NM_000089.3): c.1197+5G>C

GCCCTCCAGGACCTCCTGGGCTGAGAgtag(g/c)tttcaaatgctcccaacaccctaacacacc

[NM_000089.3](http://www.ncbi.nlm.nih.gov/entrez/viewer.fcgi?db=nucleotide&id=NM_000089.3): c.1404+1G>A

ATCGGCCCCGCTGGAAAAGAAGGTCCTGTC(g/a)taagtattgctcattttccattatattttc

[NM_000089.3](http://www.ncbi.nlm.nih.gov/entrez/viewer.fcgi?db=nucleotide&id=NM_000089.3): c.1404+1G>C

ATCGGCCCCGCTGGAAAAGAAGGTCCTGTC(g/c)taagtattgctcattttccattatattttc

[NM_000089.3](http://www.ncbi.nlm.nih.gov/entrez/viewer.fcgi?db=nucleotide&id=NM_000089.3): c.1504-1G>C

ttctttgttttgtttttcatttttactcta(g/c)GGTGATCCTGGCAAAAACGGTGATAAAGGT

[NM_000089.3](http://www.ncbi.nlm.nih.gov/entrez/viewer.fcgi?db=nucleotide&id=NM_000089.3): c.1557+3A>G

AGGTCATGCTGGTCTTGCTGGTGCTCGGgt(a/g)ggtgctaacttgtgtacagatctattcaca

[NM_000089.3](http://www.ncbi.nlm.nih.gov/entrez/viewer.fcgi?db=nucleotide&id=NM_000089.3): c.1612-2A>G

gctatttatgctctctttcctgtcactttc(a/g)gGGTGTTCAAGGTGGAAAAGGTGAACAGGG

[NM_000089.3](http://www.ncbi.nlm.nih.gov/entrez/viewer.fcgi?db=nucleotide&id=NM_000089.3): c.1720-2A>G

gatttcatgctttattctcatgttttgtct(a/g)gGGTCTCCATGGTGAGTTTGGTCTCCCTGG

[NM_000089.3](http://www.ncbi.nlm.nih.gov/entrez/viewer.fcgi?db=nucleotide&id=NM_000089.3): c.1764+1G>A

TTTGGTCTCCCTGGTCCTGCTGGTCCAAGA(g/a)taagtgttacttcattaactttcataaact

[NM_000089.3](http://www.ncbi.nlm.nih.gov/entrez/viewer.fcgi?db=nucleotide&id=NM_000089.3): c.1971+1G>A

GCTGGCATACCTGGAGGCAAGGGAGAAAAG(g/a)tacgtgttgacccctattacatattgttga

[NM_000089.3](http://www.ncbi.nlm.nih.gov/entrez/viewer.fcgi?db=nucleotide&id=NM_000089.3): c.2025+4A>G

GGTAACCCTGGCAGAGATGGTGCTCGTgtg(a/g)gtagaattttgtttgtatgtttcttcgtac

[NM_000089.3](http://www.ncbi.nlm.nih.gov/entrez/viewer.fcgi?db=nucleotide&id=NM_000089.3): c.2025+5G>A

GTAACCCTGGCAGAGATGGTGCTCGTgtga(g/a)tagaattttgtttgtatgtttcttcgtact

[NM_000089.3](http://www.ncbi.nlm.nih.gov/entrez/viewer.fcgi?db=nucleotide&id=NM_000089.3): c.2133+5G>A

GTCCTGCTGGTCCTCGGGGAAGCCCTgtaa(g/a)taagaacctgggtcattttgtatactcaca

[NM_000089.3](http://www.ncbi.nlm.nih.gov/entrez/viewer.fcgi?db=nucleotide&id=NM_000089.3): c.2133+6T>A

TCCTGCTGGTCCTCGGGGAAGCCCTgtaag(t/a)aagaacctgggtcattttgtatactcacac

[NM_000089.3](http://www.ncbi.nlm.nih.gov/entrez/viewer.fcgi?db=nucleotide&id=NM_000089.3): c.2187+2T>C

CTGGCCCCAATGGATTTGCTGGTCCTGCTg(t/c)gagtatcacataatgaagattaatctgaaa

[NM_000089.3](http://www.ncbi.nlm.nih.gov/entrez/viewer.fcgi?db=nucleotide&id=NM_000089.3): c.2296-2A>G

tgatttgactccatctttttgtttgcattt(a/g)gGGTCCAAATGGTCCCCCCGGTCCTGCTGG

[NM_000089.3](http://www.ncbi.nlm.nih.gov/entrez/viewer.fcgi?db=nucleotide&id=NM_000089.3): c.2295+1G>T

ACAGGCCCCGTTGGAGCTGCTGGCCCAGCT(g/t)taagttgaattcactggtggtccacacagc

[NM_000089.3](http://www.ncbi.nlm.nih.gov/entrez/viewer.fcgi?db=nucleotide&id=NM_000089.3): c.2565+1G>A

AAGGGTCCCTCTGGAGAGGCTGGTACTGCT(g/a)taagtgatttccaactcctctttcttaata

[NM_000089.3](http://www.ncbi.nlm.nih.gov/entrez/viewer.fcgi?db=nucleotide&id=NM_000089.3): c.2565+5G>A

GTCCCTCTGGAGAGGCTGGTACTGCTgtaa(g/a)tgatttccaactcctctttcttaatacctt

[NM_000089.3](http://www.ncbi.nlm.nih.gov/entrez/viewer.fcgi?db=nucleotide&id=NM_000089.3): c.2674-3T>G

gggaatgtcattttatcttctctgcctgtt(t/g)agGGTGAACCTGGTCCTCTTGGCATTGCCG

[NM_000089.3](http://www.ncbi.nlm.nih.gov/entrez/viewer.fcgi?db=nucleotide&id=NM_000089.3): c.2673+1G>A

CGTGGTCTACCAGGTGTTGCTGGTGCTGTG(g/a)tgagtgcttgacagtattctgactccatta

[NM_000089.3](http://www.ncbi.nlm.nih.gov/entrez/viewer.fcgi?db=nucleotide&id=NM_000089.3): c.2835+1G>A

CCAGGTCGCGATGGTCAACCCGGACACAAG(g/a)tcagtacacttttcatctttctctaattca

[NM_000089.3](http://www.ncbi.nlm.nih.gov/entrez/viewer.fcgi?db=nucleotide&id=NM_000089.3): c.2944-2A>G

gctaagttgtgtttttctttttcatttcac(a/g)gGGTCCTTCTGGTCCTGTTGGTCCTGCTGG

[NM_000089.3](http://www.ncbi.nlm.nih.gov/entrez/viewer.fcgi?db=nucleotide&id=NM_000089.3): c.3105+2T>C

ATGGATTGCAAGGTCTGCCTGGTATCGCTg(t/c)aagtaaactgtagccatctcgcacataaac

[NM_000089.3](http://www.ncbi.nlm.nih.gov/entrez/viewer.fcgi?db=nucleotide&id=NM_000089.3): c.3159+5G>C

GCTCCGTGGGTCCTGCTGGTCCTAGGgtag(g/c)tggactcaagagaagacagttcatctctga

**Small deletions**

[NM_000089.3](http://www.ncbi.nlm.nih.gov/entrez/viewer.fcgi?db=nucleotide&id=NM_000089.3): c.432+3_432+13delAAGTATTTACT

TGGCCCTCCTGGCAAGGCTGGTGAAGATgt(aagtatttact/-)cttaagcactttcaaaatgctatttaaata

[NM_000089.3](http://www.ncbi.nlm.nih.gov/entrez/viewer.fcgi?db=nucleotide&id=NM_000089.3): c.432+4_432+7delAGTA

GGCCCTCCTGGCAAGGCTGGTGAAGATgta(agta/-)tttactcttaagcactttcaaaatgctatt

[NM_000089.3](http://www.ncbi.nlm.nih.gov/entrez/viewer.fcgi?db=nucleotide&id=NM_000089.3): c.487-4_501delTAAGGGTGCTCGTGGTTTC

acattattcaccatcttctgtatttctttc(taagGGTGCTCGTGGTTTC/-)CCTGGAACTCCTGGACTTCCTGGCTTCAAA

[NM_000089.3](http://www.ncbi.nlm.nih.gov/entrez/viewer.fcgi?db=nucleotide&id=NM_000089.3): c.594+1_594+19delGTAAATATTAAATTAGAAG

AAGGGACAGCCCGGTGCTCCTGGTGTGAAG(gtaaatattaaattagaag/-)cactgtttttaagcacttgattgaaattcc

[NM_000089.3](http://www.ncbi.nlm.nih.gov/entrez/viewer.fcgi?db=nucleotide&id=NM_000089.3): c.639+4_639+22delAGTATTGACTACTTCATTG

GGTGAAAATGGAACTCCAGGTCAAACAgta(agtattgactacttcattg/-)taaatttaaatgtgtacactctttatgaga

[NM_000089.3](http://www.ncbi.nlm.nih.gov/entrez/viewer.fcgi?db=nucleotide&id=NM_000089.3): c.1035_1035+2delTGT

TGCCGGTGCTACTGGTGCCAGAGGACTTGT(Tgt/-)aagtggtcatgactgtggttctcatcatcc

[NM_000089.3](http://www.ncbi.nlm.nih.gov/entrez/viewer.fcgi?db=nucleotide&id=NM_000089.3): c.1035+1_1035+2delGT

GCCGGTGCTACTGGTGCCAGAGGACTTGTT(gt/-)aagtggtcatgactgtggttctcatcatcc

[NM_000089.3](http://www.ncbi.nlm.nih.gov/entrez/viewer.fcgi?db=nucleotide&id=NM_000089.3): c.1380_1397delCCCCGCTGGAAAAGAAGG

gGGTCTTCCTGGTTCCCCTGGAAATATCGG(CCCCGCTGGAAAAGAAGG/-)TCCTGTCgtaagtattgctcattttccatt

[NM_000089.3](http://www.ncbi.nlm.nih.gov/entrez/viewer.fcgi?db=nucleotide&id=NM_000089.3): c.2113_2121delGCTGGTCCT

GAAGCTGGGGCTGCTGGTCCTGCTGGTCCT(GCTGGTCCT/-)CGGGGAAGCCCTgtaagtaagaacctgggt

[NM_000089.3](http://www.ncbi.nlm.nih.gov/entrez/viewer.fcgi?db=nucleotide&id=NM_000089.3): c.2415_2432delCCCTCCTGGTCCCCCTGG

cttgtacatttgctcatagGGTATTTCTGG(CCCTCCTGGTCCCCCTGG/-)TCCTGCTGGGAAAGAAGGGCTTCGTGGTCC

[NM_000089.3](http://www.ncbi.nlm.nih.gov/entrez/viewer.fcgi?db=nucleotide&id=NM_000089.3): c.2712_2729delGGCCCGTGGTCCTCCTGG

TGGTCCTCTTGGCATTGCCGGCCCTCCTGG(GGCCCGTGGTCCTCCTGG/-)TGCTGTGGGTAGTCCTGGAGTCAACGGTGC

[NM_000089.3](http://www.ncbi.nlm.nih.gov/entrez/viewer.fcgi?db=nucleotide&id=NM_000089.3): c.3171_3188delTCCTTCTGGCCCTGCTGG

tggtgtctgtcttccttagGGCCCTGCTGG(TCCTTCTGGCCCTGCTGG/-)AAAAGATGGTCGCACTGGACATCCTGGTAC

[NM_000089.3](http://www.ncbi.nlm.nih.gov/entrez/viewer.fcgi?db=nucleotide&id=NM_000089.3): c.3279_3287delTCCCCCTGG

ctcactttcacctttgcagGGCCCCCCTGG(TCCCCCTGG/-)CCCTCCTGGACCTCCAGGTGTAAGCGGTGG

[NM_000089.3](http://www.ncbi.nlm.nih.gov/entrez/viewer.fcgi?db=nucleotide&id=NM_000089.3): c.3294_3302delTGGACCTCC

gcagGGCCCCCCTGGTCCCCCTGGCCCTCC(TGGACCTCC/-)AGGTGTAAGCGGTGGTGGTTATGACTTTGG

[NM_000089.3](http://www.ncbi.nlm.nih.gov/entrez/viewer.fcgi?db=nucleotide&id=NM_000089.3): c.4001_4004delATAA

GGGGAAAGACAATCATTGAATACAAAACAA(ATAA/-)GCCATCACGCCTGCCCTTCCTTGATATTGC

**Small insertions**

[NM_000089.3](http://www.ncbi.nlm.nih.gov/entrez/viewer.fcgi?db=nucleotide&id=NM_000089.3): c.293dupC

tctgctttctttacagGGCTTAATGGGACC(-/c)TAGAGGCCCACCTGGTGCAGCTGGAGCCCC

[NM_000089.3](http://www.ncbi.nlm.nih.gov/entrez/viewer.fcgi?db=nucleotide&id=NM_000089.3): c.2048_2049insTCATGGTGCTGTAGGTGC

gtactagGGTGCTCCTGGTGCTGTAGGTGC(-/tcatggtgctgtaggtgc)CCCTGGTCCTGCTGGAGCCACAGGTGACCG

[NM_000089.3](http://www.ncbi.nlm.nih.gov/entrez/viewer.fcgi?db=nucleotide&id=NM_000089.3): c.2391_2393dupCCC

CCCTGGTGCTGCTGGACGGACTGGTCCCCC(-/ccc)AGGACCCTCTgtaagtaaatcactgtaaac

[NM_000089.3](http://www.ncbi.nlm.nih.gov/entrez/viewer.fcgi?db=nucleotide&id=NM_000089.3): c.2427_2435dupCCCTGGTCC

TATTTCTGGCCCTCCTGGTCCCCCTGGTCC(-/ccctggtcc)TGCTGGGAAAGAAGGGCTTCGTGGTCCTCG

[NM_000089.3](http://www.ncbi.nlm.nih.gov/entrez/viewer.fcgi?db=nucleotide&id=NM_000089.3): c.2960_2968dupTTGGTCCTG

cacagGGTCCTTCTGGTCCTGTTGGTCCTG(-/ttggtcctg)CTGGTGCTGTTGGCCCAAGAGGTCCTAGTg

[NM_000089.3](http://www.ncbi.nlm.nih.gov/entrez/viewer.fcgi?db=nucleotide&id=NM_000089.3): c.2964_2981dupTCCTGCTGGTGCTGTTGG

TGGTCCTGTTGGTCCTGCTGGTGCTGTTGG(-/tcctgctggtgctgttgg)CCCAAGAGGTCCTAGTgtatgtacatgctg

[NM_000089.3](http://www.ncbi.nlm.nih.gov/entrez/viewer.fcgi?db=nucleotide&id=NM_000089.3): c.2967_2984dupTGCTGGTGCTGTTGGCCC

TCCTGTTGGTCCTGCTGGTGCTGTTGGCCC(-/tgctggtgctgttggccc)AAGAGGTCCTAGTgtatgtacatgctgaag

**Small indels**

[NM_000089.3](http://www.ncbi.nlm.nih.gov/entrez/viewer.fcgi?db=nucleotide&id=NM_000089.3): c.1009_1010delGGinsCC

CGCGGTATTCCTGGCCCTGTTGGTGCTGCC(GG/cc)TGCTACTGGTGCCAGAGGACTTGTTgtaag

[NM_000089.3](http://www.ncbi.nlm.nih.gov/entrez/viewer.fcgi?db=nucleotide&id=NM_000089.3): c.1226_1227delGAinsTT

agGGTAGTCCTGGTTCTCGTGGTCTTCCTG(GA/tt)GCTGATGGCAGAGCTGGCGTCATGgtaagc

[NM_000089.3](http://www.ncbi.nlm.nih.gov/entrez/viewer.fcgi?db=nucleotide&id=NM_000089.3): c.1369_1370delGGinsCT

tttattttatagGGTCTTCCTGGTTCCCCT(GG/ct)AAATATCGGCCCCGCTGGAAAAGAAGGTCC

[NM_000089.3](http://www.ncbi.nlm.nih.gov/entrez/viewer.fcgi?db=nucleotide&id=NM_000089.3): c.2542_2543delCCinsTT

GGTCCCCCTGGCTTCGCTGGTGAGAAGGGT(CC/tt)CTCTGGAGAGGCTGGTACTGCTgtaagtga

GGTCCCCCTGGCTTCGCTGGTGAGAAGGGT(CC/tt)CTCTGGAGAGGCTGGTACTGCTgtaagtga

[NM_000089.3](http://www.ncbi.nlm.nih.gov/entrez/viewer.fcgi?db=nucleotide&id=NM_000089.3): c.3115_3116delGGinsTT

tttcctcttctgtctttaaagGGTCACCAT(GG/tt)TGATCAAGGTGCTCCTGGCTCCGTGGGTCC
