# Supplementary material for: Development of a High-Throughput Resequencing Array for the Detection of Pathogenic Mutations in Osteogenesis Imperfecta
Source: PLoS One. 2015 Mar 5;10(3):e0119553. doi: 10.1371/journal.pone.0119553 (PMC4350936; doi:10.1371/journal.pone.0119553)
Supplement: S3 Datasets — According to the selected mutations, we have made them into the probes to tiles on the chip. (ZIP) [file pone.0119553.s003.zip › CRTAP.doc]

**Missense/nonsense**

[NM_006371.4](http://www.ncbi.nlm.nih.gov/entrez/viewer.fcgi?db=nucleotide&id=NM_006371.4): c.3G>A; [NP_006362.1](http://www.ncbi.nlm.nih.gov/entrez/viewer.fcgi?db=nucleotide&id=NP_006362.1): p.M1I

ccttccttccttcctttcgccgggcgcgAT(G/A)GAGCCGGGGCGCCGGGGGGCCGCGGCGCTG

[NM_006371.4](http://www.ncbi.nlm.nih.gov/entrez/viewer.fcgi?db=nucleotide&id=NM_006371.4): c.38C>A; [NP_006362.1](http://www.ncbi.nlm.nih.gov/entrez/viewer.fcgi?db=nucleotide&id=NP_006362.1): p.A13E

CGGGGCGCCGGGGGGCCGCGGCGCTGCTAG(C/A)GCTGCTGTGCGTGGCCTGCGCGCTGCGCGC

[NM_006371.4](http://www.ncbi.nlm.nih.gov/entrez/viewer.fcgi?db=nucleotide&id=NM_006371.4): c.198C>A; [NP_006362.1](http://www.ncbi.nlm.nih.gov/entrez/viewer.fcgi?db=nucleotide&id=NP_006362.1): p.Y66X

CGGCGAGCACTGGGCCGAGAGCGTGGGCTA(C/A)CTGGAGATCAGCCTGCGGCTGCACCGCTTG

[NM_006371.4](http://www.ncbi.nlm.nih.gov/entrez/viewer.fcgi?db=nucleotide&id=NM_006371.4): c.200T>C; [NP_006362.1](http://www.ncbi.nlm.nih.gov/entrez/viewer.fcgi?db=nucleotide&id=NP_006362.1): p.L67P

GCGAGCACTGGGCCGAGAGCGTGGGCTACC(T/C)GGAGATCAGCCTGCGGCTGCACCGCTTGCT

[NM_006371.4](http://www.ncbi.nlm.nih.gov/entrez/viewer.fcgi?db=nucleotide&id=NM_006371.4): c.469A>G; [NP_006362.1](http://www.ncbi.nlm.nih.gov/entrez/viewer.fcgi?db=nucleotide&id=NP_006362.1): p.K157E

CCCTACAAGTTCCTGCAGTTCGCTTACTTC(A/G)AGgcaagtccgcctcgccccgtcccaggcc

[NM_006371.4](http://www.ncbi.nlm.nih.gov/entrez/viewer.fcgi?db=nucleotide&id=NM_006371.4): c.826C>T; [NP_006362.1](http://www.ncbi.nlm.nih.gov/entrez/viewer.fcgi?db=nucleotide&id=NP_006362.1): p.Q276X

CATTATGTAGAAGTTCTGGAATGCAAAATA(C/T)AGTGTGAAGAGAACCTCACCCCAGTTATAG

**Splicing**

[NM_006371.4](http://www.ncbi.nlm.nih.gov/entrez/viewer.fcgi?db=nucleotide&id=NM_006371.4): c.472-1021C>G

ccaggaatccagataatgaatgacacacag(c/g)tgggtcccccgaggatcatagtctgatggg

[NM_006371.4](http://www.ncbi.nlm.nih.gov/entrez/viewer.fcgi?db=nucleotide&id=NM_006371.4): c.471+1G>C

TACAAGTTCCTGCAGTTCGCTTACTTCAAG(g/c)caagtccgcctcgccccgtcccaggccccg

[NM_006371.4](http://www.ncbi.nlm.nih.gov/entrez/viewer.fcgi?db=nucleotide&id=NM_006371.4): c.471+2C>A

ACAAGTTCCTGCAGTTCGCTTACTTCAAGg(c/a)aagtccgcctcgccccgtcccaggccccgg

[NM_006371.4](http://www.ncbi.nlm.nih.gov/entrez/viewer.fcgi?db=nucleotide&id=NM_006371.4): c.923-2A>G

tttcttcatttgtcttttcttcccaaccct(a/g)gTGAACGACCTGAAGAATGCAGCCCCCTGT

**Small deletions**

[NM_006371.4](http://www.ncbi.nlm.nih.gov/entrez/viewer.fcgi?db=nucleotide&id=NM_006371.4): c.24_31delCGCGGCGC

gggcgcgATGGAGCCGGGGCGCCGGGGGGC(CGCGGCGC/-)TGCTAGCGCTGCTGTGCGTGGCCTGCGCGC

[NM_006371.4](http://www.ncbi.nlm.nih.gov/entrez/viewer.fcgi?db=nucleotide&id=NM_006371.4): c.404delG

GCCTGCCAGCCTTCCGCCAGTCCCAGCCCA(G/-)CCGCGAGGTGCTGGCGGACTTCCAGCGCCG

[NM_006371.4](http://www.ncbi.nlm.nih.gov/entrez/viewer.fcgi?db=nucleotide&id=NM_006371.4): c.804_809delAGAAGT

atttatatgttttgtttcagATCATTATGT(AGAAGT/-)TCTGGAATGCAAAATACAGTGTGAAGAGAA

[NM_006371.4](http://www.ncbi.nlm.nih.gov/entrez/viewer.fcgi?db=nucleotide&id=NM_006371.4): c.879delT

AGTTATAGGAGGCTATCCGGTTGAGAAATT(T/-)GTGGCTACCATGTATCATTACTTGCAGTTT

**Small insertions**

[NM_006371.4](http://www.ncbi.nlm.nih.gov/entrez/viewer.fcgi?db=nucleotide&id=NM_006371.4): c.21_22dupGG

cgggcgcgATGGAGCCGGGGCGCCGGGGGG(-/gg)CCGCGGCGCTGCTAGCGCTGCTGTGCGTGG

[NM_006371.4](http://www.ncbi.nlm.nih.gov/entrez/viewer.fcgi?db=nucleotide&id=NM_006371.4): c.278_293dupAGCCCGAGCCCGCCGC

CAGCGCCGCGCCGCAGCCCGAGCCCGCCGC(-/agcccgagcccgccgc)CGGCCTCGCCAGCTATCCCGAGCTGCGCCT

**Small indels**

[NM_006371.4](http://www.ncbi.nlm.nih.gov/entrez/viewer.fcgi?db=nucleotide&id=NM_006371.4): c.822_826delAATACinsT

agATCATTATGTAGAAGTTCTGGAATGCAA(AATAC/t)AGTGTGAAGAGAACCTCACCCCAGTTATAG
