# Supplementary material for: Development of a High-Throughput Resequencing Array for the Detection of Pathogenic Mutations in Osteogenesis Imperfecta
Source: PLoS One. 2015 Mar 5;10(3):e0119553. doi: 10.1371/journal.pone.0119553 (PMC4350936; doi:10.1371/journal.pone.0119553)
Supplement: S3 Datasets — According to the selected mutations, we have made them into the probes to tiles on the chip. (ZIP) [file pone.0119553.s003.zip › FKBP10.doc]

**Missense/nonsense**

[NM_021939.3](http://www.ncbi.nlm.nih.gov/entrez/viewer.fcgi?db=nucleotide&id=NM_021939.3): c.344G>A; [NP_068758.3](http://www.ncbi.nlm.nih.gov/entrez/viewer.fcgi?db=nucleotide&id=NP_068758.3): p.R115Q

GCCTCATGGGCATGTGTGTCAACGAGCGGC(G/A)ACGCCTCATTGTGCCTCCCCACCTGGGCTA

[NM_021939.3](http://www.ncbi.nlm.nih.gov/entrez/viewer.fcgi?db=nucleotide&id=NM_021939.3): c.1207C>T; [NP_068758.3](http://www.ncbi.nlm.nih.gov/entrez/viewer.fcgi?db=nucleotide&id=NP_068758.3): p.R403X

AATGAGACCACCAAGCTTGGGGACTTTGTT(C/T)GATACCATTACAACTGTTCTTTGCTGGACG

**Splicing**

[NM_021939.3](http://www.ncbi.nlm.nih.gov/entrez/viewer.fcgi?db=nucleotide&id=NM_021939.3): c.1399+1G>A

CCCCGCACCTGGCCCACGGGGAGAGTGGAG(g/a)tgaggggctgagaccataatcttttttttt

**Small deletions**

[NM_021939.3](http://www.ncbi.nlm.nih.gov/entrez/viewer.fcgi?db=nucleotide&id=NM_021939.3): c.600_604delTTATG

cattttctgcagCTACAGTAAGGGCGGCAC(TTATG/-)ACACCTACGTCGGCTCTGGTTGGCTGATCA

**Small insertions**

[NM_021939.3](http://www.ncbi.nlm.nih.gov/entrez/viewer.fcgi?db=nucleotide&id=NM_021939.3): c.743dupC

tatcccttccccagGGACAGTGATCCCCCC(-/c)ACAGGCCTCGCTGGTCTTTCACGTCCTCCT

[NM_021939.3](http://www.ncbi.nlm.nih.gov/entrez/viewer.fcgi?db=nucleotide&id=NM_021939.3): c.831dupC

GTCCAGCTAGAGACGCTGGAGCTCCCCCCC(-/c)GGCTGTGTCCGCAGAGCCGGGGCCGGGGAC

[NM_021939.3](http://www.ncbi.nlm.nih.gov/entrez/viewer.fcgi?db=nucleotide&id=NM_021939.3): c.1016_1023dupGGAGAATT

GGTGCCTGCATGGGGGAACGCCGGAGAATT(-/ggagaatt)ACCATCCCCCCGCACCTCGCCTATGGGGAG

[NM_021939.3](http://www.ncbi.nlm.nih.gov/entrez/viewer.fcgi?db=nucleotide&id=NM_021939.3): c.1276dupC

tctcccccagGCATGACTACGGGGCCCCCC(-/c)AGGAGGCGACTCTCGGGGCCAACAAGGTGA
