# Supplementary material for: Development of a High-Throughput Resequencing Array for the Detection of Pathogenic Mutations in Osteogenesis Imperfecta
Source: PLoS One. 2015 Mar 5;10(3):e0119553. doi: 10.1371/journal.pone.0119553 (PMC4350936; doi:10.1371/journal.pone.0119553)
Supplement: S3 Datasets — According to the selected mutations, we have made them into the probes to tiles on the chip. (ZIP) [file pone.0119553.s003.zip › LEPRE1.doc]

**Missense/nonsense**

[NM_022356.3](http://www.ncbi.nlm.nih.gov/entrez/viewer.fcgi?db=nucleotide&id=NM_022356.3): c.392C>A; [NP_071751.3](http://www.ncbi.nlm.nih.gov/entrez/viewer.fcgi?db=nucleotide&id=NP_071751.3): p.S131X

GCCGCTGCCTCGGGCCGCCGGCCGCCCACT(C/A)GCTCAGCGAAGAGATGGAGCTGGAGTTCCG

[NM_022356.3](http://www.ncbi.nlm.nih.gov/entrez/viewer.fcgi?db=nucleotide&id=NM_022356.3): c.628C>T; [NP_071751.3](http://www.ncbi.nlm.nih.gov/entrez/viewer.fcgi?db=nucleotide&id=NP_071751.3): p.R210X

agtgctccttcttcccagtagCAAGAATTT(C/T)GACTGGGAGTGCGACTCTACTCAGAGGAAC

[NM_022356.3](http://www.ncbi.nlm.nih.gov/entrez/viewer.fcgi?db=nucleotide&id=NM_022356.3): c.640C>T; [NP_071751.3](http://www.ncbi.nlm.nih.gov/entrez/viewer.fcgi?db=nucleotide&id=NP_071751.3): p.R214X

tcccagtagCAAGAATTTCGACTGGGAGTG(C/T)GACTCTACTCAGAGGAACAGCCACAGGAAG

[NM_022356.3](http://www.ncbi.nlm.nih.gov/entrez/viewer.fcgi?db=nucleotide&id=NM_022356.3): c.765C>A; [NP_071751.3](http://www.ncbi.nlm.nih.gov/entrez/viewer.fcgi?db=nucleotide&id=NP_071751.3): p.Y255X

CTGCGAAGGGCCCTATGACTACGATGGCTA(C/A)AACTACCTTGAGTACAACGCTGACCTCTTC

[NM_022356.3](http://www.ncbi.nlm.nih.gov/entrez/viewer.fcgi?db=nucleotide&id=NM_022356.3): c.933C>G; [NP_071751.3](http://www.ncbi.nlm.nih.gov/entrez/viewer.fcgi?db=nucleotide&id=NP_071751.3): p.Y311X

ATCGCATTATAATTATCTGCAGTTTGCCTA(C/G)TATAACAgtaaggcctactttccttcctct

[NM_022356.3](http://www.ncbi.nlm.nih.gov/entrez/viewer.fcgi?db=nucleotide&id=NM_022356.3): c.1102C>T; [NP_071751.3](http://www.ncbi.nlm.nih.gov/entrez/viewer.fcgi?db=nucleotide&id=NP_071751.3): p.R368X

taccctcagAGTGCCAAGGAGTACCGACAG(C/T)GAAGCCTACTGGAAAAAGAACTGCTTTTCT

[NM_022356.3](http://www.ncbi.nlm.nih.gov/entrez/viewer.fcgi?db=nucleotide&id=NM_022356.3): c.1466T>C; [NP_071751.3](http://www.ncbi.nlm.nih.gov/entrez/viewer.fcgi?db=nucleotide&id=NP_071751.3): p.L489P

CTGACCACGAGTGTCAGGAGCTGCAGAGAC(T/C)GACCAATgtaagggaagcccagtgactgca

[NM_022356.3](http://www.ncbi.nlm.nih.gov/entrez/viewer.fcgi?db=nucleotide&id=NM_022356.3): c.1656C>A; [NP_071751.3](http://www.ncbi.nlm.nih.gov/entrez/viewer.fcgi?db=nucleotide&id=NP_071751.3): p.Y552X

GGAGAAGGTGCGGCGCATCATGGAGTCCTA(C/A)TTCCGCCTGGATACGCCCCTCTACTTTTCC

[NM_022356.3](http://www.ncbi.nlm.nih.gov/entrez/viewer.fcgi?db=nucleotide&id=NM_022356.3): c.2041C>T; [NP_071751.3](http://www.ncbi.nlm.nih.gov/entrez/viewer.fcgi?db=nucleotide&id=NP_071751.3): p.R681X

GCCATCGCCCTGTGGTTCACCCTGGACCCT(C/T)GACACAGCGAGCGGgtgagagcagctcgag

**Splicing**

[NM_022356.3](http://www.ncbi.nlm.nih.gov/entrez/viewer.fcgi?db=nucleotide&id=NM_022356.3): c.1080+1G>T

GAACACACCAGATCCATCGGCCCCCGTGAG(g/t)tgagagacttccttctgttatgacagctgc

[NM_022356.3](http://www.ncbi.nlm.nih.gov/entrez/viewer.fcgi?db=nucleotide&id=NM_022356.3): c.1170+2T>A

ATGTTTTTGGAATTCCCTTTGTGGATCCGg(t/a)gagctagggtacaggagccggaagggagcg

[NM_022356.3](http://www.ncbi.nlm.nih.gov/entrez/viewer.fcgi?db=nucleotide&id=NM_022356.3): c.1170+5G>C

TTTTTGGAATTCCCTTTGTGGATCCGgtga(g/c)ctagggtacaggagccggaagggagcgggg

[NM_022356.3](http://www.ncbi.nlm.nih.gov/entrez/viewer.fcgi?db=nucleotide&id=NM_022356.3): c.1473+1G>T

GAGTGTCAGGAGCTGCAGAGACTGACCAAT(g/t)taagggaagcccagtgactgcaccttcccc

[NM_022356.3](http://www.ncbi.nlm.nih.gov/entrez/viewer.fcgi?db=nucleotide&id=NM_022356.3): c.1473+3A>C

GTGTCAGGAGCTGCAGAGACTGACCAATgt(a/c)agggaagcccagtgactgcaccttccccca

[NM_022356.3](http://www.ncbi.nlm.nih.gov/entrez/viewer.fcgi?db=nucleotide&id=NM_022356.3): c.1915-1G>A

tgcaacccccctccctttctgtccccctta(g/a)GCAGAGGTGCAGCCTCAGTGTGGAAGAGCC

[NM_022356.3](http://www.ncbi.nlm.nih.gov/entrez/viewer.fcgi?db=nucleotide&id=NM_022356.3): c.2055+18G>A

ACACAGCGAGCGGgtgagagcagctcgagc(g/a)ggtgagagcagctggtgctgtggtgacccg

**Small deletions**

[NM_022356.3](http://www.ncbi.nlm.nih.gov/entrez/viewer.fcgi?db=nucleotide&id=NM_022356.3): c.232delC

CTCCGCGCCCTTCGCCTGCGCTGCCGCACC(C/-)AGTGTGCCGCCGACTTCCCGTGGGAGCTGG

[NM_022356.3](http://www.ncbi.nlm.nih.gov/entrez/viewer.fcgi?db=nucleotide&id=NM_022356.3): c.401_402delAA

TCGGGCCGCCGGCCGCCCACTCGCTCAGCG(AA/-)GAGATGGAGCTGGAGTTCCGCAAGCGGAGC

[NM_022356.3](http://www.ncbi.nlm.nih.gov/entrez/viewer.fcgi?db=nucleotide&id=NM_022356.3): c.484delG

cttcattcttagATCAACAAGTTGGAGAAA(G/-)CTGTTGCTGCAGCACACACCTTCTTCGTGG

[NM_022356.3](http://www.ncbi.nlm.nih.gov/entrez/viewer.fcgi?db=nucleotide&id=NM_022356.3): c.747delC

TGAGGAGTGCCGTGCCCTCTGCGAAGGGCC(C/-)TATGACTACGATGGCTACAACTACCTTGAG

**Small insertions**

[NM_022356.3](http://www.ncbi.nlm.nih.gov/entrez/viewer.fcgi?db=nucleotide&id=NM_022356.3): c.1383_1389dupGAACTCC

TATGAAGGCATCAGTCTCACCATGAACTCC(-/gaactcc)AAACTCCTGAATGGTTCCCAGCGGGTGGTG

[NM_022356.3](http://www.ncbi.nlm.nih.gov/entrez/viewer.fcgi?db=nucleotide&id=NM_022356.3): c.1675dupC

TGGAGTCCTACTTCCGCCTGGATACGCCCC(-/c)TCTACTTTTCCTACTCTCATCTGGTGTGCC

[NM_022356.3](http://www.ncbi.nlm.nih.gov/entrez/viewer.fcgi?db=nucleotide&id=NM_022356.3): c.2154dupC

CAGCCCCTGGATGCCCAGCAGGGTCCCCCC(-/c)GAACCTGCACAAGAGTCTCTCTCAGGCAGT

**Small indels**

[NM_022356.3](http://www.ncbi.nlm.nih.gov/entrez/viewer.fcgi?db=nucleotide&id=NM_022356.3): c.1365_1366delAGinsC

ttgctttgcagGTGGCCCCCTGCTGTATGA(AG/c)GCATCAGTCTCACCATGAACTCCAAACTCC
